# Significant variation of filamentation phenotypes in clinical *Candida albicans* strains

**DOI:** 10.3389/fcimb.2023.1207083

**Published:** 2023-10-20

**Authors:** Nichole D. Brandquist, Cierra Lampman, Elias J. Smith, Lizeth Basilio, Akram Almansob, Peter C. Iwen, Jill R. Blankenship

**Affiliations:** ^1^ Department of Biology, University of Nebraska at Omaha, Omaha, NE, United States; ^2^ Nebraska Public Health Laboratory, University of Nebraska Medical Center, Omaha, NE, United States

**Keywords:** *Candida albicans*, filamentation, hyphal morphogenesis, clinical strains, phylogeny

## Abstract

**Introduction:**

*Candida albicans* is an opportunistic human pathogen that typically resides as part of the microbiome in the gastrointestinal and genitourinary tracts of a large portion of the human population. This fungus lacks a true sexual cycle and evolves in a largely clonal pattern. The ability to cause disease is consistent across the species as strains causing systemic infections appear across the known *C. albicans* intra-species clades.

**Methods:**

In this work, strains collected from patients with systemic *C. albicans* infections isolated at the Nebraska Medicine clinical laboratory were typed by MLST analysis. Since the ability to form filaments has been linked to pathogenesis in *C. albicans*, these clinical strains, as well as a previously genotyped set of clinical strains, were tested for their ability to filament across a variety of inducing conditions.

**Results:**

Genotyping of the clinical strains demonstrated that the strains isolated at one of the major medical centers in our region were as diverse as strains collected across the United States. We demonstrated that clinical strains exhibit a variety of filamentation patterns across differing inducing conditions. The only consistent pattern observed in the entire set of clinical strains tested was an almost universal inability to filament in standard solid inducing conditions used throughout the *C. albicans* field. A different solid filamentation assay that produces more robust filamentation profiles from clinical strains is proposed in this study, although not all strains expected to filament *in vivo* were filamentous in this assay.

**Discussion:**

Our data supports growing evidence that broad phenotypic diversity exists between the *C. albicans* type strain and clinical strains, suggesting that the type strain poorly represents filamentation patterns observed in most clinical isolates. These data further highlight the need to use diverse clinical strains in pathogenesis assays.

## Introduction

*Candida albicans* is a human pathogenic fungus that is also a common commensal yeast in the human gastrointestinal flora and genitourinary mucosa. Infections can range from superficial to systemic. It is hypothesized that homeostasis between the fungus and the human host is disrupted under conditions where the immune system is compromised, and that this disruption allows *C. albicans* to invade the mucosal barrier, leading to candidiasis and candidemia. *Candida* infections are the fourth-leading cause of nosocomial infections, with *C. albicans* infections responsible for more than half of those infections. Mortality associated with invasive candidiasis is approximately 40% despite aggressive antifungal intervention (Pfaller and Diekema, 2007).

There are roughly 18 distinct genetic clades in *C. albicans.* Systemic infections are generally linked to 5 major clades (1, 2, 3, 4, and 11). With a lack of a full sexual cycle, intermixing between clades is rare (Soll and Pujol, 2003; Lott et al., 2005; Bougnoux et al., 2008). As a result of their independent evolution, the strains in each clade have distinct genetic backgrounds, and these differences have consequences for antifungal drug susceptibility and pathogenesis. Specifically, resistance to the antifungal drug flucytosine is most common in clade 1. The resistant strains within clade 1 have a common amino acid mutation in the product of the *FUR1* gene. However, strains within other clades that present with flucytosine resistance lack this mutation (Dodgson et al., 2004). Prior research has shown that varying genetic backgrounds exhibit phenotypic differences as well (Thewes et al., 2008; Hirakawa et al., 2015; Cavalieri et al., 2017; Smith and Hickman, 2020). However, *C. albicans* research has continued to focus largely on strains derived from a single strain, clade 1 strain SC5314, a clade that accounts for approximately 35% of *C. albicans* infections in the United States (Soll and Pujol, 2003; Jones et al., 2004; MacCallum et al., 2009).

Prior research indicated that *in vitro* filamentation phenotypes can vary between clinical strains (Hirakawa et al., 2015; Dunn et al., 2020). However, both yeast-like and filamentous cells appear to be required for invasive candidiasis. Cells trapped as either yeast or filaments are incapable of establishing infections *in vivo*, which indicates that the ability of *C. albicans* to transition between cell types is important for pathogenesis (Saville et al., 2003; Noble et al., 2017; Wakade et al., 2021). Filamentation is a complex process that is induced by a variety of environmental factors including pH, temperature, and CO_2_. As a field, we use a variety of *in vitro* conditions to mimic these environmental factors and induce filamentation. Prior studies in our lab have shown that filamentation media are not interchangeable and have vastly different effects on filamentation. These differences were both in the phenotypic morphology and the transcriptome (Azadmanesh et al., 2017). Thus, a multifactorial approach was used to study clinical strain filamentation in this work. Our goal was to determine whether there were consistent patterns of filamentation across clinical *C. albicans* strains.

In order to get a sense of the genetic backgrounds of *C. albicans* strains causing systemic infections in our region as well as their pathogenic traits, twenty clinical strains that had been detected in specimens from patients with systemic candidiasis at the Nebraska Medicine clinical laboratory at the University of Nebraska Medical Center (UNMC strains) were genotypically and phenotypically characterized. We observed that the UNMC strains showed a similar range of clades by MLST analysis as has been noted in larger studies of strains in the United States (Soll and Pujol, 2003; Odds et al., 2007). In addition, we tested these clinical strains, as well as a set of previously-typed clinical isolates (Hirakawa et al., 2015), for filamentation across a variety of inducing conditions. We expected that isolates from systemic infections would filament across most conditions and that we might see variation in filamentation phenotypes in strains that were not isolated from systemic infections. Instead, we saw a marked inability of most clinical strains to filament across standard solid inducing conditions. When these clinical strains were tested in a solid filamentation assay that was time-matched with liquid filamentation assays, we observed more robust filamentation. However, filamentation in solid media was not universal at this time point either, even though the tested strains were all from systemic infections. These observations strongly suggest that the type strain is not an ideal model for the species in filamentation assays and that the *C. albicans* field should revisit the filamentation phenotypic assays we use in drug development and pathogenesis studies.

## Materials and methods

### Strains and media

Deidentified strains from a collection of 20 C*. albicans* strains (**Table 1**) isolated from patients with systemic candidiasis (UNMC strains), 20 strains from a previously published work (Hirakawa et al., 2015), and the type strain SC5314 were used in this analysis. All strains were grown on yeast extract-peptone-dextrose (YPD), 10% FBS media with glucose (solid only), 10% FBS in YPD (liquid only), Lee’s media (Lee et al., 1975), RPMI media with 2.1 mM L-glutamine and buffered with 165 mM MOPs, and spider medium (10 g D-mannitol, 10 g nutrient broth, 2 g K_2_HPO_4_, in 1 L of H_2_O) in both liquid or on solid agar plates as previously described (Azadmanesh et al., 2017). For yeast-like cell growth, strains were grown in YPD at 30°C and for filamentous growth strains were grown at 37° C in 10% FBS, Lee’s, RPMI-MOPS, and spider media.

**Table 1:** MLST analysis of the UNMC clinical strains.

Strain^1^	*AAT1*	*ACC1*	*ADP1*	*MPIb^2^*	*SYA1* ^2^	*VPS13* ^2^	*ZWF1*	ST^2^	Clade^4^
B404-15	2	5	5	2	2	24	19	**3691**	1
B527-15	2	5	2	9	2	21	5	1174	1
B564-14	2	5	5	9	2	20	5	3063	1
B212-12	2	5	5	4	2	21	25	**3692**	1
B733-15	2	2	5	2	2	6	5	277	1
B1559-15	2	5	5	2	2	**313**	5	**3699**	1
B1257-15	36	7	6	4	215	24	20	**3700**	2
B808-15	35	4	4	4	215	4	239	**3693**	2
B1091-15	13	7	15	6	42	32	15	**3701**	3
B510-12	13	10	15	6	7	37	15	344	3
B2527-12	13	10	15	6	7	49	15	**3702**	11
B1168-15	21	7	25	18	128	20	6	**3703**	3
B568-15	13	10	15	6	56	32	15	591	3
B421-15	13	13	15	6	7	32	15	**3694**	5
B687-15	35	7	38	22	**240**	**310**	29	**3695**	6
B1762-15	6	3	37	2	38	32	12	492	7
B1486-15	33	14	38	**176**	78	81	137	**3704**	8
B46-15	13	10	15	6	2	4	279	**3696**	17
B618-15	13	7	10	24	7	37	15	**3697**	17
B444-12	20	2	38	4	26	294	25	**3698**	S

^1.^Strain designations are from the UNMC strain collection.

^2.^Allele and ST designations bold text are unique to this paper.

^3.^ ST designations identify strains with allelic profiles that match Strain Types in the *C. albicans* MLST database.

^4.^ Clade assignments were determined by ST designation or dendrogram comparisons.

### Amplification of DNA for MLST analysis

Cells from a single colony of each of the UNMC strains were used to inoculate overnight YPD cultures. Genomic DNA was extracted from these cultures using the Smash and Grab method (Hoffman and Winston, 1987). DNA was measured by Nanodrop and all genomic DNA was diluted to a concentration of 250 ng/µL to proceed with PCR for Multilocus sequence typing (MLST) analysis.

Primers and housekeeping genes for MLST analysis in *C. albicans* were previously optimized by others in the field (**Table S1**) (Bougnoux et al., 2003). 250 ng of genomic DNA was used as a template and DNA for each gene was amplified using the Q5 polymerase (New England Biolabs). The amplification began with an initial denaturation at 98˚C for 3 minutes, followed by 30 cycles of denaturation at 98˚C for 10 seconds, annealing at 54˚C for 30 seconds, and extension at 72˚C for 1 minute, and completed with a final extension at 72˚C for 6 minutes.

Amplicons were confirmed by DNA gel eletrophoresis. The successful PCR products were then purified using a GeneJet PCR purification kit (Fisher Scientific). 1-2 ng of purified PCR product were submitted to the UNMC sequencing core for Sanger sequencing using the primers listed in **Table S1**.

### MLST analysis and clade designation

Consensus sequences were generated from the Sanger sequence results using Geneious Prime software (version 2022.2.2). The ends of the forward and reverse sequences were trimmed to the core sequence identified in previous work (Bougnoux et al., 2003; Odds and Jacobsen, 2008), and the sequences were analyzed for heterozygosity. Heterozygous bases were determined if two peaks were at least 50% of the height of the initial base call and there were only two peaks (Odds and Jacobsen, 2008). *De Novo* assembly with high sensitivity and slow assembly was used to generate the final consensus sequence.

The consensus sequence for each gene in each strain was then entered into the MLST database (Jolley et al., 2018) to determine if the sequence was a known allele within the database. For strains with heterozygous alleles, the heterozygous genes were entered into the database using the degenerate nucleotide code to designate the heterozygous alleles. Novel allele sequences were trimmed to the MLST length and submitted for addition to the database. Allelic profiles for each strain were entered into the MLST database to compare against known strains.

In addition to the use of the MLST database for allele analysis, sequences for each gene were concatenated together for each clinical strain for phylogenetic analysis. Sequences were concatenated in the following order: *AAT1, AAC1, ADP1, MPIb, SYA1, VPS13*, and *ZWF1*. The concatenated sequences for the novel strains were compared to concatenated sequences from known reference strains (**Table S2**), and a dendrogram was generated using the unweighted pair group method with arithmetic mean in Geneious Prime.

Clinical strains were assigned a clade designation based on the reference strains they were clustering with in accordance with methods established by others (Tavanti et al., 2003; Bougnoux et al., 2004; Odds et al., 2007; Odds, 2010).

### Filamentation assays

Overnight cultures from a single colony were grown in 3 mL of YPD at 30°C, shaking at 200 rpm in a Benchmark Incu-Shaker 10L. The cells were washed twice in 1 mL of phosphate buffered saline (PBS) pH 7.4 and resuspended in 1 mL of PBS.

For liquid assays, 10µL of cell suspension was added to 2mL of pre-warmed media (10% FBS, Lee’s, RPMI, and Spider) in 35mm petri dishes with 14mm microwell glass bottom (MatTek Corporation) and incubated with shaking (100 rpm) at 37°C for 2 hours. 10µL of washed cells were added to a glass slide for control phenotypes. Cells were imaged on a Zeiss Axiovision fluorescence microscope at 20X magnification. Five images were collected from each assay. For the UNMC strains, each strain was tested in triplicate with 5 images collected for each replicate.

For traditional solid assays, 1µL of washed cell suspension was plated in triplicate on agar plates (YPD, 10% FBS, Lee’s, RPMI, and Spider). The plates were incubated for five days at 37°C, and colony edges were imaged at 4X on an Evos FL inverted microscope. Each strain was tested with 3-5 replicates, with one image per replicate. For the short filamentation assays, 10 µl of the washed cell suspension and 40 µl of autoclaved dH_2_0 were spread on the surface of pre-warmed agar plates (YPD, 10% FBS, Lee’s, RPMI, and Spider) using 4 mM glass beads. The plates were incubated at 37˚C for 3 hours and imaged at 10X on an Evos FL inverted microscope. Each strain was tested in triplicate, with 5 images from each replicate.

Strains from the solid assays were scored as previously described (Azadmanesh et al., 2017). Briefly, each strain was scored from 0-4 on the ability to form filaments compared to the control strain SC5314. A score of 0 correlated to abnormal morphology and a score of 4 indicated a morphology similar to the control. To obtain the average score for each strain and condition, all images were scored independently by 3-5 scorers. Computational methods were utilized to score liquid filamentation, as described in the supplemental methods. An average score of 2.5 or below indicated a severe phenotype defect.

## Results

### MLST analysis of clinical strains

MLST analysis was used to characterize the genetic relatedness of clinical *C. albicans* strains collected from patients with systemic infections at Nebraska Medicine. Fragments of the genes *AAT1, AAC1, ADP1, MPIb, SYA1, VPS13*, and *ZWF1* were amplified in order to compare allelic sequences to the *C. albicans* MLST database (Jolley et al., 2018). Exact matches for the allele sequences were found in the database for most gene fragments, except for 4 genes in 3 strains, which were added to the MLST database (**Table 1**, highlighted numbers). Strain B1559-15 had a novel allele sequence for *VPS13* and was assigned allele number 313 in the MLST database. B687-15 had novel allele sequences for both *SYA1* and *VPS13* and these were assigned allele numbers 240 and 310, respectively. Lastly, strain B1486-15 had a novel allele sequence for *MPIb* and was assigned 176 in the MLST database. For known allele sequences, the allele number in the MLST database was used to generate allelic profiles for each strain (**Table 1**).

**Figure 1:**
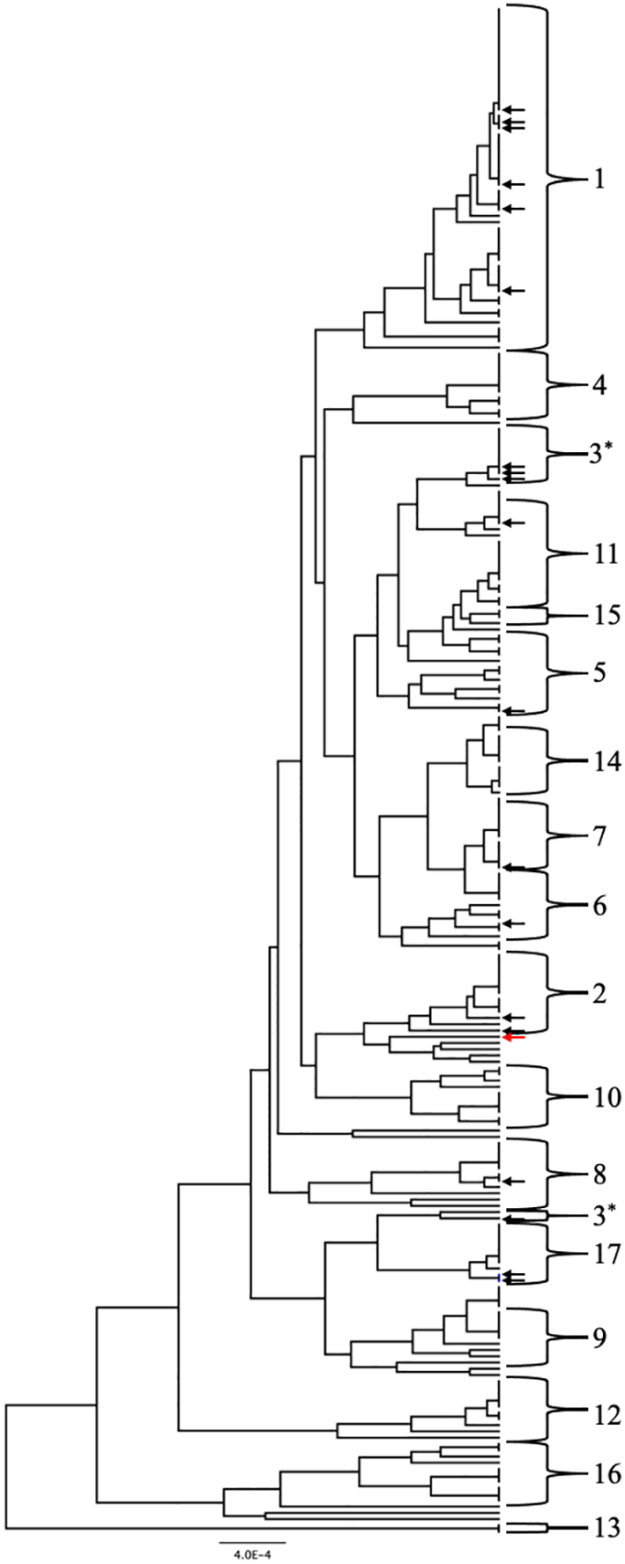
UPGMA dendrogram consisting of the 20 clinical isolates and 224 reference strains of *C. albicans*. *C. albicans* clades are indicated by the brackets. Arrows indicate location of the clinical isolates from the UNMC collection genotyped in this study. Black arrows are within known clades while the red arrow designates a singleton strain.

Two approaches were used to identify clade designations for the UNMC clinical strains. The allelic profiles for each strain were entered into the MLST database to search for known Strain Types (ST). Six strains in the set had STs with clade designations (**Table 1**, unhighlighted numbers). The allelic profiles of the remaining 14 strains were submitted to the MLST database. These strains were assigned unique strain ID number as well as STs upon addition to the database, which are indicated in **Table 1** (highlighted numbers). In addition, the UPGMA algorithm was used to generate an unrooted dendrogram using 20 clinical isolates (UNMC strains) and 224 representative reference strains to identify the clade designations of the unknown clinical isolates (**Figure 1**). The reference strains spanned a broad genetic background and included strains from all 17 clades and Singletons identified by Odds et al. (Odds and Jacobsen, 2008). Clades 1, 3, and 2 are represented by 30%, 15%, and 10% of the UNMC clinical isolates, respectively (**Figure 1**; **Table 1**). The clade 1 data is proportional to the 35% of infections from clade 1 seen nationally within the United States (Soll and Pujol, 2003).

### Filamentation analysis of clinical strains

The ability of *C. albicans* to filament has been linked to pathogenesis for systemic infections. However, there are a variety of assays used to measure filamentation, and genetic requirements for filamentation vary between conditions (Azadmanesh et al., 2017; Wang et al., 2021; Sharma et al., 2023). We therefore wanted to look at the filamentation profile of our clinical strains across a number of *in vitro* filamentation conditions. As a comparison, we also profiled filamentation in a clinical strain set that was previously sequenced, representing a variety of infection sites (Hirakawa et al., 2015). We hypothesized that strains collected from systemic infections would exhibit robust filamentation across the filamentation assays, mirroring the phenotype of the type strain SC5314, but that we might see variability in the filamentation phenotype of strains collected from other sites.

**Figure 2:**
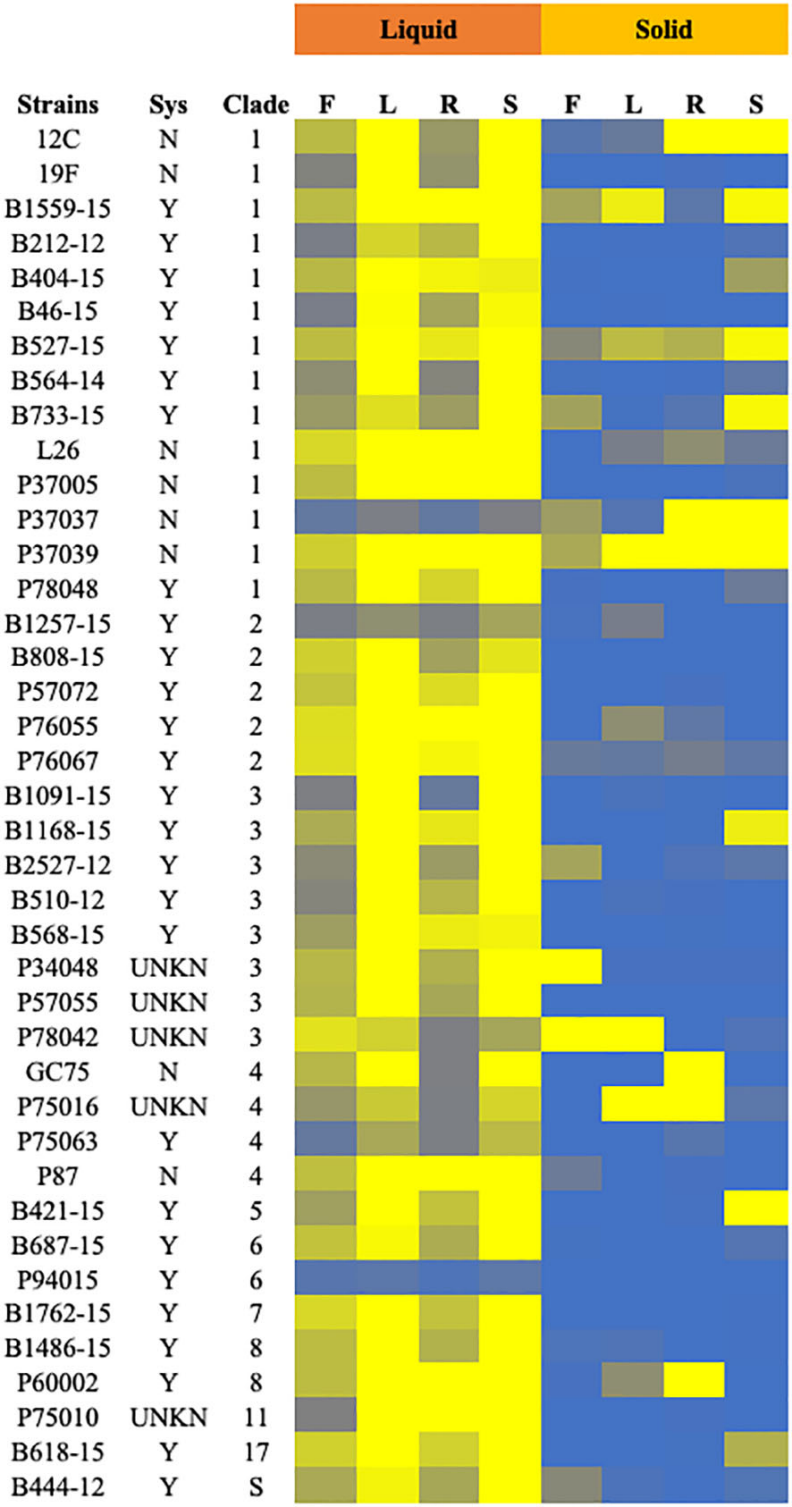
Filamentation phenotypes of the clinical isolates. Cells of the indicated strains were tested for filamentation in standard solid and liquid filamentation assays in FBS (F), Lee’s (L), RPMI (R), or spider (S) media. Cells that filamented like the type strain were given a score of 4 (yellow) while strains that did not filament were given a score of 0 (blue). Strains with scores below 2.5 were considered defective in filamentation. Strains labeled “Y” in the “Sys” column were isolated from systemic infections, while those labeled “N” were isolated from other infections or healthy patients. Strains labeled “UNKN” in the “SYS” column had an unknown origin. Clade identification of each strain is also indicated.

Clinical isolates and the control strain SC5314 were tested for filamentation across 8 inducing conditions in solid and liquid media as well as non-inducing solid and liquid conditions. Strains in solid media were scored from 0-4, with 0 representing no filamentation and a score of 4 representing filamentation as robust as the control strain. Strains in liquid media were scored computationally, normalized to the SC5314 filamentation score (set at 4) for each condition. Strains with filamentation scores of 2.5 or below in either liquid or solid media were considered defective in filamentation. Contrary to our hypothesis, both the systemic isolates from UNMC and the Hirakawa et al. strain set displayed variable filamentation profiles, which did not look different from non-systemic strains (**Figure 2A**; **Table S3**).

**Figure 3:**
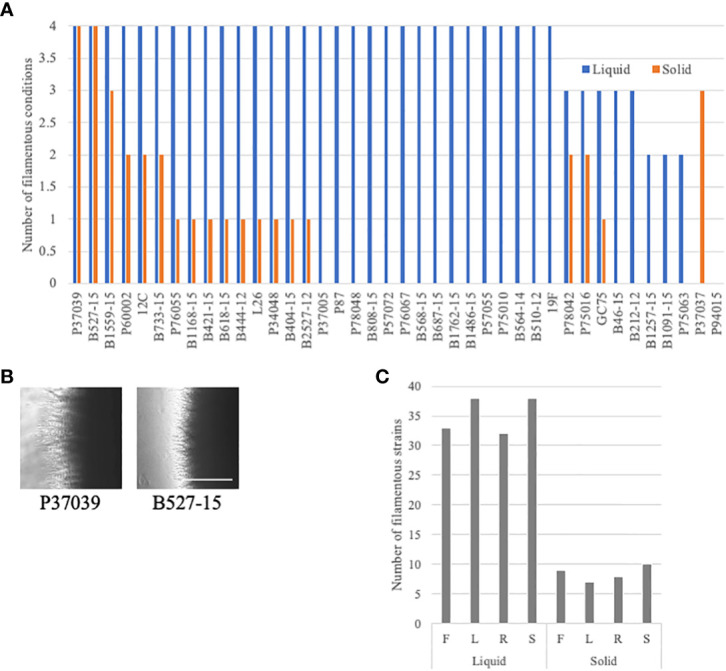
Additional filamentation observations in the clinical strains. **(A)** Clinical strains were generally defective in filamentation on solid media. The number of assays with filamentation scores above 2.5 in liquid (blue) or solid (orange) filamentation conditions was calculated for each strain. Absence of a bar indicates that the strain filamented in none of the conditions. **(B)** Cells that filament well in solid media are often hyperfilamentous. P37039 and B527-15 cells were grown on solid YPD media for 4-5 days. Images were taken at the edge of the colonies at 4X on an Evos microscope. The white bar represents 400 µm. **(C)** Clinical *C. albicans* strains clearly filament more robustly in liquid media. The number of strains with a filamentation score above 2.5 was calculated for each filamentation condition. Letters correspond to the letters used for filamentation assays in **Figure 2**.

Filamentation phenotypes varied between strains, but there were a few broad observations. Almost all of the clinical isolates of *C. albicans* exhibited severe filamentation defects on solid media, with only P37039 and B527-15 filamenting to some degree across all solid conditions (**Figures 2**, **3A**, **S2**). It was notable that these strains are hyperfilamentous on non-inducing solid media and also filamented across all liquid conditions (**Figures 2**, **3A, B**; **Table S3**). One other strain, P37037, also filamented on 3 of the 4 solid conditions (**Figures 2**, **3A, B**). All three of those strains are, like type-strain SC5314, members of Clade 1, although we did not find any correlation between clades in terms of filamentation phenotypes (**Figure S8**). There were limited representatives of each clade, however, and it is possible that clade-specific patterns might become apparent with a larger sample size. Filamentation was much more robust in liquid media, and indeed, a number of strains exhibited filamentation scores above 4 in Lee’s and Spider media (it should be noted that our solid scoring did not allow for scores above 4) (**Figure S5**). Only two strains, P37037 and P94015 were defective in filamentation in all liquid inducing conditions tested (**Figures 2**, **3A**). P94015 was unable to filament in any condition, while P37037, as mentioned above, was hyperfilamentous in non-inducing solid media and across 3 of 4 filamentation conditions.

When we focus on filamentation conditions rather than the strains, a few clear trends emerge. There is little variability between solid conditions in the number of strains that are considered filamentous in each condition, ranging from 7 to 10 strains (**Figure 3C**). The number of filamentatous strains in liquid filamentation conditions were much higher. Filamentation was more robust across strains in Lee’s and Spider media (38 strains with scores above 2.5) compared to FBS (33 strains) and RPMI (32 strains) (**Figure 3C**). The significance of this variation in score is unclear, but it is worthwhile to note.

### Solid filamentation at early timepoints

One thing that differentiates the standard liquid and solid filamentation assays in *C. albicans*, besides the solidity of the media, is the time between induction initiation and analysis of filamentation. Liquid filamentation is measured just a few hours after induction, while the standard solid filamentation assay is measured days after induction. Therefore, standard liquid assays determine initial response to induction in liquid media while standard solid assays measure sustained filamentation on solid surfaces. We found in previous work (Creger and Blankenship, 2018), that solid filamentation can be measured at the same timepoints used in liquid assays. We also found that two of the clinical strains tested above (P87 and P76067), which do not filament in standard solid filamentation assays, *do* filament at these earlier time points (Creger and Blankenship, 2018). By examining filamentation in solid media at the same timepoint that we examined liquid filamentation, we eliminated the confounding time factor in our comparisons. We hypothesized that clinical strains from systemic infections would filament in solid media at these earlier timepoints as robustly as they do in liquid filamentation conditions.

**Figure 4:**
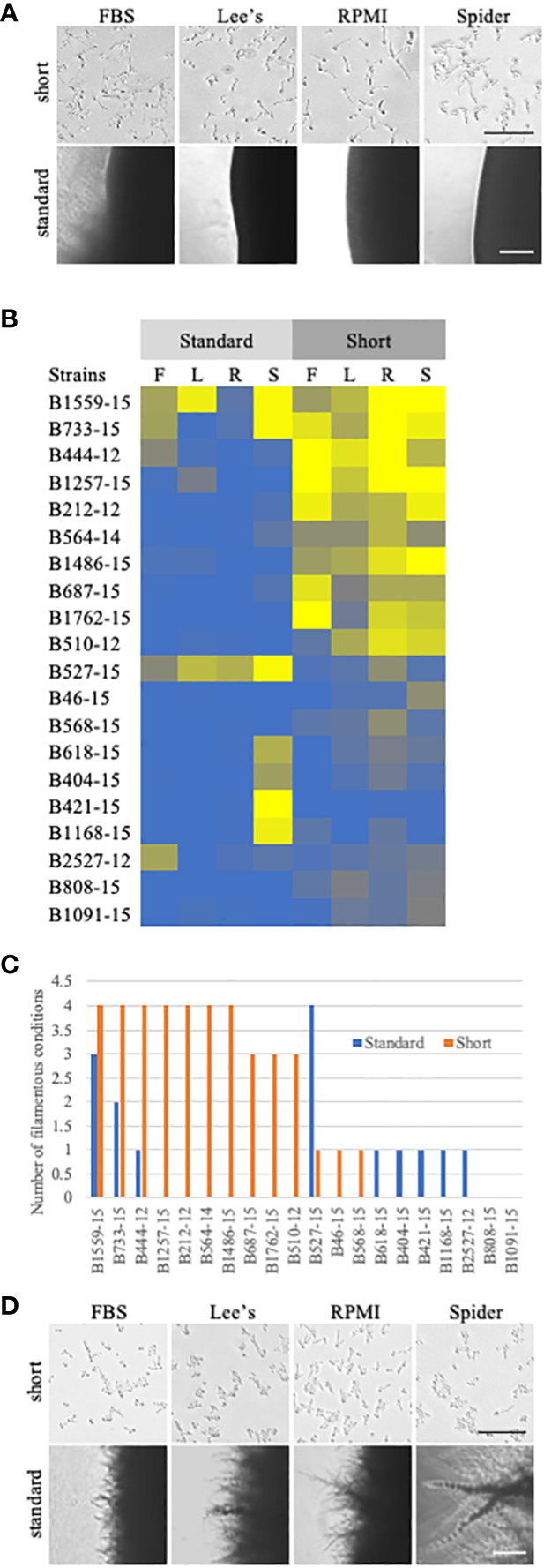
Comparison of standard solid filamentation assays and a shortened solid filamentation assay. **(A)** Respective images from standard and shortened solid filamentation assays for strain B444-12. The strain was grown in the indicated filamentation induction media for 4-5 days (standard) or for 3 hours (short) at 37˚C and imaged through the bottom of the agar plate on an Evos microscope at 4X (standard) or 10X (short). Bars in the images represent 100 µm for each figure set (short or standard). **(B)** A heat map of filamentation scores in standard and shortened solid filamentation assays. Cells of the indicated strains were tested for filamentation in standard and shortened solid filamentation assays in FBS (F), Lee’s (L), RPMI (R), or spider (S) media. Cells that filamented like the type strain were given a score of 4 (yellow) while strains that were afilamentous were given a score of 0 (blue). Strains with scores below 2.5 were considered defective in filamentation. **(C)** Some clinical strains *can* filament in solid media, but only in the shortened assay. The number of assays with filamentation scores above 2.5 in standard (blue) or shortened (orange) solid filamentation conditions was calculated for each strain. Absence of a bar indicates that the strain filamented in none of the conditions. **(D)** B527-15 filaments in the standard solid filamentation assay, but few cells were filamentous in the shortened assay. Respective images from standard and shortened solid filamentation assays for strain B527-15. The strain was grown in the indicated filamentation for 4-5 days (standard) or for 3 hours (short) and imaged on an Evos microscope at 4X (standard) or 10X (short). Bars in the images represent 100 µm for each figure set (short or standard).

For these shortened filamentation assays, we focused on filamentation in the UNMC set of clinical strains. These strains were all isolated from patients with systemic infections. The expectation was that all of these strains should be filamentatous *in vivo* and thus filamentous *in vitro* in assays that model *in vivo* induction. Seven of the clinical strains (B212-12, B444-12, B564-15, B733-15, B1257-15, B1486-15, and B1559-15) filamented across all solid conditions in this shortened solid filamentation assay and seven of the clinical strains (B404-15, B421-15, B618-15, B808-15, B1091-15, B1168-15, and B2527-12) had filamentation defects across all solid inducing media (**Figures 4A–C**, **S3**). Strain 527-15 was notable because it did filament in the standard solid assay but was defective in most shortened solid filamentation assays (**Figure 4D**). It is possible that this strain responds more slowly to filamentation signals or that it was growing, and thus filamenting, more slowly. Clearly, it can filament in solid media as evidenced in the longer assay (**Figures 2**, **4D**). The rest of the strains showed varying patterns of filamentation in the shortened assays. Overall, it appears that a number of the clinical strains *can* filament on solid media, but few are able to maintain that filamentation over a period of days.

## Discussion

We genotyped 20 clinical strains isolated from patients with systemic *Candida albicans* infections at Nebraska Medicine. Using MLST analysis, we identified 14 novel strains and 6 strains with genotypes matching strains already in the MLST database. In addition to the 14 novel strains identified, we also identified 4 novel allele sequences. Our analysis suggests that the infecting strains observed clinically in Nebraska are similar to what we observe across the US. Clade 1 accounts for 30% of infections in our sample and 35% of infections across the US. The second largest clade represented in our study was clade 3, which represented 15% of the isolates, while Clade 2 accounted for 10% of the UNMC strains. 4 of the 5 major clades were identified in the UNMC strains, and the absence of strains from clade 4 could be due to the small sample size of the study. Despite the small sample size, a broad distribution of clades was identified. In addition to the 3 major clades, several other clades (5-8, 11, 17) and a singleton were identified.

Although we observed no correlation between filamentation ability and clade, there were stark phenotypic difference between the liquid and traditional solid media filamentation assays. Most of the clinical strains we tested, including the set of strains from UNMC and a set of strains genotyped in the Hirakawa et al. study (Hirakawa et al., 2015), filamented in most liquid inducing media. Few, however, filamented well on solid inducing media using a standard assay. These stark differences were less apparent when examining solid and liquid filamentation at the same timepoint, although filamentation in solid media was still not robust for approximately half of the strains tested in those shortened assays. The assays used to examine phenotypes in *C. albicans* have largely been used to test derivatives of the type strain SC5314, but it is clear that SC5314 is an outlier in filamentation phenotypes on solid media, as has been noted by others (Dunn et al., 2020). Rather than calling most of the strains tested in our assays “defective” in traditional solid filamentation assays, it is more likely that SC5314 is particularly robust at maintenance of filamentation induction in solid media. This finding suggests that the traditional solid filamentation assays are not good models for filamentation in the species as a whole and would not be ideal for drug development.

One strain from our assay, P37037, deserves some discussion because its pattern of filamentation suggests that it responds to distinct signals for filamentation. This strain did not filament well in any liquid condition, but was filamentous in three of the four standard solid filamentation assays. These observations suggest that this strain does not respond to inducing nutrient signals in the media, but that surface contact in combination with inducing signals, inducing signals can trigger filamentation, with one exception. This strain may allow us to separate physical and nutrient filamentation cues. However, it should be noted that our results contrast with those recently published, in which P37037 had a strong filamentation defect in solid spider medium (Wang et al., 2021). P37037 is heterozygous at the *EFG1* locus, a gene that is an important regulator of filamentation, but undergoes spontaneous homozygosis (Wang et al., 2021). One allele of *EFG1* is functional and the other non-functional in this strain and others have shown that loss of heterozygosity and gene conversions can occur at this locus (Liang et al., 2019). The differing observations between our work and the work from Wang *el al* may be linked to homozygosity of the functional *EFG1* allele in our version of this strain, although we have not tested for this possibility.

In addition to the discrepancies with the P37037 data, we also want to note that there are differences between our results and results from the Hirakawa et al. and Dunn et al. studies, which overlapped the non-UNMC strains tested (Hirakawa et al., 2015; Dunn et al., 2020). Overall, we see much more robust filamentation in liquid RPMI than is noted for strains in Dunn et al. (Dunn et al., 2020). We hypothesize that methodology is the likely cause of these differences. In our liquid assays, we induce cells in glass bottom dishes with shaking and image directly from these dishes, while the Dunn et al. paper pipetted cells induced in culture tubes onto glass slides for observation. We generally see more than 90% of SC5314 cells filamenting in our glass-bottom assays compared to much lower numbers, overall, in the Dunn et al. paper. We see similar filamentation profiles in SC5314 filamentous cells using the same approach used in Dunn et al. (J. Blankenship, unpublished observations). There are also some differences in our solid assay results, although these assay show much greater similarity between studies. These discrepancies may come down to the scale of observation. The Dunn et al. and Hirakawa et al. papers look at a macroscopic view of whole colonies, measuring, in part, the length of filamentous-like growth from the central yeast-like colony. We look at a micro scale, observing the filaments on the edges of the colony and scoring for absence or presence of filaments from the edges of colonies (as well as how much of the colony is demonstrating filamentation). A strain may score low in the Dunn et al. assay if the strain filaments, but those filaments do not extend far from the main colony. If the filamentation of that strain, however, is robust around the whole colony, we would score it highly. There are other possible factors that could play a role in these differences, but these seem the most likely.

The ability of *C. albicans* cells to transition between filamentous and yeast-like forms has long been linked to virulence (Lo et al., 1997; Murad et al., 2001). In addition, filamentous cells express adhesins that allow them to penetrate and be internalized by the host cell, as well as the toxin, candidalysin, that can permeabilize host cells (Staab et al., 1999; Granger et al., 2005; Phan et al., 2007; Moyes et al., 2016). We hypothesized that *C. albicans* strains from systemic infections should filament across all filament inducing conditions, in a manner similar to the type strain, SC5314. Most of the systemic strains did filament in across liquid filamentation conditions. However, this study supports other work demonstrating systemic strains generally do not filament in standard solid filamentation assays. We observed that only half of the invasive candidiasis strains tested for solid filamentation at earlier timepoints filamented in 3 or more conditions. Additional studies are needed to better define the importance of filamentation for systemic infection and to define the *in vitro* assays that best mimic *in vivo* filamentation phenotype. It should be noted that we do not have information about severity of disease in the de-identified strains. It is quite possible that strains with filamentation defects *in vivo* can become systemic, but may be unable to cause significant disease.

Studying pathogenic phenotypes *in vivo* can be difficult and cost-prohibitive. Thus, many of us rely on *in vitro* assays that are meant to model *in vivo* conditions. Most of our knowledge about the pathways that help initiate and maintain filamentation is rooted in work performed in *in vitro* model systems, and in the SC5314 genetic background. In addition, some efforts are being made to develop anti-filamentation drugs for *C. albicans* treatments, relying largely on *in vitro* models of filamentation (Romo et al., 2018; MacAlpine et al., 2021). The observations described here and by others suggest that we must be careful in our reliance on *in vitro* models for filamentation and that the SC5314 genetic background is not the ideal background for filamentation studies.

## Data availability statement

The original contributions presented in the study are included in the article/**Supplementary Materials**, further inquiries can be directed to the corresponding author.

## Author contributions

NB and JB conceived and planned the experiments. NB, CL, ES, LB, and AA carried out the experiments. PI provided strains for the research. JB performed the computational analysis. NB and JB wrote the manuscript with input from PI and ES. JB supervised the research. All authors contributed to the article and approved the submitted version.

## Supplementary material

Supplementary Figure 1 Clinical strain filamentation in the standard liquid media assay. Cells of the indicated strains were tested for filamentation in standard and shortened solid filamentation assays in FBS, Lee’s, RPMI, or spider liquid media. Cells were grown overnight at 30˚C with shaking, washed, and then incubated in the inducing liquid media for 3 hours at 37˚C. Also shown are images from the overnight cells grown in YPD. The images shown are a representative example of images assessed bioinformatically due to file size limitations.

Supplementary Figure 2 Clinical strain filamentation in the standard solid media assay. Cells of the indicated strains were tested for filamentation in standard and shortened solid filamentation assays in FBS, Lee’s, RPMI, or spider liquid media. Cells were grown overnight at 30˚C with shaking, washed, and then spotted on inducing media agar plates and grown at 37˚C. Cells were also spotted on YPD agar media and grown at 30˚C. Colony edged were imaged after 4-5 days of incubation.

Supplementary Figure 3 Clinical strain filamentation in the shortened solid media assay. Cells of the indicated strains were tested for filamentation in standard and shortened solid filamentation assays in FBS, Lee’s, RPMI, or spider liquid media. Cells were grown overnight at 30˚C with shaking, washed, and then spread on inducing media agar plates and grown at 37˚C. Cells were also spread on YPD agar media and grown at 30˚C. Cells were imaged through the bottom of the agar plate after 3 hours of incubation. The images shown are a representative example of images assessed due to file size limitations.

Supplementary Table 1 Primer Sequences for gene amplification and Sanger Sequencing.

Supplementary Table 2 Reference strains and clade designations.

Supplementary Table 3 Filamentation scores of the clinical strains. ^1^F-FBS media, L- Lee’s Media, R- RPMI media, S-Spider media.
