# Supplementary material for: Significant variation of filamentation phenotypes in clinical Candida albicans strains
Source: Front Cell Infect Microbiol. 2023 Oct 20;13:1207083. doi: 10.3389/fcimb.2023.1207083 (PMC10623444; doi:10.3389/fcimb.2023.1207083)
Supplement: Supplementary file 1 [file DataSheet_1.zip › coding/Filscoring.pdf]

Filamentation differentiation

jrblankenship

2023-08-02

Loading the packages

In this first step, we will load the packages we'll need for the analysis later.

```
library(dplyr) #This one helps work with the data and is being use when you see the "%>%" commands
library(writexl) #This one can be used to export tables to Excel
library(readxl) #This one helps import Excel files to R
library(ggplot2) #This one helps you visualize the data with nice graphs
require(xgboost) #This helps build the prediction model and to calculate the predictions
require(Matrix) #This one helps you work with matrices that are essential for XGboost
require(data.table) #This one helps you work with data tables both pre- and post-boosting
if (!require('vcd')) install.packages('vcd') # I believe that this package is required for XGboost
```

Getting the Training/Test data into a table

The goal of these steps is to import the training and test data from the Excel files where I put them together.

As I put these together, the Train data included all the data from the first 3 SC5314 images in FBS (101-103) and the first 6 images of the SC5314 images in YPD (101-106). The Test data had all the data from the last two SC5314 images in FBS (104-106) and the last 4 images of SC5314 in YPD (107-110)

```
Train <- read_excel("/Users/jrblankenship/Desktop/Research/UNO/Graduate_projects/Nichole_Brandquist/Pape
Test <- read_excel("/Users/jrblankenship/Desktop/Research/UNO/Graduate_projects/Nichole_Brandquist/Paper
df_clinfil_train <- data.table(Train) #makes a dataframe out of the train data
df_clinfil_test <- data.table(Test) #makes a dataframe out of the test data
```

Testing the data to look for outliers

I am worried that the masking program is measuring some very small fragments that are not real cells. I will query the train - YPD data to see what it looks like. There should not be filaments in this data and I should be able to ID objects that are too small to be real. Based on this, I may be removing these extremely small artifacts.

```
YPD_composition <- df_clinfil_train[df_clinfil_train$Filamentous == 'FALSE',] # This should just pull t
p <- ggplot(df_clinfil_train, aes(x = Filamentous, y= AreaShape_Area)) + geom_violin() #This looks at a
p <- ggplot(df_clinfil_train, aes(x = Filamentous, y= AreaShape_EquivalentDiameter)) + geom_violin() #T
p <- ggplot(df_clinfil_train, aes(x = Filamentous, y= AreaShape_MinorAxisLength)) + geom_violin() #Look
```

In the end, I am not seeing a clear "too small" set of data that is obvious.

Getting ready to model

Matrices

The following code converts those Train and Test dataframes into matrices (reguired for XGboost).

```
mat_train <- as.matrix(df_clinfil_train) #converts the dataframe into a matrix
mat_test <- as.matrix(df_clinfil_test)
```

Building the model

This builds and tests a model using XGboost. We are using it because it has been used by others for C. albicans data. The outcome of the predictions of this model are a regression, with data values ranging from 0 (not filamentous) to 1 (fully filamentous). We should be able to use these numbers to score our strains in each condition. The regression values will likely need to be weighted for each condition (even FBS…).

```
#This builds a model
bst <- xgboost(data = mat_train[,2:49], label = mat_train[,"Filamentous"], max_depth = 4, eta = 1, nthre
# This at least appears to work to set up a model of the data, using data as columns 2-49 and the predi

pred <- predict(bst, mat_test[,2:49]) #This provides predictions for the testing data based on the mode

err <- mean(as.numeric(pred > 0.5) != mat_test[,"Filamentous"]) # This is where we test whether the pre

print(paste("test-error=", err)) # This allows you to see the error rates calculated above. It looks in

xgb.plot.importance(importance_matrix = importance_matrix) #This allows you to look at the measurements

xgb.save(bst, "liqfil_boost.model") #This saves the model as a universal binary document that can be re
```

Going live with real data

In this next setup, I'm going to using predictions to "score" the FBS data from the Hirakawa strains (CLIN strains). The CLIN strains appear to be missing some strains in FBS and were only done in technical replicates (different images from the same overnight). I need to see if there are additional images and find those missing strains. Note that the UNMC strains have technical and biological replicates.

```
CLIN_FBS <- read_excel("/Users/jrblankenship/Desktop/Research/UNO/Graduate_projects/Nichole_Brandquist/
df_clin_FBS <- data.table(CLIN_FBS) #converts the file to a data table.
mat_CLIN_FBS <- as.matrix(df_clin_FBS) #converts the file to a matrix for XGboost
bst <- xgboost::xgb.load('/Users/jrblankenship/Desktop/Research/UNO/Graduate_projects/Nichole_Brandquis
pred_clin_FBS <- predict(bst, mat_CLIN_FBS[,3:50]) #I apparently did not remove the object number from
plate_clin_FBS <-as.vector(mat_CLIN_FBS[,"ImageNumber"]) # Pulling out that image number as a vector fr
clin_FBS_data <- cbind(plate_clin_FBS, pred_clin_FBS) # putting the two together as a table.
clinFBSplateID <- read_excel("/Users/jrblankenship/Desktop/Research/UNO/Graduate_projects/Nichole_Brand
clin_FBS_data <- merge(clin_FBS_data,clinFBSplateID) #this merges the files and includes the cell count
colnames(clin_FBS_data)[2] = "pred" #changes that prediction column to a more universal name that can b

clin_FBS_avg <- clin_FBS_data %>%
    group_by(FileName_Original) %>%
```

```
    summarise(median_fil = median(pred), mean_fil = mean(pred), stdev_fil = sd(pred)) #this gives the m

clinFBSstrains <- read_excel("/Users/jrblankenship/Desktop/Research/UNO/Graduate_projects/Nichole_Brand

clin_FBS_avg <- merge(clin_FBS_avg, clinFBSstrains) #merges strain names with replicate name file. Agai

q <- ggplot(clin_FBS_avg, aes(x = Strain, y = mean_fil)) + geom_violin()
q
# This gives us a violin plot showing the data by strain.
q <- ggplot(clin_FBS_avg, aes(x = Strain, y = mean_fil)) + geom_boxplot()
q
#This gives a boxplot instead, which is a nice view.

clin_FBS_avg$weighted <-clin_FBS_avg$mean_fil/mean(clin_FBS_avg[101:105,3])*4 #This adds a weighting co

q <- ggplot(clin_FBS_avg, aes(x = Strain, y = weighted)) + geom_boxplot()
q
#this gives me a boplot of the values. Remember, that this is for differing images from the same overni

clin_FBS_strain <- clin_FBS_avg %>%
    group_by(Strain) %>%
    summarise(mean_mean = mean(mean_fil), stdev_fil = sd(mean_fil))
# This summarizes the mean of the replicates per strain
```

Clinical UNMC FBS data

```
UNMC_FBS <- read_excel("/Users/jrblankenship/Desktop/Research/UNO/Graduate_projects/Nichole_Brandquist/
df_UNMC_FBS <- data.table(UNMC_FBS) #makes it a dataframe
mat_UNMC_FBS <- as.matrix(df_UNMC_FBS) #converts it to a matrix for XGboost
bst <- xgboost::xgb.load('/Users/jrblankenship/Desktop/Research/UNO/Graduate_projects/Nichole_Brandquis
pred_UNMC_FBS <- predict(bst, mat_UNMC_FBS[,2:49]) #makes the predictions and the outcome is a matrix

#Now I need to pull out the plate number as a vector, put it together with the labels for the plates an
plate_UNMC_FBS <-as.vector(mat_UNMC_FBS[,"ImageNumber"]) # Pulling out that image number as a vector
UNMC_FBS_data <- cbind(plate_UNMC_FBS, pred_UNMC_FBS) # putting the two together as a table.
unmcFBSplateID <- read_excel("/Users/jrblankenship/Desktop/Research/UNO/Graduate_projects/Nichole_Brand
UNMC_FBS_data <- merge(UNMC_FBS_data,unmcFBSplateID) #this merges the files and also includes the cells
colnames(UNMC_FBS_data)[2] = "pred" #changes that prediction column to a more universal name

UNMC_FBS_avg <- UNMC_FBS_data %>%
    group_by(FileName_Original) %>%
    summarise(median_fil = median(pred), mean_fil = mean(pred), stdev_fil = sd(pred)) #this gives the m

UNMCFBSstrains <- read_excel("/Users/jrblankenship/Desktop/Research/UNO/Graduate_projects/Nichole_Brand
UNMC_FBS_data <- merge(UNMC_FBS_data,UNMCFBSstrains) #this adds replicate and strain names to the file

UNMC_FBS_reps <- UNMC_FBS_data %>%
    group_by(Replicate) %>%
    summarise(median_fil = median(pred), mean_fil = mean(pred), stdev_fil = sd(pred))
#This will get the averages per replicate
UNMC_FBS_reps <- merge(UNMC_FBS_reps,UNMCFBSstrains) #this adds the strain names back for the step two
```

```
p <- ggplot(UNMC_FBS_reps, aes(x = Strain, y = mean_fil)) + geom_boxplot()
p #this plots out the data as a boxplot (can do a violin plot instead)

UNMC_FBS_reps$weighted <-UNMC_FBS_reps$mean_fil/mean(clin_FBS_avg[101:105,3])*4 #This adds a weighting

q <- ggplot(UNMC_FBS_reps, aes(x = Strain, y = weighted)) + geom_boxplot() + theme(axis.text.x = element
q #that last piece with the theme was to get the X axis labels placed vertically

UNMC_FBS_strains <- UNMC_FBS_reps %>%
    group_by(Strain) %>%
    summarise(mean_score = median(weighted), stdev_score = sd(weighted))
#This gives the averages and standard deviation for the score values that could be used in a heatmap
```

RPMI data

Hirakawa first

```
CLIN_RPMI <- read_excel("/Users/jrblankenship/Desktop/Research/UNO/Graduate_projects/Nichole_Brandquist/
df_CLIN_RPMI <- data.table(CLIN_RPMI) #makes it a dataframe
mat_CLIN_RPMI <- as.matrix(df_CLIN_RPMI) #converts it to a matrix for XGboost
bst <- xgboost::xgb.load('/Users/jrblankenship/Desktop/Research/UNO/Graduate_projects/Nichole_Brandquist
pred_CLIN_RPMI <- predict(bst, mat_CLIN_RPMI[,2:49]) #makes the predictions and the outcome is a matrix

#Now I need to pull out the plate number as a vector, put it together with the labels for the plates an
plate_CLIN_RPMI <-as.vector(mat_CLIN_RPMI[,"plate_CLIN_RPMI"]) # Pulling out that image number as a vec
CLIN_RPMI_data <- cbind(plate_CLIN_RPMI, pred_CLIN_RPMI) # putting the two together as a table.
clinRPMIplateID <- read_excel("/Users/jrblankenship/Desktop/Research/UNO/Graduate_projects/Nichole_Bran
CLIN_RPMI_data <- merge(CLIN_RPMI_data,clinRPMIplateID) #this merges the files and also includes the ce
colnames(CLIN_RPMI_data)[2] = "pred" #changes that prediction column to a more universal name

CLIN_RPMI_avg <- CLIN_RPMI_data %>%
    group_by(FileName_Original) %>%
    summarise(median_fil = median(pred), mean_fil = mean(pred), stdev_fil = sd(pred)) #this gives the m

CLINRPMIstrains <- read_excel("/Users/jrblankenship/Desktop/Research/UNO/Graduate_projects/Nichole_Bran
CLIN_RPMI_avg <- merge(CLIN_RPMI_avg,CLINRPMIstrains) #this adds replicate and strain names to the file

CLIN_RPMI_avg$weighted <-CLIN_RPMI_avg$mean_fil/mean(CLIN_RPMI_avg[108:112,3])*4 #This adds a weighting

write_xlsx(CLIN_RPMI_avg,"/Users/jrblankenship/Desktop/Research/UNO/Graduate_projects/Nichole_Brandquis


p <- ggplot(CLIN_RPMI_avg, aes(x = Strain, y = weighted)) + geom_boxplot() + theme(axis.text.x = element
p #this plots out the data as a boxplot (can do a violin plot instead)

CLIN_RPMI_strains <- CLIN_RPMI_avg %>%
    group_by(Strain) %>%
    summarise(mean_score = mean(weighted), stdev_score = sd(weighted))
```

```
library("writexl")
write_xlsx(CLIN_RPMI_strains,"/Users/jrblankenship/Desktop/Research/UNO/Graduate_projects/Nichole_Brando
```

RPMI UNMC strains

```
UNMC_RPMI <- read_excel("/Users/jrblankenship/Desktop/Research/UNO/Graduate_projects/Nichole_Brandquist/
df_UNMC_RPMI <- data.table(UNMC_RPMI) #makes it a dataframe
mat_UNMC_RPMI <- as.matrix(df_UNMC_RPMI) #converts it to a matrix for XGboost
bst <- xgboost::xgb.load('/Users/jrblankenship/Desktop/Research/UNO/Graduate_projects/Nichole_Brandquis
pred_UNMC_RPMI <- predict(bst, mat_UNMC_RPMI[,2:49]) #makes the predictions and the outcome is a matrix

#Now I need to pull out the plate number as a vector, put it together with the labels for the plates an
plate_UNMC_RPMI <-as.vector(mat_UNMC_RPMI[,"ImageNumber"]) # Pulling out that image number as a vector
UNMC_RPMI_data <- cbind(plate_UNMC_RPMI, pred_UNMC_RPMI) # putting the two together as a table.
unmc_RPMIplateID <- read_excel("/Users/jrblankenship/Desktop/Research/UNO/Graduate_projects/Nichole_Bra
UNMC_RPMI_data <- merge(UNMC_RPMI_data,unmc_RPMIplateID) #this merges the files and also includes the c
colnames(UNMC_RPMI_data)[2] = "pred" #changes that prediction column to a more universal name

UNMC_RPMI_avg <- UNMC_RPMI_data %>%
    group_by(FileName_Original) %>%
    summarise(median_fil = median(pred), mean_fil = mean(pred), stdev_fil = sd(pred)) #this gives the m

UNMC_RPMI_avg <- merge(UNMC_RPMI_avg, unmc_RPMIplateID)

UNMC_RPMI_reps <- UNMC_RPMI_avg %>%
    group_by(Replicate) %>%
    summarise(mean_mean = mean(mean_fil), stdev_mean = sd(mean_fil))

UNMC_RPMI_reps <- merge(UNMC_RPMI_reps,unmc_RPMIplateID) #this adds replicate and strain names to the f

UNMC_RPMI_reps$weighted<-UNMC_RPMI_reps$mean_mean/mean(CLIN_RPMI_avg[108:112,3])*4 #This adds a weighti

p <- ggplot(UNMC_RPMI_reps, aes(x = Strain, y = weighted)) + geom_boxplot() + theme(axis.text.x = elemen
p #this plots out the data as a boxplot (can do a violin plot instead)
```

YPD data

Hirakawa YPD data

```
CLIN_YPD <- read_excel("/Users/jrblankenship/Desktop/Research/UNO/Graduate_projects/Nichole_Brandquist/
df_CLIN_YPD <- data.table(CLIN_YPD) #makes it a dataframe
mat_CLIN_YPD <- as.matrix(df_CLIN_YPD) #converts it to a matrix for XGboost
bst <- xgboost::xgb.load('/Users/jrblankenship/Desktop/Research/UNO/Graduate_projects/Nichole_Brandquis
pred_CLIN_YPD <- predict(bst, mat_CLIN_YPD[,3:50]) #makes the predictions and the outcome is a matrix

#Now I need to pull out the plate number as a vector, put it together with the labels for the plates an
plate_CLIN_YPD <-as.vector(mat_CLIN_YPD[,"plate_CLIN_YPD"]) # Pulling out that image number as a vector
CLIN_YPD_data <- cbind(plate_CLIN_YPD, pred_CLIN_YPD) # putting the two together as a table.
```

```
clinYPDplateID <- read_excel("/Users/jrblankenship/Desktop/Research/UNO/Graduate_projects/Nichole_Brand
CLIN_YPD_data <- merge(CLIN_YPD_data,clinYPDplateID) #this merges the files and also includes the cells
colnames(CLIN_YPD_data)[2] = "pred" #changes that prediction column to a more universal name

CLIN_YPD_avg <- CLIN_YPD_data %>%
    group_by(FileName_Original) %>%
    summarise(median_fil = median(pred), mean_fil = mean(pred), stdev_fil = sd(pred)) #this gives the m

CLIN_YPD_avg <- merge(CLIN_YPD_avg,clinYPDplateID) #this adds replicate and strain names to the file fo

CLIN_YPD_avg$weighted <- (1-CLIN_YPD_avg$mean_fil)/(1-mean(CLIN_YPD_avg[101:110,3]))*4 #This adds a wei

p <- ggplot(CLIN_YPD_avg, aes(x = Strain, y = weighted)) + geom_boxplot() + theme(axis.text.x = element
p #this plots out the data as a boxplot (can do a violin plot instead)

CLIN_YPD_strains <- CLIN_YPD_avg %>%
    group_by(Strain) %>%
    summarise(mean_score = mean(weighted), stdev_score = sd(weighted))
```

UNMC YPD data

```
UNMC_YPD <- read_excel("/Users/jrblankenship/Desktop/Research/UNO/Graduate_projects/Nichole_Brandquist/
df_UNMC_YPD <- data.table(UNMC_YPD) #makes it a dataframe
mat_UNMC_YPD <- as.matrix(df_UNMC_YPD) #converts it to a matrix for XGboost
bst <- xgboost::xgb.load('/Users/jrblankenship/Desktop/Research/UNO/Graduate_projects/Nichole_Brandquis
pred_UNMC_YPD <- predict(bst, mat_UNMC_YPD[,3:50]) #makes the predictions and the outcome is a matrix

#Now I need to pull out the plate number as a vector, put it together with the labels for the plates an
plate_UNMC_YPD <-as.vector(mat_UNMC_YPD[,"plate_UNMC_YPD"]) # Pulling out that image number as a vector
UNMC_YPD_data <- cbind(plate_UNMC_YPD, pred_UNMC_YPD) # putting the two together as a table.
unmc_YPDplateID <- read_excel("/Users/jrblankenship/Desktop/Research/UNO/Graduate_projects/Nichole_Bran
UNMC_YPD_data <- merge(UNMC_YPD_data,unmc_YPDplateID) #this merges the files and also includes the cell
colnames(UNMC_YPD_data)[2] = "pred" #changes that prediction column to a more universal name

UNMC_YPD_avg <- UNMC_YPD_data %>%
    group_by(FileName_Original) %>%
    summarise(median_fil = median(pred), mean_fil = mean(pred), stdev_fil = sd(pred)) #this gives the m

UNMC_YPD_avg <- merge(UNMC_YPD_avg, unmc_YPDplateID)

UNMC_YPD_reps <- UNMC_YPD_avg %>%
    group_by(Replicate) %>%
    summarise(mean_mean = mean(mean_fil), stdev_mean = sd(mean_fil))

#below is removing variation between replicates.
UNMC_YPD_reps <- merge(UNMC_YPD_reps,unmc_YPDplateID) #this adds replicate and strain names to the file

UNMC_YPD_reps$weighted<-(1-UNMC_YPD_reps$mean_mean)/(1-mean(CLIN_YPD_avg[101:110,3]))*4 #This adds a we

p <- ggplot(UNMC_YPD_reps, aes(x = Strain, y = weighted)) + geom_boxplot() + theme(axis.text.x = elemen
p #this plots out the data as a boxplot (can do a violin plot instead)
```

Spider

Hirakawa Spider data

```
CLIN_Spider <- read_excel("/Users/jrblankenship/Desktop/Research/UNO/Graduate_projects/Nichole_Brandquis
df_CLIN_Spider <- data.table(CLIN_Spider) #makes it a dataframe
mat_CLIN_Spider <- as.matrix(df_CLIN_Spider) #converts it to a matrix for XGboost
bst <- xgboost::xgb.load('/Users/jrblankenship/Desktop/Research/UNO/Graduate_projects/Nichole_Brandquis
pred_CLIN_Spider <- predict(bst, mat_CLIN_Spider[,3:50]) #makes the predictions and the outcome is a ma

#Now I need to pull out the plate number as a vector, put it together with the labels for the plates an
plate_CLIN_spider <-as.vector(mat_CLIN_Spider[,"plate_CLIN_spider"]) # Pulling out that image number as
CLIN_Spider_data <- cbind(plate_CLIN_spider, pred_CLIN_Spider) # putting the two together as a table.
clinSpiderplateID <- read_excel("/Users/jrblankenship/Desktop/Research/UNO/Graduate_projects/Nichole_Br
CLIN_Spider_data <- merge(CLIN_Spider_data,clinSpiderplateID) #this merges the files and also includes
colnames(CLIN_Spider_data)[2] = "pred" #changes that prediction column to a more universal name

# NOT WORKING BELOW
CLIN_Spider_avg <- CLIN_Spider_data %>%
    group_by(FileName_Original) %>%
    summarise(median_fil = median(pred), mean_fil = mean(pred), stdev_fil = sd(pred)) #this gives the m

CLIN_Spider_avg <- merge(CLIN_Spider_avg,clinSpiderplateID) #this adds replicate and strain names to th

CLIN_Spider_avg$weighted <- (CLIN_Spider_avg$mean_fil)/(mean(CLIN_Spider_avg[122:132,3]))*4 #This adds 

p <- ggplot(CLIN_Spider_avg, aes(x = Strain, y = weighted)) + geom_boxplot() + theme(axis.text.x = eleme
p #this plots out the data as a boxplot (can do a violin plot instead)

CLIN_Spider_strains <- CLIN_Spider_avg %>%
    group_by(Strain) %>%
    summarise(mean_score = mean(weighted), stdev_score = sd(weighted))
```

Spider UNMC strains

```
UNMC_Spider <- read_excel("/Users/jrblankenship/Desktop/Research/UNO/Graduate_projects/Nichole_Brandquis
df_UNMC_Spider <- data.table(UNMC_Spider) #makes it a dataframe
mat_UNMC_Spider <- as.matrix(df_UNMC_Spider) #converts it to a matrix for XGboost
bst <- xgboost::xgb.load('/Users/jrblankenship/Desktop/Research/UNO/Graduate_projects/Nichole_Brandquis
pred_UNMC_Spider <- predict(bst, mat_UNMC_Spider[,3:50]) #makes the predictions and the outcome is a ma

#Now I need to pull out the plate number as a vector, put it together with the labels for the plates an
plate_UNMC_Spider <-as.vector(mat_UNMC_Spider[,"plate_UNMC_Spider"]) # Pulling out that image number as
UNMC_Spider_data <- cbind(plate_UNMC_Spider, pred_UNMC_Spider) # putting the two together as a table.
unmc_SpiderplateID <- read_excel("/Users/jrblankenship/Desktop/Research/UNO/Graduate_projects/Nichole_B
UNMC_Spider_data <- merge(UNMC_Spider_data,unmc_SpiderplateID) #this merges the files and also includes
colnames(UNMC_Spider_data)[2] = "pred" #changes that prediction column to a more universal name

UNMC_Spider_avg <- UNMC_Spider_data %>%
    group_by(FileName_Original) %>%
```

```
    summarise(median_fil = median(pred), mean_fil = mean(pred), stdev_fil = sd(pred)) #this gives the m

UNMC_Spider_avg <- merge(UNMC_Spider_avg, unmc_SpiderplateID)

UNMC_Spider_reps <- UNMC_Spider_avg %>%
    group_by(Replicate) %>%
    summarise(mean_mean = mean(mean_fil), stdev_mean = sd(mean_fil))

UNMC_Spider_reps <- merge(UNMC_Spider_reps,unmc_SpiderplateID) #this adds replicate and strain names to

UNMC_Spider_reps$weighted<-UNMC_Spider_reps$mean_mean/mean(CLIN_Spider_avg[122:132,3])*4 #This adds a w

p <- ggplot(UNMC_Spider_reps, aes(x = Strain, y = weighted)) + geom_boxplot() + theme(axis.text.x = eler
p #this plots out the data as a boxplot (can do a violin plot instead)
```

Lees

Hirakawa Lees data

```
CLIN_Lees <- read_excel("/Users/jrblankenship/Desktop/Research/UNO/Graduate_projects/Nichole_Brandquist/
df_CLIN_Lees <- data.table(CLIN_Lees) #makes it a dataframe
mat_CLIN_Lees <- as.matrix(df_CLIN_Lees) #converts it to a matrix for XGboost
bst <- xgboost::xgb.load('/Users/jrblankenship/Desktop/Research/UNO/Graduate_projects/Nichole_Brandquist
pred_CLIN_Lees <- predict(bst, mat_CLIN_Lees[,2:49]) #makes the predictions and the outcome is a matrix

#Now I need to pull out the plate number as a vector, put it together with the labels for the plates an
plate_CLIN_Lees <-as.vector(mat_CLIN_Lees[,"plate_CLIN_Lees"]) # Pulling out that image number as a vec
CLIN_Lees_data <- cbind(plate_CLIN_Lees, pred_CLIN_Lees) # putting the two together as a table.
clinLeesplateID <- read_excel("/Users/jrblankenship/Desktop/Research/UNO/Graduate_projects/Nichole_Bran
CLIN_Lees_data <- merge(CLIN_Lees_data,clinLeesplateID) #this merges the files and also includes the ce
colnames(CLIN_Lees_data)[2] = "pred" #changes that prediction column to a more universal name

# NOT WORKING BELOW
CLIN_Lees_avg <- CLIN_Lees_data %>%
    group_by(FileName_Original) %>%
    summarise(median_fil = median(pred), mean_fil = mean(pred), stdev_fil = sd(pred)) #this gives the m

CLIN_Lees_avg <- merge(CLIN_Lees_avg,clinLeesplateID) #this adds replicate and strain names to the file

CLIN_Lees_avg$weighted <- (CLIN_Lees_avg$mean_fil)/(mean(CLIN_Lees_avg[121:130,3]))*4 #This adds a weig

p <- ggplot(CLIN_Lees_avg, aes(x = Strain, y = weighted)) + geom_boxplot() + theme(axis.text.x = elemen
p #this plots out the data as a boxplot (can do a violin plot instead)

CLIN_Lees_strains <- CLIN_Lees_avg %>%
    group_by(Strain) %>%
    summarise(mean_score = mean(weighted), stdev_score = sd(weighted))
```

Lees UNMC strains

```
UNMC_Lees <- read_excel("/Users/jrblankenship/Desktop/Research/UNO/Graduate_projects/Nichole_Brandquist/
df_UNMC_Lees <- data.table(UNMC_Lees) #makes it a dataframe
mat_UNMC_Lees <- as.matrix(df_UNMC_Lees) #converts it to a matrix for XGboost
bst <- xgboost::xgb.load('/Users/jrblankenship/Desktop/Research/UNO/Graduate_projects/Nichole_Brandquist
pred_UNMC_Lees <- predict(bst, mat_UNMC_Lees[,3:50]) #makes the predictions and the outcome is a matrix

#Now I need to pull out the plate number as a vector, put it together with the labels for the plates an
plate_UNMC_Lees <-as.vector(mat_UNMC_Lees[,"plate_UNMC_Lees"]) # Pulling out that image number as a vec
UNMC_Lees_data <- cbind(plate_UNMC_Lees, pred_UNMC_Lees) # putting the two together as a table.
UNMC_LeesplateID <- read_excel("/Users/jrblankenship/Desktop/Research/UNO/Graduate_projects/Nichole_Bran
UNMC_Lees_data <- merge(UNMC_Lees_data,UNMC_LeesplateID) #this merges the files and also includes the c
colnames(UNMC_Lees_data)[2] = "pred" #changes that prediction column to a more universal name

UNMC_Lees_avg <- UNMC_Lees_data %>%
    group_by(FileName_Original) %>%
    summarise(median_fil = median(pred), mean_fil = mean(pred), stdev_fil = sd(pred)) #this gives the m

UNMC_Lees_avg <- merge(UNMC_Lees_avg, UNMC_LeesplateID)

UNMC_Lees_reps <- UNMC_Lees_avg %>%
    group_by(Replicate) %>%
    summarise(mean_mean = mean(mean_fil), stdev_mean = sd(mean_fil))

UNMC_Lees_reps <- merge(UNMC_Lees_reps,UNMC_LeesplateID) #this adds replicate and strain names to the f

UNMC_Lees_reps$weighted<-UNMC_Lees_reps$mean_mean/mean(CLIN_Lees_avg[121:130,3])*4 #This adds a weighti

p <- ggplot(UNMC_Lees_reps, aes(x = Strain, y = weighted)) + geom_boxplot() + theme(axis.text.x = elemen
p #this plots out the data as a boxplot (can do a violin plot instead)
```

Exporting dataframes to Excel for image generation

```
write_xlsx(clin_FBS_avg,"/Users/jrblankenship/Desktop/Research/UNO/Graduate_projects/Nichole_Brandquist/
write_xlsx(UNMC_FBS_reps,"/Users/jrblankenship/Desktop/Research/UNO/Graduate_projects/Nichole_Brandquist
write_xlsx(CLIN_RPMI_avg,"/Users/jrblankenship/Desktop/Research/UNO/Graduate_projects/Nichole_Brandquist
write_xlsx(UNMC_RPMI_reps,"/Users/jrblankenship/Desktop/Research/UNO/Graduate_projects/Nichole_Brandqui
write_xlsx(CLIN_YPD_avg,"/Users/jrblankenship/Desktop/Research/UNO/Graduate_projects/Nichole_Brandquist/
write_xlsx(UNMC_YPD_reps,"/Users/jrblankenship/Desktop/Research/UNO/Graduate_projects/Nichole_Brandquist
write_xlsx(CLIN_Spider_avg,"/Users/jrblankenship/Desktop/Research/UNO/Graduate_projects/Nichole_Brandqu
write_xlsx(UNMC_Spider_reps,"/Users/jrblankenship/Desktop/Research/UNO/Graduate_projects/Nichole_Brandq
write_xlsx(CLIN_Lees_avg,"/Users/jrblankenship/Desktop/Research/UNO/Graduate_projects/Nichole_Brandquist
write_xlsx(UNMC_Lees_reps,"/Users/jrblankenship/Desktop/Research/UNO/Graduate_projects/Nichole_Brandqui
```

Lost cells

Hirakawa lost data

```
Lost_cells <- read_excel("/Users/jrblankenship/Desktop/Research/UNO/Graduate_projects/Nichole_Brandquis
df_Lost_cells <- data.table(Lost_cells) #makes it a dataframe
mat_Lost_cells <- as.matrix(df_Lost_cells) #converts it to a matrix for XGboost
bst <- xgboost::xgb.load('/Users/jrblankenship/Desktop/Research/UNO/Graduate_projects/Nichole_Brandquis
pred_Lost_cells <- predict(bst, mat_Lost_cells[,2:49]) #makes the predictions and the outcome is a matr

#Now I need to pull out the plate number as a vector, put it together with the labels for the plates an
plate_LOSTcells <-as.vector(mat_Lost_cells[,"plate_LOSTcells"]) # Pulling out that image number as a ve
Lost_cells_data <- cbind(plate_LOSTcells, pred_Lost_cells) # putting the two together as a table.
Lost_cellsID <- read_excel("/Users/jrblankenship/Desktop/Research/UNO/Graduate_projects/Nichole_Brandqu
Lost_cells_data <- merge(Lost_cells_data,Lost_cellsID) #this merges the files and also includes the cel
colnames(Lost_cells_data)[2] = "pred" #changes that prediction column to a more universal name

Lost_cells_avg <- Lost_cells_data %>%
    group_by(FileName_Original) %>%
    summarise(median_fil = median(pred), mean_fil = mean(pred), stdev_fil = sd(pred)) #this gives the m

Lost_cells_avg <- merge(Lost_cells_avg,Lost_cellsID) #this adds replicate and strain names to the file

Lost_cells_avg$weightedFBS <- (Lost_cells_avg$mean_fil)/(mean(clin_FBS_avg[101:105,3]))*4 #This adds a

Lost_cells_avg$weightedYPD <- (1-Lost_cells_avg$mean_fil)/(1-mean(CLIN_YPD_avg[101:110,3]))*4

Lost_cells_avg$weightedRPMI <- (Lost_cells_avg$mean_fil)/(mean(CLIN_RPMI_avg[108:112,3]))*4

write_xlsx(Lost_cells_avg,"/Users/jrblankenship/Desktop/Research/UNO/Graduate_projects/Nichole_Brandqui
```
