# Supplementary material for: Significant variation of filamentation phenotypes in clinical Candida albicans strains
Source: Front Cell Infect Microbiol. 2023 Oct 20;13:1207083. doi: 10.3389/fcimb.2023.1207083 (PMC10623444; doi:10.3389/fcimb.2023.1207083)
Supplement: Supplementary file 1 [file DataSheet_1.zip › coding/filclusters.pdf]

Nichole paper

jrblankenship

2023-03-26

Looking at clade vs. filamentation profiles

The question is, does clade influence phenotype?

```
library(readxl)
TempstandardMEV <- read_excel("Desktop/Research/UNO/Graduate_projects/Nichole_Brandquist/Paper/resubmis
Filscores <- as.data.frame(TempstandardMEV) #converting to a data frame
rownames(Filscores) <- Filscores[ ,1] #adding the rownames
Filscores <- Filscores[ ,-1] # removing the strain names from the table
library(pvclust)
cluster_filscore <- pvclust(Filscores, nboot = 10000) # clustering analysis with the condensed dataset
```

```
## Bootstrap (r = 0.5)... Done.
## Bootstrap (r = 0.6)... Done.
## Bootstrap (r = 0.7)... Done.
## Bootstrap (r = 0.8)... Done.
## Bootstrap (r = 0.9)... Done.
## Bootstrap (r = 1.0)... Done.
## Bootstrap (r = 1.1)... Done.
## Bootstrap (r = 1.2)... Done.
## Bootstrap (r = 1.3)... Done.
## Bootstrap (r = 1.4)... Done.
```

```
plot(cluster_filscore) #quick look at the data
```

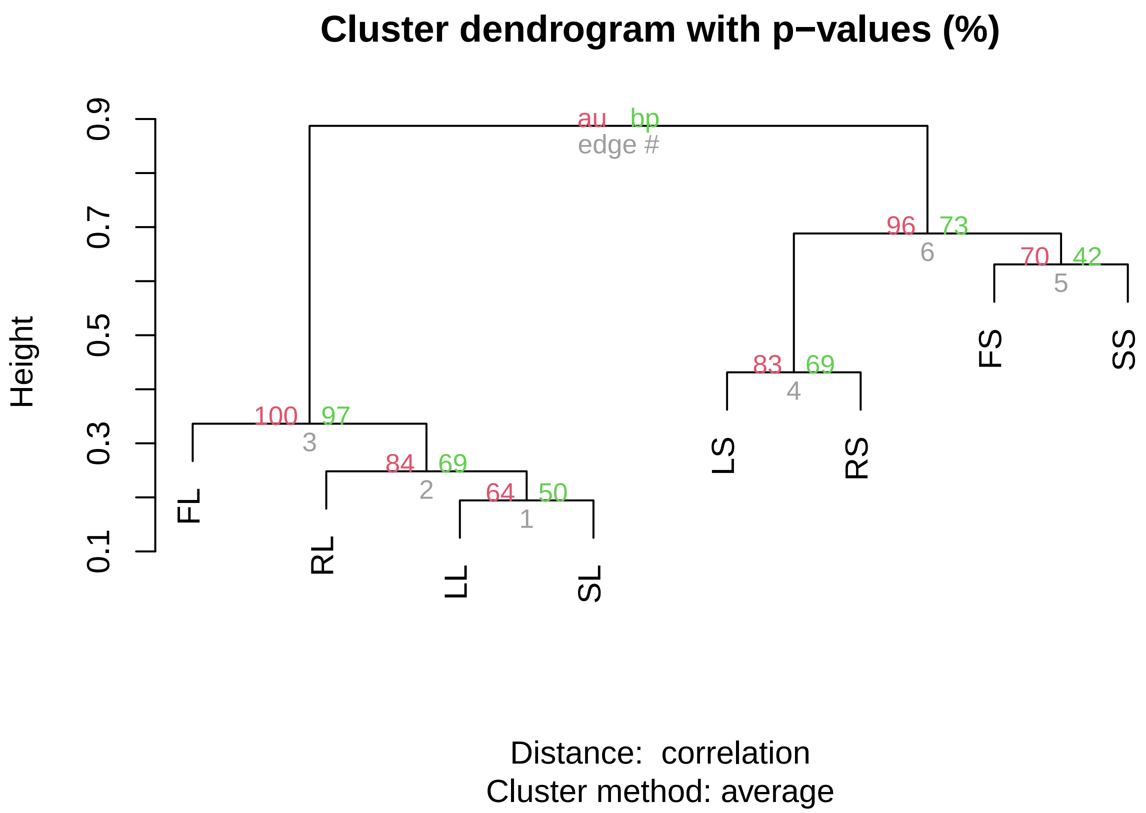


```r
#This follows https://rpubs.com/emptyhb/645504 example to cluster genes in expression data.
filscaled<-scale(t(Filscores)) #scaling subtracts the mean score from each condition, standardizing the
t(filscaled) #not necessary but shows the scaled data.
```

```
##                    FL          LL          RL          SL          FS
## P37039    -0.82081344  1.37853302  0.08572721 1.630787812 -1.15978082
## B527-15   -0.53561088  1.63483845  0.17853696 0.976702198 -1.40379061
## B1559-15  -0.36693738  0.99003858  0.20077705 1.405439385 -0.54694439
## P60002    -0.19612915  1.09355142 -0.39436583 1.526874694 -1.42111749
## 12C        0.07428393  0.60396067  0.09366235 1.243448432  0.13241918
## B733-15    0.41470667  1.18080747  0.83891125 0.756602897 -1.82661304
## P76055     0.48255958  1.05213809  0.85700471 0.814813710 -1.15234172
## B1168-15   0.25249544  1.29556116  0.66033775 1.309998057 -0.90967467
## B421-15    0.40253920  1.49806320  0.55045805 0.971102290 -0.99036179
## B618-15    0.36166535  1.11197642  0.30513506 1.579636052 -0.95908769
## B444-12    0.34431207  1.60858211  0.43770333 1.059280952 -0.88254681
## L26        0.32510123  1.27781895  0.70837848 1.255917394 -0.90138594
## P34048     0.25421320  0.89337783  0.71906020 1.601213032 -0.95544547
## B404-15   -0.81920020 -0.02457601 -0.81920020 0.008192002  0.37956003
## B2527-12   0.29565328  1.10682787  0.46708708 1.399519738 -1.21798179
## P37005     0.75199258  1.47871820  0.49691669 0.872311388 -0.90359428
## P87        0.41406151  1.13128143  0.52961361 1.461999496 -0.90084166
## P78048     0.19892091  0.94695883  0.32756438 1.790288203 -0.81593308
## B808-15    0.58639244  0.86141737  0.61782386 1.057863745 -1.06375714
## P57072     0.16418021  0.95572454  0.46862034 1.312354399 -1.14926147
## P76067     0.15919240  1.10909608  0.13397372 1.365486011  0.51645706
## B568-15    0.02491133  0.98733936  0.12212628 1.692147773  0.17559451
```

```
## B687-15   0.52021775  1.14167739  0.98885945 1.029610902 -0.93664665
## B1762-15  0.60121699  1.26695778  0.52885387 1.218715692 -0.90393608
## B1486-15  0.25220399  1.04387570  0.51006278 1.622927014 -0.91042247
## P57055    0.66148498  0.53350723 -0.11917928 0.207163978  0.87904715
## P75010    0.47290552  1.45578028 -0.05971376 1.400871077 -0.87236998
## B564-14   0.20227895  1.07431336  0.60243327 1.512218082 -0.39165953
## B510-12   0.09310693  0.51381231 -0.17586864 0.603470828 -1.81041248
## 19F       0.46851331  0.94152538  0.09318852 1.044354086 -1.15103887
## P78042    0.03843141  1.20427725  0.75417221 1.366610220 -0.99459656
## P75016    0.24878921  0.81105283  0.45279636 1.169309289 -1.18423664
## GC75      0.57753950  0.87865989  0.45520934 1.593820813 -0.92335745
## B46-15    0.84890650  1.19353720  0.11910032 1.335443961 -0.87424700
## B212-12   0.75872803  1.13152039  0.63446391 1.160196723 -0.91883757
## B1257-15 -0.08635639  1.04469585  0.51769666 1.329493182 -1.32861386
## B1091-15  0.43921491  0.98823356  0.38650912 1.633879478 -0.78619470
## P75063    0.12971443  1.21061316  0.74910584 1.388738793 -0.88641135
## P37037    0.52239098  0.88257555  0.52732501 1.223023982 -1.17985117
## P94015    0.29608073  0.74242085  0.28086459 1.721325885  0.09319886
##                   LS         RS         SS
## P37039   -0.37148460 -0.3714846 -0.3714846
## B527-15  -0.56361668 -0.7456544  0.4585949
## B1559-15  0.05538677 -1.8831503  0.1453903
## P60002   -1.07306493  0.2321256  0.2321256
## 12C      -1.67623308 -1.2692863  0.7977448
## B733-15  -0.45480508 -0.1382345 -0.7713757
## P76055   -1.18925885 -1.1892588  0.3243433
## B1168-15 -0.90967467 -0.9096747 -0.7893684
## B421-15  -1.06740440 -1.0674044 -0.2969922
## B618-15  -0.99506151 -0.9745050 -0.4297586
## B444-12  -0.88254681 -0.8022380 -0.8825468
## L26      -0.86305822 -0.9013859 -0.9013859
## P34048   -0.91846900 -0.9184690 -0.6754808
## B404-15  -1.39536827  1.3352963  1.3352963
## B2527-12 -0.10296377 -0.7301606 -1.2179818
## P37005   -0.90359428 -0.8891560 -0.9035943
## P87      -0.90084166 -0.8344311 -0.9008417
## P78048   -0.89534421 -0.5777044 -0.9747506
## B808-15   0.42923534 -1.2444878 -1.2444878
## P57072   -1.14926147 -1.1057700  0.5034135
## P76067   -1.09473509 -1.0947351 -1.0947351
## B568-15  -1.23402231 -1.0055672 -0.7625298
## B687-15  -0.92136486 -0.8857073 -0.9366467
## B1762-15 -0.90393608 -0.9039361 -0.9039361
## B1486-15 -0.77470732 -0.8335172 -0.9104225
## P57055    0.87904715 -1.6805078 -1.3605634
## P75010   -0.70764237 -0.8174608 -0.8723700
## B564-14  -1.02083424 -0.9579156 -1.0208342
## B510-12   0.94831130  0.9483113 -1.1207315
## 19F      -1.15103887  0.9055353 -1.1510389
## P78042   -0.99459656 -0.3797014 -0.9945966
## P75016   -1.18423664 -1.1195514  0.8060770
## GC75     -0.97040751 -0.9704075 -0.6410571
## B46-15   -0.87424700 -0.8742470 -0.8742470
## B212-12  -0.93317574 -0.9331757 -0.8997200
```

```
## B1257-15 -0.31148054  0.2309892 -1.3964241
## B1091-15 -0.74227320 -0.9750571 -0.9443121
## P75063   -0.88641135 -0.8189382 -0.8864113
## P37037   -1.17985117 -1.1305108  0.3348976
## P94015   -1.02265144 -1.1900290 -0.9212105
## attr(,"scaled:center")
##   P37039  B527-15 B1559-15   P60002      12C  B733-15   P76055 B1168-15
## 4.471250 3.602500 3.720000 3.555380 2.695000 2.885001 2.255000 2.520416
##  B421-15  B618-15  B444-12      L26   P34048  B404-15 B2527-12   P37005
## 2.309168 1.966250 1.831606 1.646250 1.808750 2.370000 2.912917 1.877500
##      P87   P78048  B808-15   P57072   P76067  B568-15  B687-15 B1762-15
## 2.260834 3.212500 1.583750 2.642500 2.771251 2.568750 1.838750 1.873750
## B1486-15   P57055   P75010  B564-14  B510-12      19F   P78042   P75016
## 2.012500 2.626250 1.588750 2.704168 2.625000 2.238750 1.347916 2.380000
##     GC75   B46-15  B212-12 B1257-15 B1091-15   P75063   P37037   P94015
## 2.062500 0.431250 1.952500 3.432254 2.220000 2.189584 2.391250 2.416250
## attr(,"scaled:scale")
##    P37039   B527-15  B1559-15    P60002       12C   B733-15    P76055  B1168-15
## 1.2685587 0.7141378 1.4443882 1.9154291 1.5481141 1.5794266 1.8961389 2.7706787
##   B421-15   B618-15   B444-12       L26    P34048   B404-15  B2527-12    P37005
## 2.1633483 1.9458596 2.0753643 1.8263542 1.8930960 1.2207028 2.3915936 2.0778131
##       P87    P78048   B808-15    P57072    P76067   B568-15   B687-15  B1762-15
## 2.5096905 2.0988241 1.2726119 2.2993027 2.3791886 2.0572967 1.9631202 2.0728789
##  B1486-15    P57055    P75010   B564-14   B510-12       19F    P78042    P75016
## 2.2105122 1.5627717 1.8211883 2.6489780 1.4499458 1.9449821 1.3552392 2.0097335
##      GC75    B46-15   B212-12  B1257-15  B1091-15    P75063    P37037    P94015
## 2.1253958 0.4932816 2.0923176 2.4578882 2.2767897 2.4701666 2.0267387 1.9715906
```

```
filscaled.dist <- dist(t(filscaled), method = "euclidean")
filscaled.clust <- hclust(filscaled.dist)
plot(filscaled.clust)
```

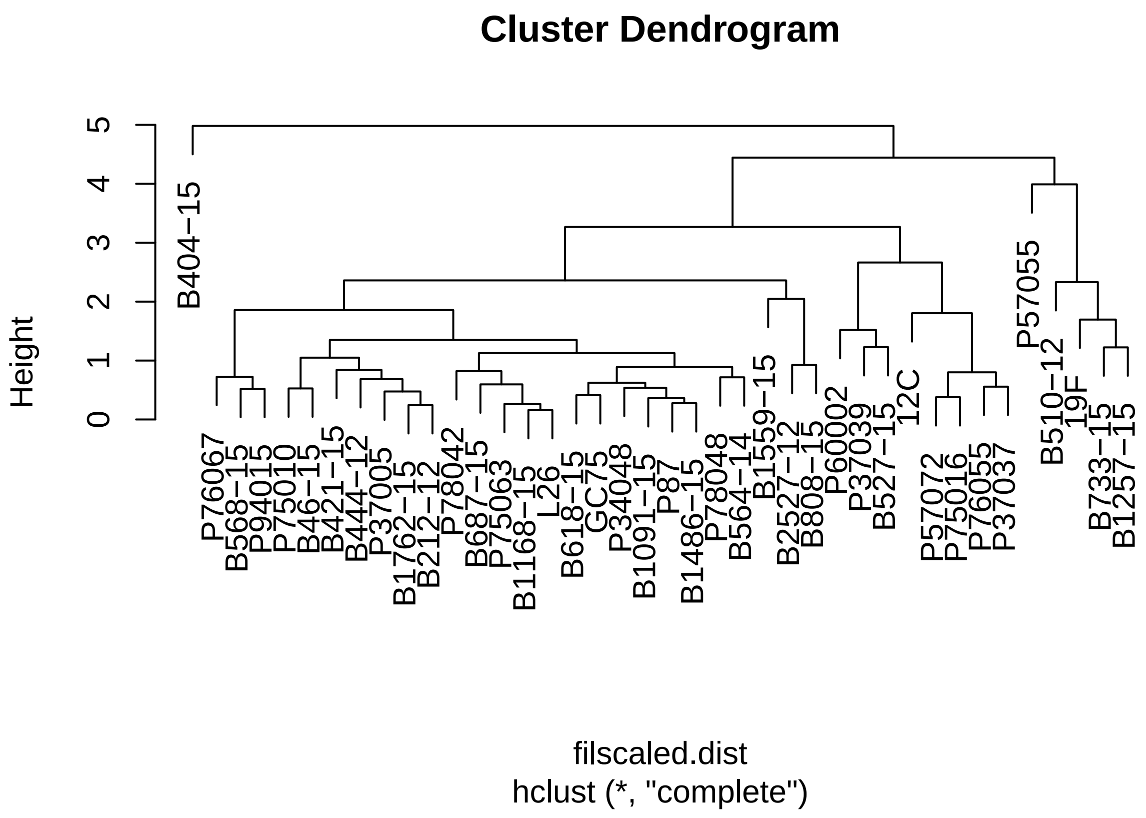

Cluster Dendrogram
Height
0
1
2
3
4
5
B404−15
P76067
B568−15
P94015
P75010
B46−15
B421−15
B444−12
P37005
B1762−15
B212−12
P78042
B687−15
P75063
B1168−15
L26
B618−15
GC75
P34048
B1091−15
P87
B1486−15
P78048
B564−14
B1559−15
B2527−12
B808−15
P60002
P37039
B527−15
12C
P57072
P75016
P76055
P37037
P57055
B510−12
19F
15
B733−15
B1257−15
filscaled.dist
hclust (*, "complete")
