# Supplementary material for: Significant variation of filamentation phenotypes in clinical Candida albicans strains
Source: Front Cell Infect Microbiol. 2023 Oct 20;13:1207083. doi: 10.3389/fcimb.2023.1207083 (PMC10623444; doi:10.3389/fcimb.2023.1207083)
Supplement: Supplementary file 2 [file DataSheet_2.pdf]

SUPPLEMENTAL METHODS AND FIGURES

Bioinformatic measurements of liquid filamentation

The approach used for computational measurements of liquid filamentation were inspired by the approach used in the Metzner, et al paper (Metzner et al., 2023). Technical differences in initial image acquisition required differences in the details of the approach, which are described below.

Masking:

Zeiss-acquired images of liquid filamentation assays were exported as jpeg images. Ten images were hand-selected across induction conditions and strains, with images ranging from almost completely filamentous to almost completely non-filamentous, to serve as a training set for the cell recognition (masking) model in Cellpose (version 2.2.2) (Stringer et al., 2021). The graphic user interface (GUI) version of Cellpose was used to train the masking model with a human-in-the-loop approach (Pachitariu and Stringer, 2022). The initial image was evaluated with the "LC2" model. The resulting cell masks were corrected by hand and these corrections were used to train the initial "Candida_filamentation1" model. For the rest of the training set, the "Candida_filamentation1" model was used for masking, and each prediction was corrected by hand and used to refine the model. Once the model was trained, all liquid filamentation images from each condition (including the non-inducing control) were masked in batches using a python script in a JupyterLab notebook (version 3.5.3) running on Anaconda Navigator (version 2.4.2) modified from the script provided by Cellpose (image masking.md). Resulting files were saved as sgy, png, and tif outputs (see Figure S4 for an example mask).

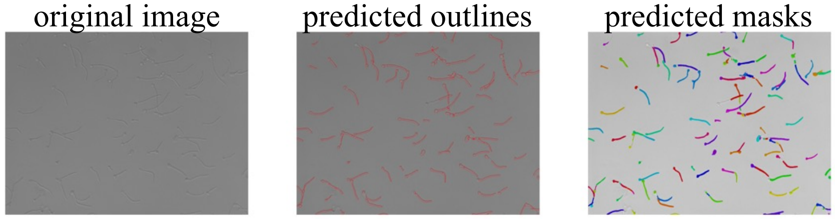

Figure S4. Mask predictions from Cellpose. The jpeg image for one of the FBS-induced GC75 images (original image), predicted cell outline, and predicted masks for that image are shown. The mask and original image were imported to the Cellprofiler program for object measurements.

Measurements:

The jpeg and png masks for each image were uploaded to CellProfiler (version 4.2.5) (Stirling et al., 2021). The "ConvertObjectsToImage" module was used to convert the mask images to black and white images, and these images were measured with the "MeasureObjectSizeShape" module, including Zernike features but not advanced features. These measurements were then exported as a CSV file using the "ExportToSpreadsheet" module. This pipeline was used for each set of images (pipeline.cppipe). This generated two pertinent files: the measurements file, with the measurements for each object in each image; and a file that contained the key that could be used to relate the measurements back to the original experiments.

Statistical comparisons:

Both the measurements file and the key file from the CellProfiler output were converted into Excel files. Measurements pertaining to the position or orientation of the object measured were removed from the measurements file and only the condition identifier, number of objects measured, and the Image number column were retained from the key file. These files were then imported into R (version 4.2.2) (Team, 2022). Measurements from SC5314 in FBS and YPD were split into testing and training sets to generate an xgboost prediction model that would differentiate between filamentous and yeast-like objects (Chen, 2016). The resulting model was used to score the likelihood that an object was a filament, with an output score for each object from 0 to 1 (yeast-like to filamentous). Scores were averaged by image and then by replicate, if applicable. Each condition was normalized to the SC5314 score for that condition and the score was converted to the 0 to 4 scale used in our previous work (Azadmanesh et al., 2017) and the scores for solid filamentation in this paper. The R code for this work is shown in the markup file Filscoring.html. Statistical analysis of the data for each condition is shown in Figure S5. As an observation, the average computational score was remarkably similar to the averaged filamentation score assigned by 3-6 individuals in the lab (data not shown).

A.

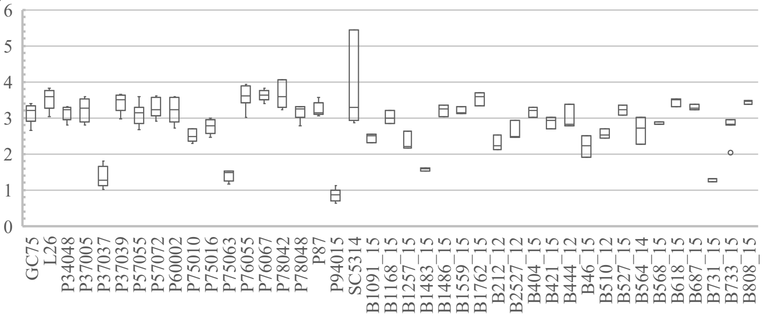

B.

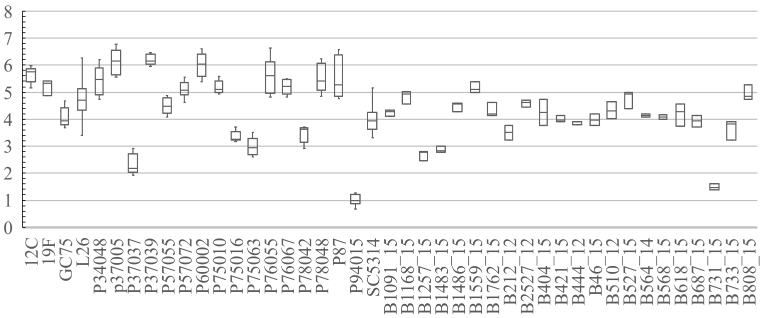

C.

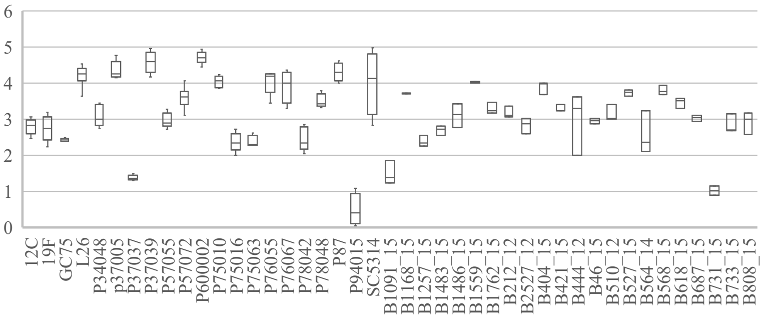

D.

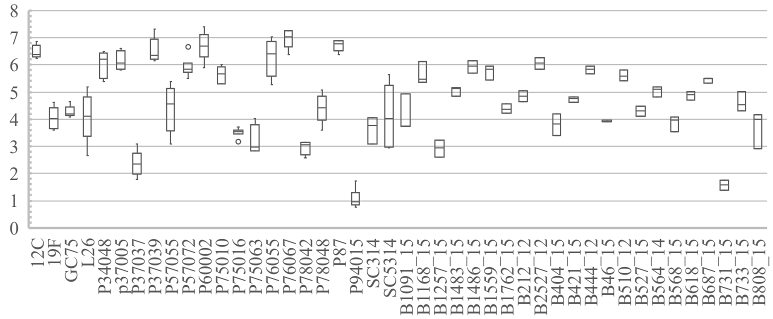

E.

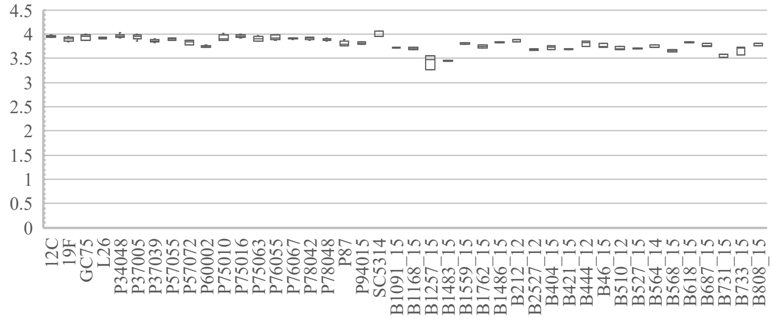

Figure S5. Statistical analysis of liquid filamentation scores. A box and whisker plot was generated for each strain tested for filamentation in liquid A) FBS, B) Lee's, C) RPMI, D) Spider, or E) YPD. The plots represent scores normalized to the SC5314 score for each condition (set at a value of 4). Variation is shown between images (5 images of each strain) for the Hirakawa set or between replicates (5 images of three replicates per strain) for the set of strains from the Nebraska Medical Center. With the exception of the YPD scoring, scores of 2.5 and below were considered defective for filamentation. In the YPD plot, scores of 2.5 and below would have been considered hyperfilamentous, although that was not observed in these sets of strains.

Statistical analysis of human-scored solid filamentation assays

Filamentation in solid media was scored by 3-5 independent scorers. Average and standard deviation scores were calculated for each strain in each condition for both the standard (Figure S6) and shortened solid data (Figure S7).

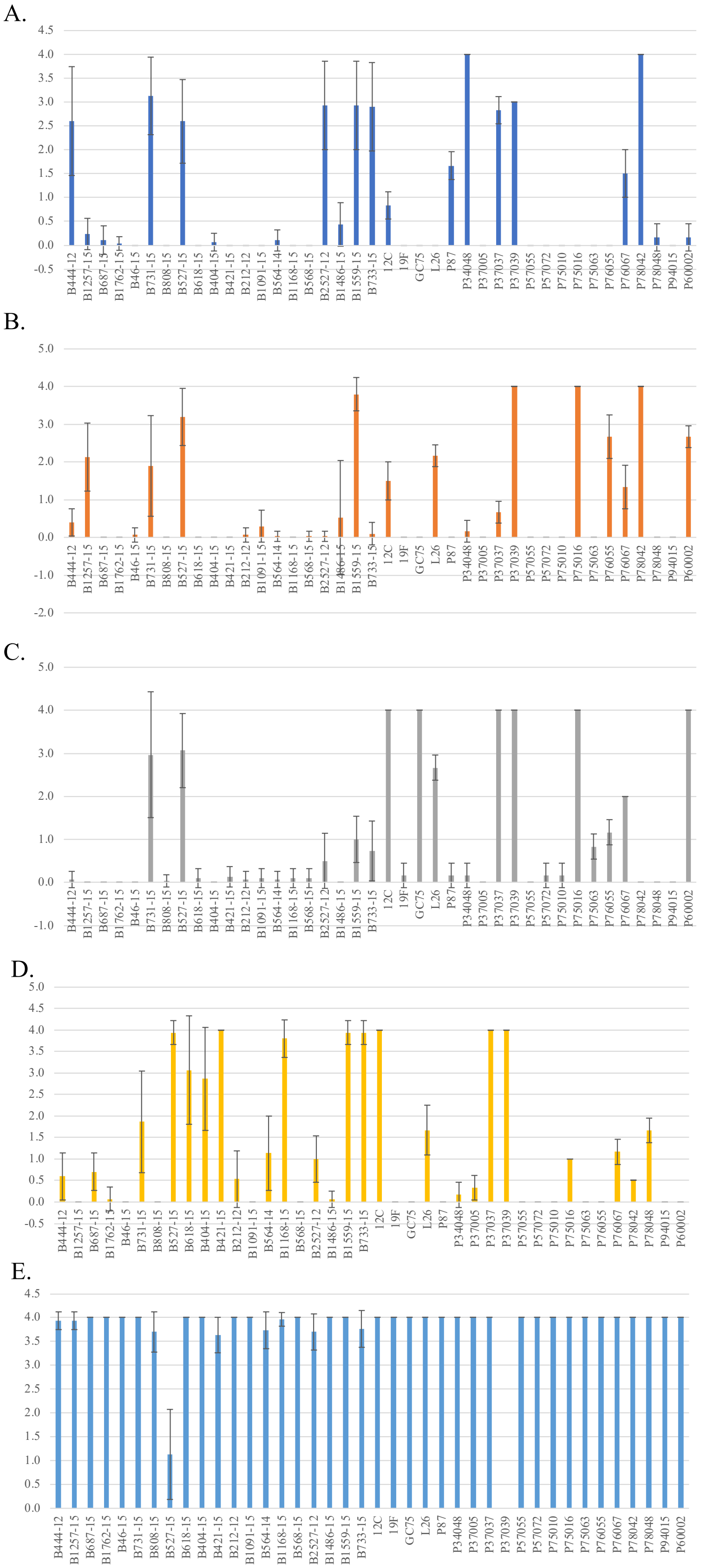
A.
4.5 4.0 3.5 3.0 2.5 2.0 1.5 1.0 0.5 0.0 -0.5
B444-12 B1257-15 B687-15 B1762-15 B46-15 B731-15 B808-15 B527-15 B618-15 B404-15 B421-15 B212-12 B1091-15 B564-14 B1168-15 B568-15 B2527-12 B1486-15 B1559-15 B733-15 12C 19F GC75 L26 P87 P34048 P37005 P37037 P37039 P57055 P57072 P75010 P75016 P75063 P76055 P76067 P78042 P78048 P94015 P60002
B.
5.0 4.0 3.0 2.0 1.0 0.0 -1.0 -2.0
B444-12 B1257-15 B687-15 B1762-15 B46-15 B731-15 B808-15 B527-15 B618-15 B404-15 B421-15 B212-12 B1091-15 B564-14 B1168-15 B568-15 B2527-12 B1486-15 B1559-15 B733-15 12C 19F GC75 L26 P87 P34048 P37005 P37037 P37039 P57055 P57072 P75010 P75016 P75063 P76055 P76067 P78042 P78048 P94015 P60002
C.
5.0 4.0 3.0 2.0 1.0 0.0 -1.0
B444-12 B1257-15 B687-15 B1762-15 B46-15 B731-15 B808-15 B527-15 B618-15 B404-15 B421-15 B212-12 B1091-15 B564-14 B1168-15 B568-15 B2527-12 B1486-15 B1559-15 B733-15 12C 19F GC75 L26 P87 P34048 P37005 P37037 P37039 P57055 P57072 P75010 P75016 P75063 P76055 P76067 P78042 P78048 P94015 P60002
D.
5.0 4.5 4.0 3.5 3.0 2.5 2.0 1.5 1.0 0.5 0.0 -0.5
B444-12 B1257-15 B687-15 B1762-15 B46-15 B731-15 B808-15 B527-15 B618-15 B404-15 B421-15 B212-12 B1091-15 B564-14 B1168-15 B568-15 B2527-12 B1486-15 B1559-15 B733-15 12C 19F GC75 L26 P87 P34048 P37005 P37037 P37039 P57055 P57072 P75010 P75016 P75063 P76055 P76067 P78042 P78048 P94015 P60002
E.
4.5 4.0 3.5 3.0 2.5 2.0 1.5 1.0 0.5 0.0
B444-12 B1257-15 B687-15 B1762-15 B46-15 B731-15 B808-15 B527-15 B618-15 B404-15 B421-15 B212-12 B1091-15 B564-14 B1168-15 B568-15 B2527-12 B1486-15 B1559-15 B733-15 12C 19F GC75 L26 P87 P34048 P37005 P37037 P37039 P57055 P57072 P75010 P75016 P75063 P76055 P76067 P78042 P78048 P94015 P60002

Figure S6. Statistical analysis of scoring for the standard solid filamentation assay. Cells were grown on solid A) FBS, B) Lee's, C) RPMI, D) Spider, and E) YPD agar plates for 4-5 days. Images were taken of the colony edges and these images were scored for their similarity to the SC5314 phenotype. Cells that filamented like the type strain were given a score of 4 while strains that did not filament were given a score of 0 for inducing conditions (FBS, Lee's, RPMI, and Spider). Cells that had a yeast-like phenotype like the type strain were given a score of 4 while strains that did not filament were given a score of 0 for the non-inducing YPD condition. The scores reflect the average scores from 3-5 independent scorers, and error bars indicate the standard deviation of the scores.

A.

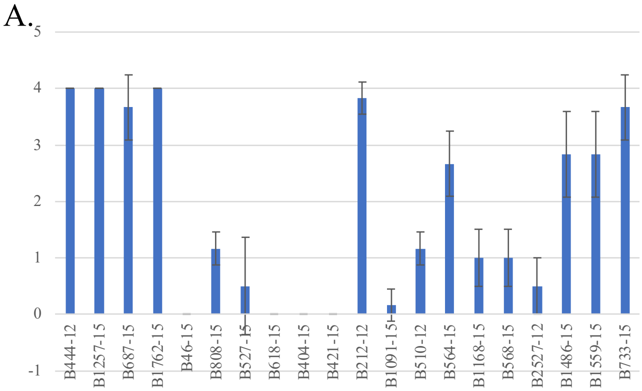

B.

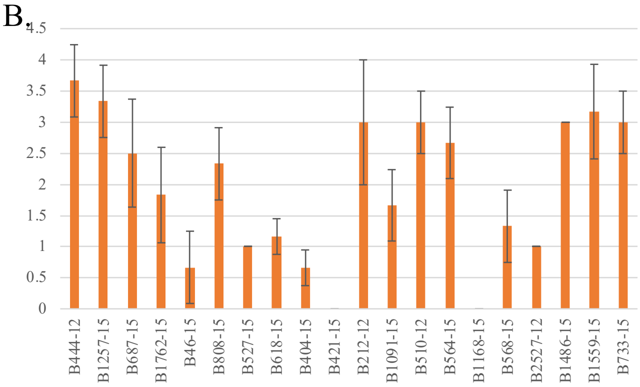

C.

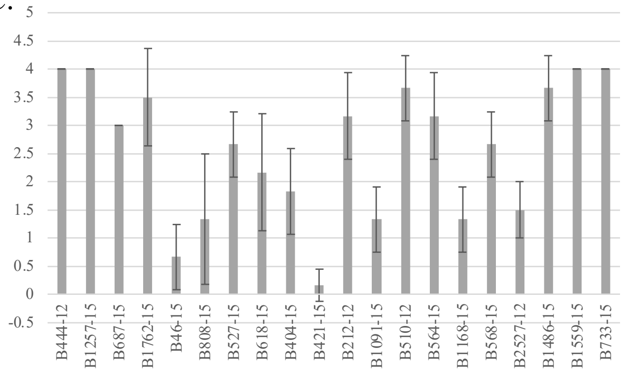

D.

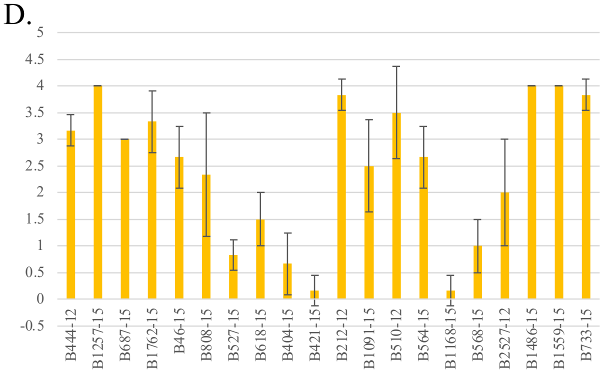

E.

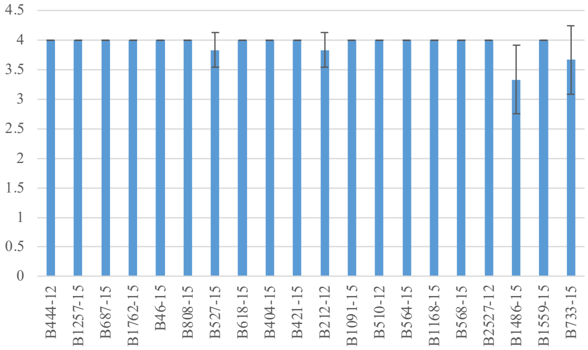

Figure S7. Statistical analysis of scoring for the shortened solid filamentation assay. Cells were grown on solid A) FBS, B) Lee's, C) RPMI, D) Spider, and E) YPD agar plates for 3 hours. Images were taken of the cells and these images were scored for their similarity to the SC5314 phenotype. Cells that filamented like the type strain were given a score of 4 while strains that did not filament were given a score of 0 for inducing conditions (FBS, Lee's, RPMI, and Spider). Cells that had a yeast-like phenotype like the type strain were given a score of 4 while strains that did not filament were given a score of 0 for the non-inducing YPD condition. The scores reflect the average scores from 3 independent scorers, and error bars indicate the standard deviation of the scores.

Hierarchical clustering of filamentation scores by strain

To determine whether there were patterns in filamentation phenotype driven by clade, we did a hierarchical clustering analysis of the data. The filamentation scoring was used to cluster the strains by similarities in phenotype scores across the filamentation assays (Figure S8). The filamentation scoring data was imported to R where it was scaled to normalize the scores by condition, and the data was analyzed by hierarchical clustering using Euclidean distance with complete linkage. The markup file for this analysis is filclusters.rmd.

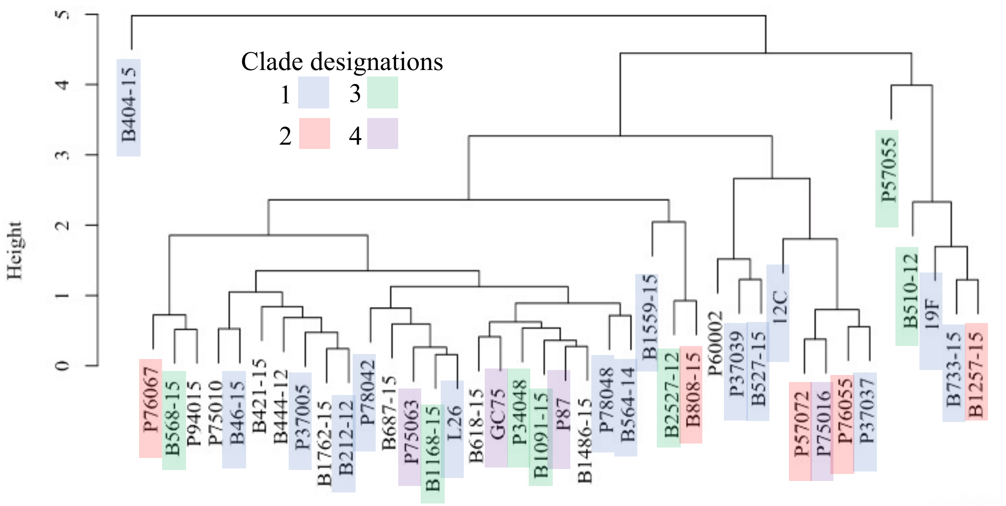

Figure S8. **Hierarchical clustering of strains by phenotype scores**. In order to determine if there are patterns that connect strains by their filamentation phenotype, the filamentation scores were used for hierarchical clustering analysis. The dendrogram shows the outcome of this analysis. Clades 1-4 are denoted by colored boxes, as indicated. Strains from clades with 2 or fewer representatives were left unboxed.

REFERENCES

AZADMANESH, J., GOWEN, A. M., CREGER, P. E., SCHAFER, N. D. & BLANKENSHIP, J. R. 2017. Filamentation Involves Two Overlapping, but Distinct, Programs of Filamentation in the Pathogenic Fungus *Candida albicans*. *G3 (Bethesda),* 7**,** 3797-3808.

CHEN, T. G., C. 2016. XGBoost: A Scalable Tree Boosting System. 22nd ACM SIGKDD International Conference on Knowledge Discovery and Data Mining, 2016 San Francisco, California, USA. New York, NY, USA: ACM, 785--794.

METZNER, K., O'MEARA, M. J., HALLIGAN, B., WOTRING, J. W., SEXTON, J. Z. & O'MEARA, T. R. 2023. Imaging-based screening identifies modulators of the eIF3 translation initiation factor complex in *Candida albicans*. *bioRxiv*.

PACHITARIU, M. & STRINGER, C. 2022. Cellpose 2.0: how to train your own model. *Nat Methods,* 19**,** 1634-1641.

STIRLING, D. R., SWAIN-BOWDEN, M. J., LUCAS, A. M., CARPENTER, A. E., CIMINI, B. A. & GOODMAN, A. 2021. CellProfiler 4: improvements in speed, utility and usability. *BMC Bioinformatics,* 22**,** 433.

STRINGER, C., WANG, T., MICHAELOS, M. & PACHITARIU, M. 2021. Cellpose: a generalist algorithm for cellular segmentation. *Nat Methods,* 18**,** 100-106.

TEAM, R. C. 2022. R: A language and environment for statistical computing. R Foundation for Statistical Computing, Vienna, Austria.
