# Supplementary material for: Significant variation of filamentation phenotypes in clinical Candida albicans strains
Source: Front Cell Infect Microbiol. 2023 Oct 20;13:1207083. doi: 10.3389/fcimb.2023.1207083 (PMC10623444; doi:10.3389/fcimb.2023.1207083)

Figure S1. Liquid filamentation assays FBS

B444-12

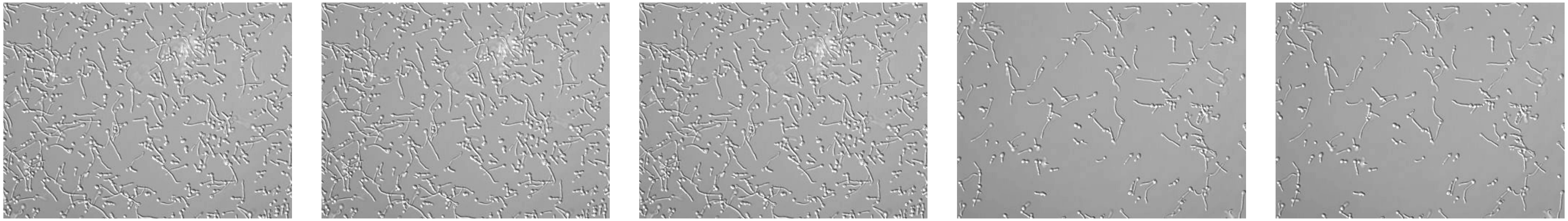

B1257-15

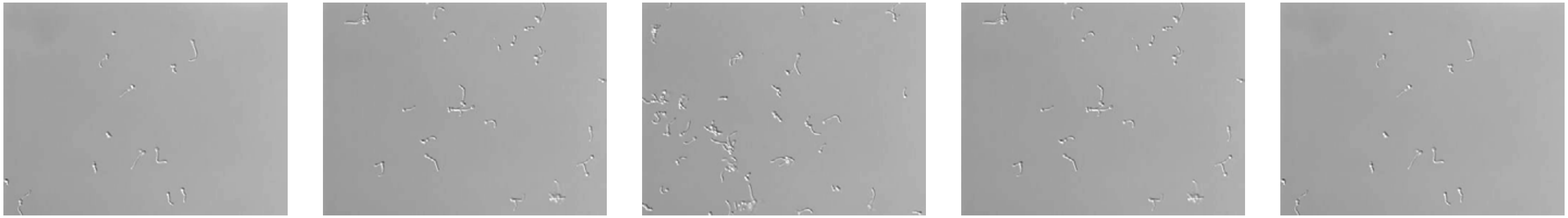

B687-15

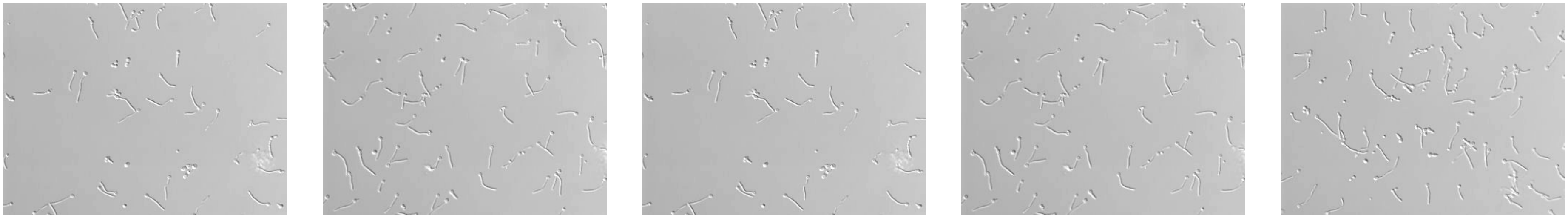

B1762-15

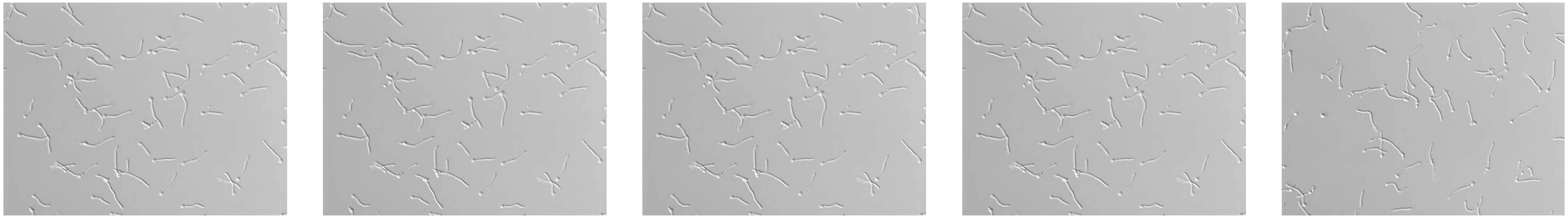

FBS

B46-15

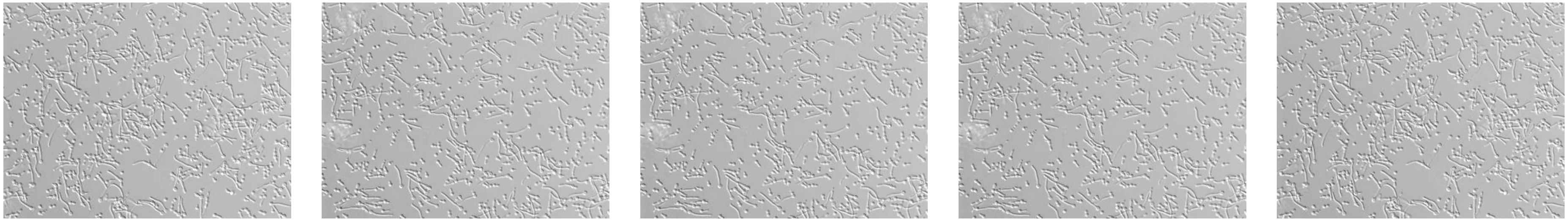

B808-15

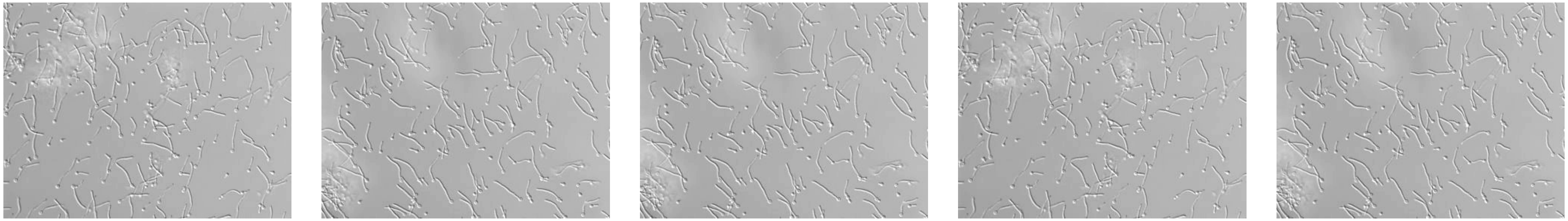

B527-15

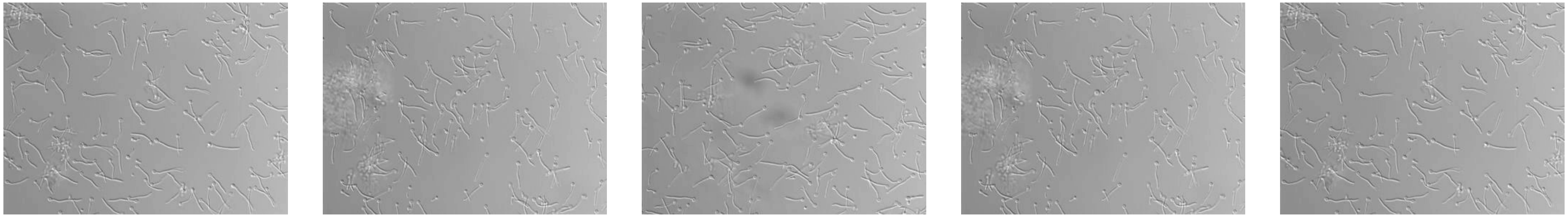

B618-15

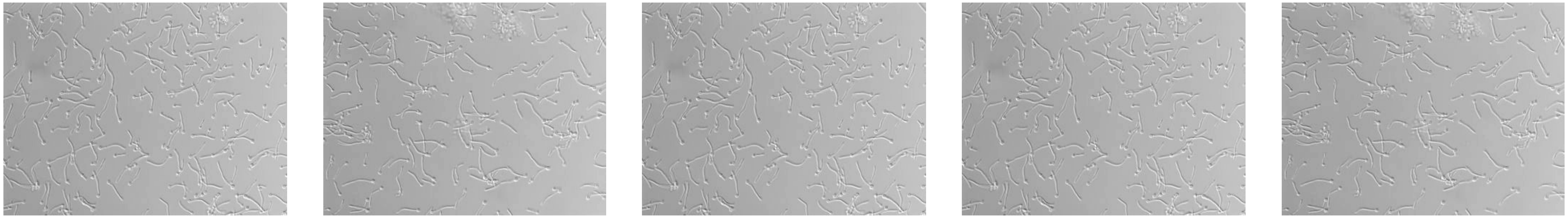

FBS

B404-15

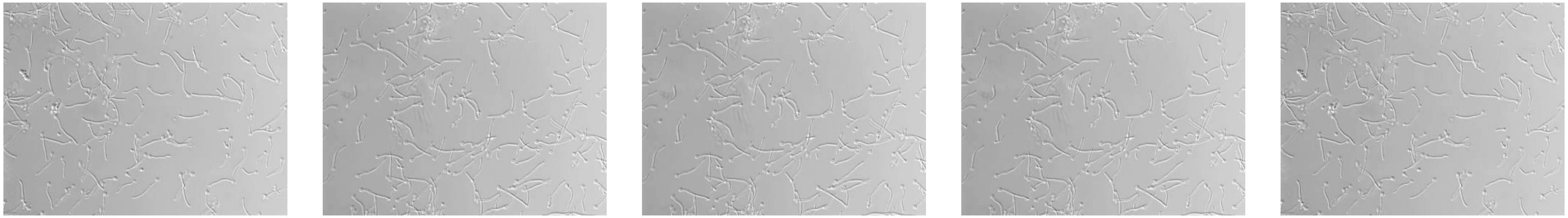

B421-15

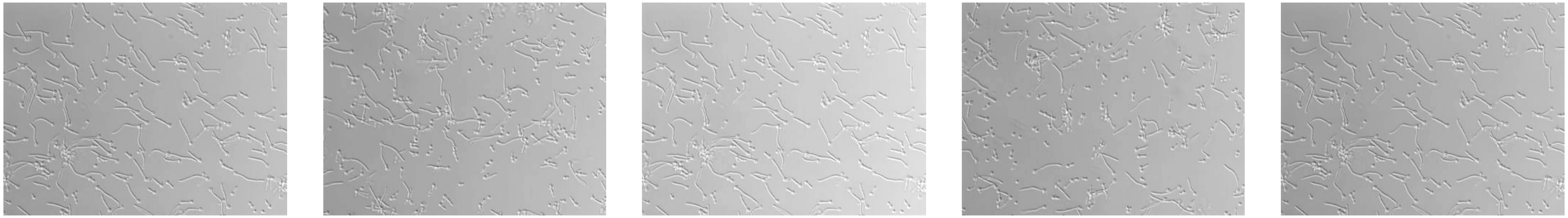

B212-12

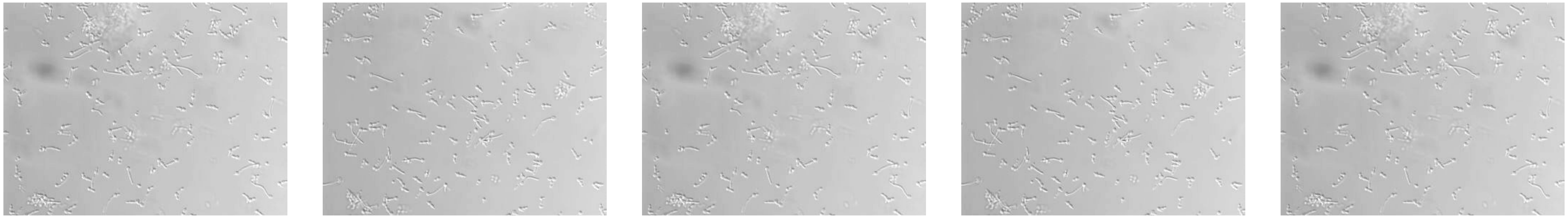

B1091-15

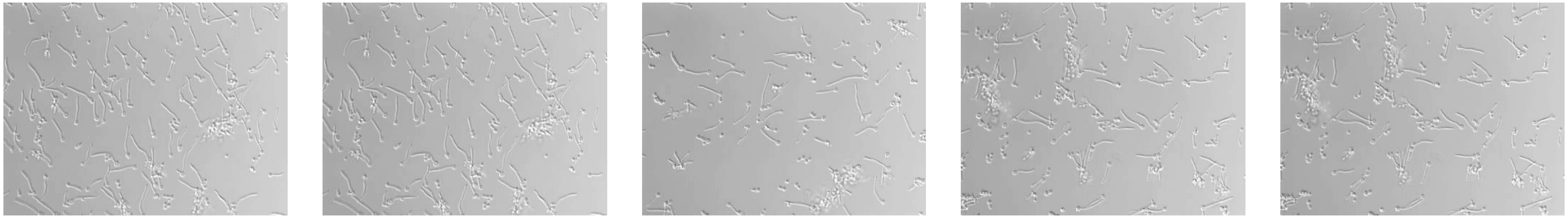

FBS

B510-12

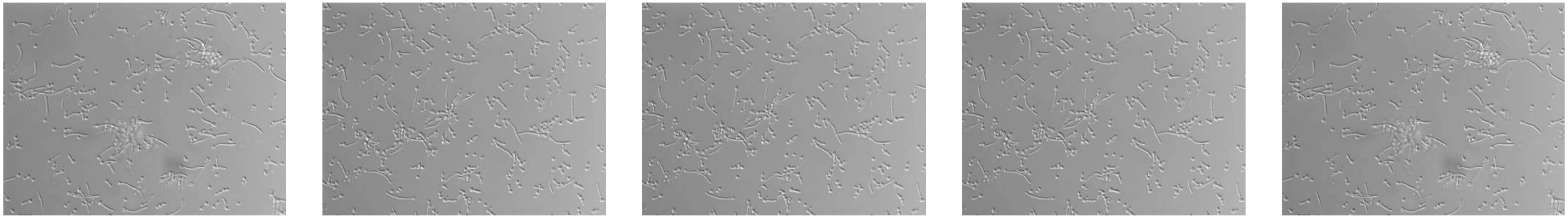

B564-15

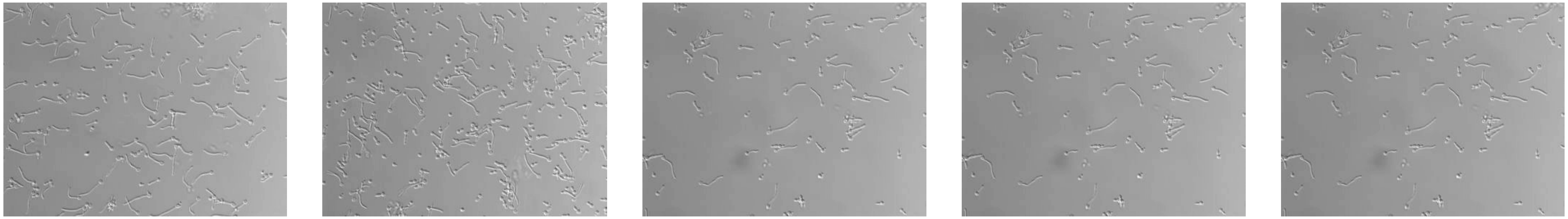

B1168-15

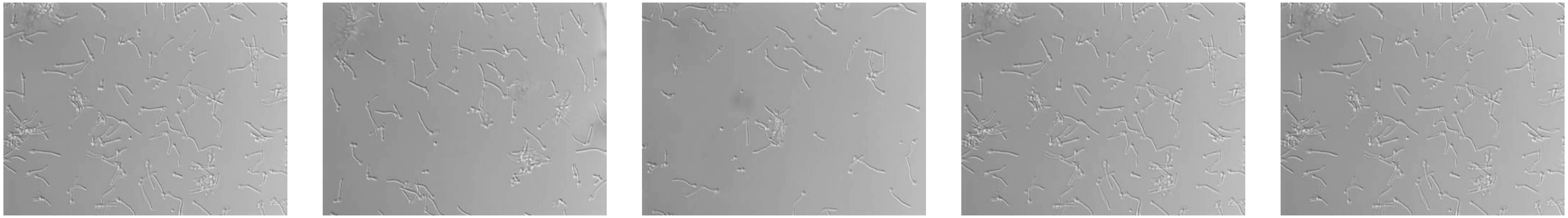

B568-15

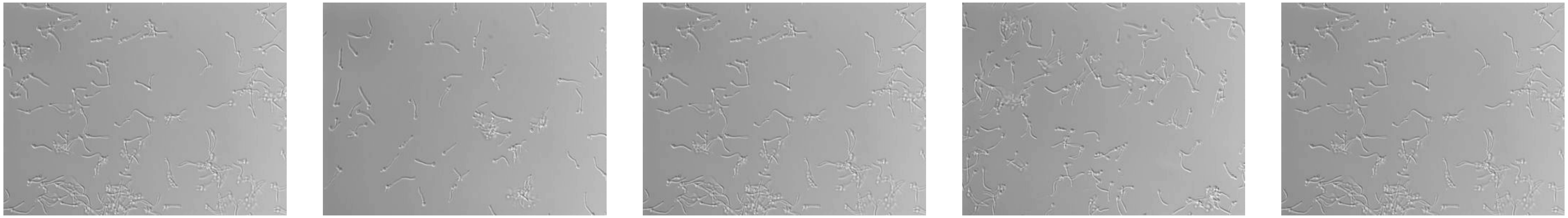

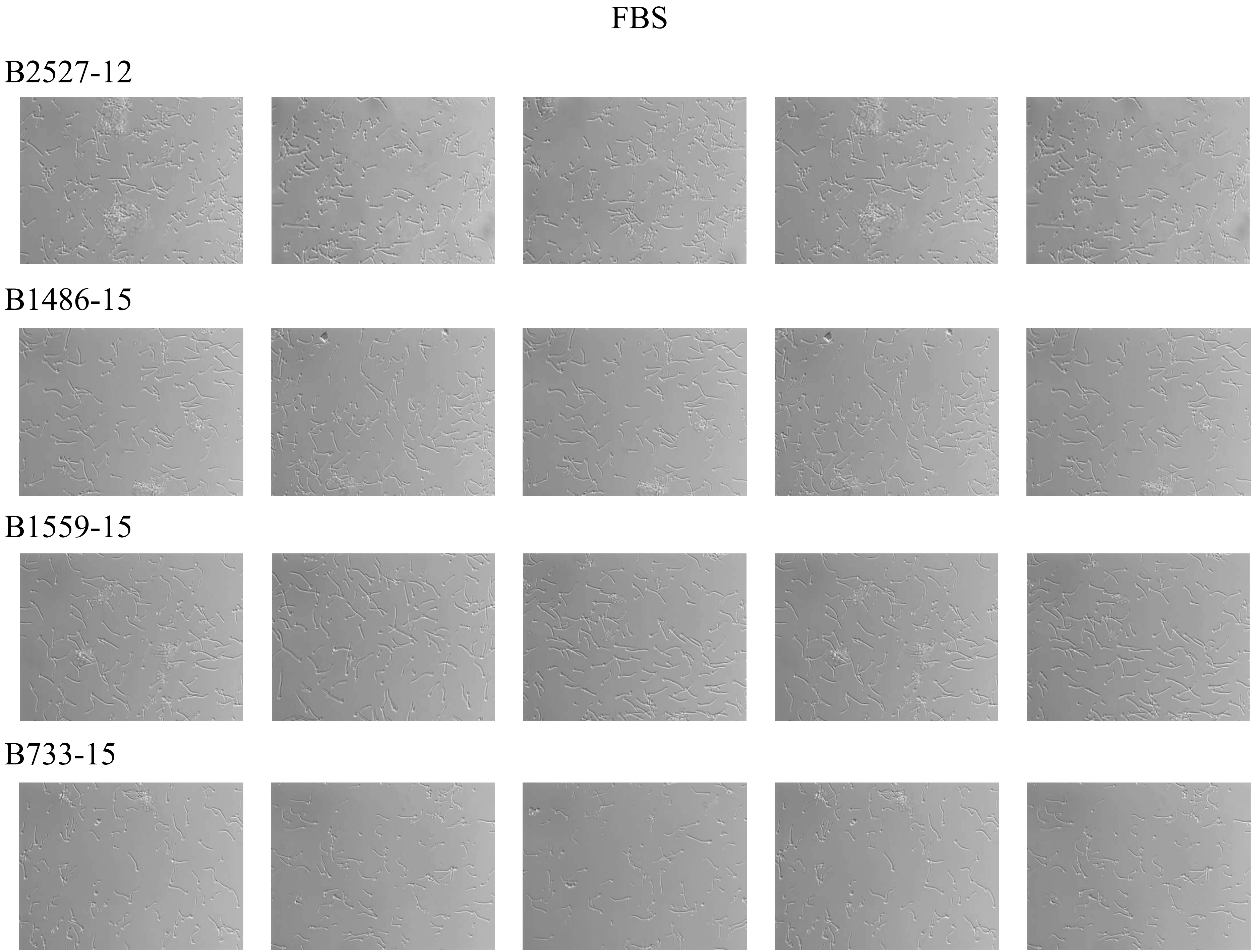
FBS
B2527-12
B1486-15
B1559-15
B733-15

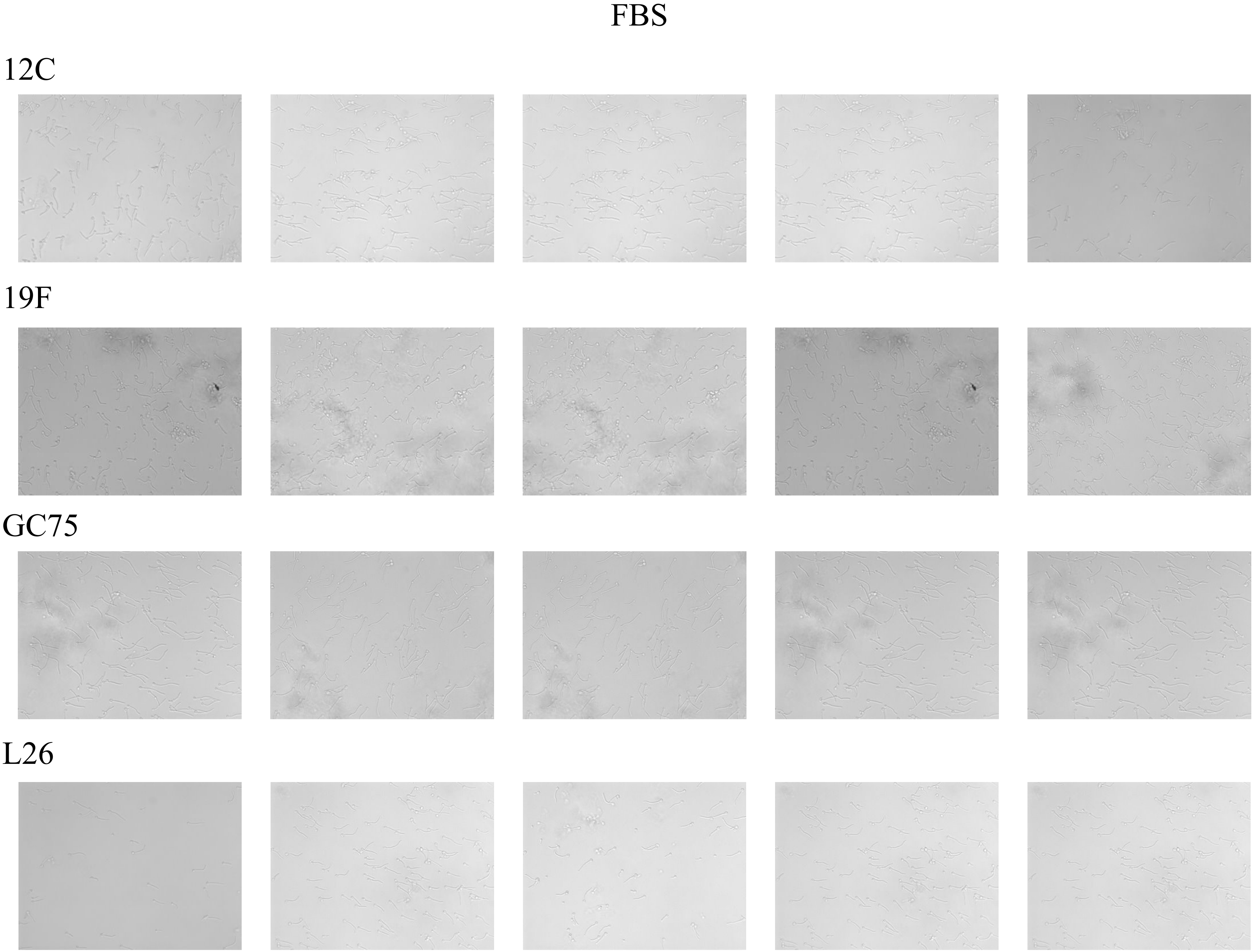
FBS
12C
19F
GC75
L26

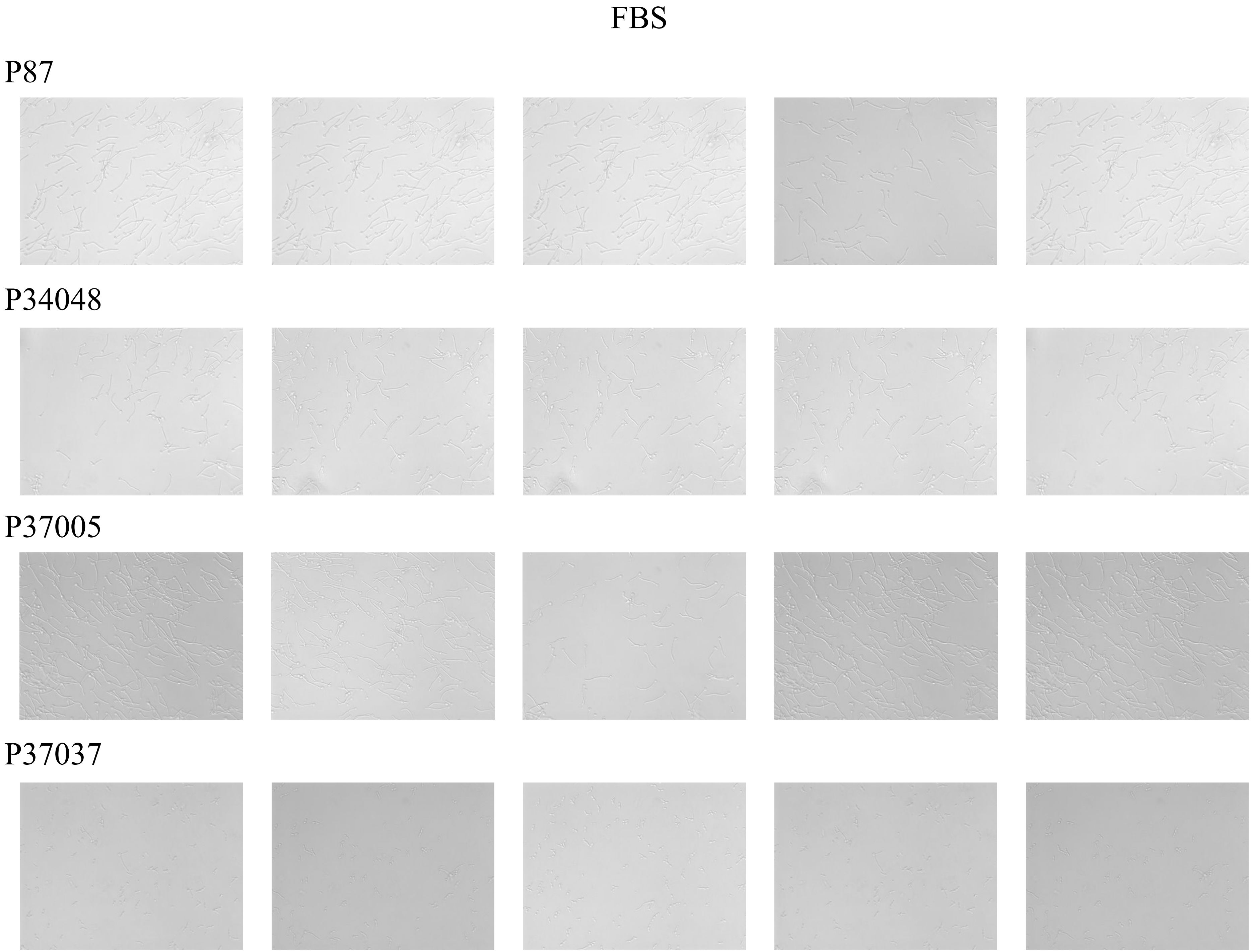
FBS
P87
P34048
P37005
P37037

FBS

P37039

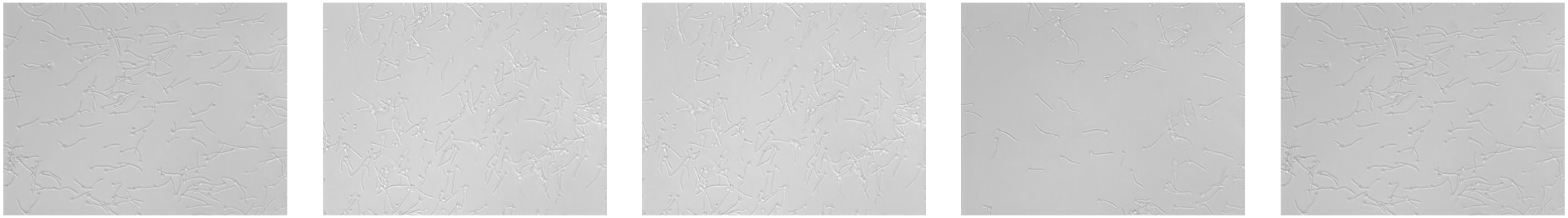

P57055

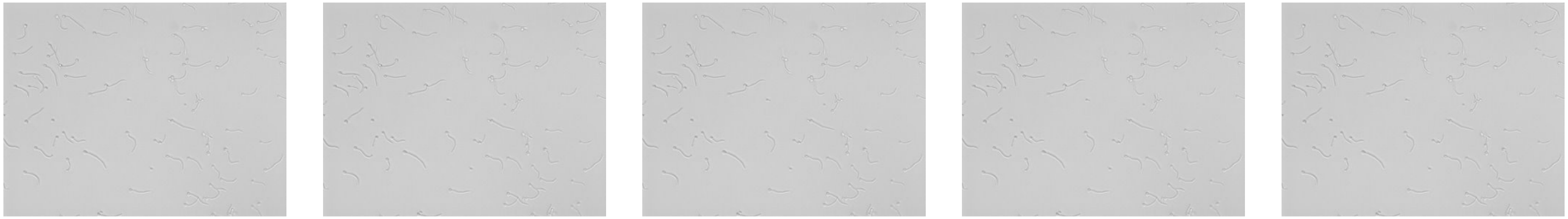

P57072

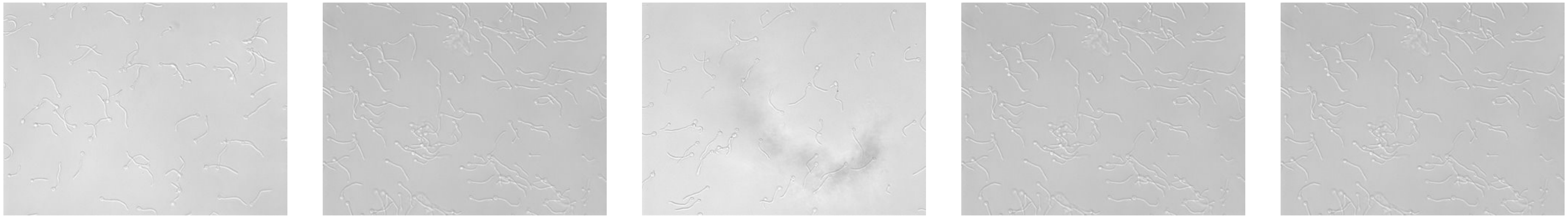

P75010

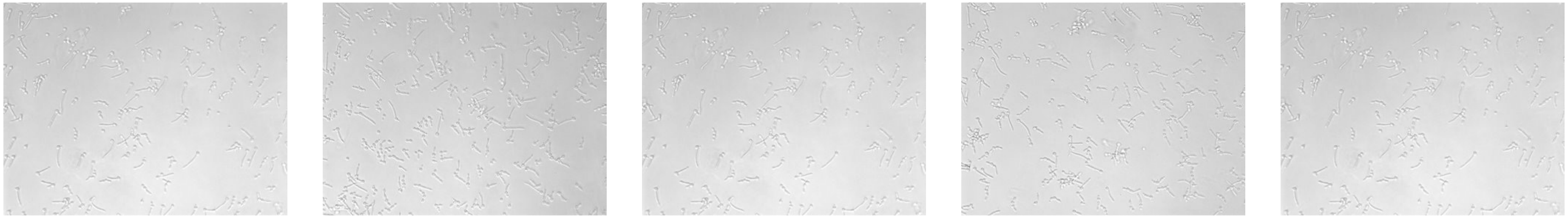

FBS

P75016

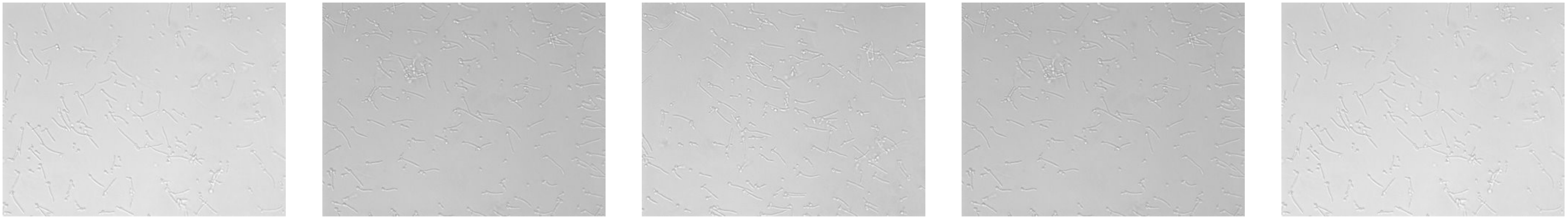

P75063

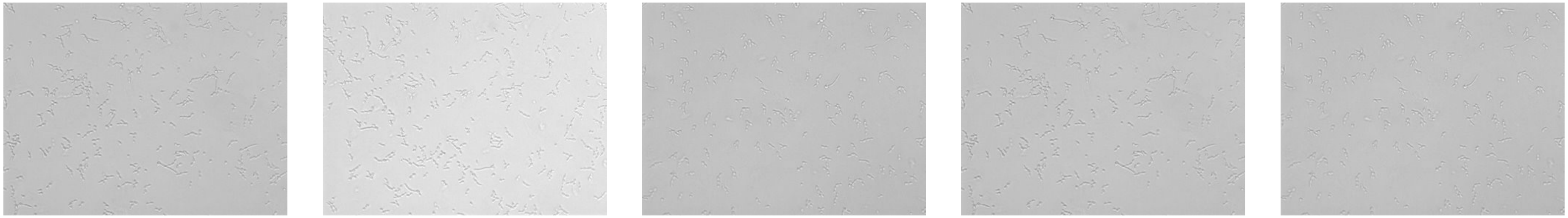

P76055

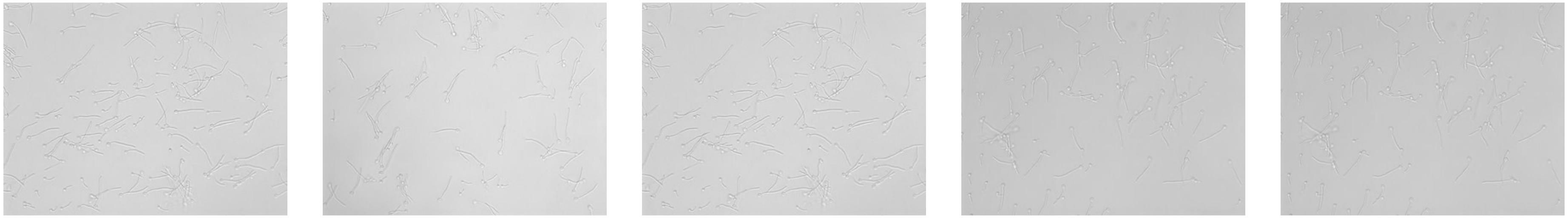

P76067

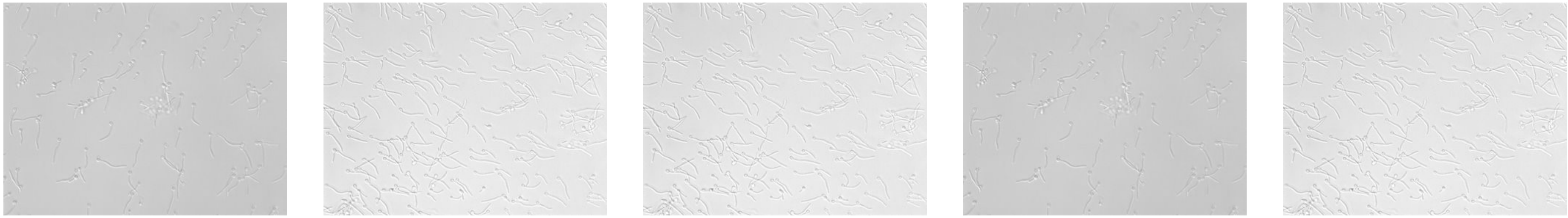

FBS

P78042

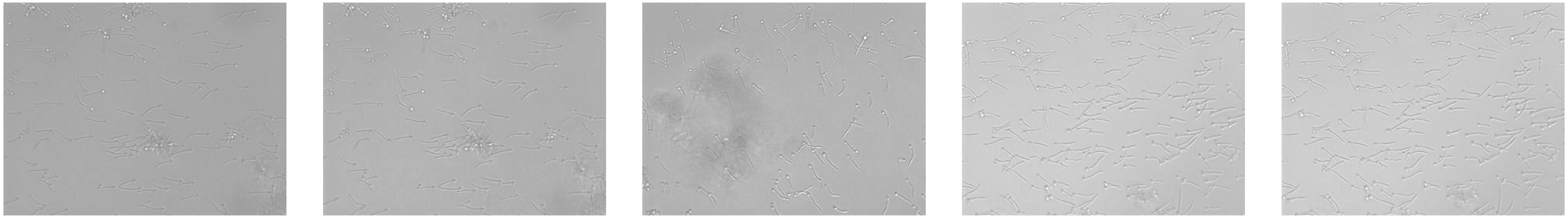

P78048

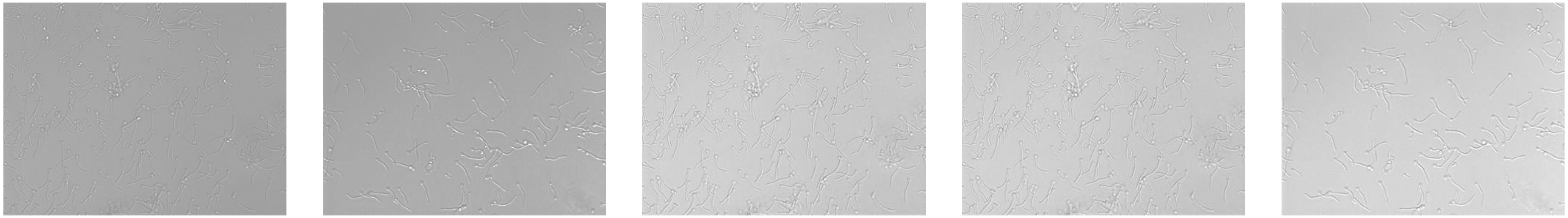

P94015

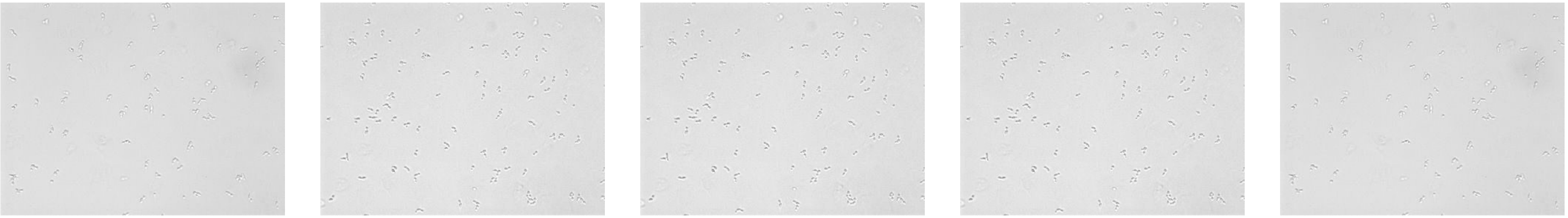

P60002

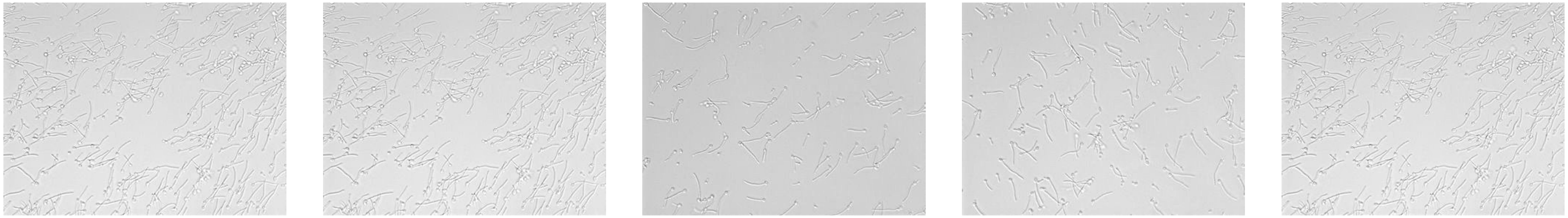

Lee's

B444-12

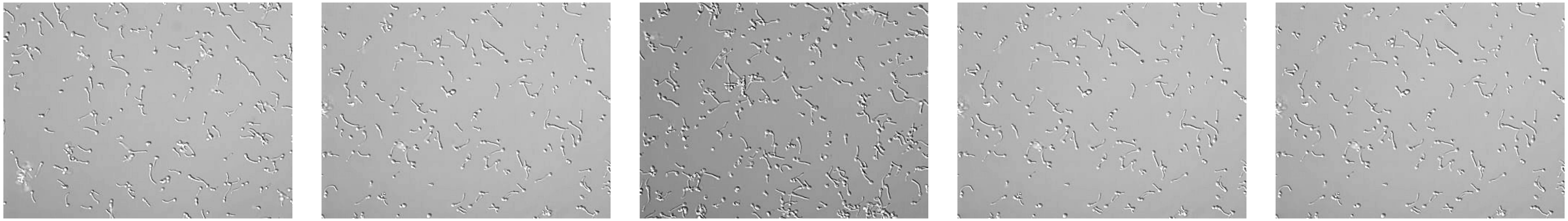

B1257-15

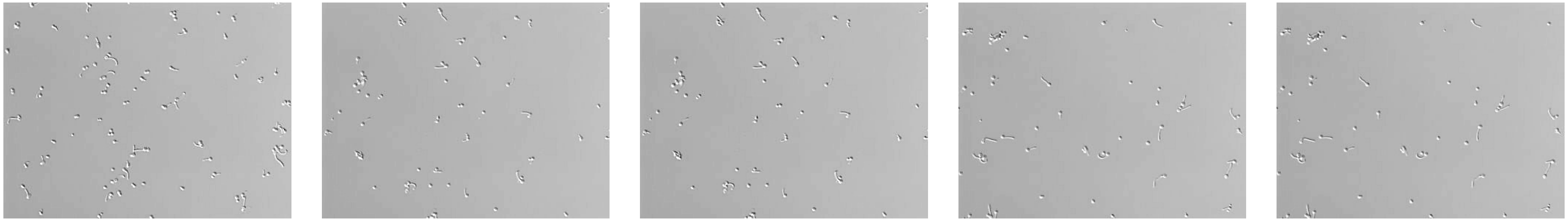

B687-15

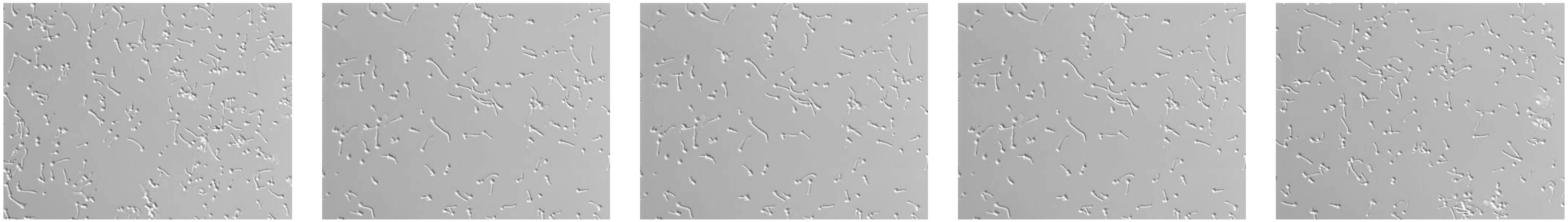

B1762-15

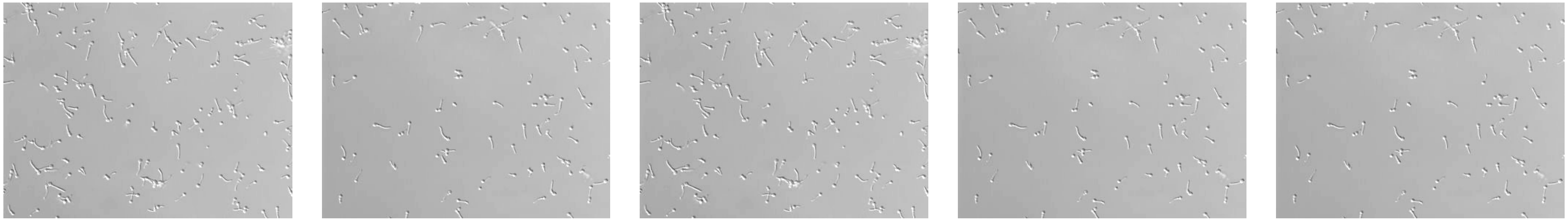

Lee's

B46-15

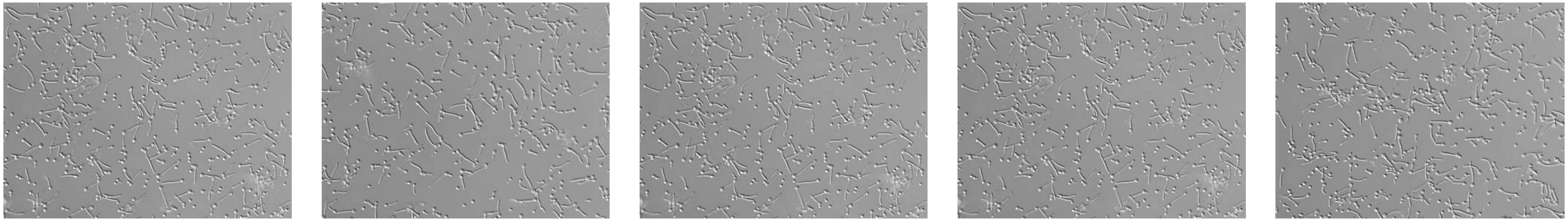

B808-15

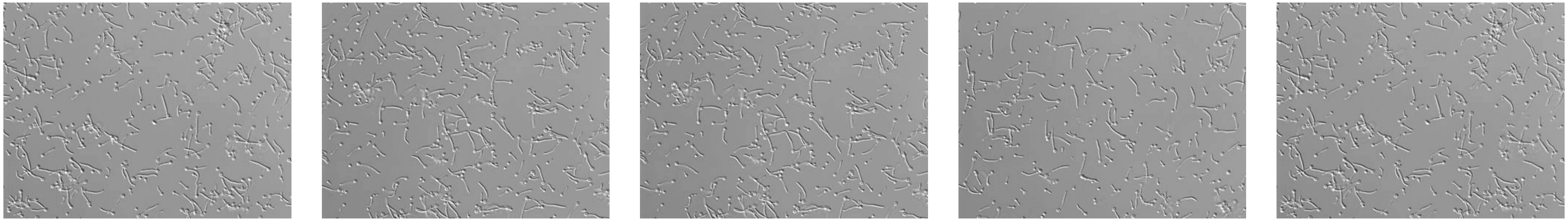

B527-15

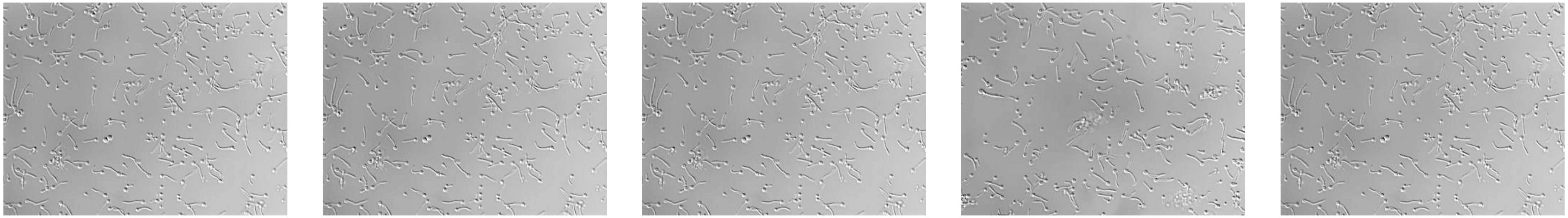

B618-15

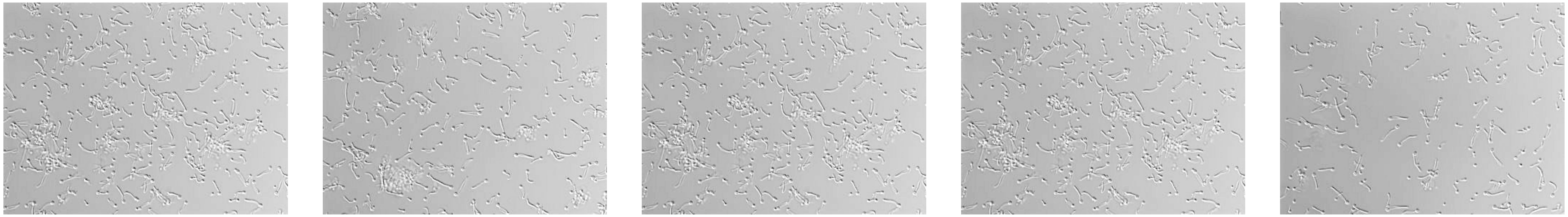

Lee's

B404-15

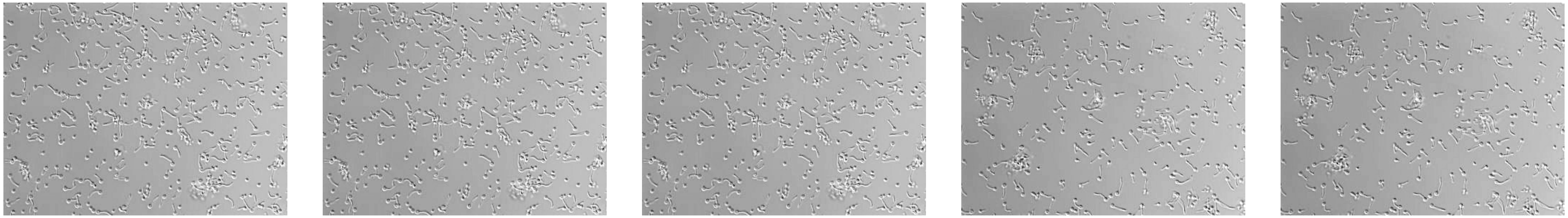

B421-15

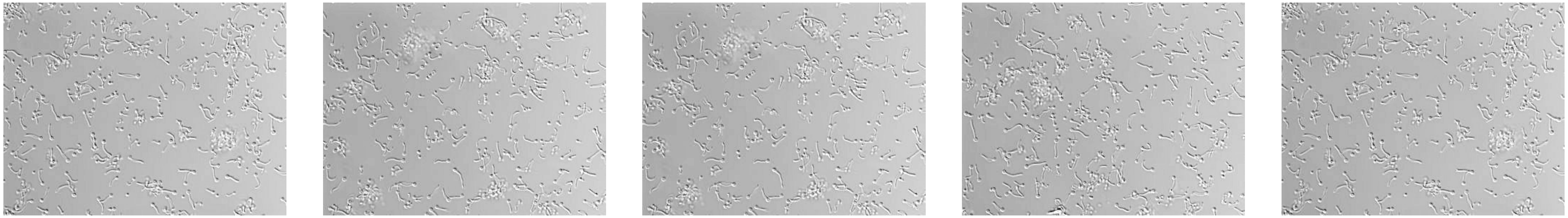

B212-12

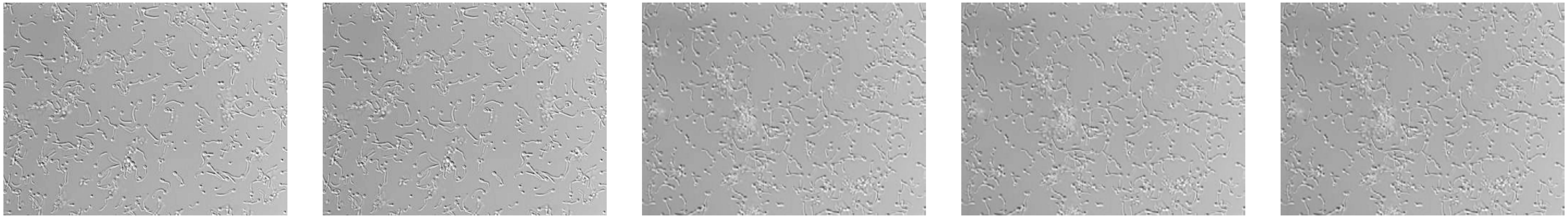

B1091-15

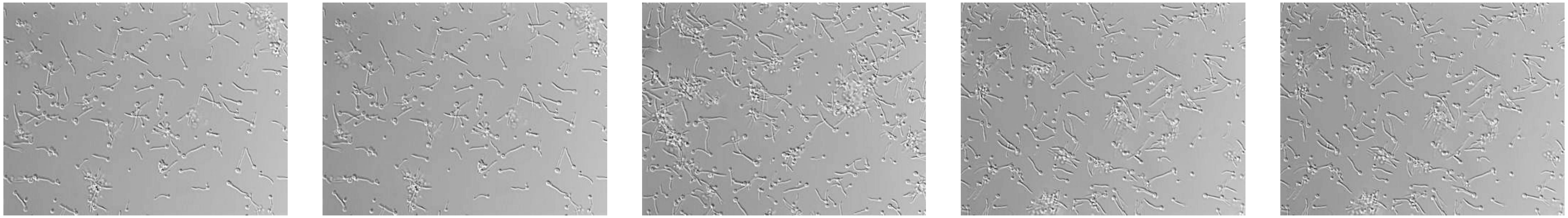

Lee's

B510-12

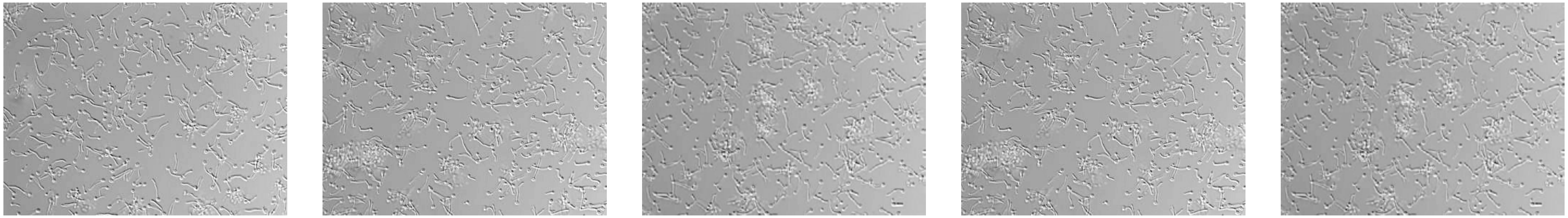

B564-15

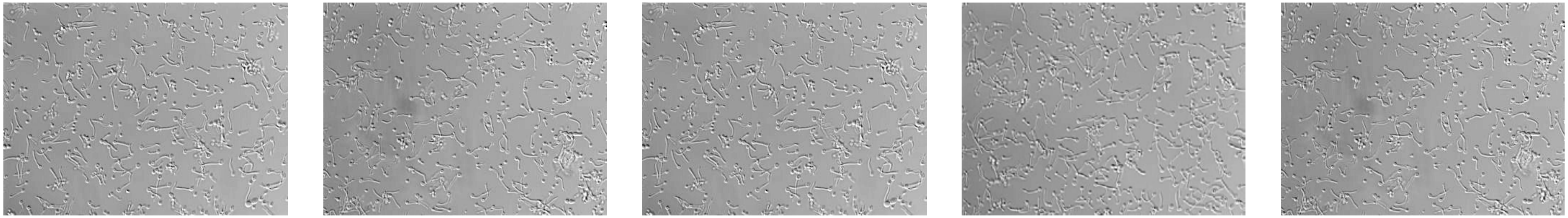

B1168-15

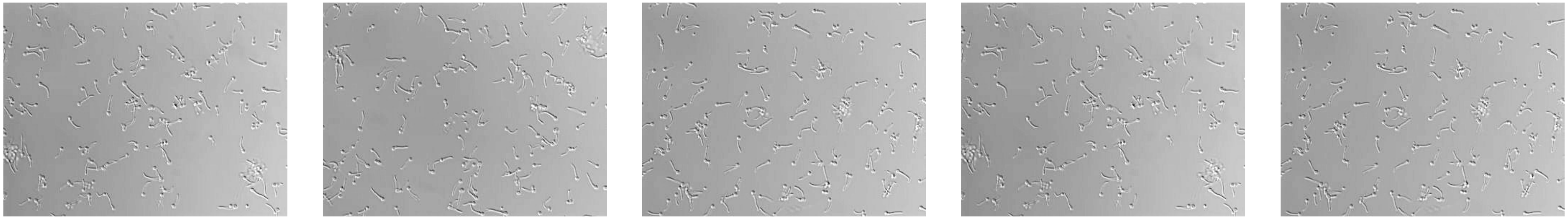

B568-15

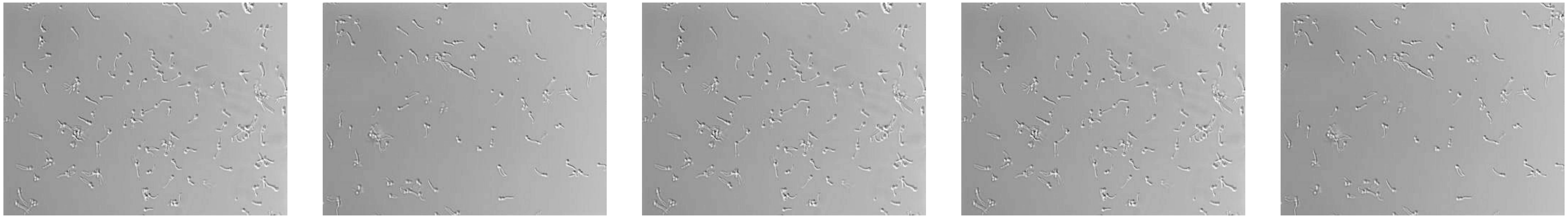

Lee's

B2527-12

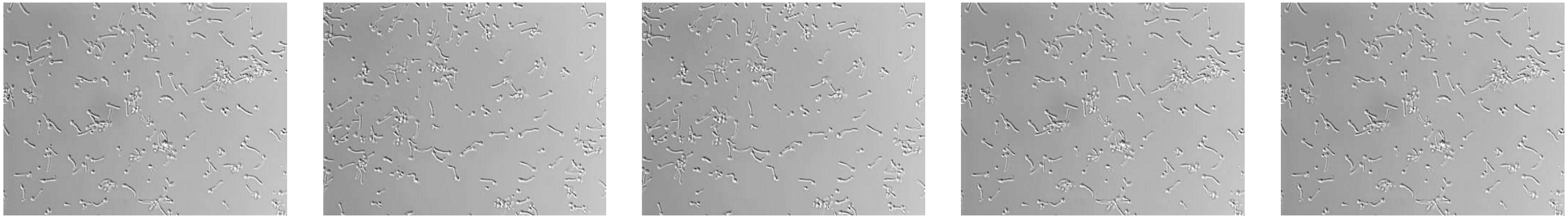

B1486-15

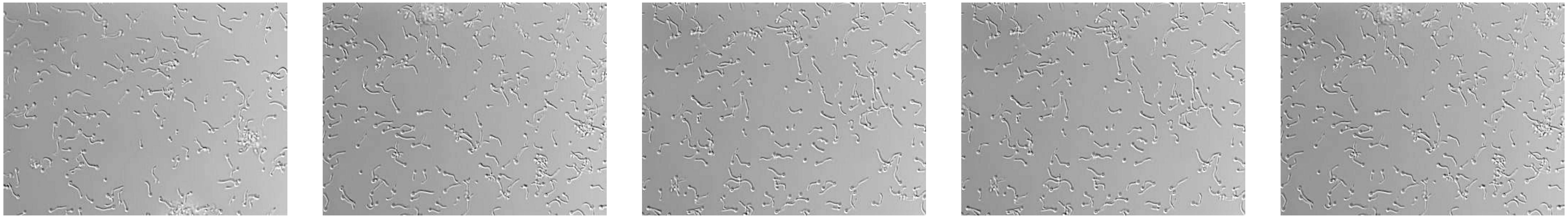

B1559-15

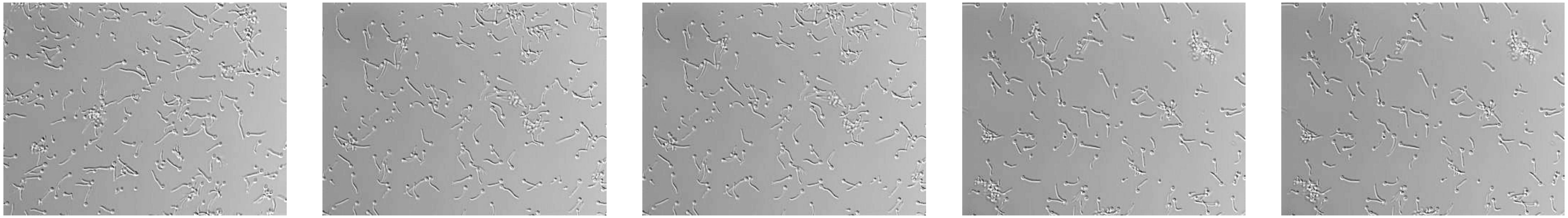

B733-15

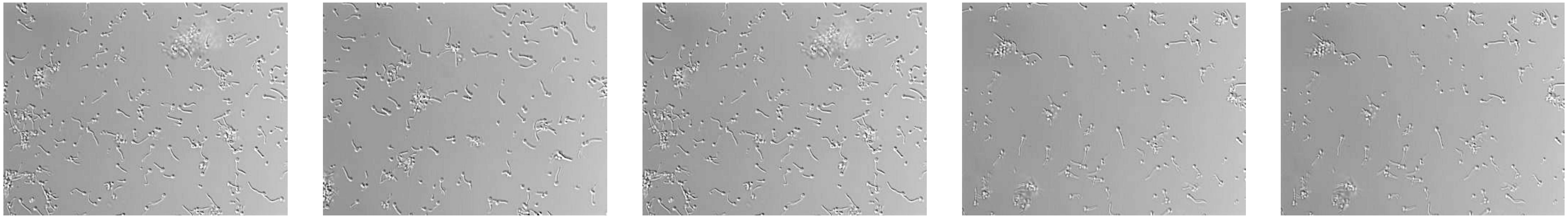

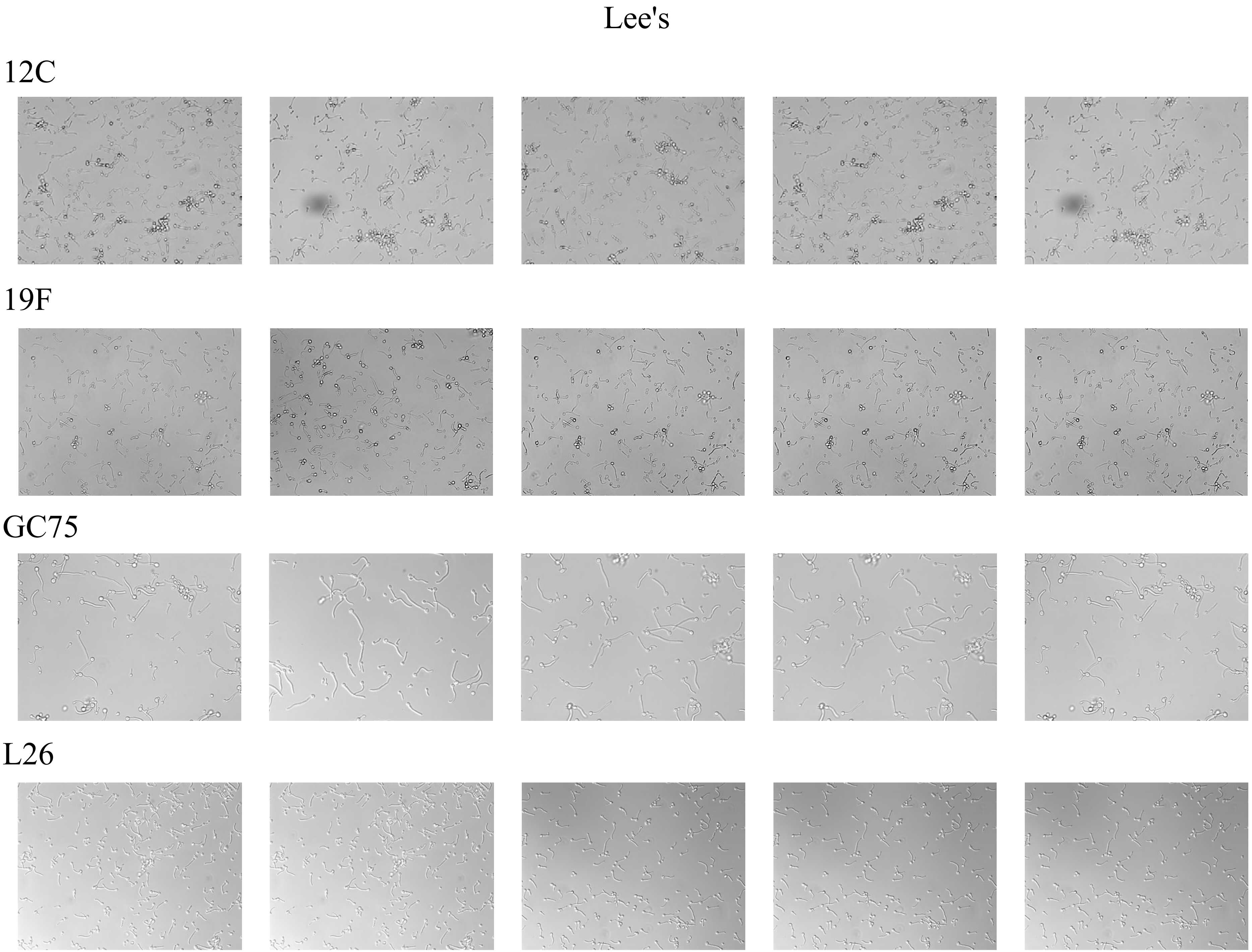
Lee's
12C
19F
GC75
L26

Lee's

P87

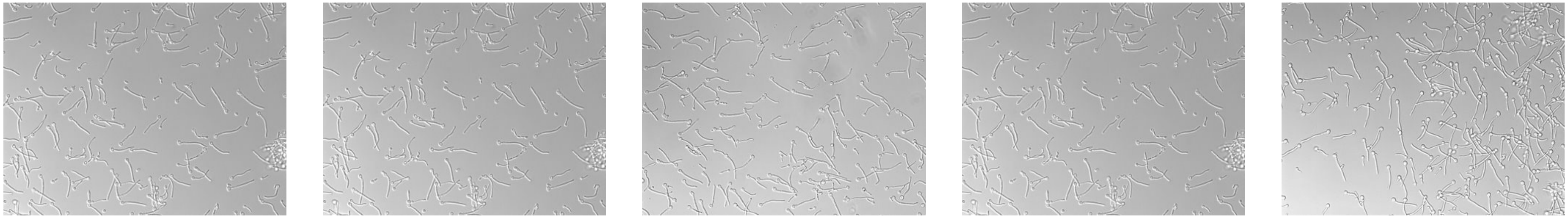

P34048

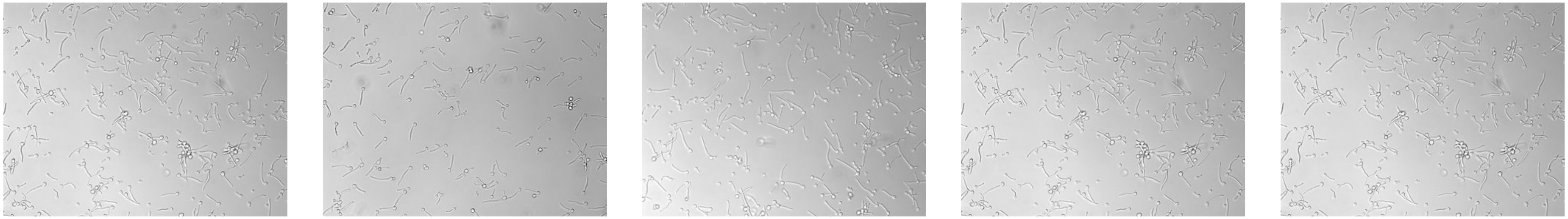

P37005

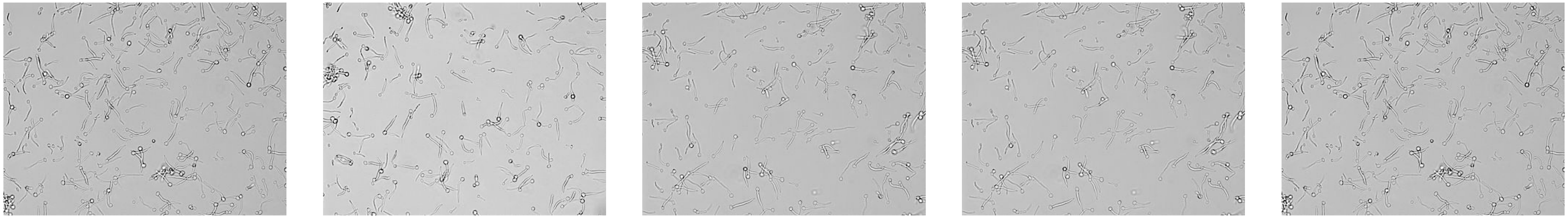

P37037

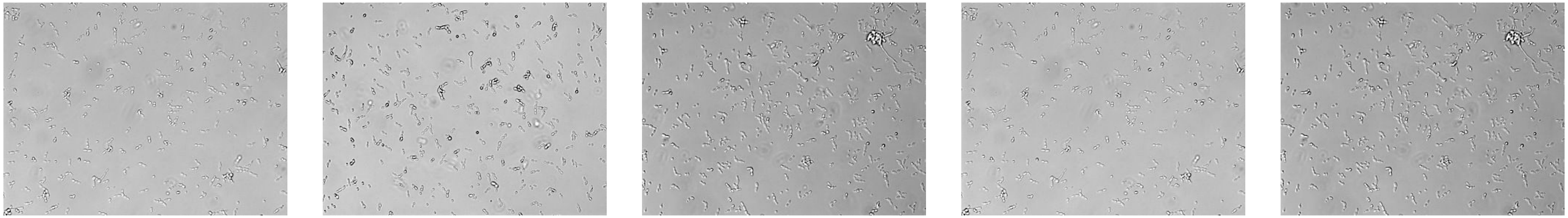

Lee's

P37039

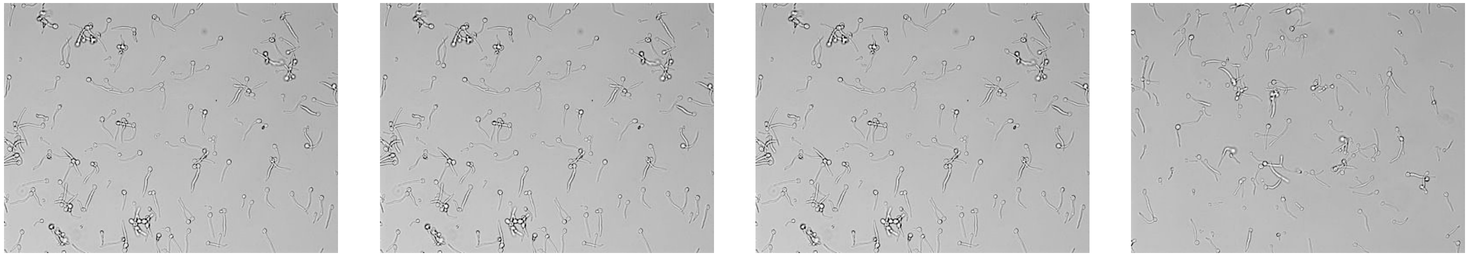

P57055

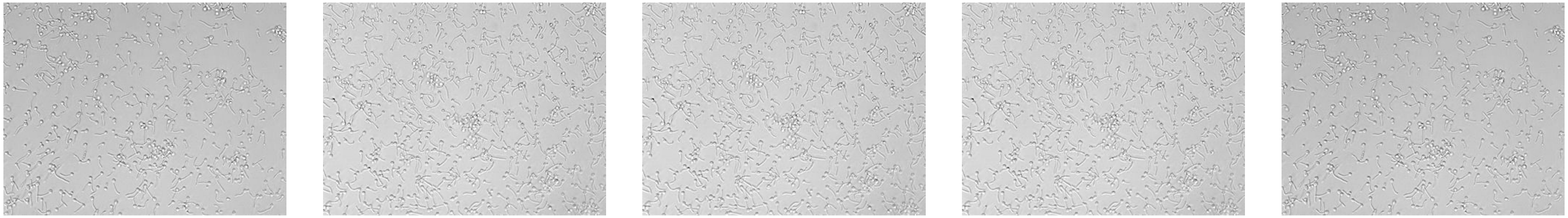

P57072

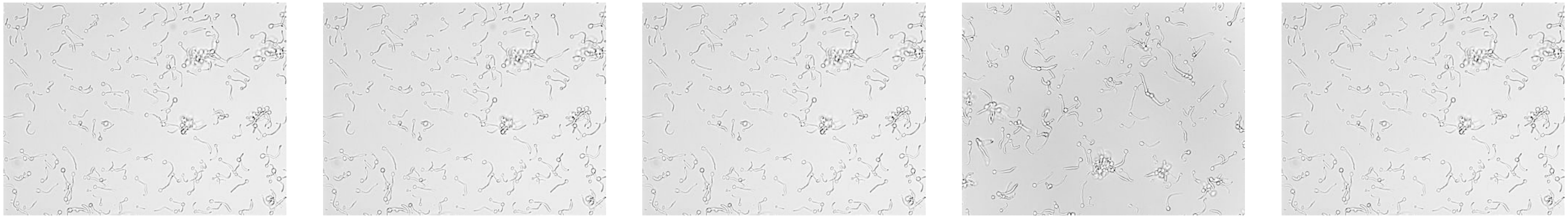

P75010

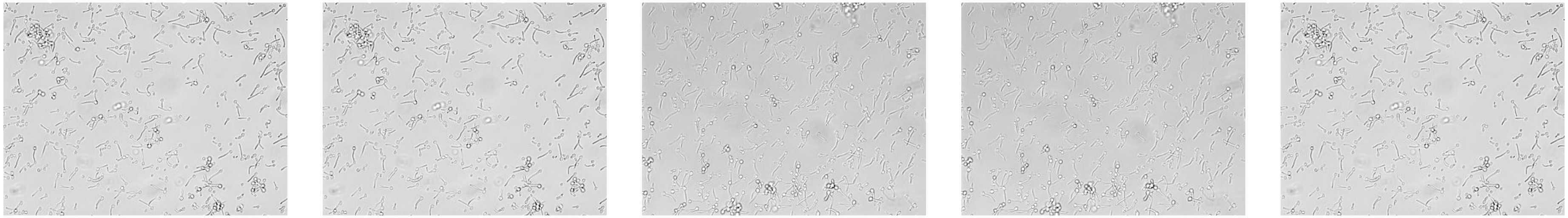

Lee's

P75016

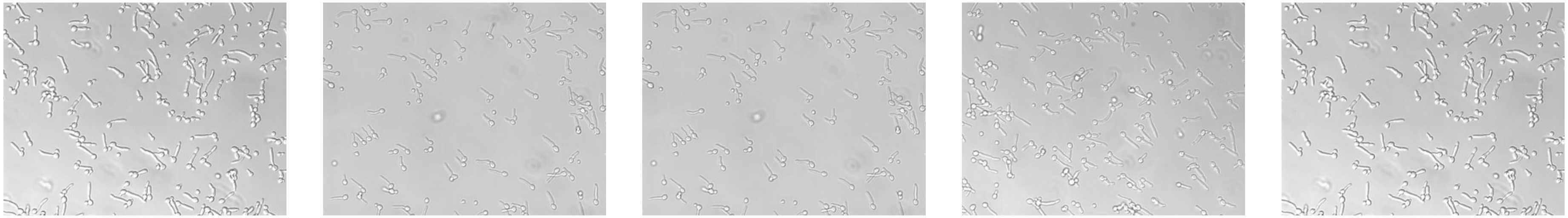

P75063

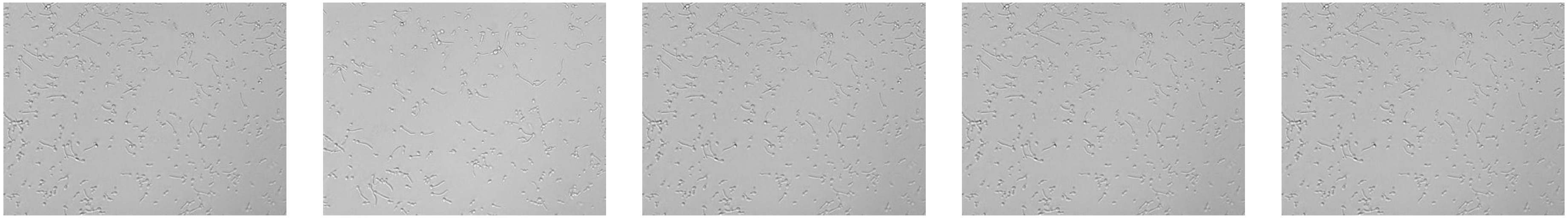

P76055

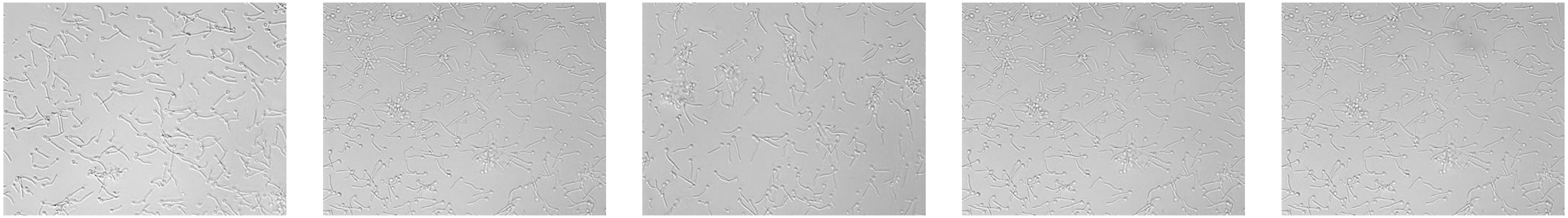

P76067

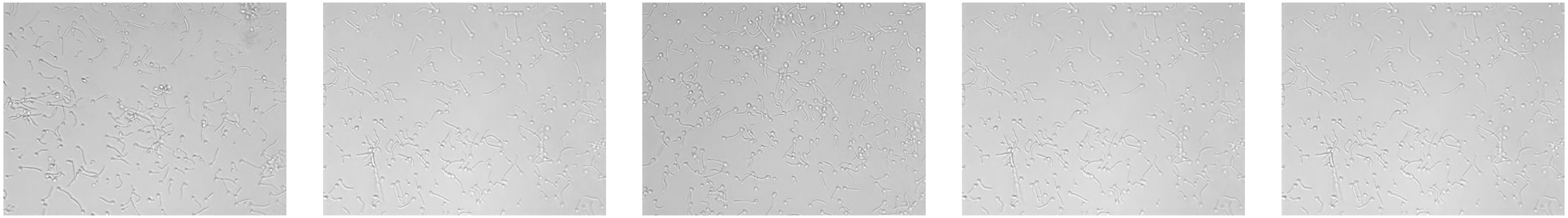

Lee's

P78042

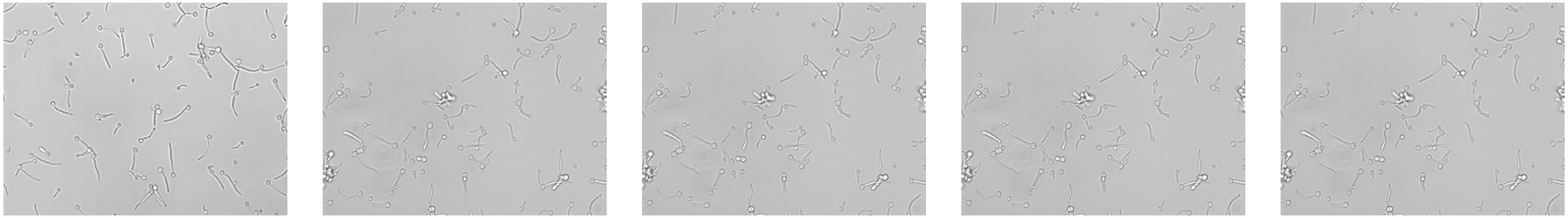

P78048

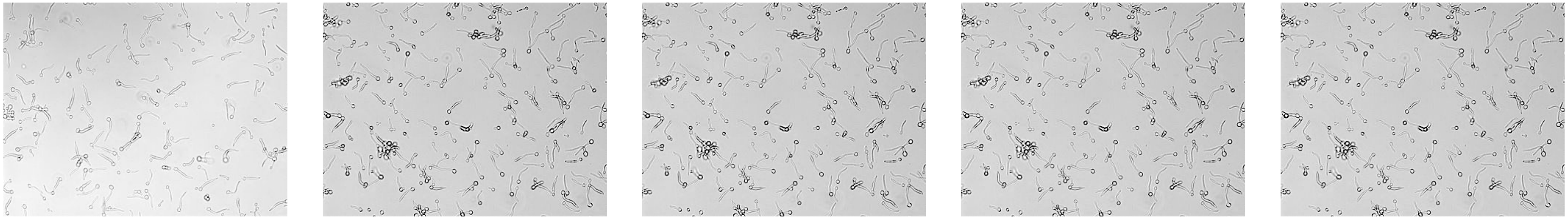

P94015

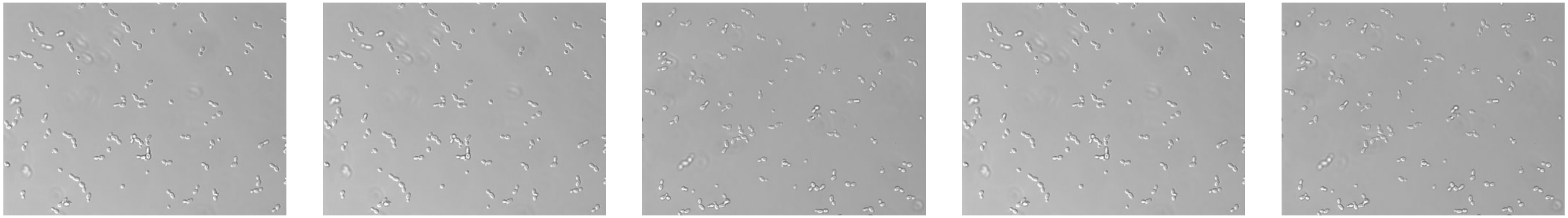

P60002

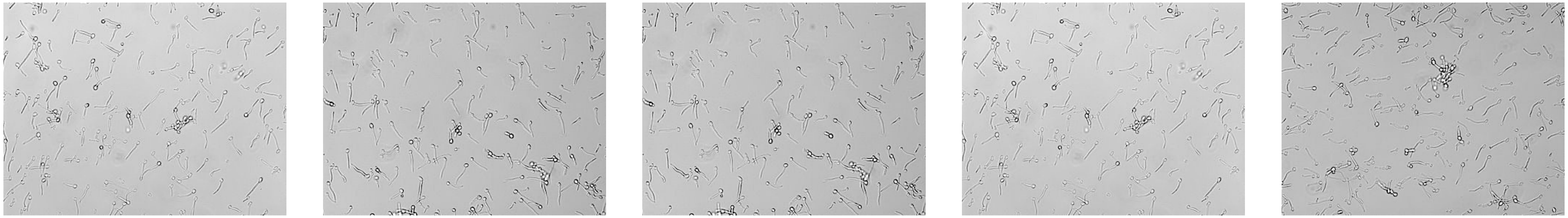

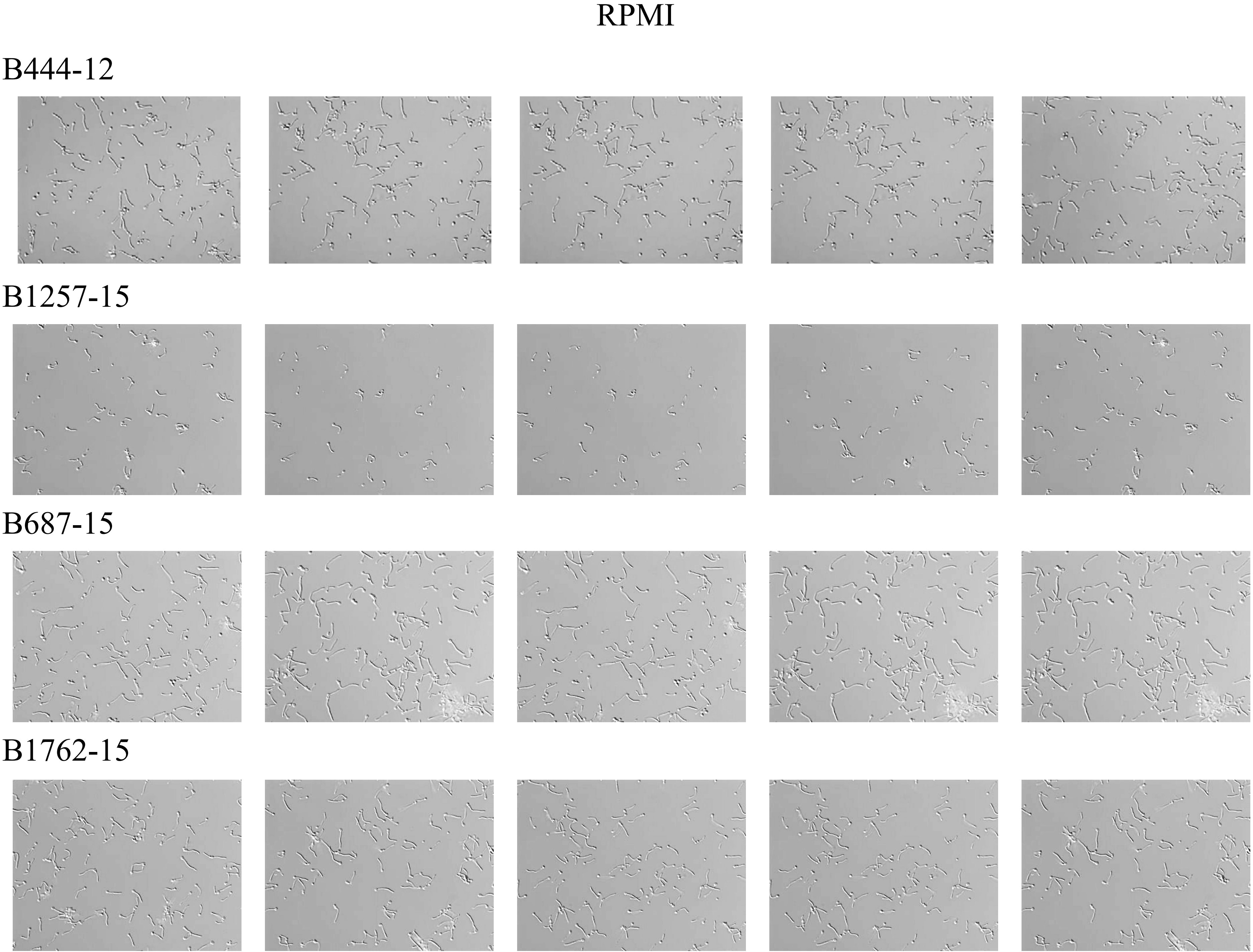
RPMI
B444-12
B1257-15
B687-15
B1762-15

RPMI

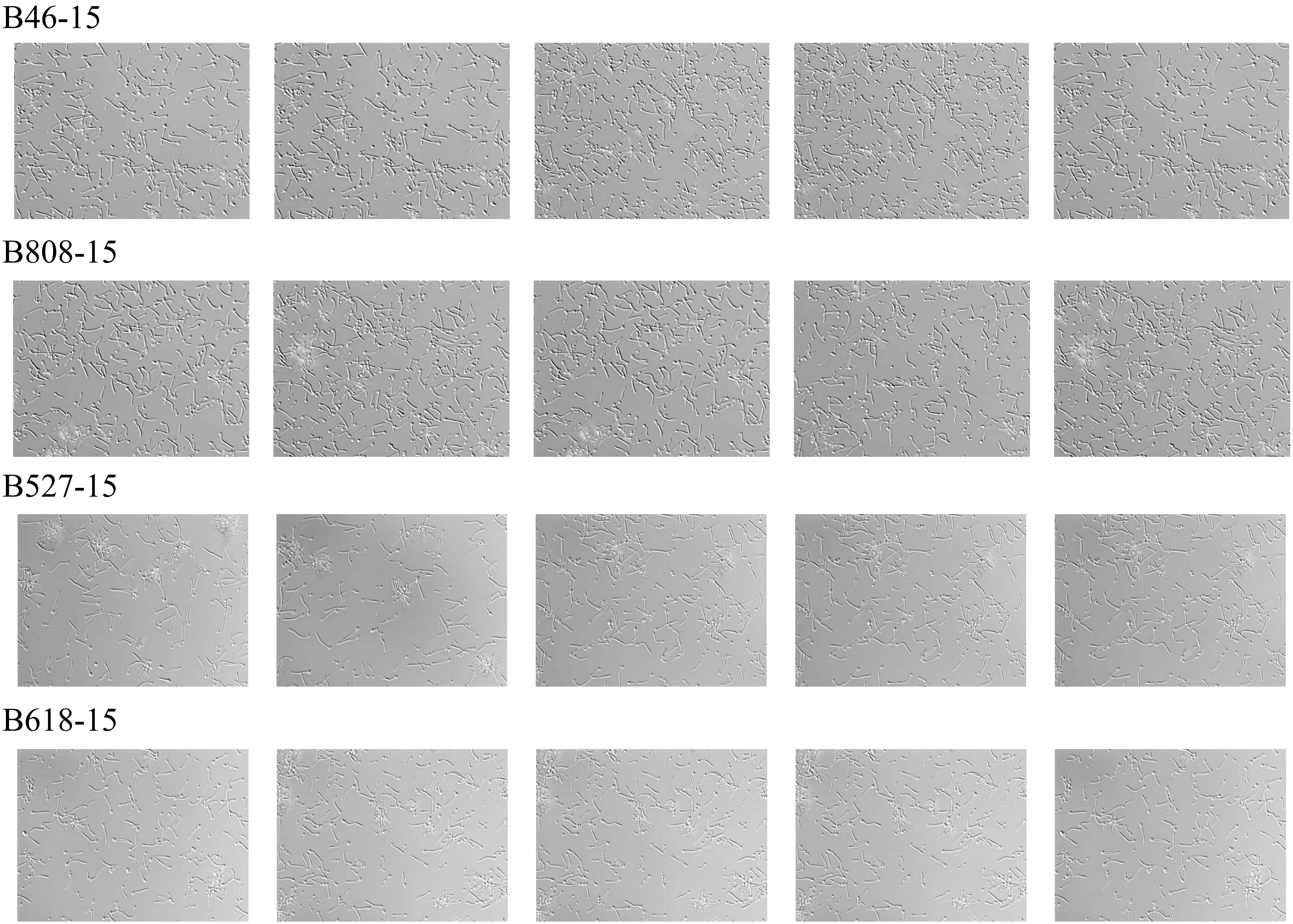

RPMI

B404-15

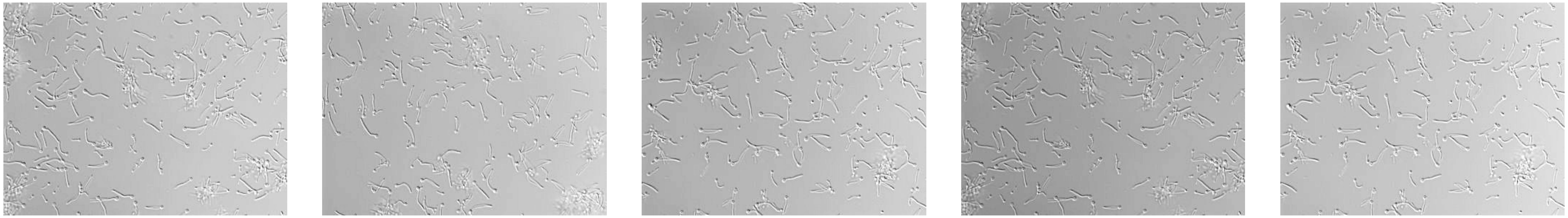

B421-15

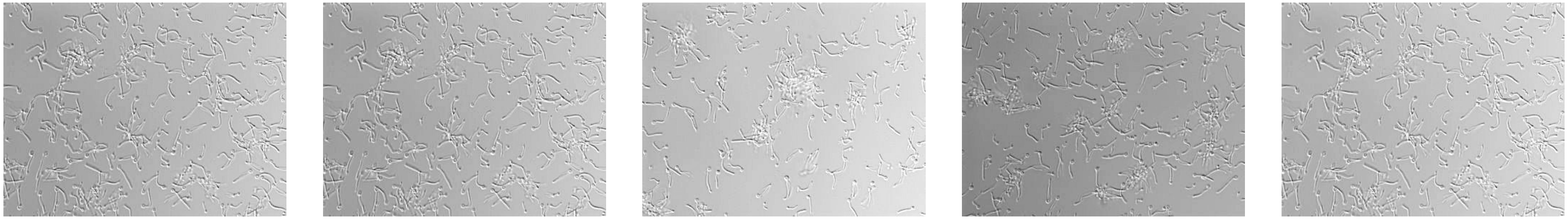

B212-12

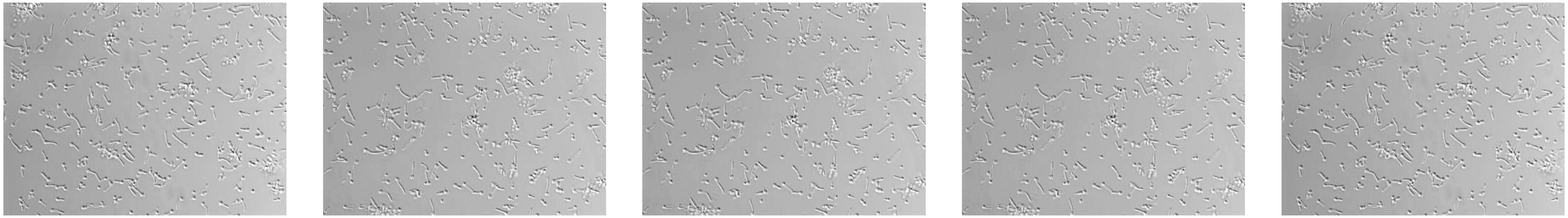

B1091-15

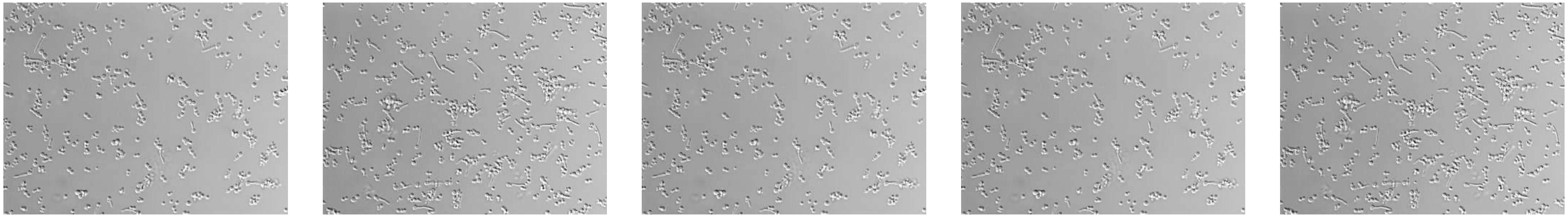

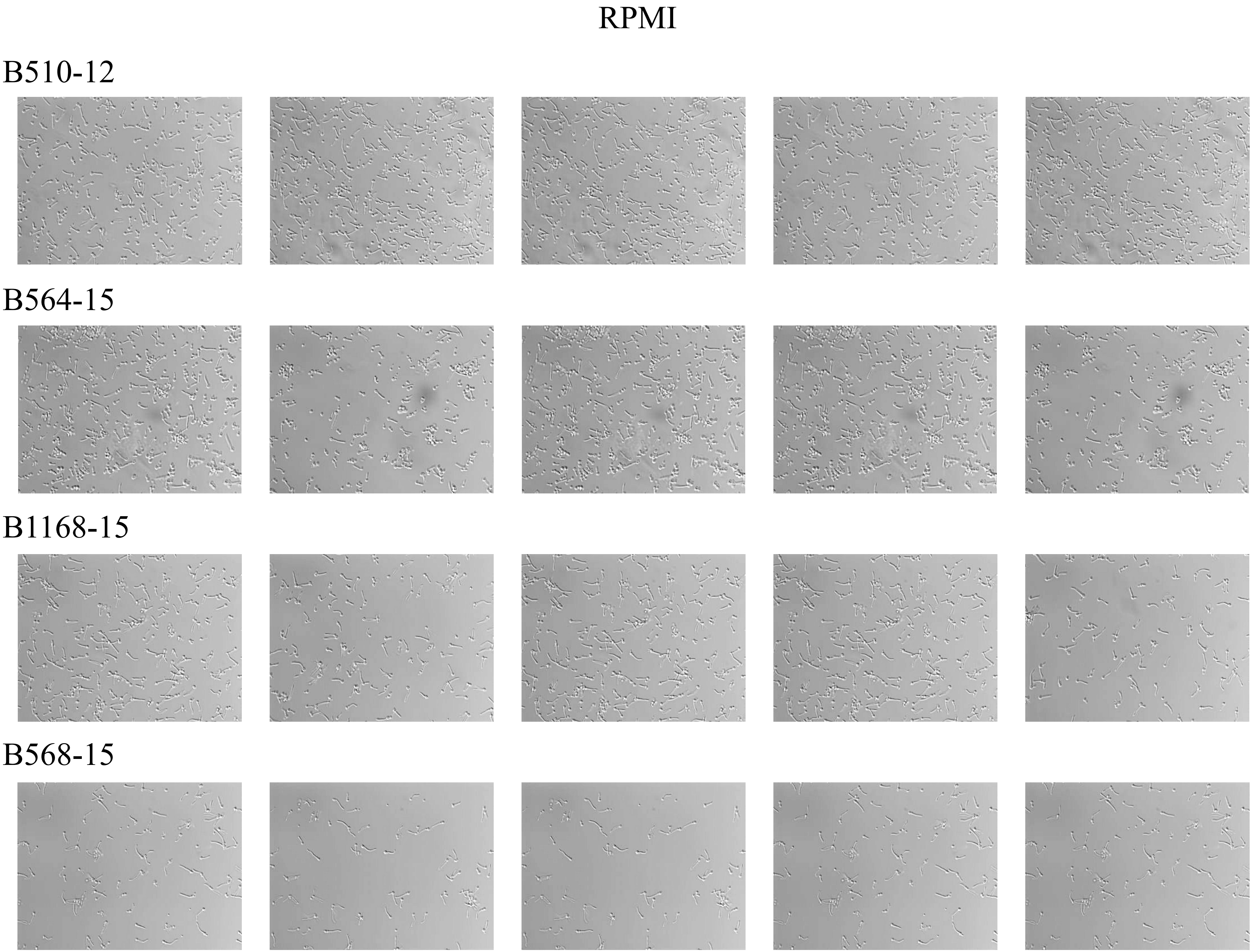
RPMI
B510-12
B564-15
B1168-15
B568-15

RPMI

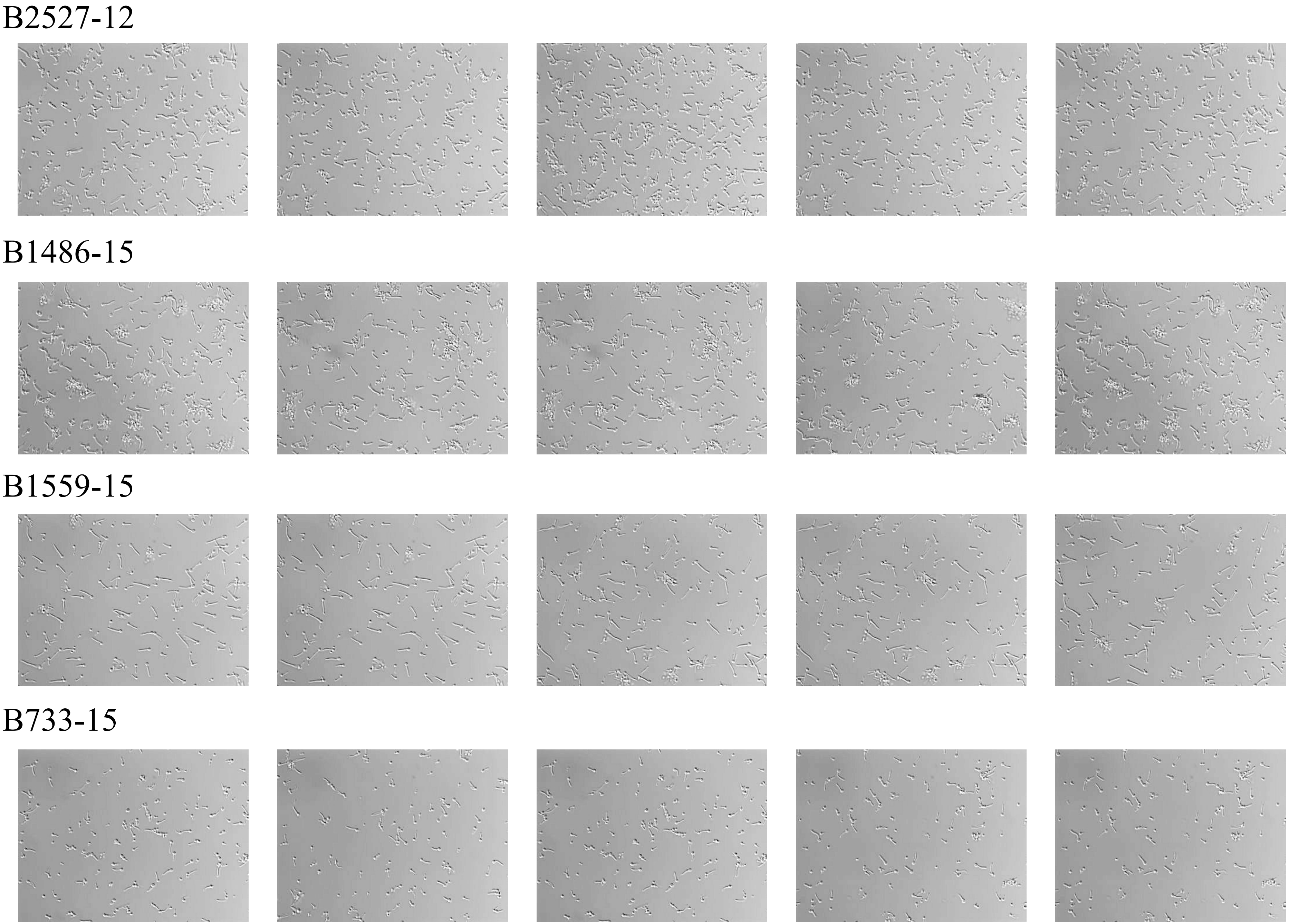

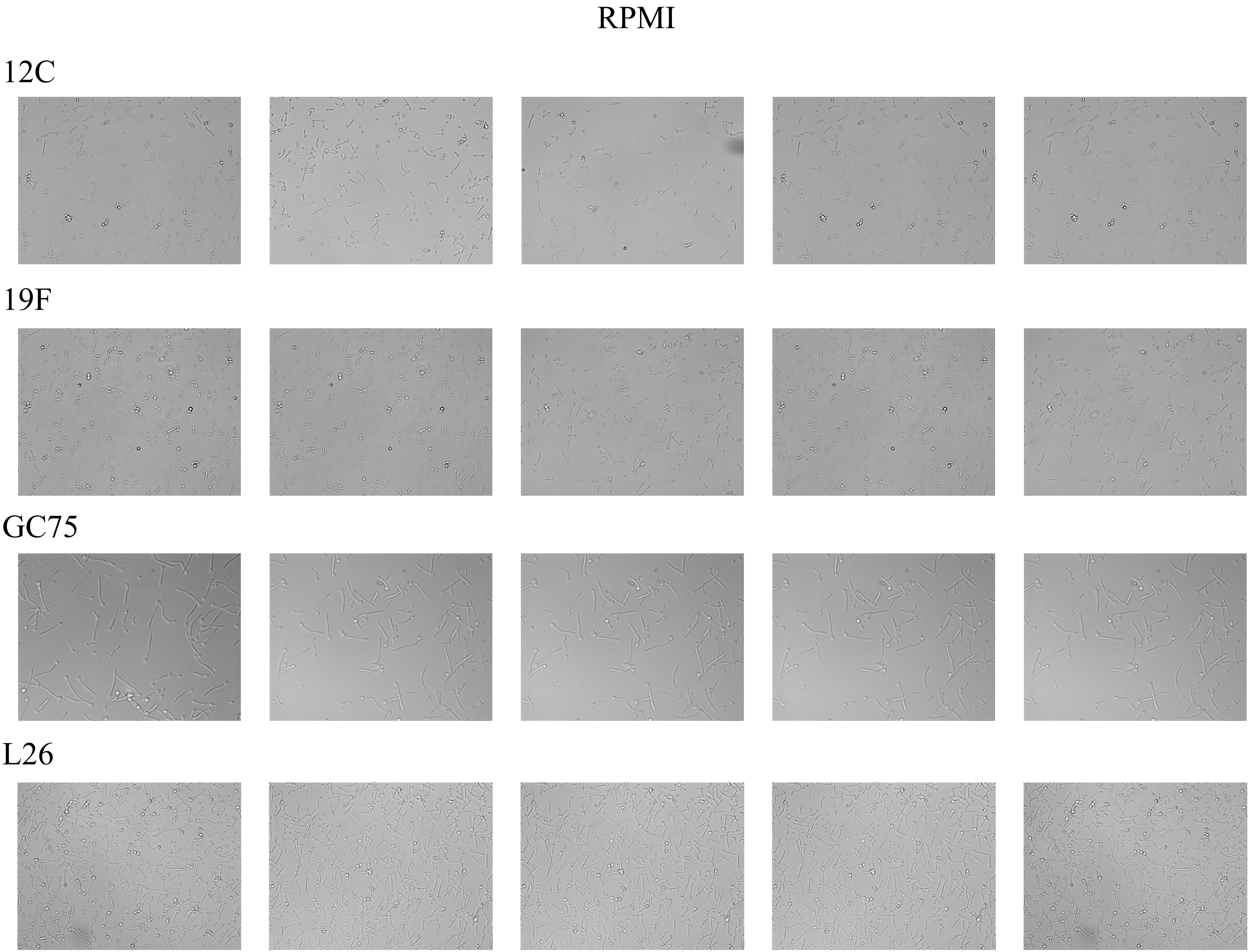
RPMI
12C
19F
GC75
L26

RPMI

P87

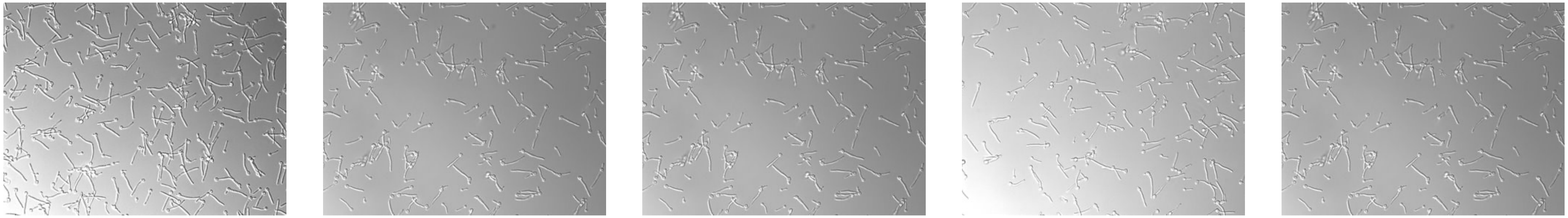

P34048

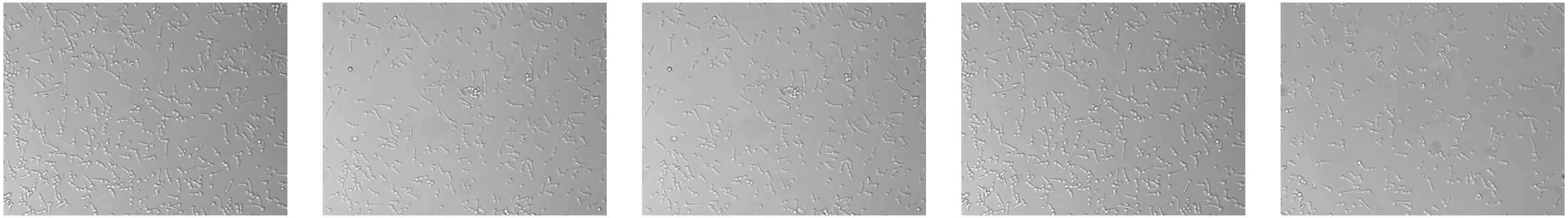

P37005

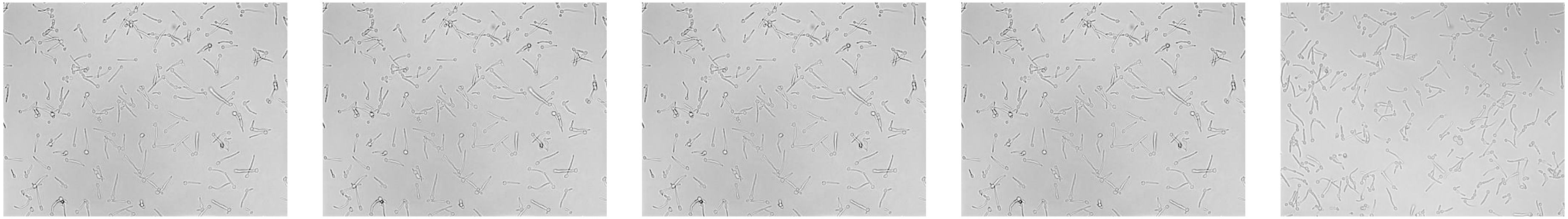

P37037

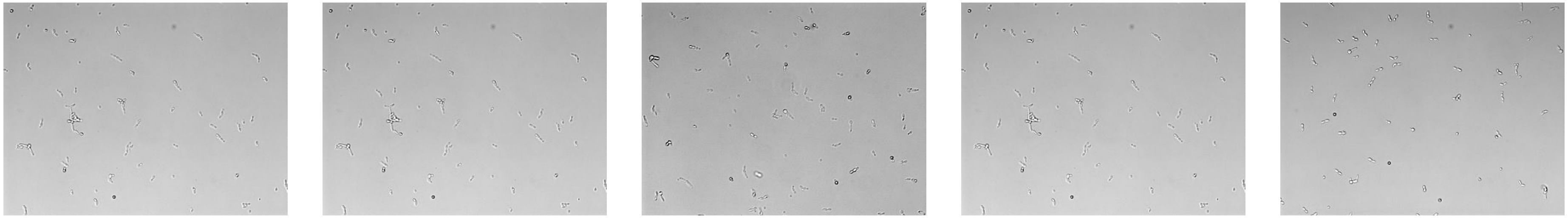

RPMI

P37039

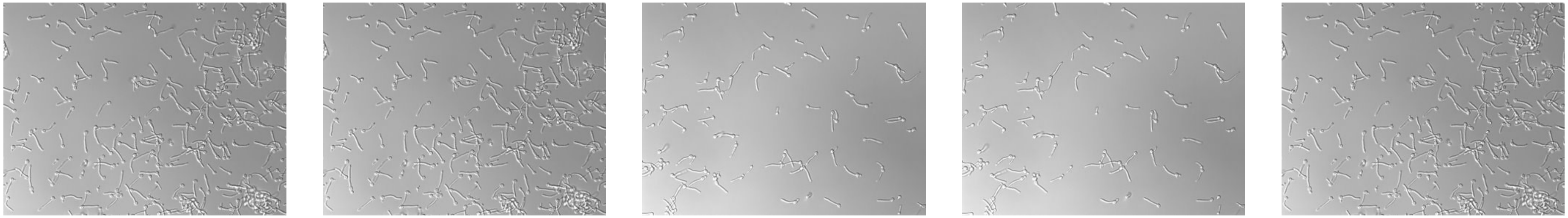

P57055

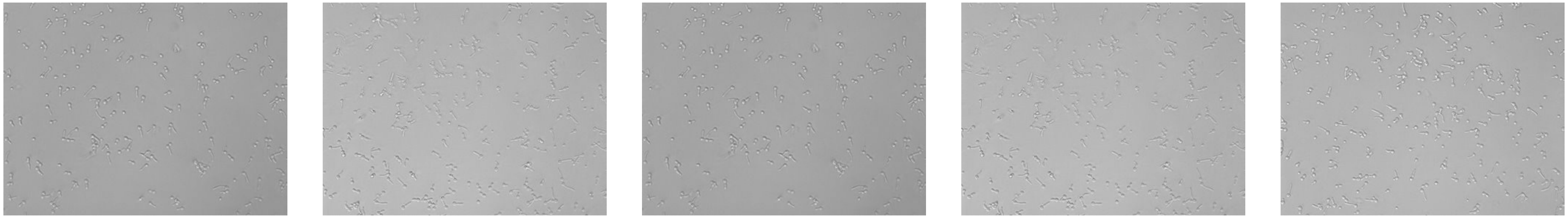

P57072

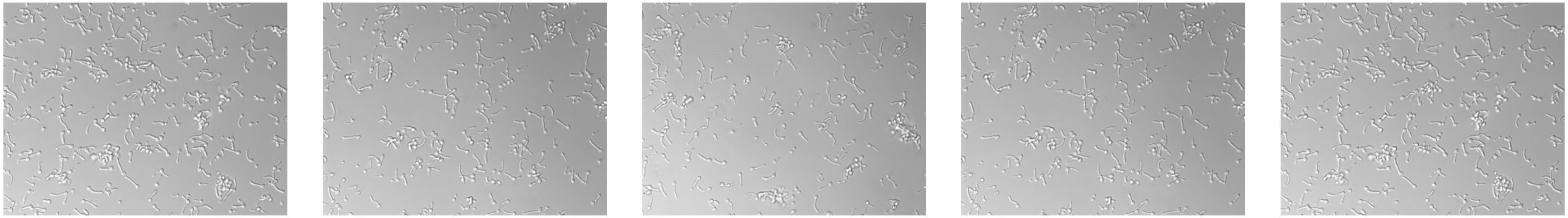

P75010

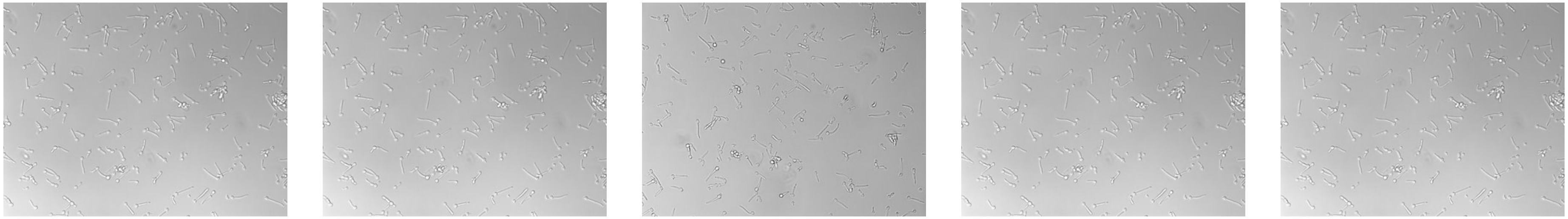

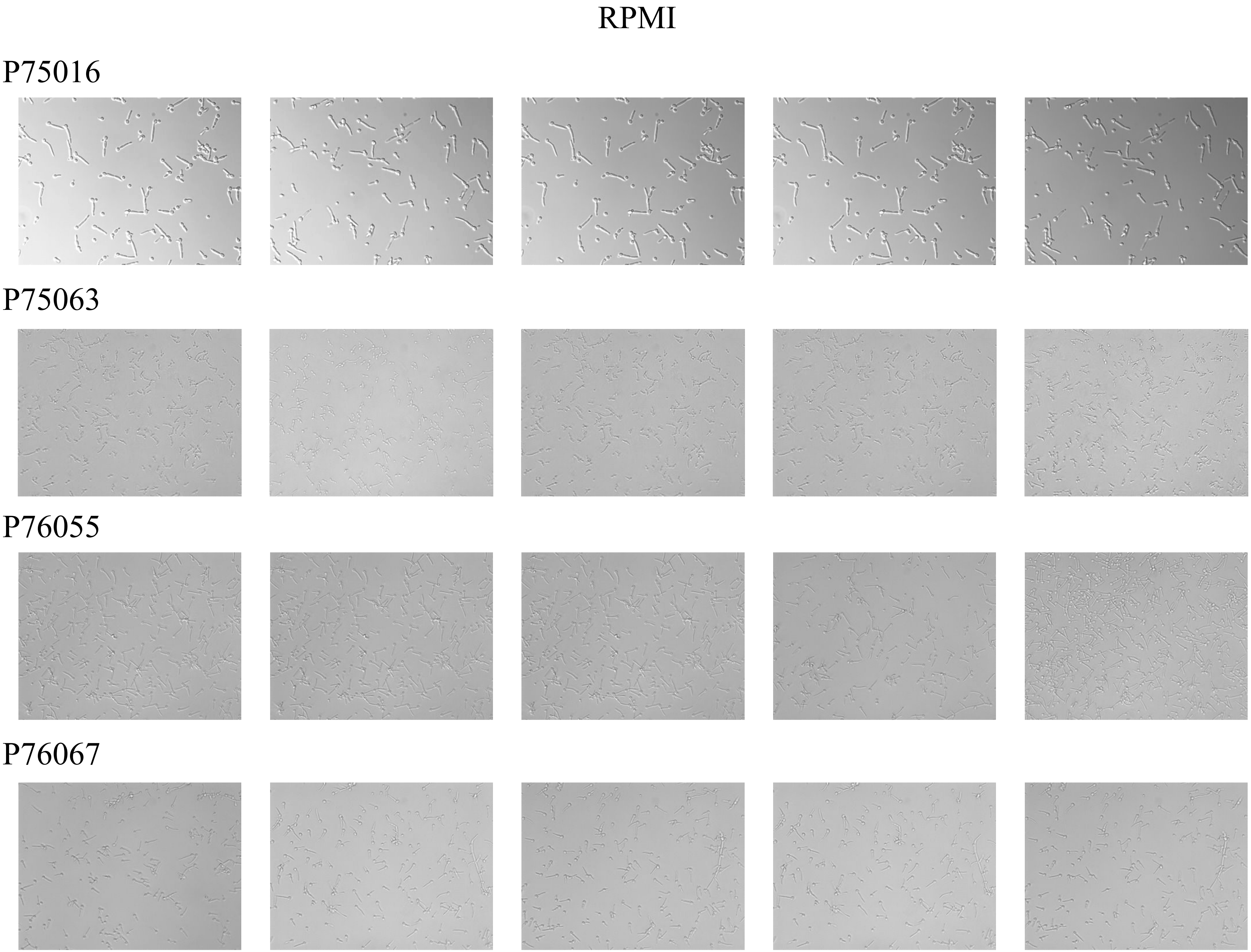
RPMI
P75016
P75063
P76055
P76067

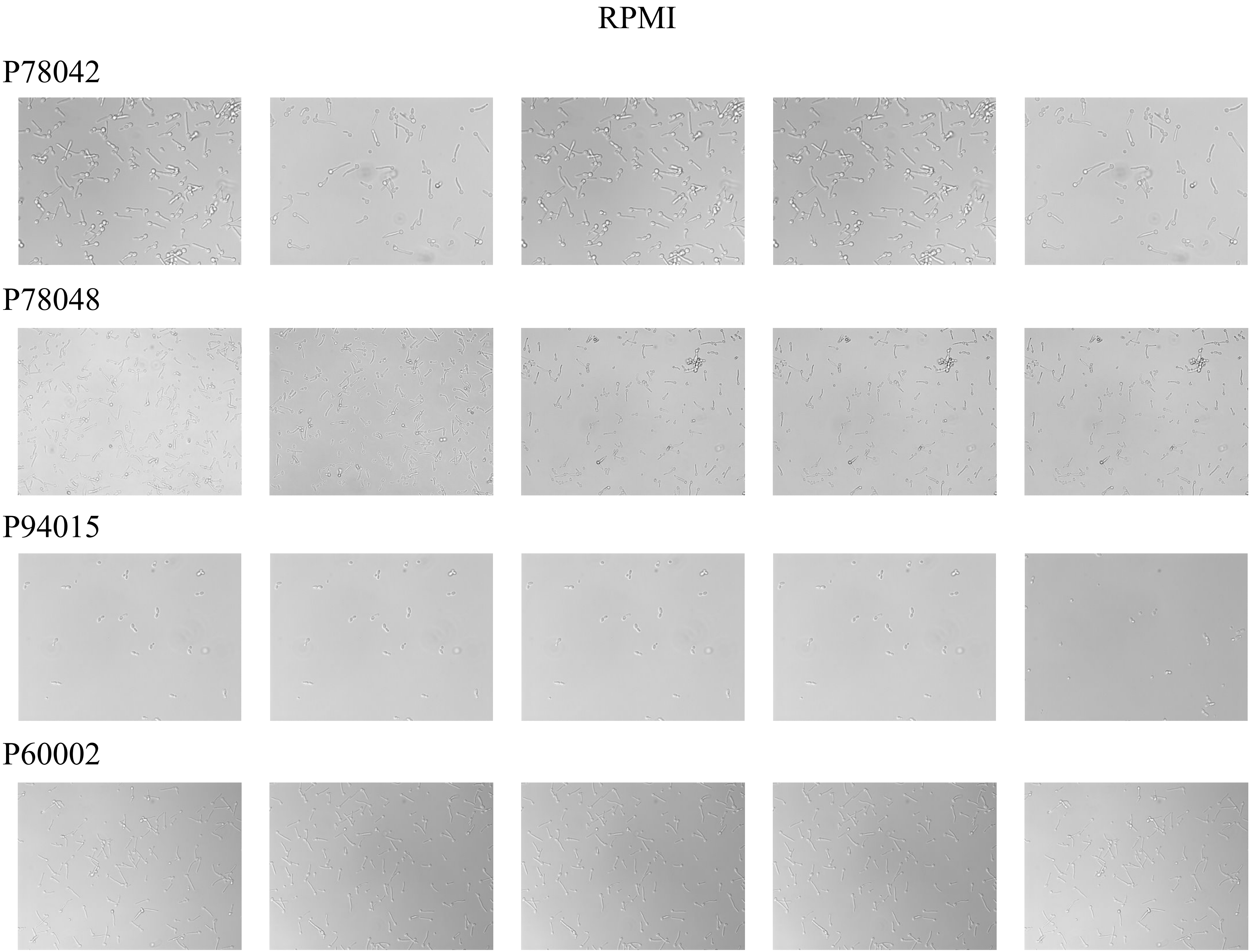
RPMI
P78042
P78048
P94015
P60002

Spider

B444-12

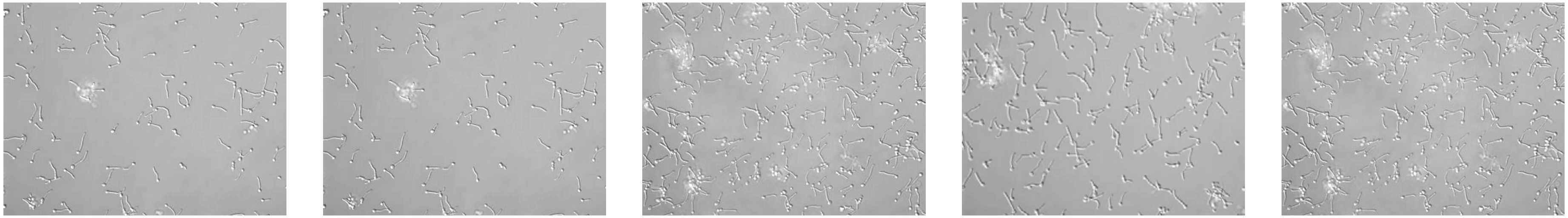

B1257-15

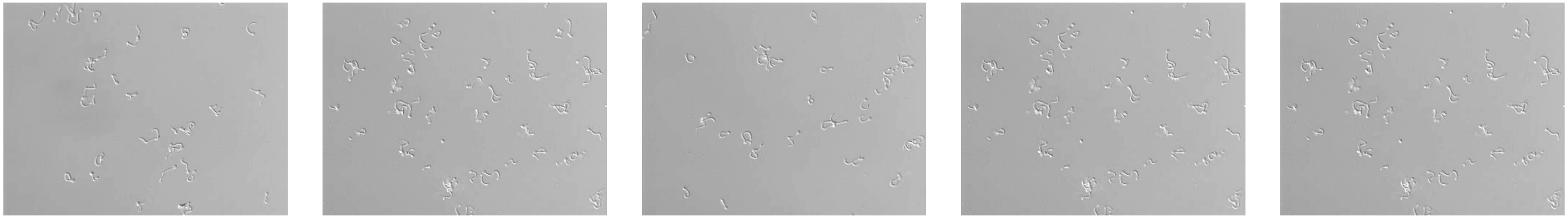

B687-15

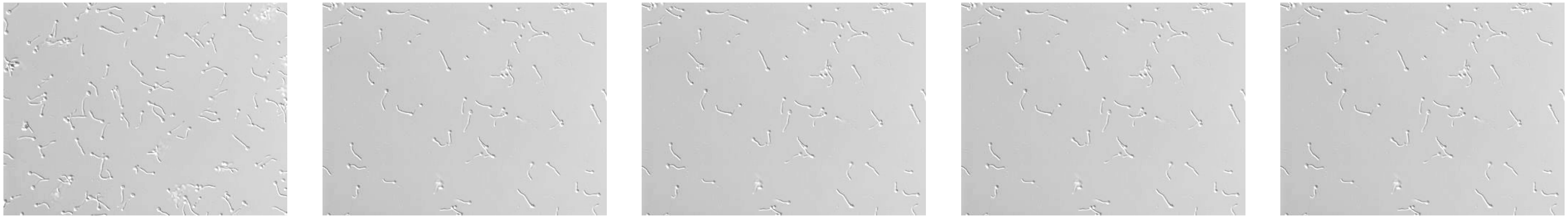

B1762-15

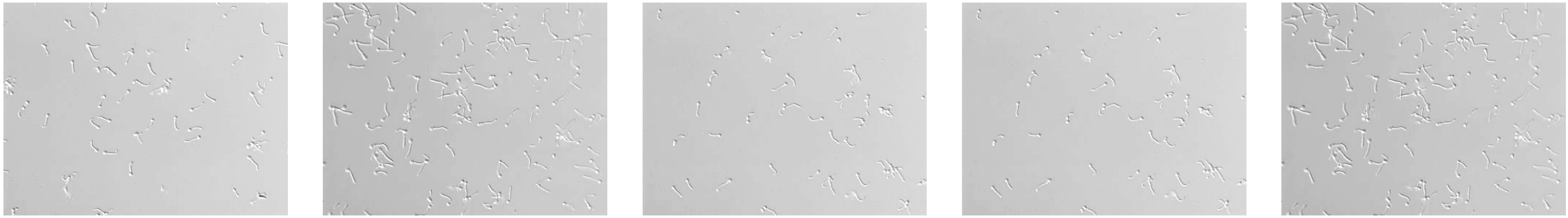

Spider

B46-15

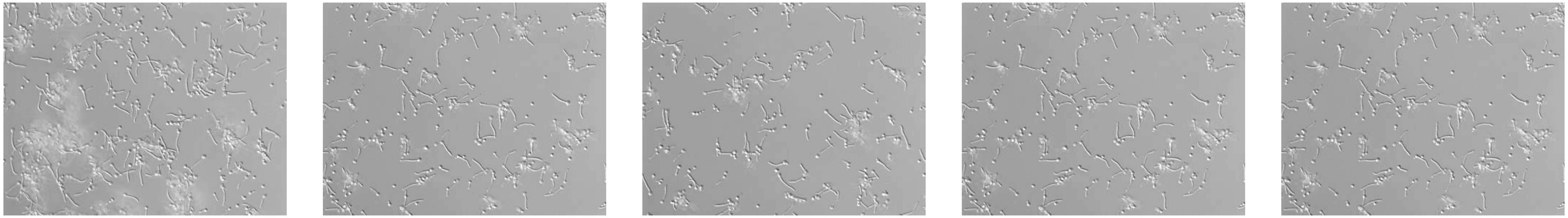

B808-15

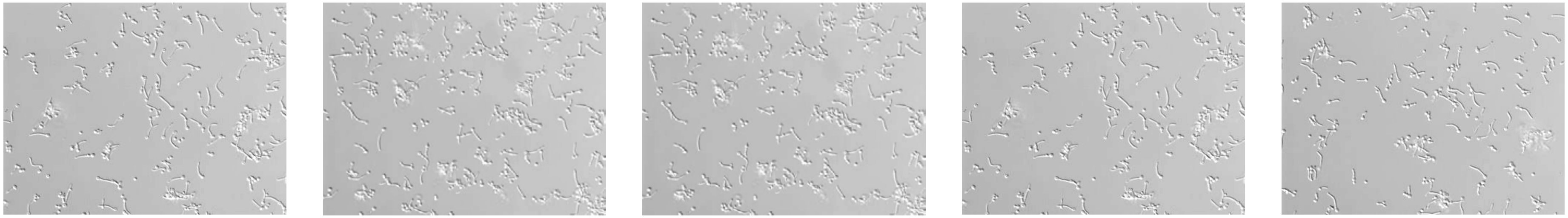

B527-15

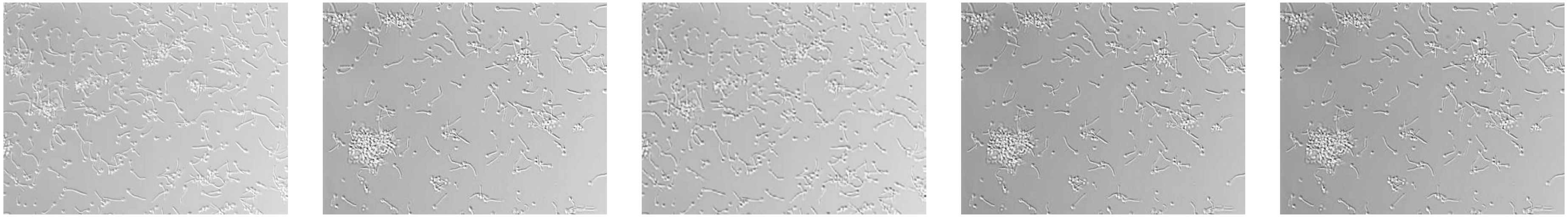

B618-15

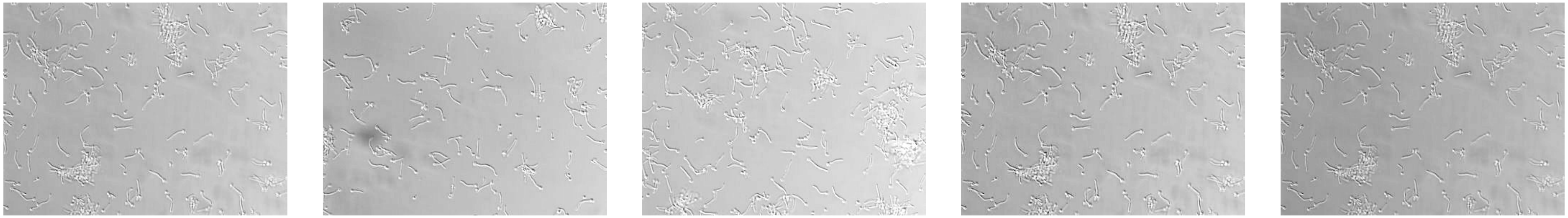

Spider

B404-15

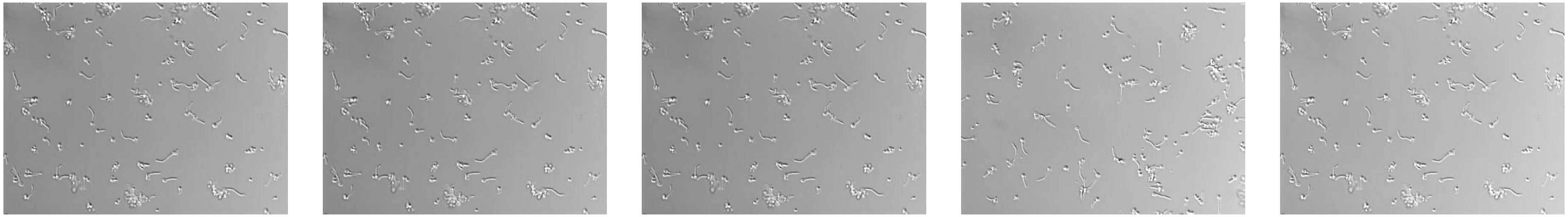

B421-15

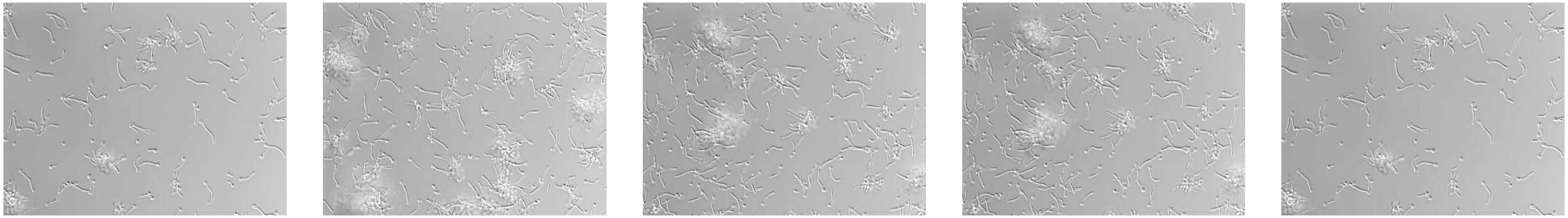

B212-12

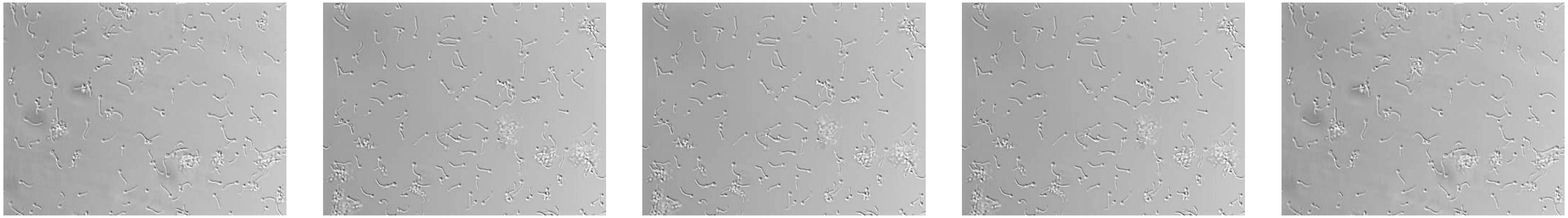

B1091-15

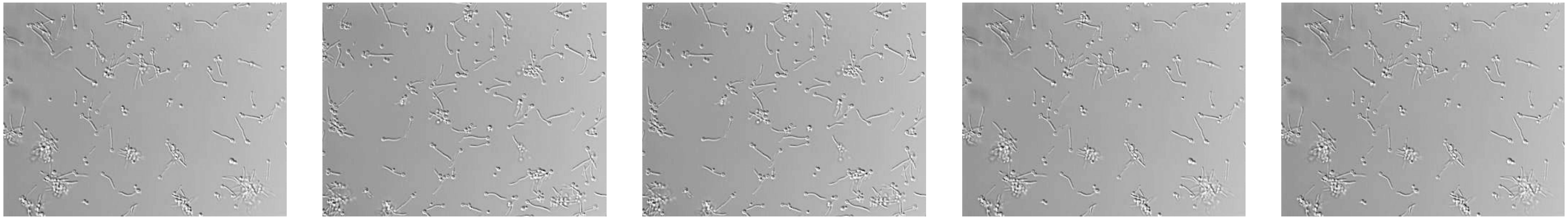

Spider

B510-12

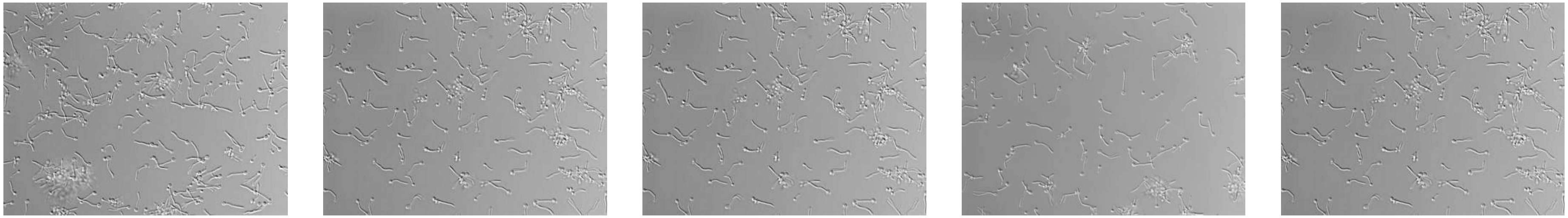

B564-15

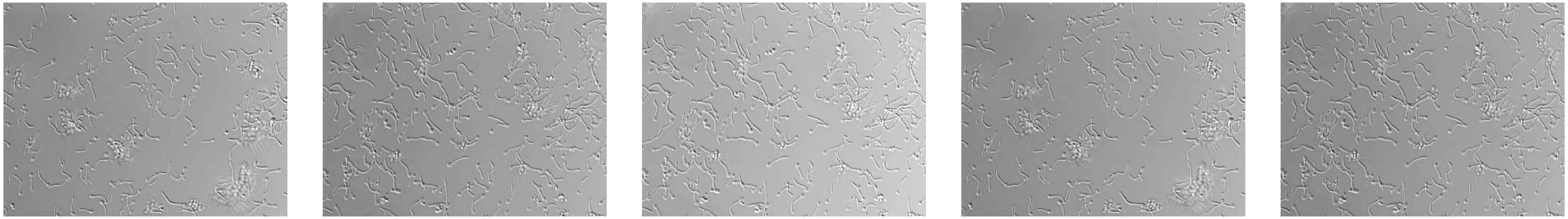

B1168-15

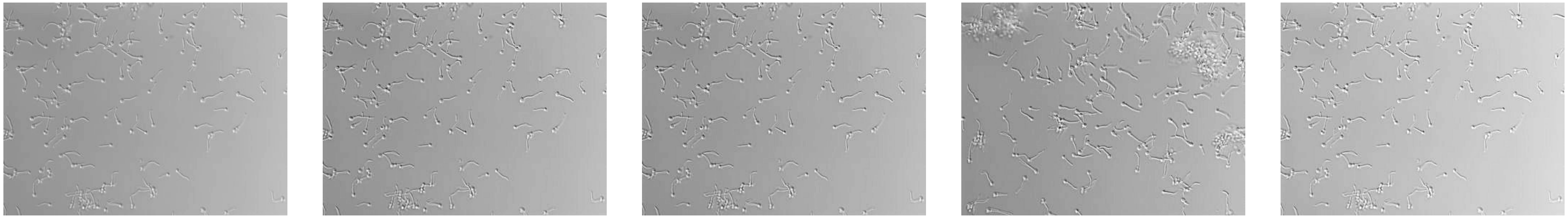

B568-15

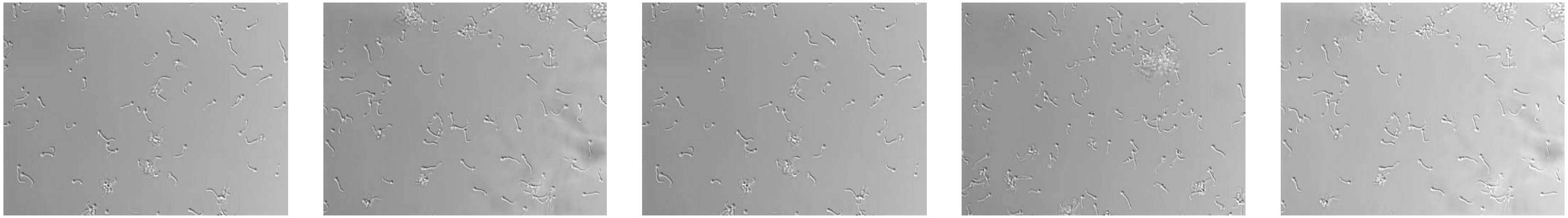

Spider

B2527-12

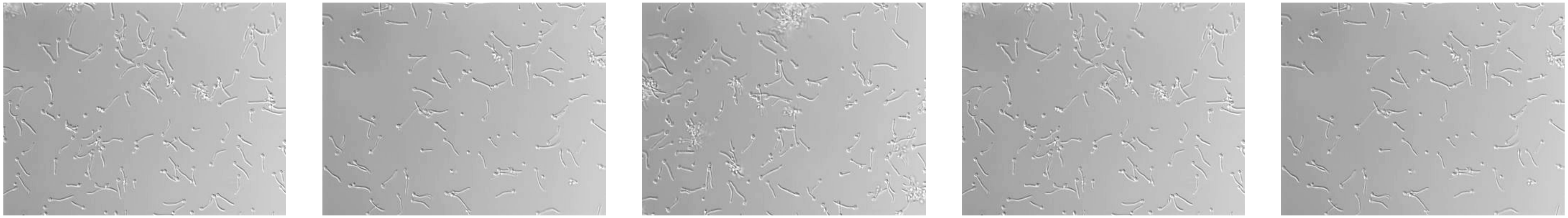

B1486-15

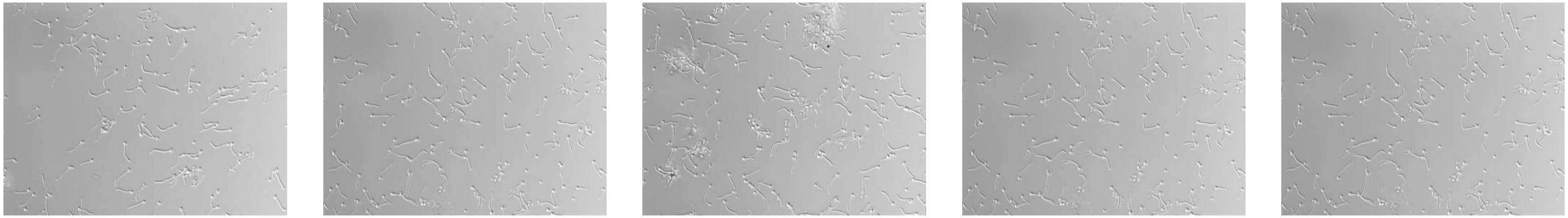

B1559-15

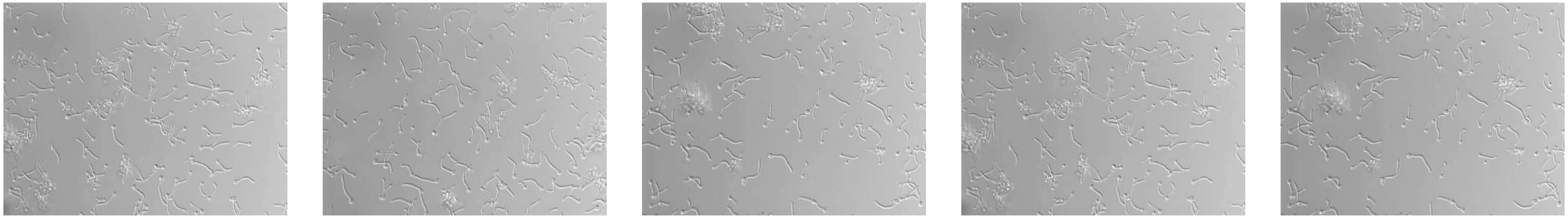

B733-15

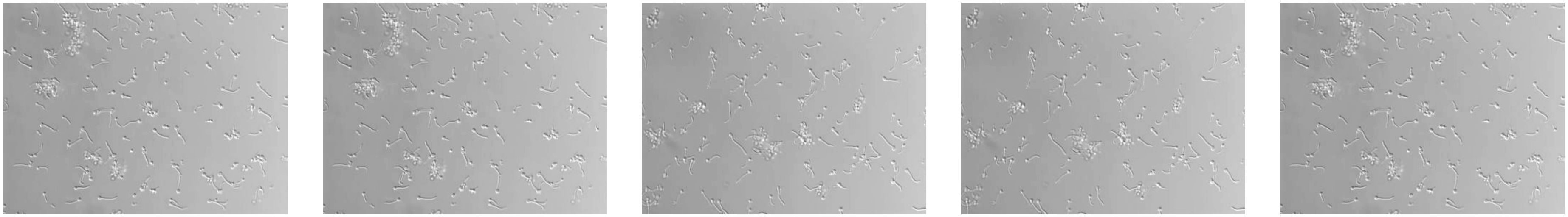

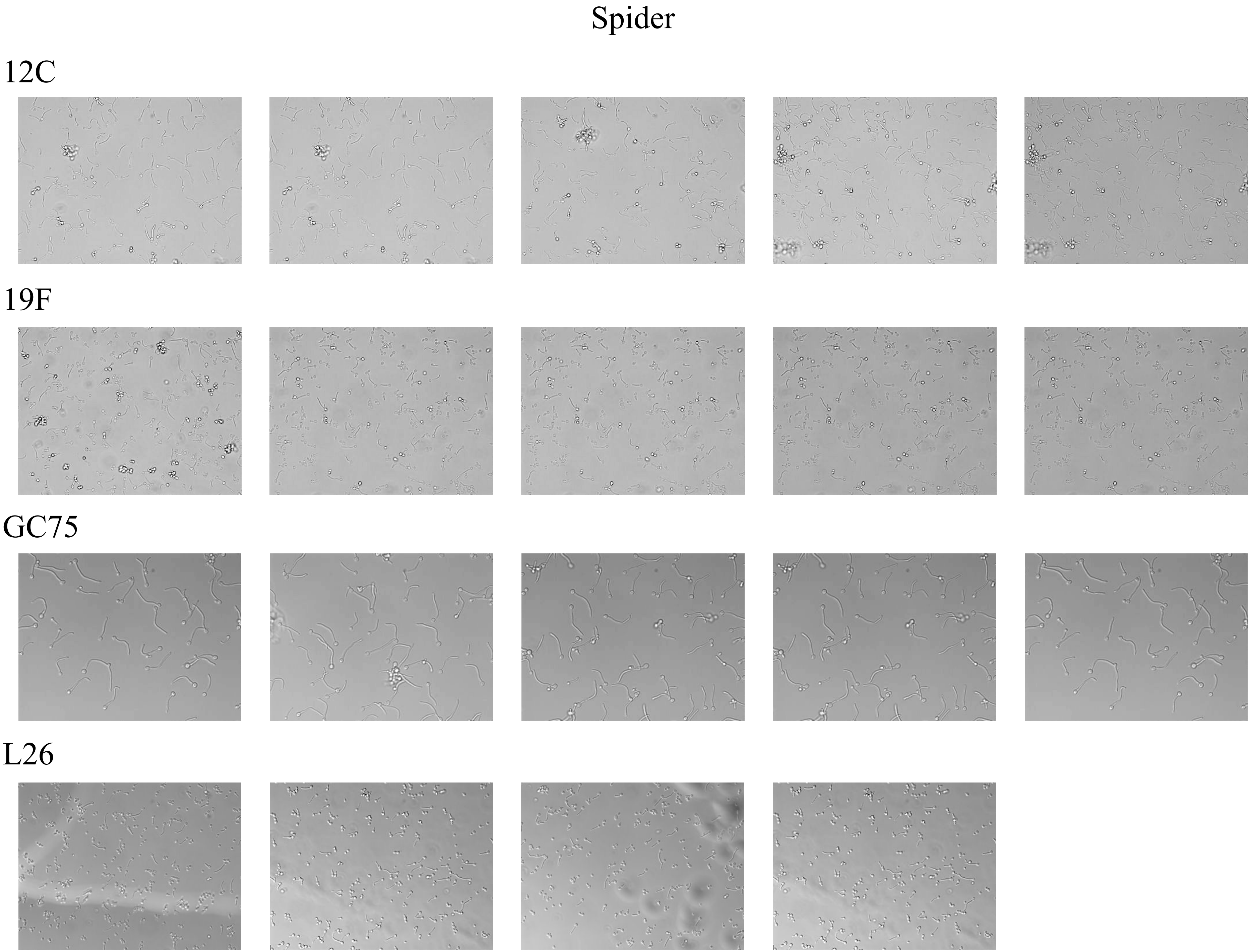
Spider
12C
19F
GC75
L26

Spider

P87

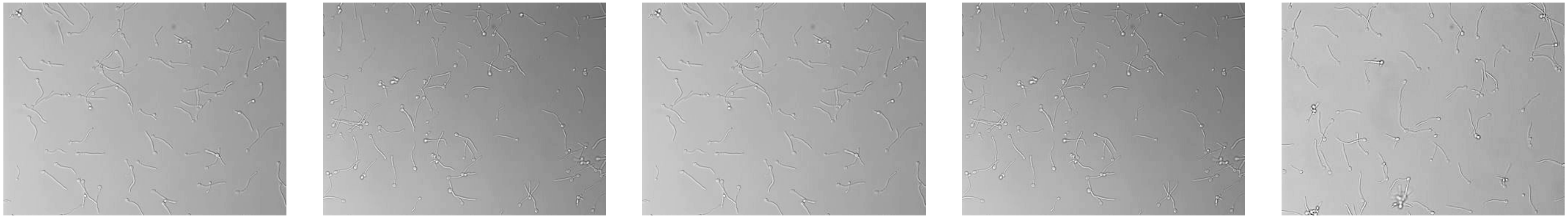

P34048

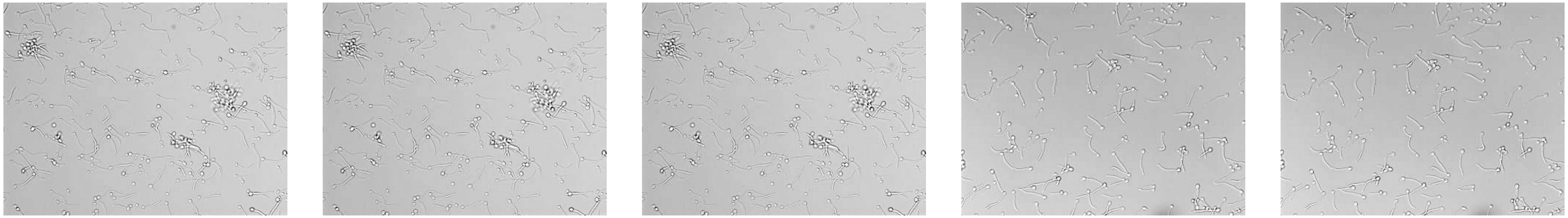

P37005

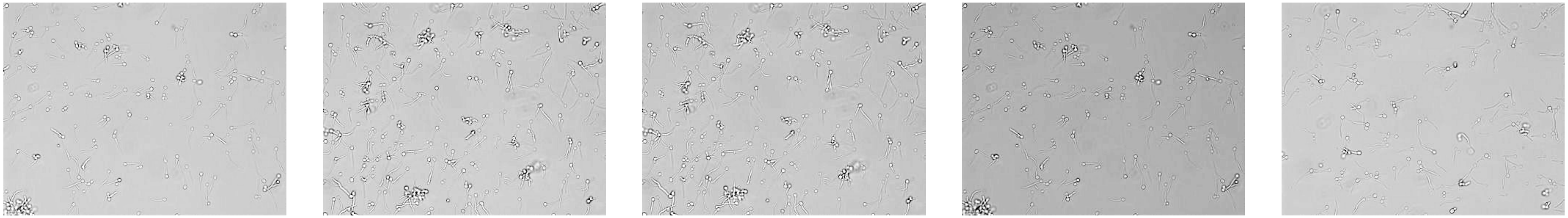

P37037

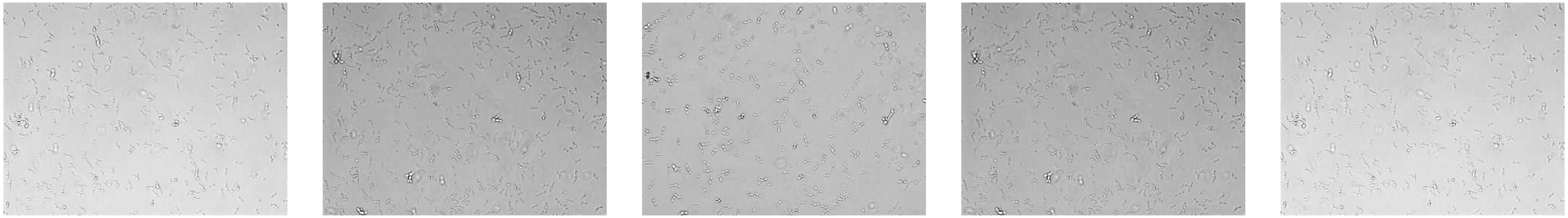

Spider

P37039

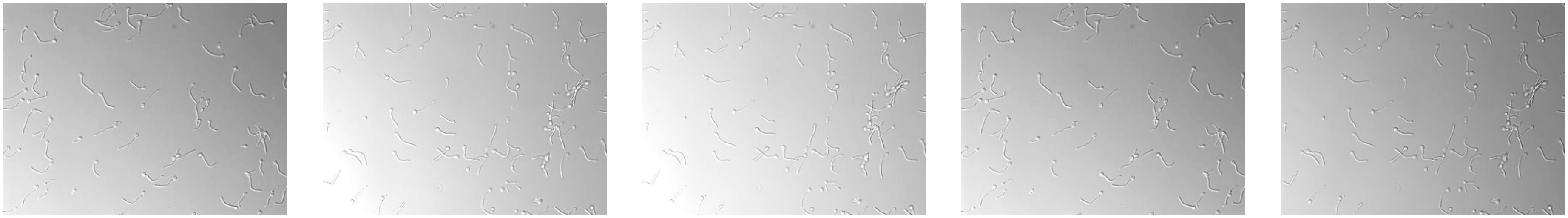

P57055

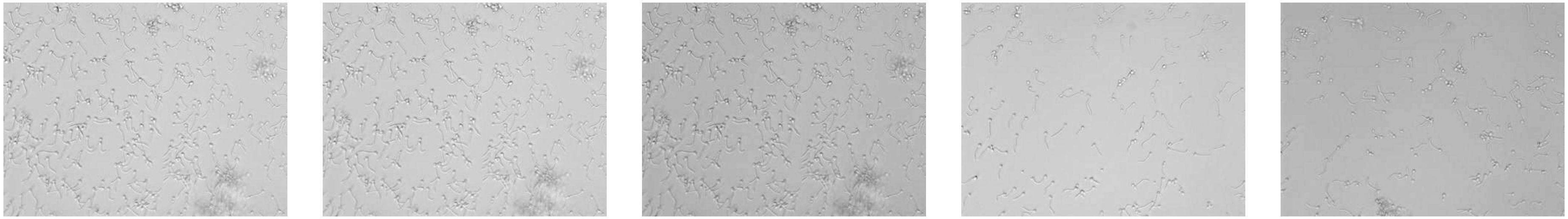

P57072

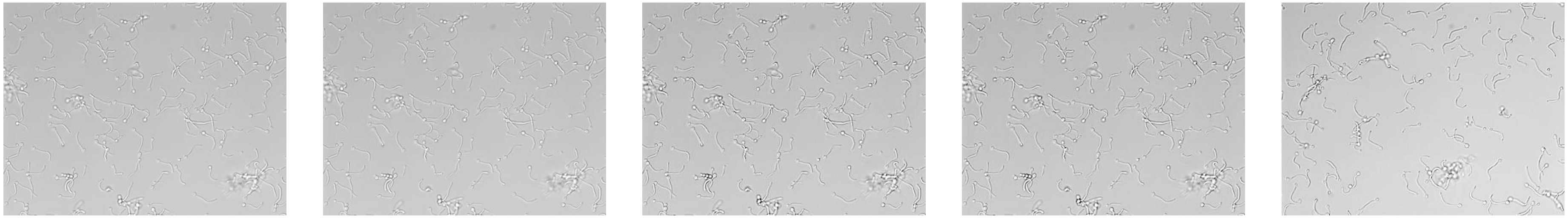

P75010

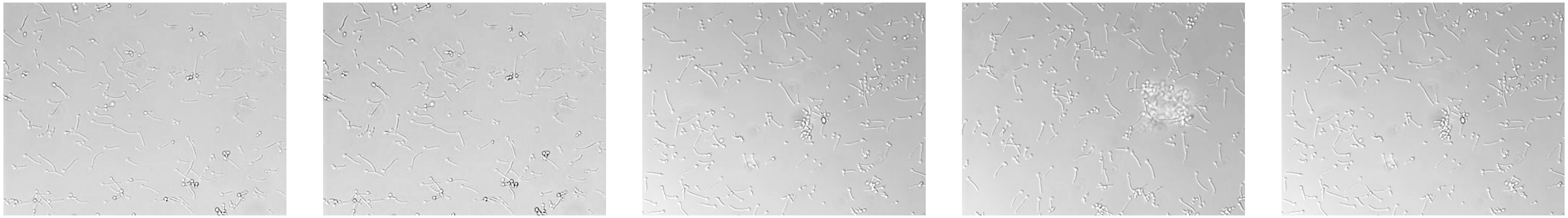

Spider

P75016

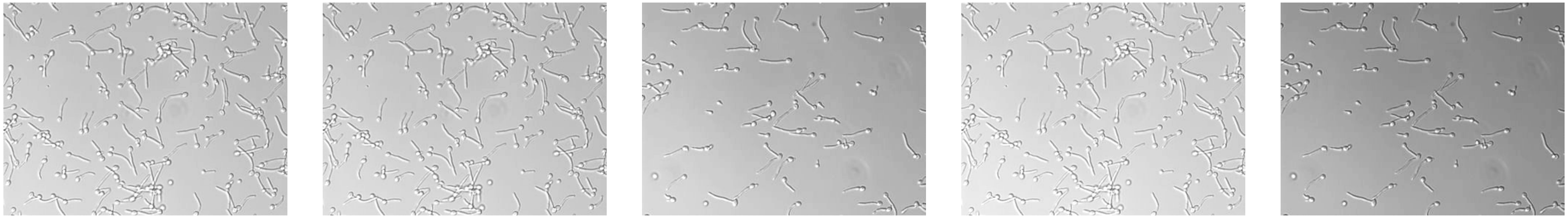

P75063

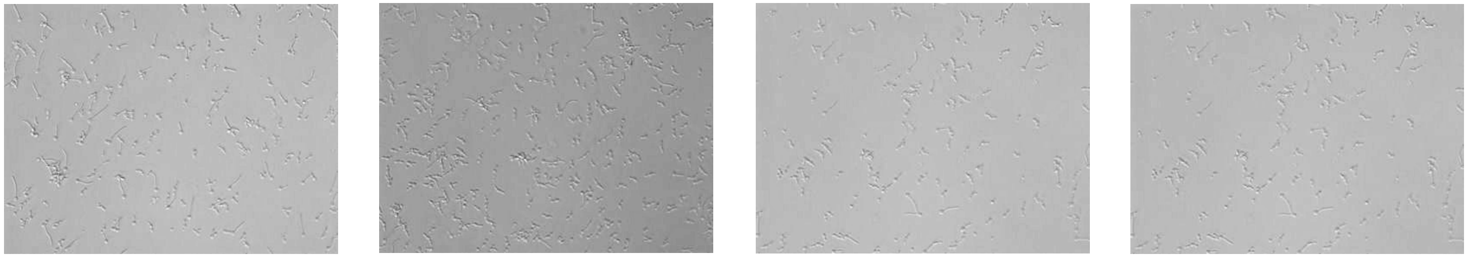

P76055

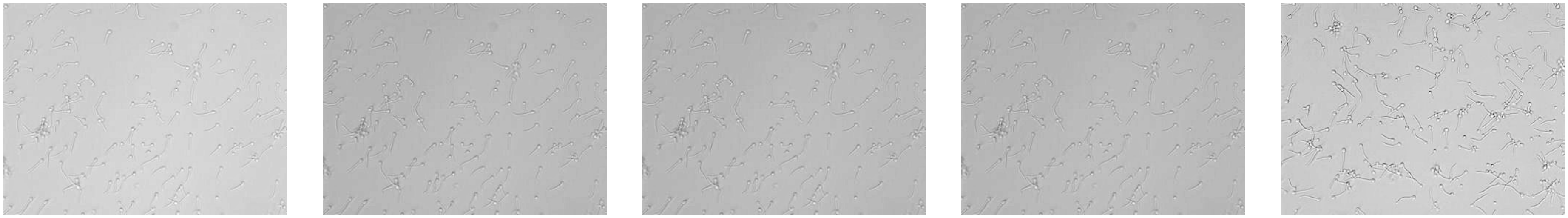

P76067

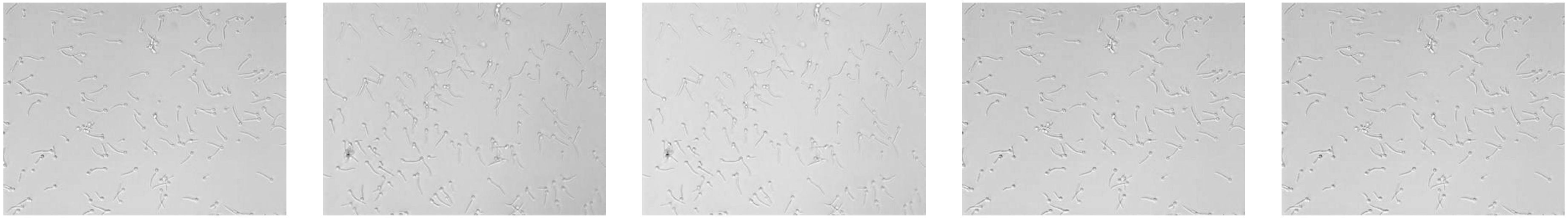

Spider

P78042

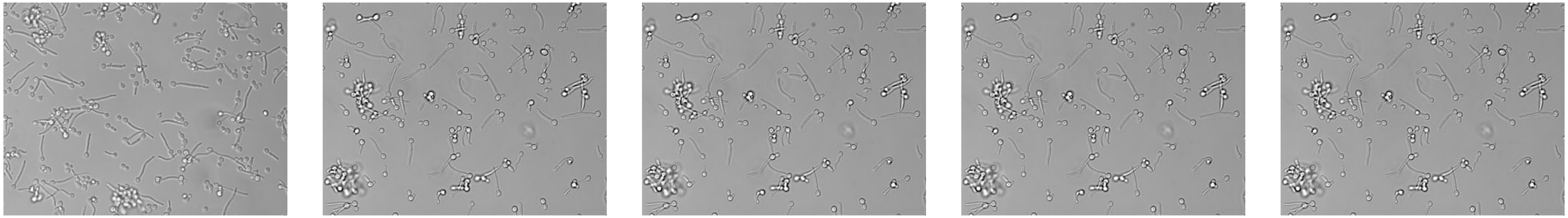

P78048

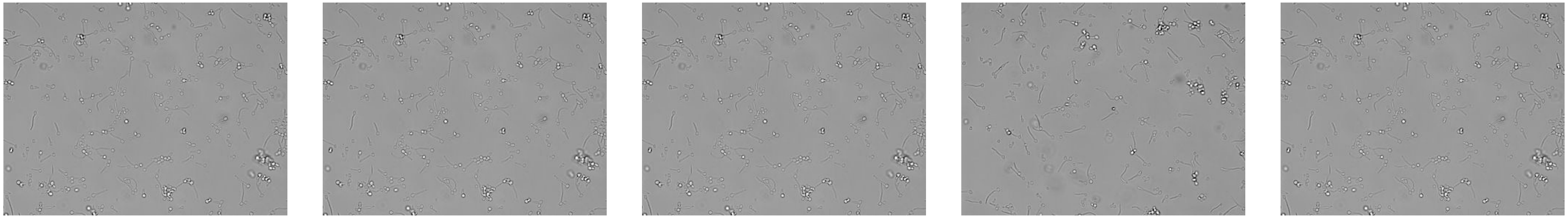

P94015

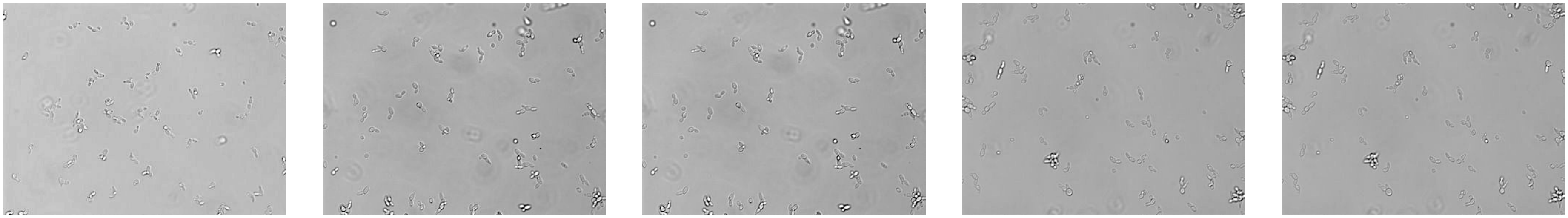

P60002

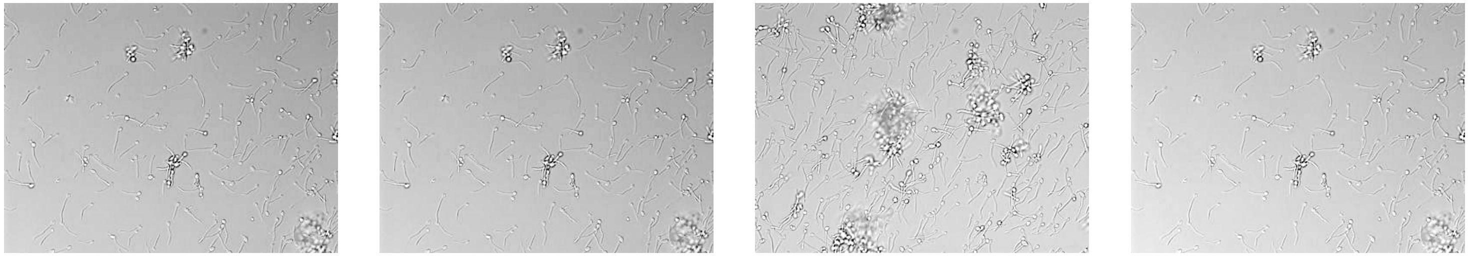

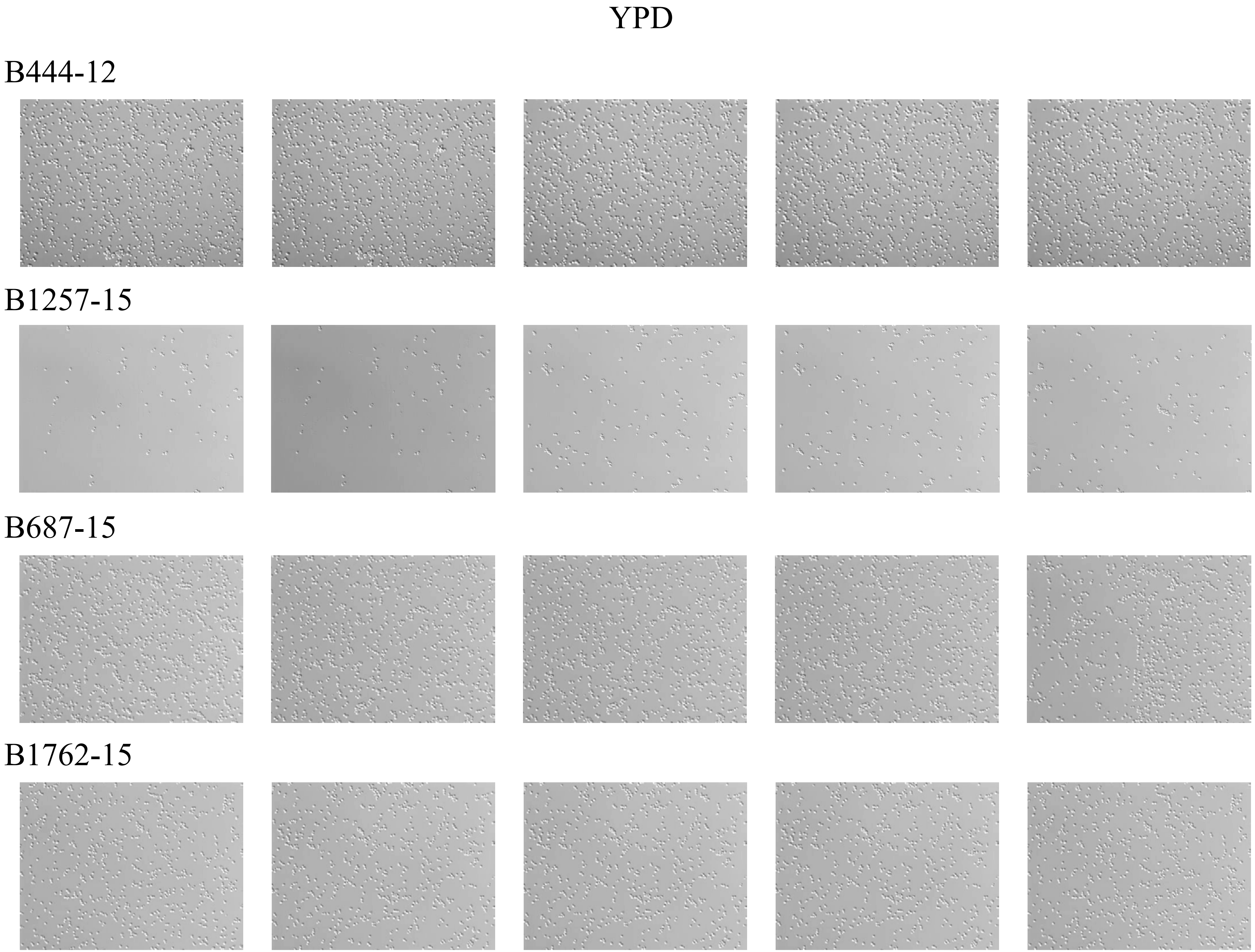
YPD
B444-12
B1257-15
B687-15
B1762-15

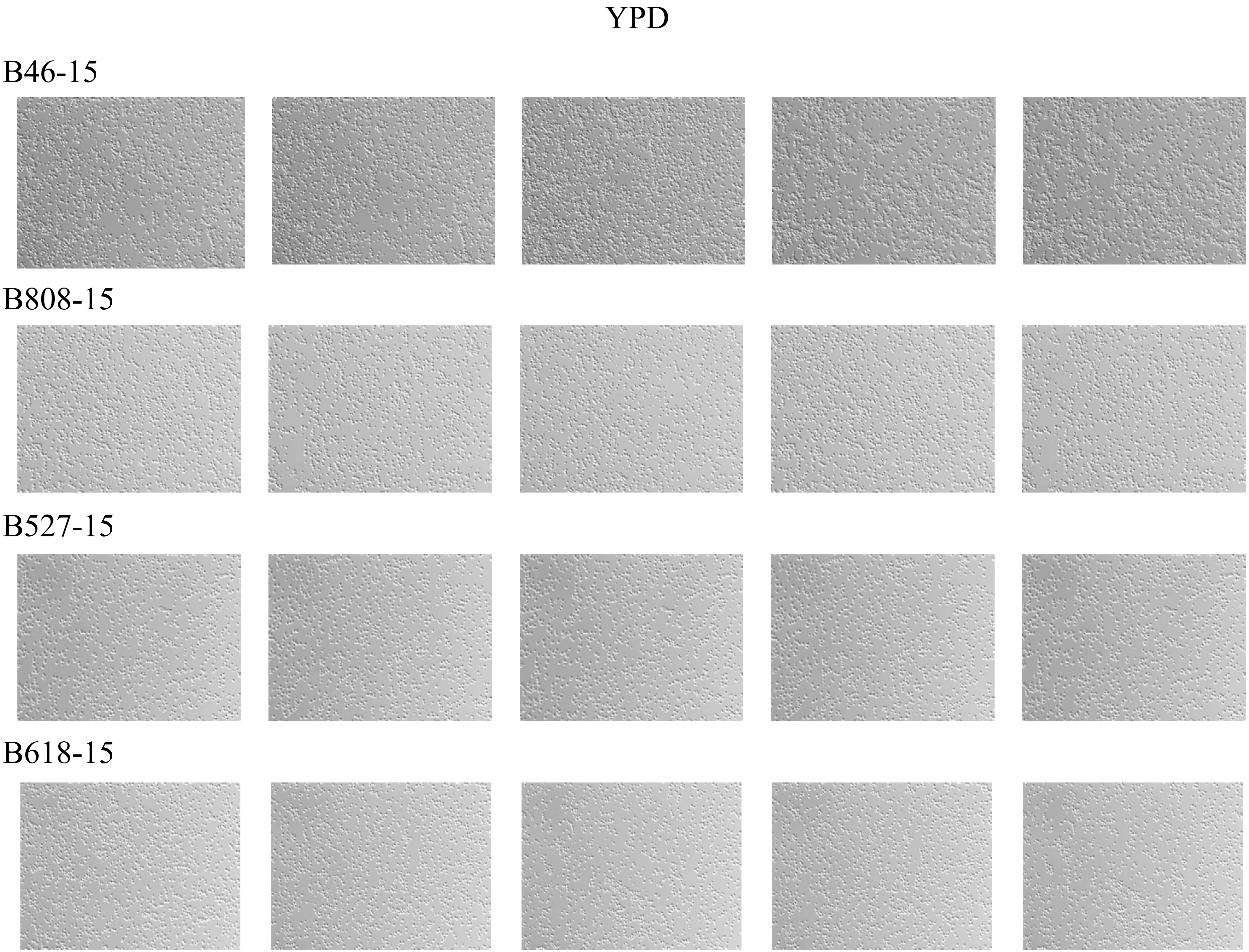
YPD
B46-15
B808-15
B527-15
B618-15

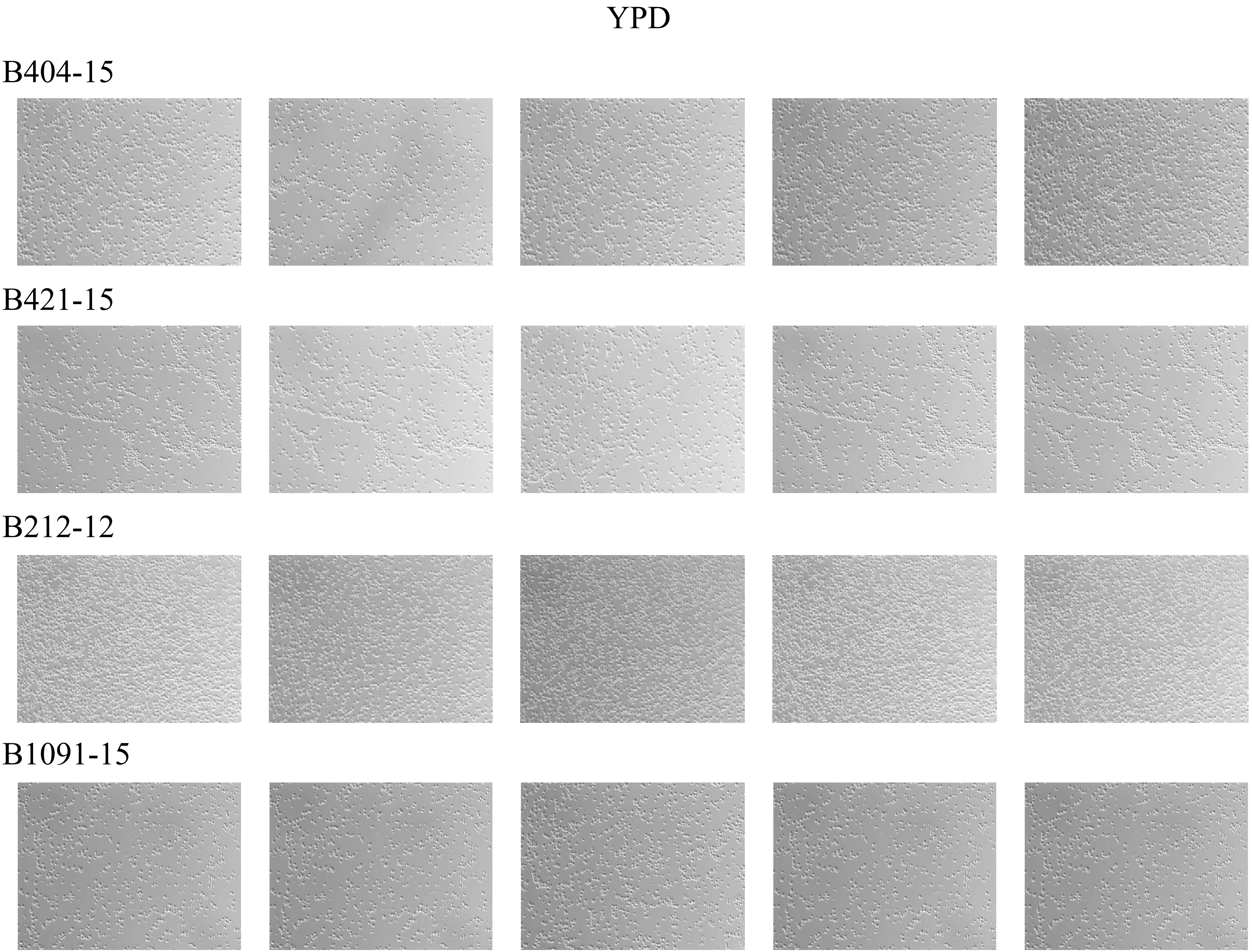
YPD
B404-15
B421-15
B212-12
B1091-15

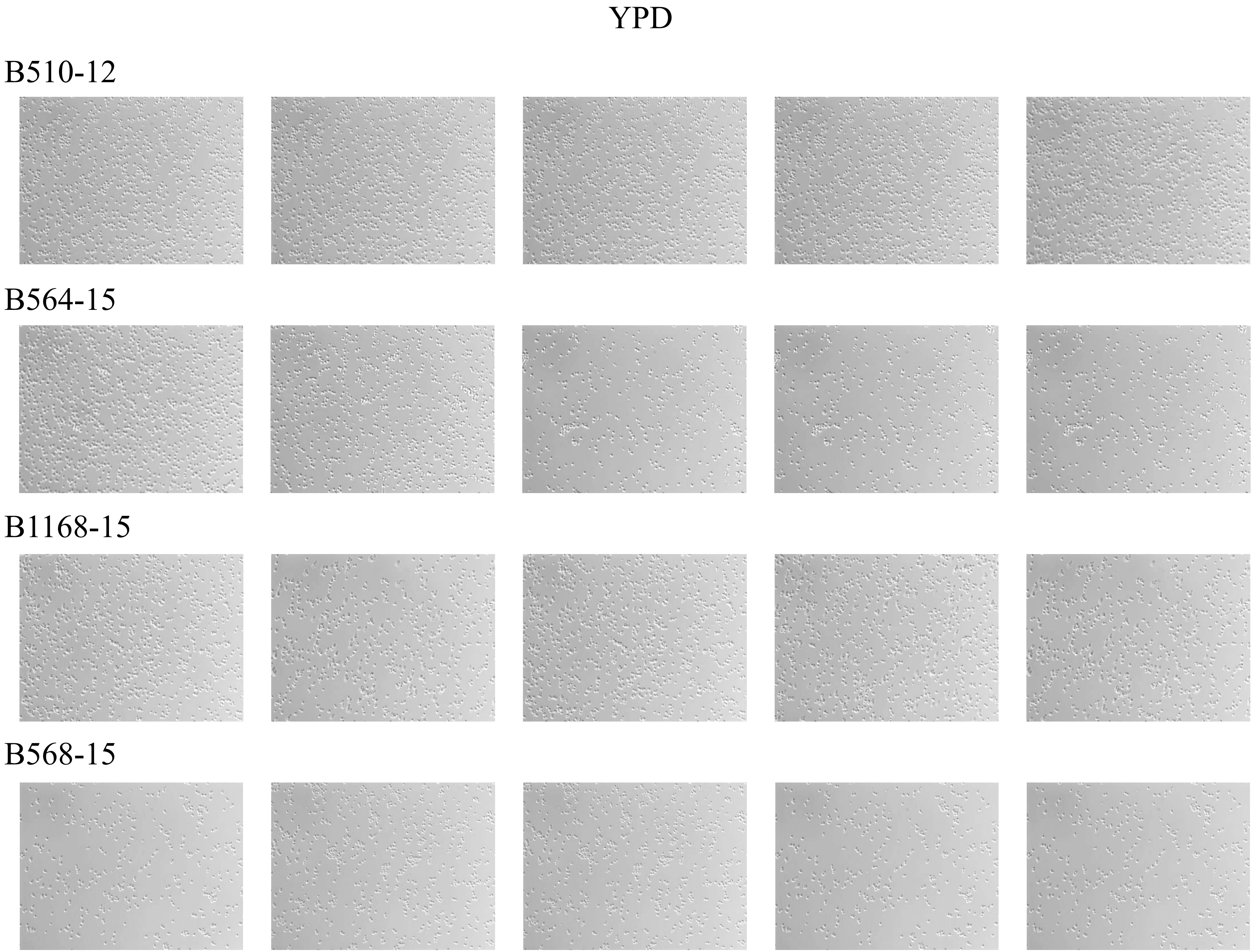
YPD
B510-12
B564-15
B1168-15
B568-15

YPD

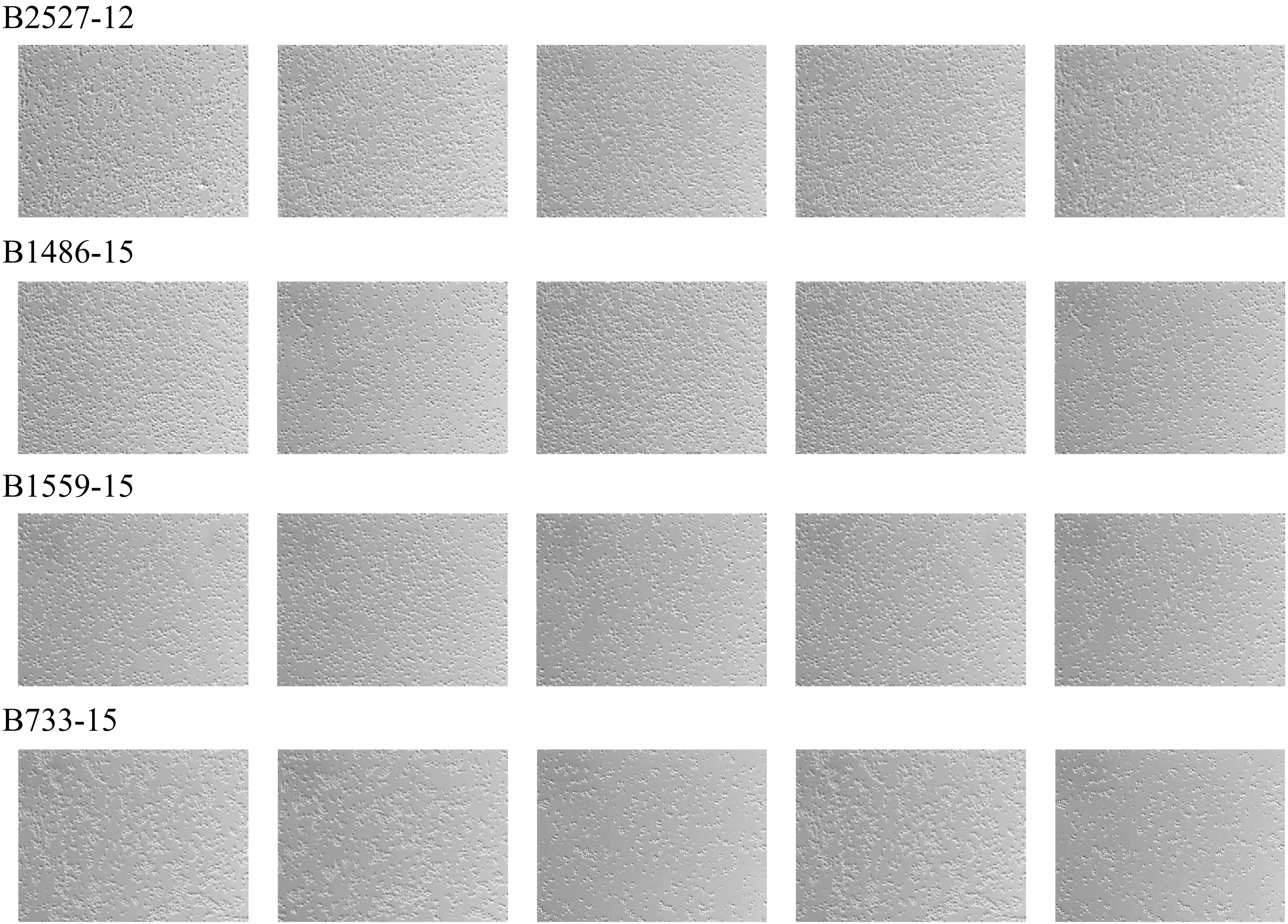

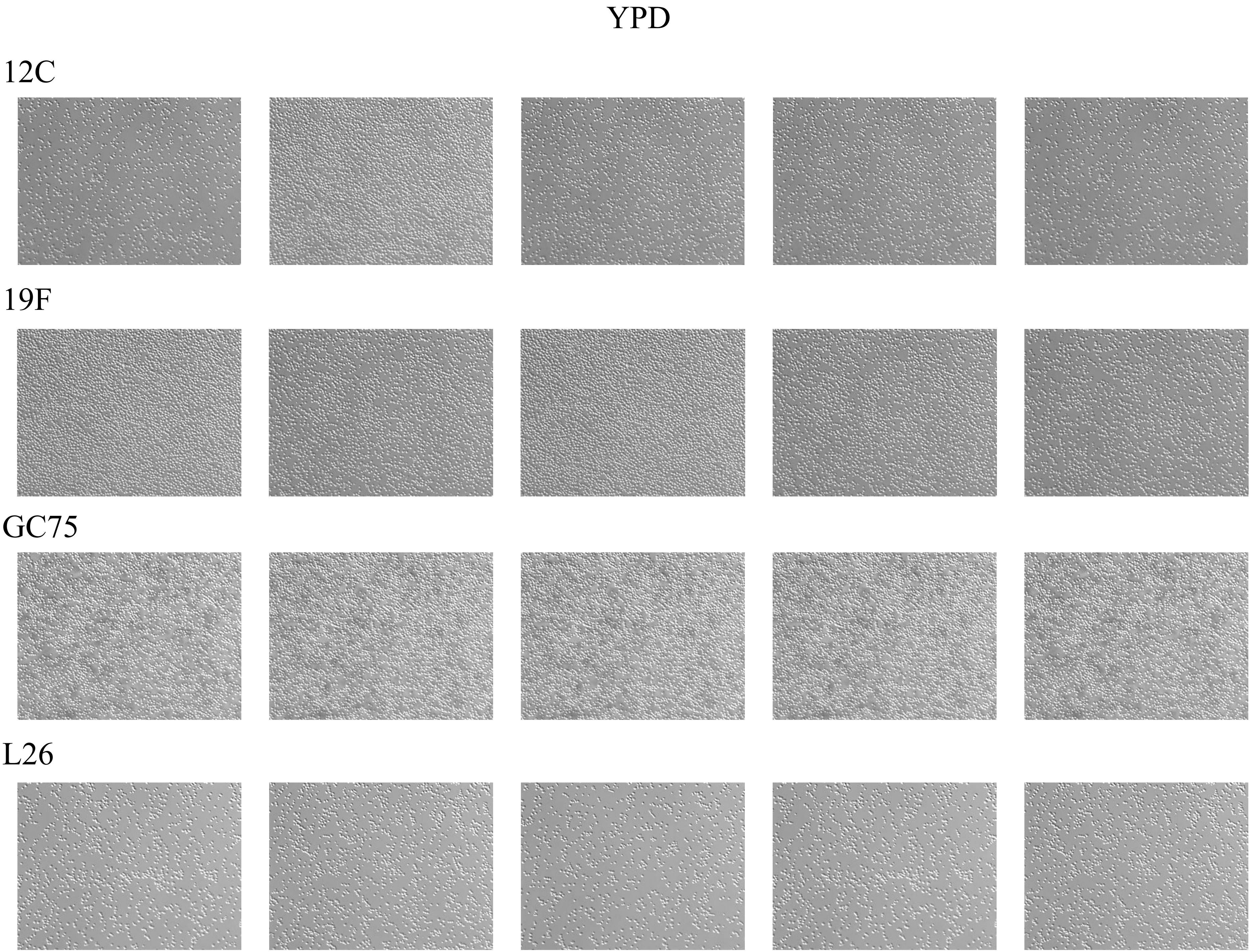
YPD
12C
19F
GC75
L26

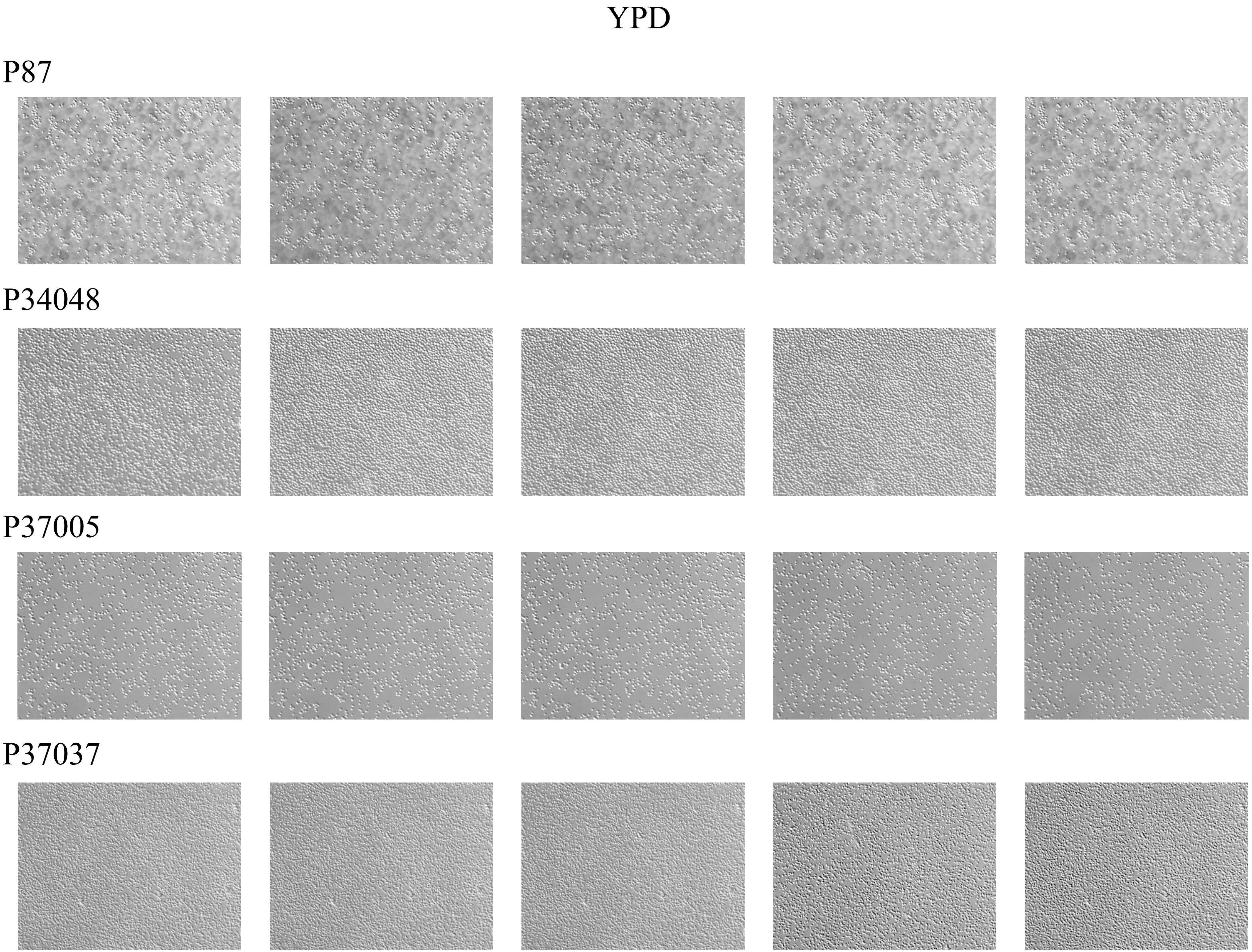
YPD
P87
P34048
P37005
P37037

YPD

P37039

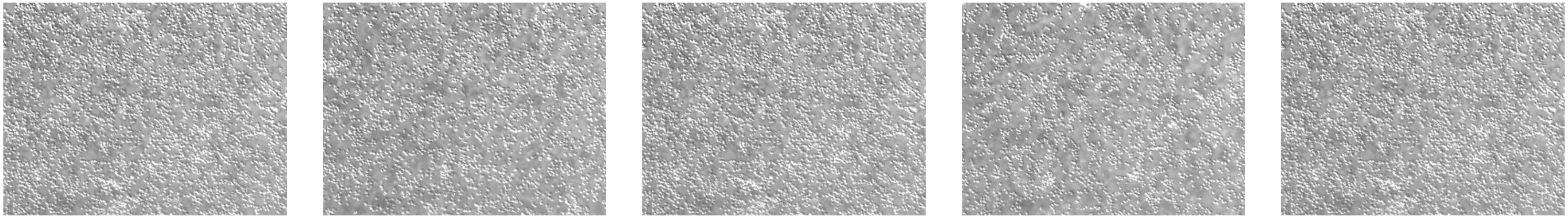

P57055

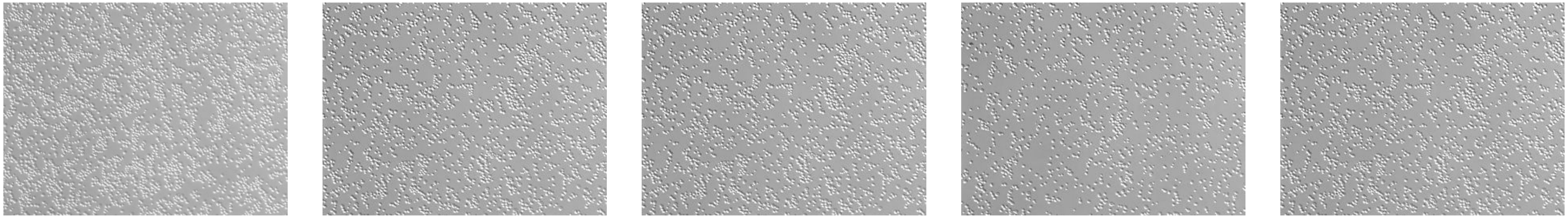

P57072

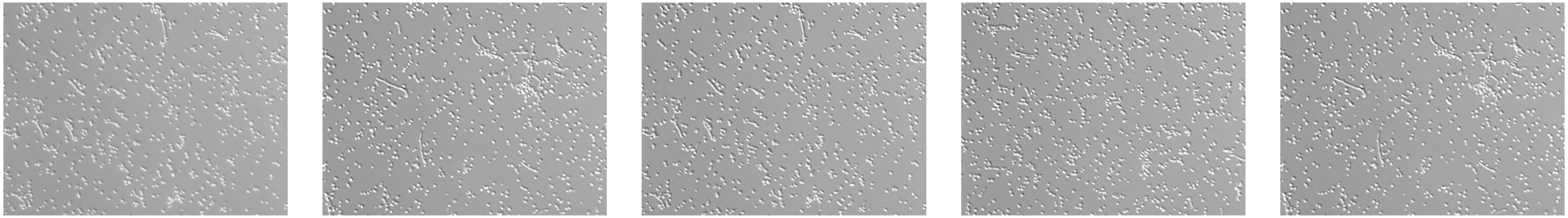

P75010

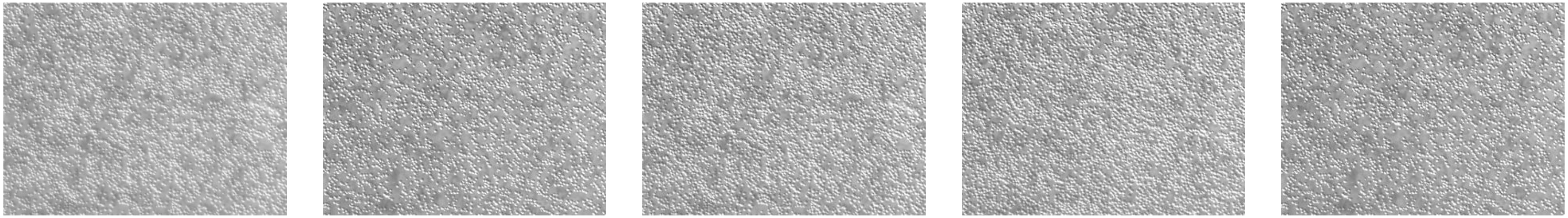

YPD

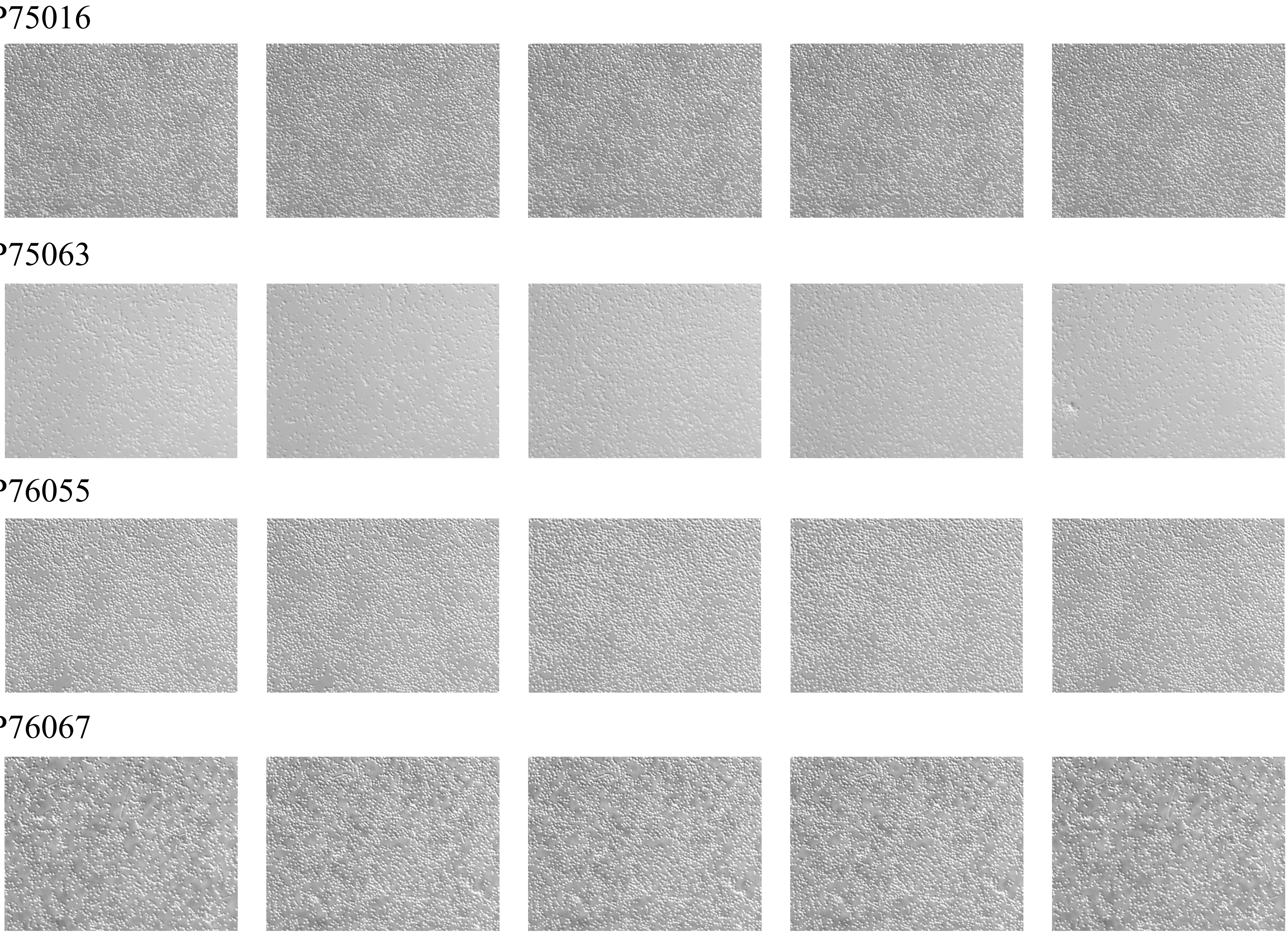

YPD

P78042

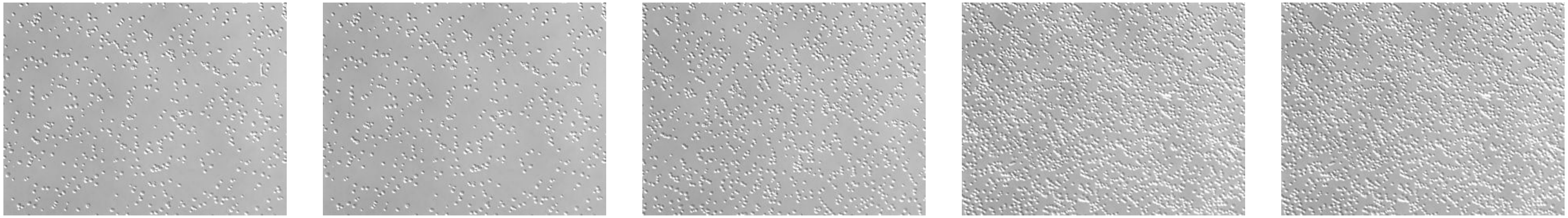

P78048

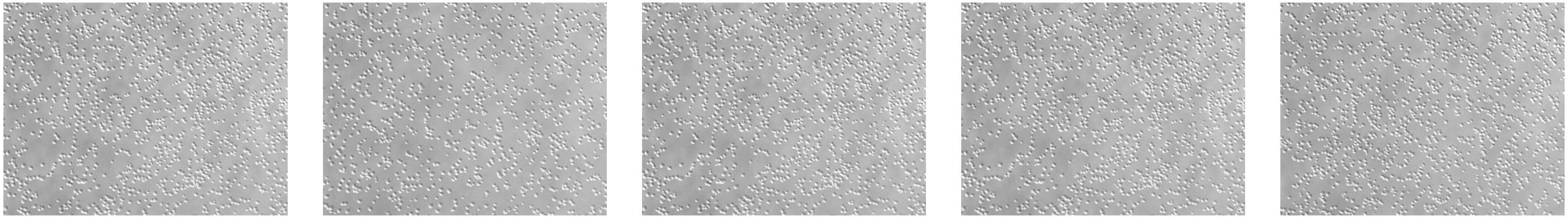

P94015

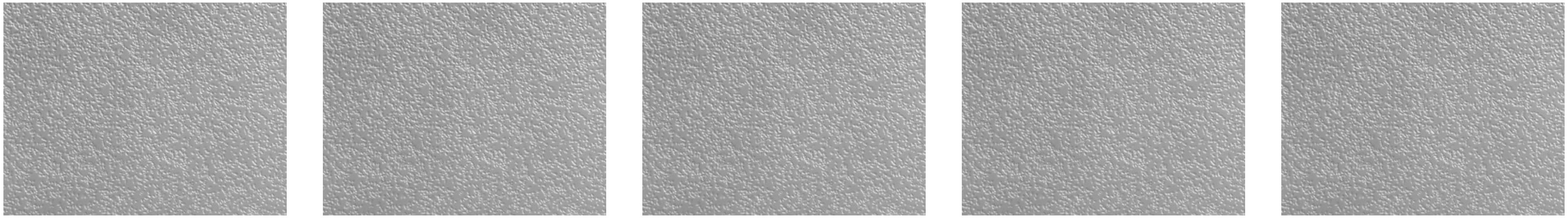

P60002

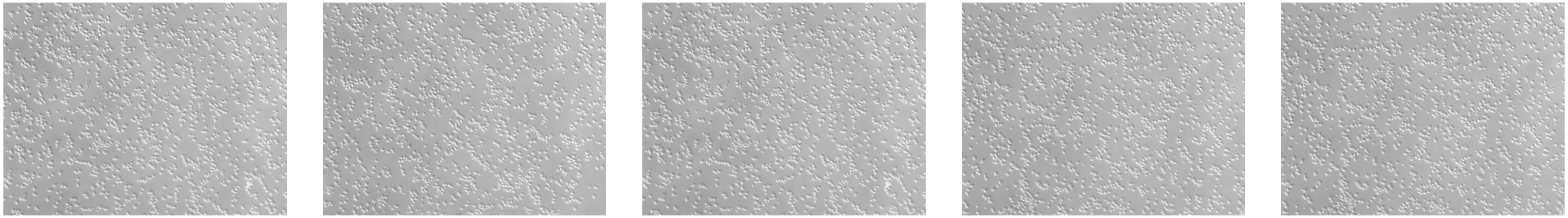

Supplement: Supplementary Figure 1 — Clinical strain filamentation in the standard liquid media assay. Cells of the indicated strains were tested for filamentation in standard and shortened solid filamentation assays in FBS, Lee’s, RPMI, or spider liquid media. Cells were grown overnight at 30˚C with shaking, washed, and then incubated in the inducing liquid media for 3 hours at 37˚C. Also shown are images from the overnight cells grown in YPD. The images shown are a representative example of images assessed bioinformatically due to file size limitations. [file DataSheet_3.pdf]
