# Supplementary material for: Significant variation of filamentation phenotypes in clinical Candida albicans strains
Source: Front Cell Infect Microbiol. 2023 Oct 20;13:1207083. doi: 10.3389/fcimb.2023.1207083 (PMC10623444; doi:10.3389/fcimb.2023.1207083)

Figure S2. Solid filamentation assays FBS

B444-12

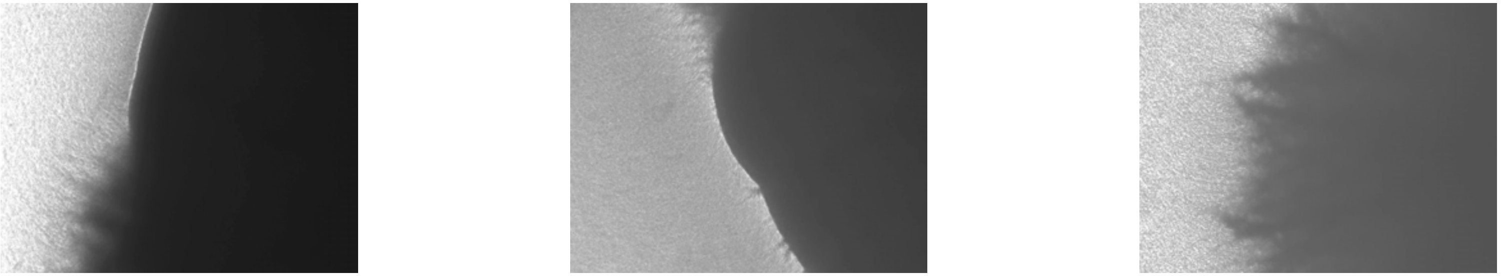

B1257-15

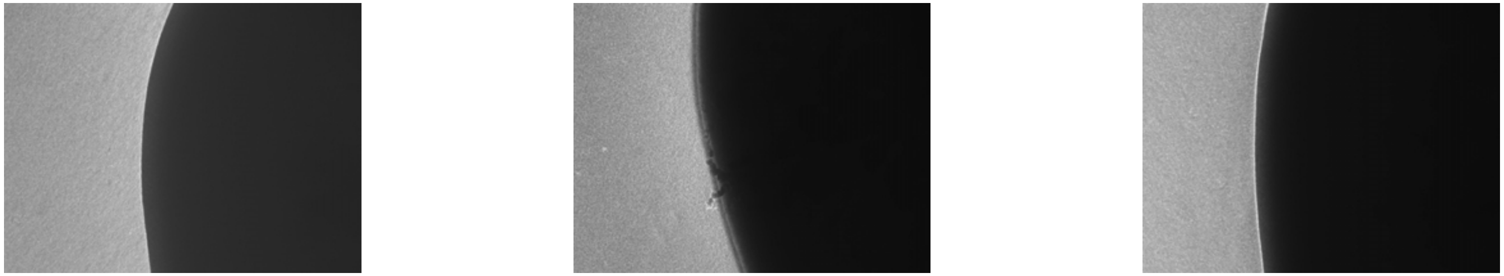

B687-15

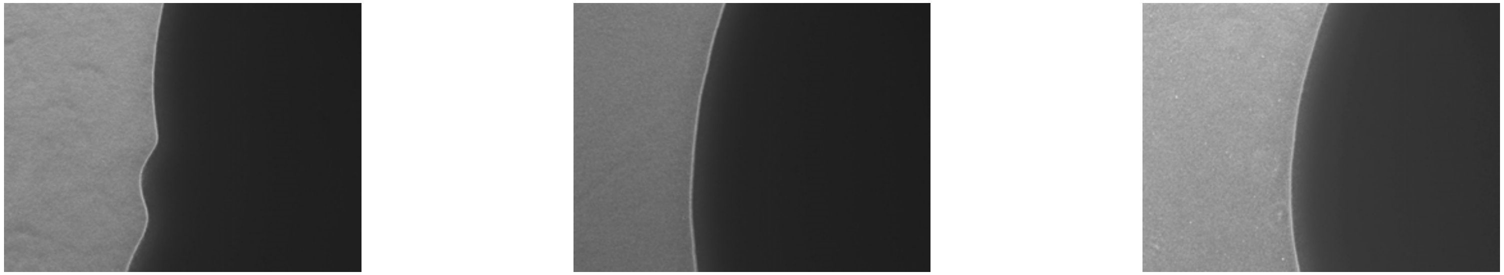

B1762-15

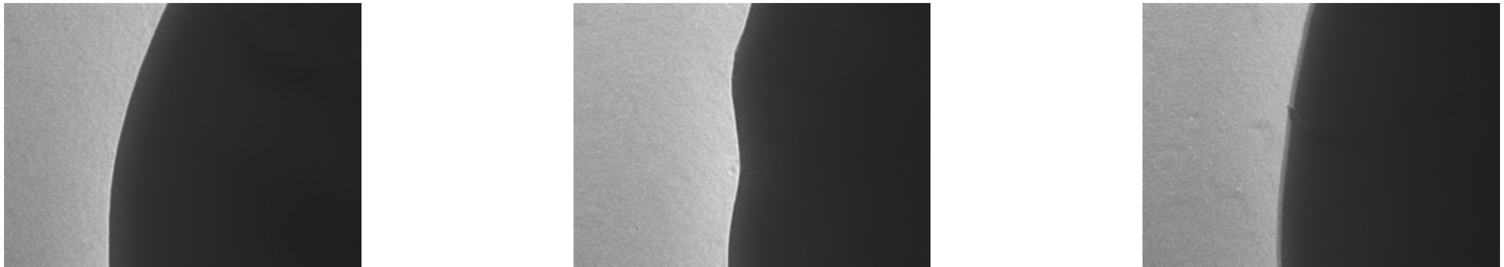

FBS

B46-15

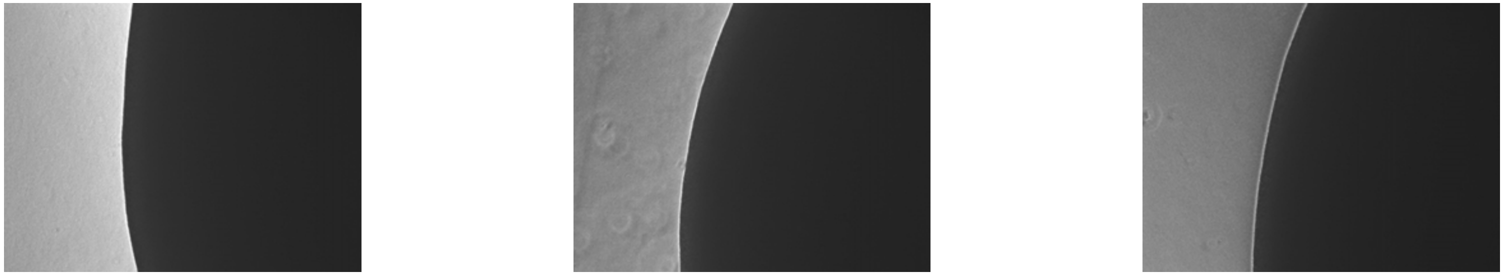

B808-15

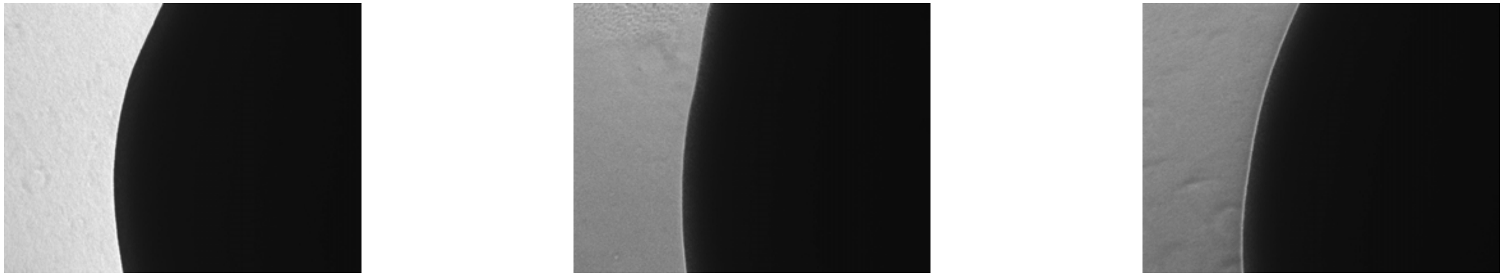

B527-15

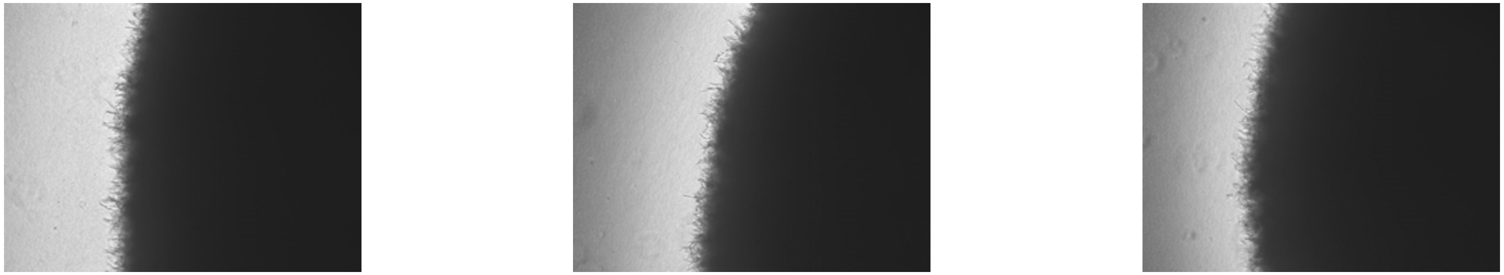

B618-15

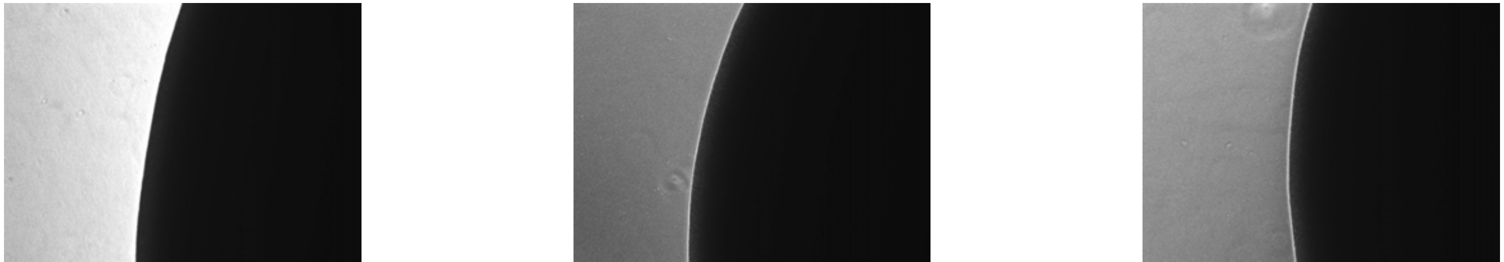

FBS

B404-15

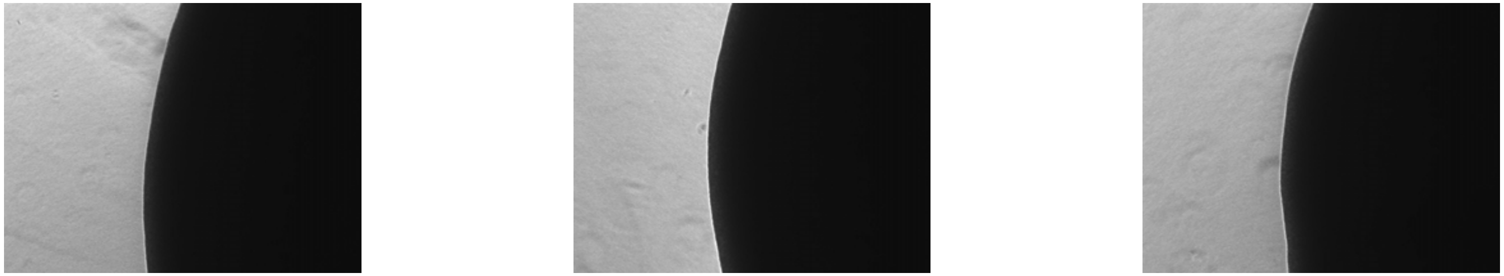

B421-15

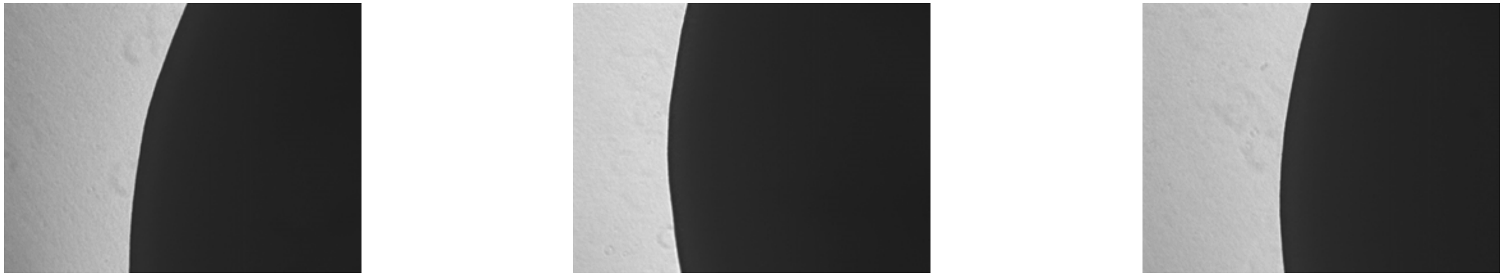

B212-12

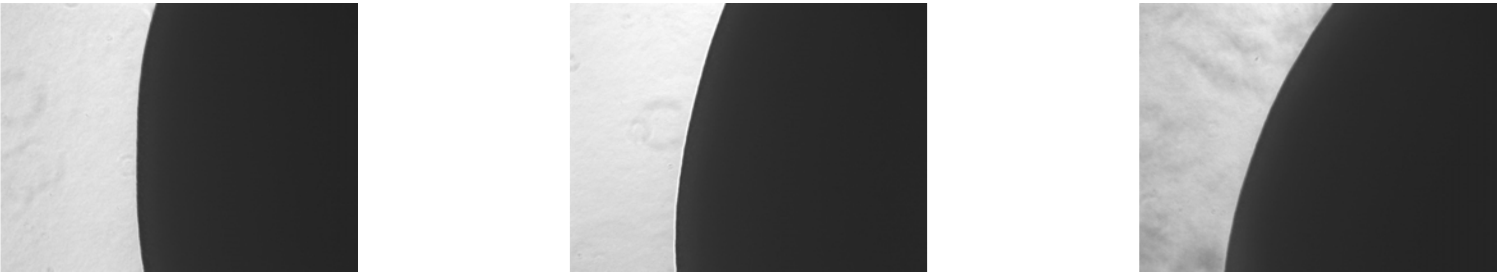

B1091-15

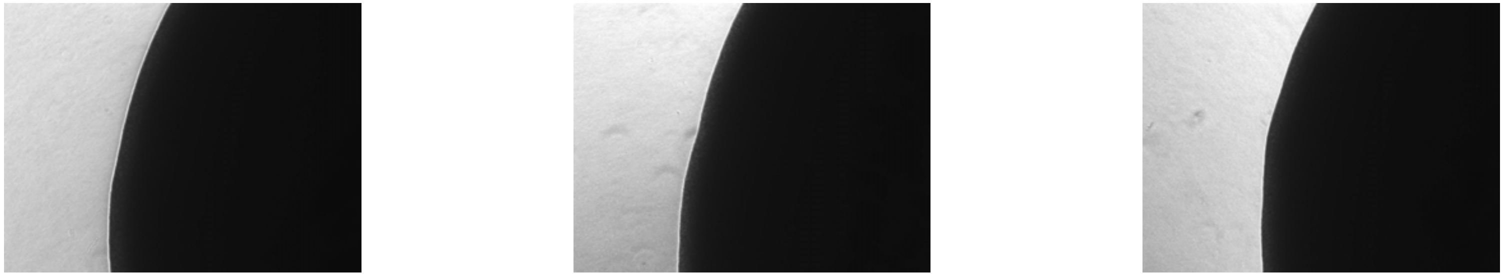

FBS

B510-12

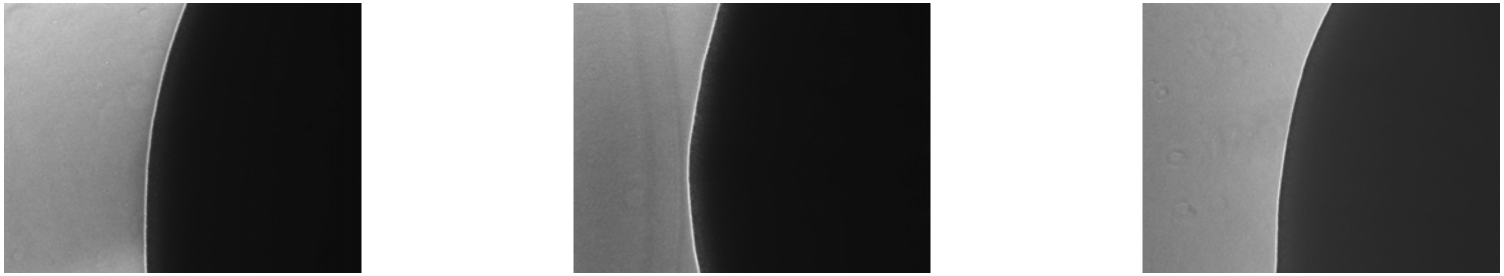

B564-15

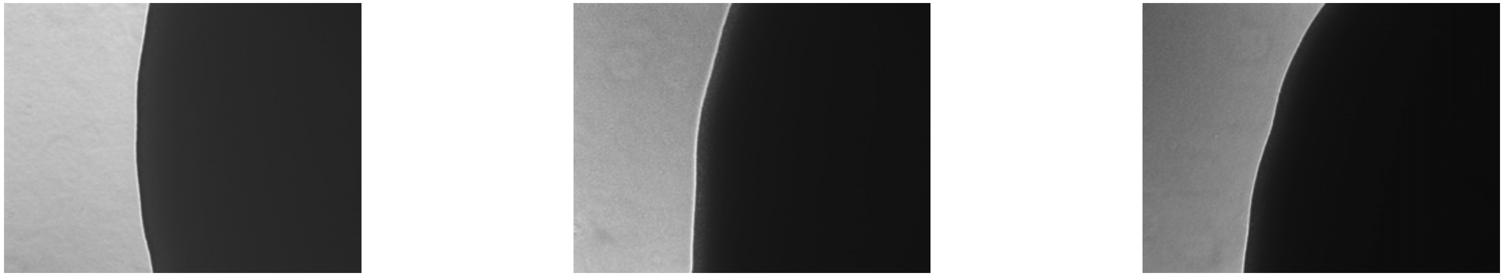

B1168-15

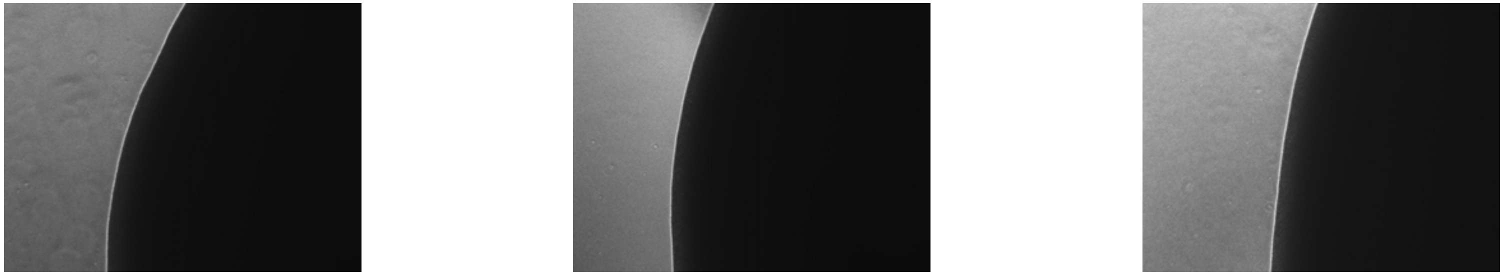

B568-15

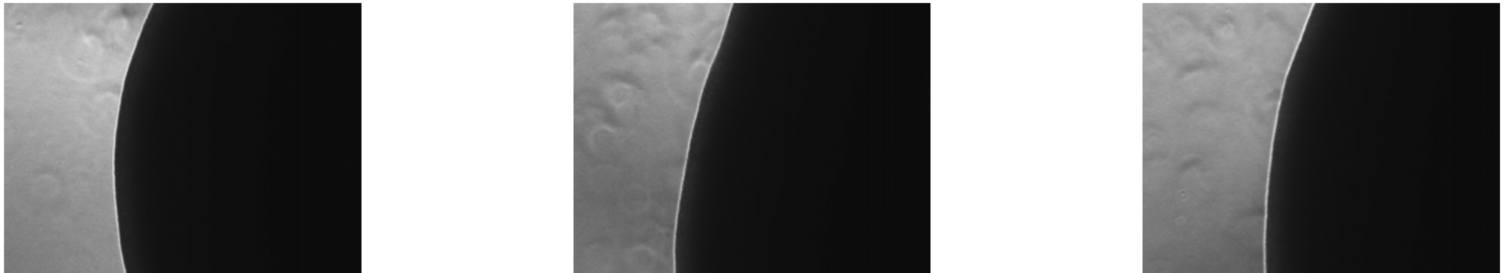

FBS

B2527-12

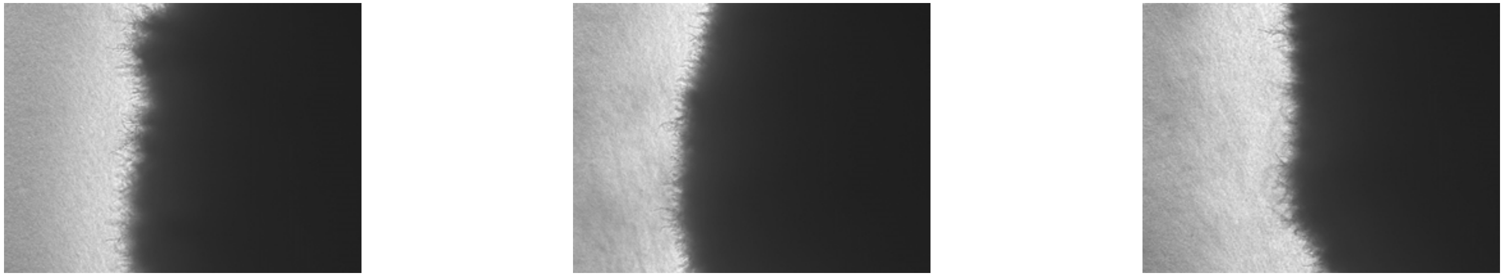

B1486-15

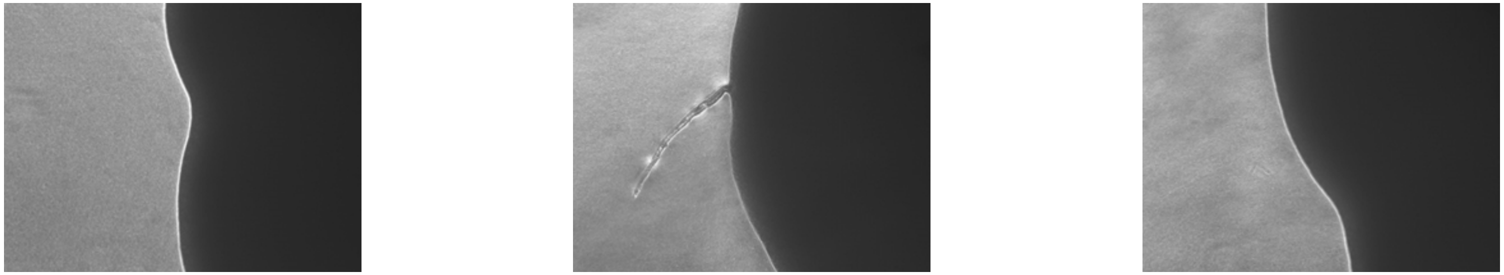

B1559-15

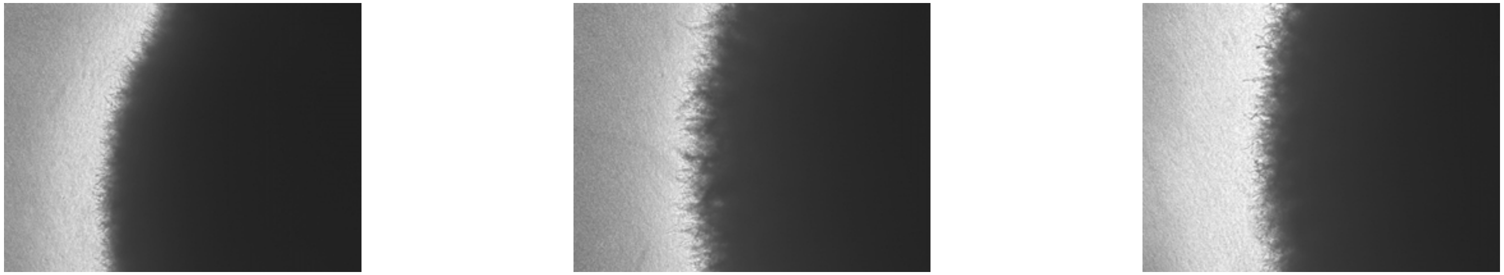

B733-15

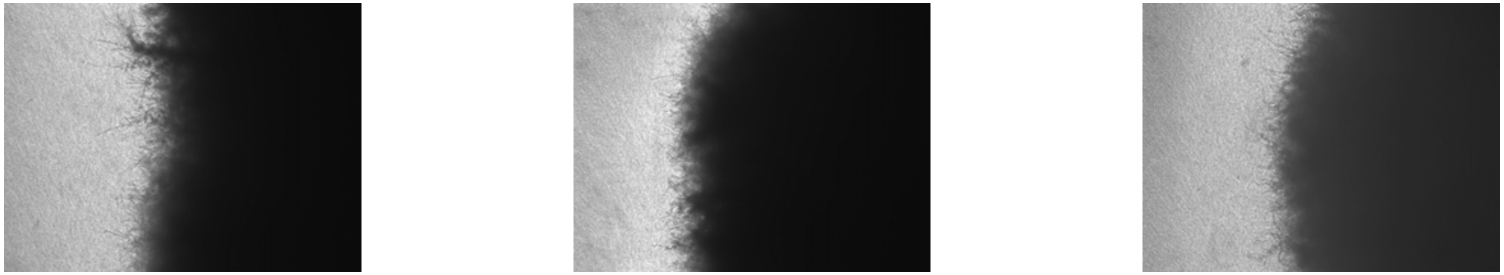

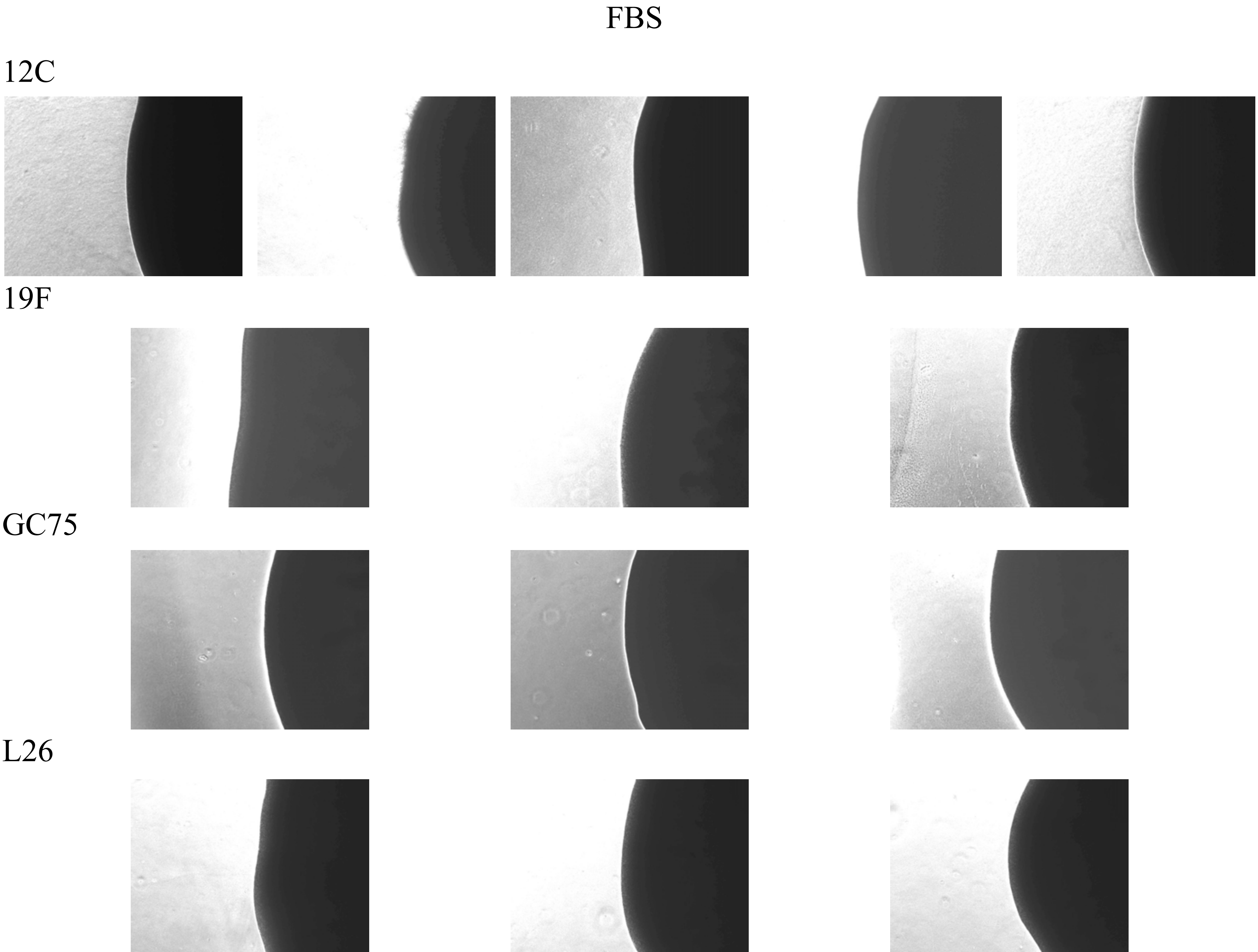

FBS
12C
19F
GC75
L26

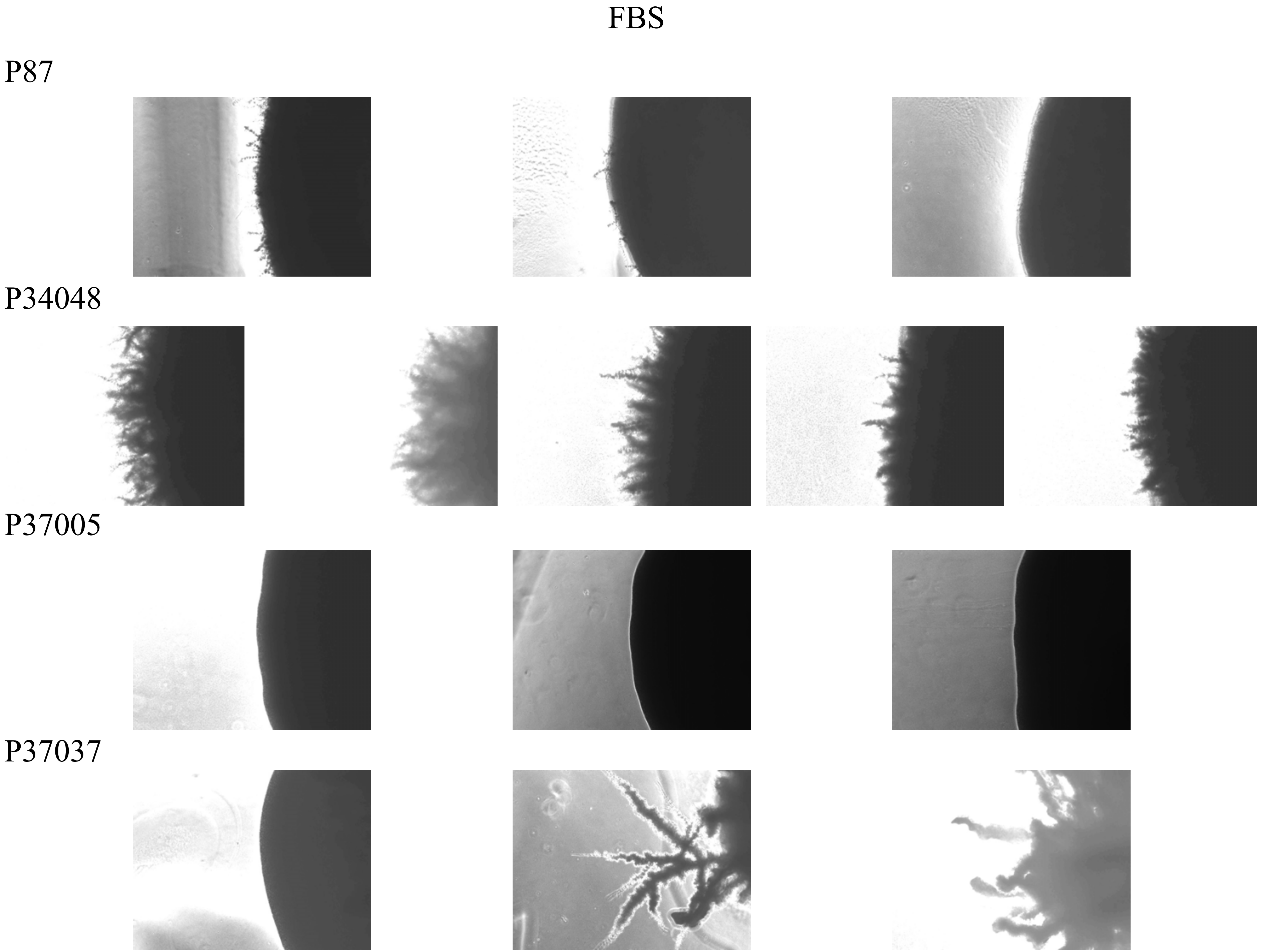
FBS
P87
P34048
P37005
P37037

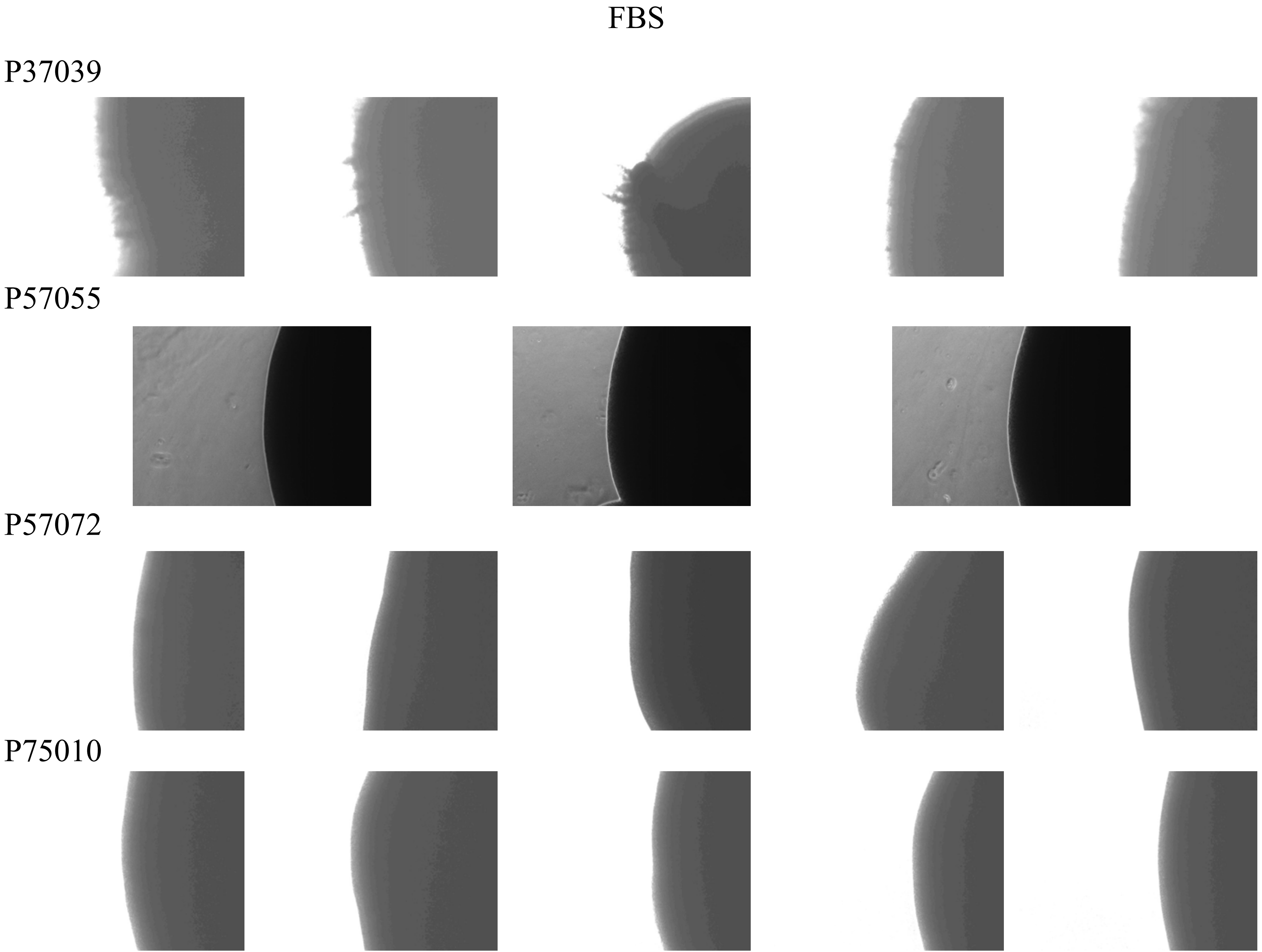
FBS
P37039
P57055
P57072
P75010

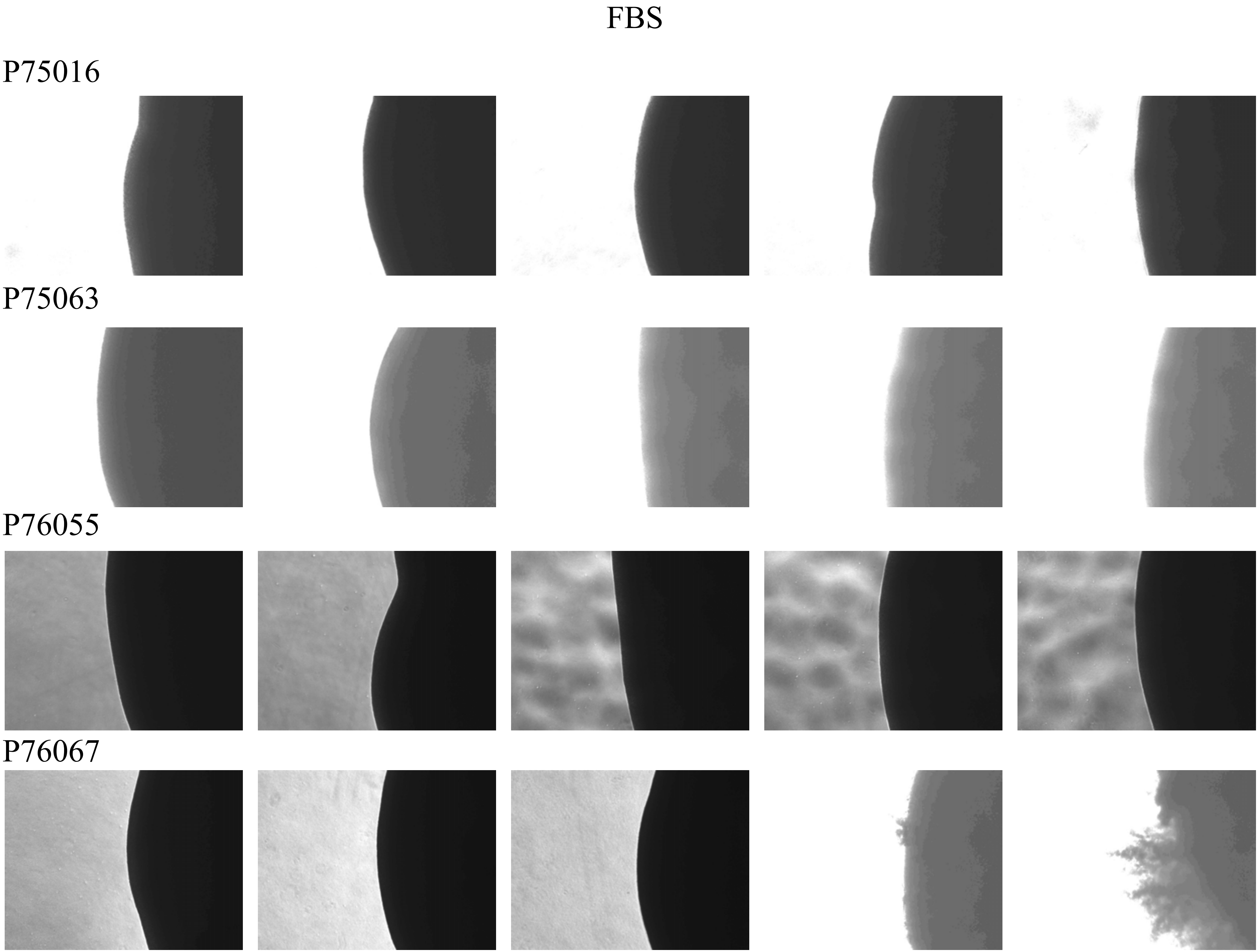
FBS
P75016
P75063
P76055
P76067

FBS

P78042

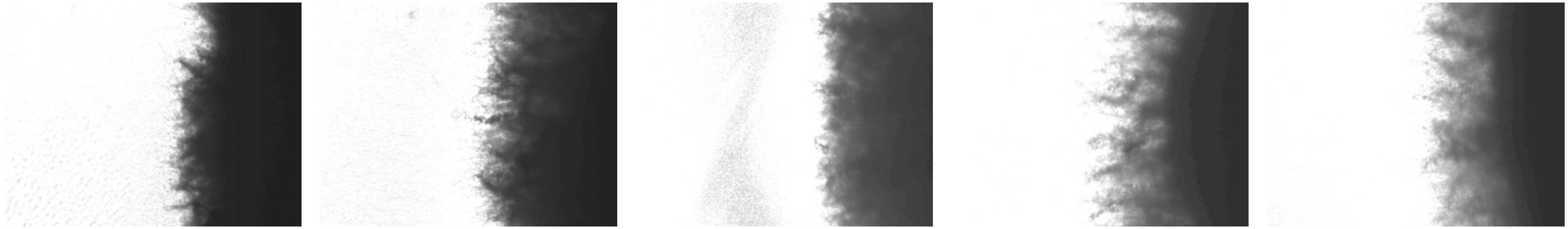

P78048

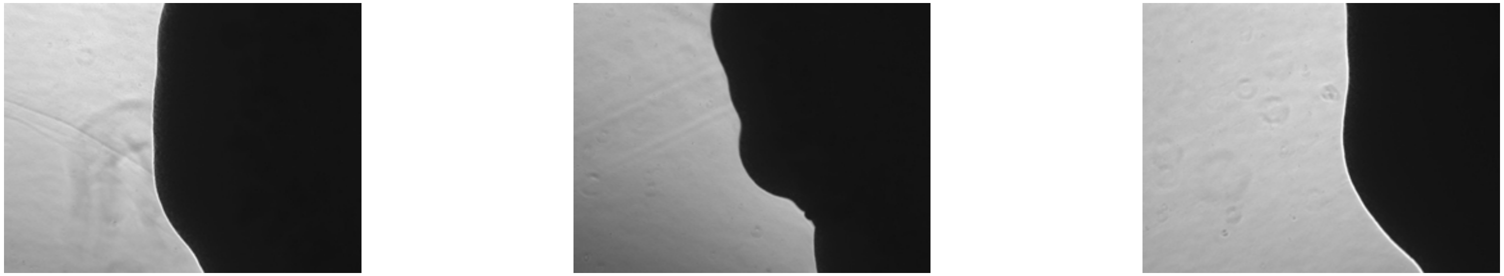

P94015

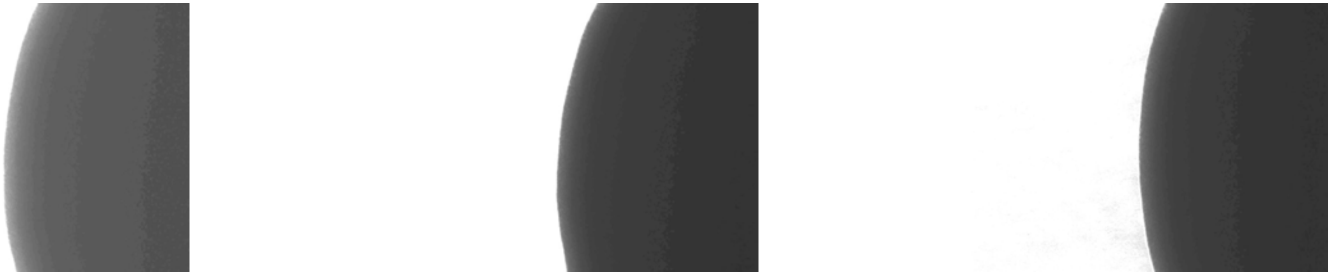

P60002

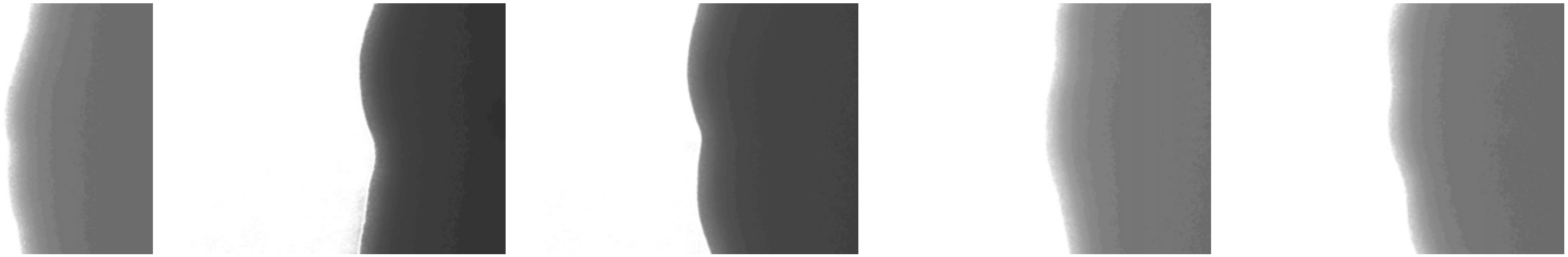

Lee's

B444-12

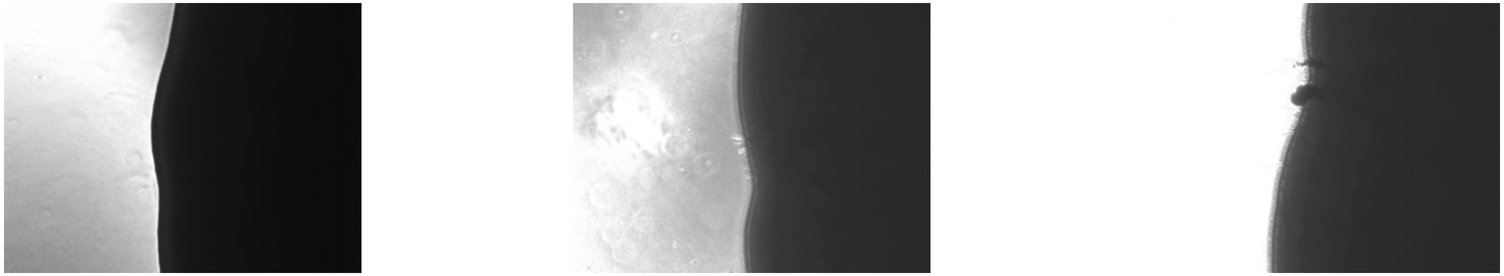

B1257-15

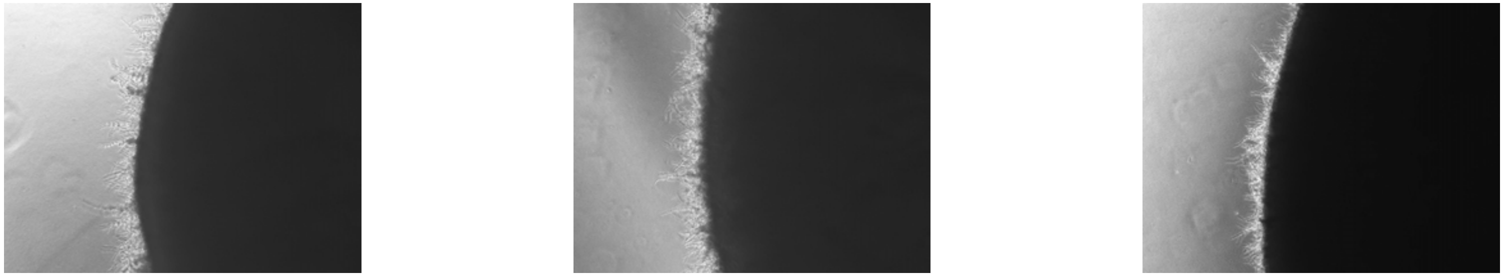

B687-15

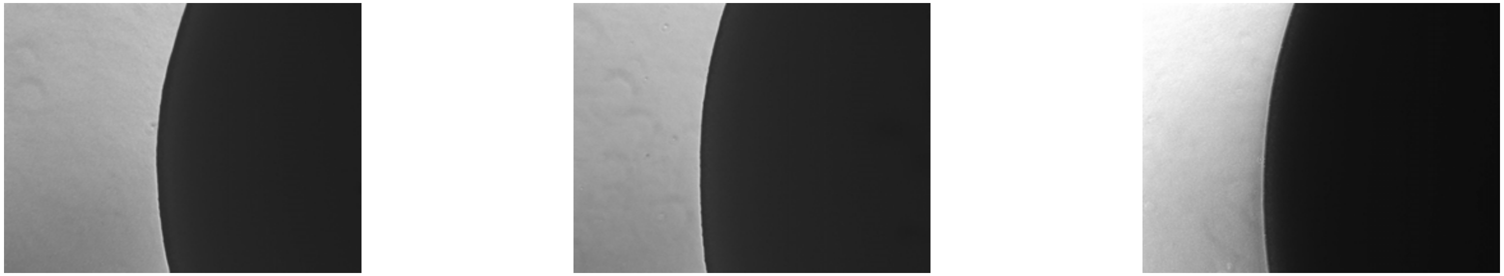

B1762-15

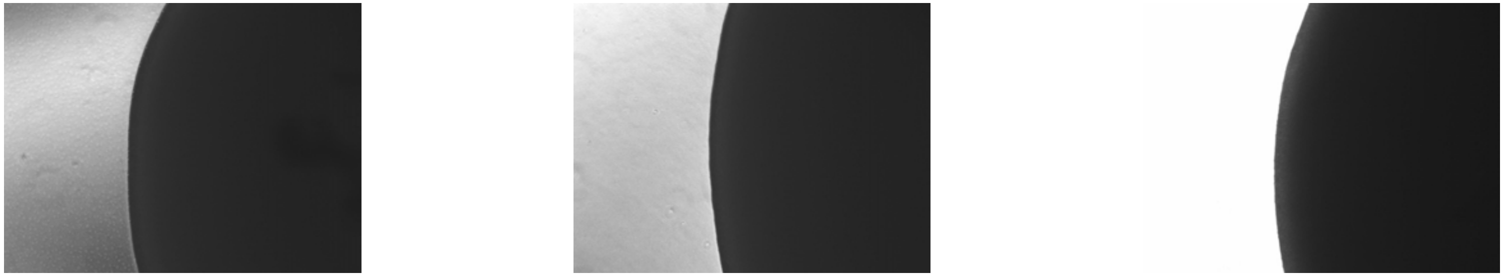

Lee's

B46-15

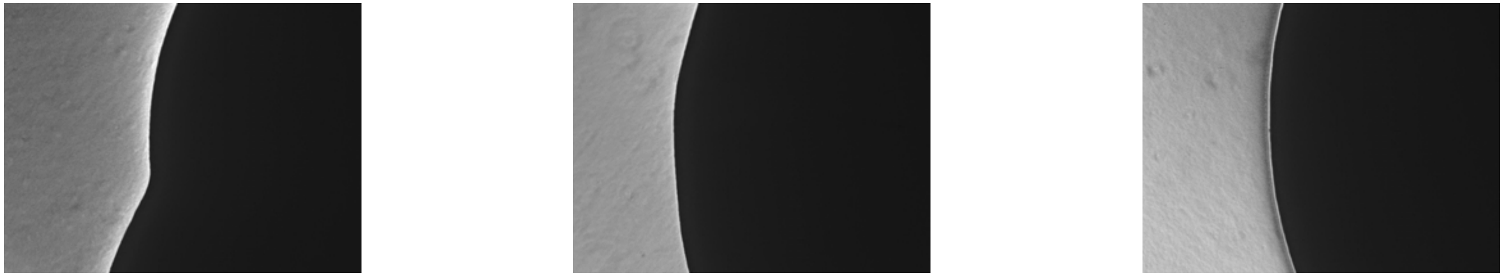

B808-15

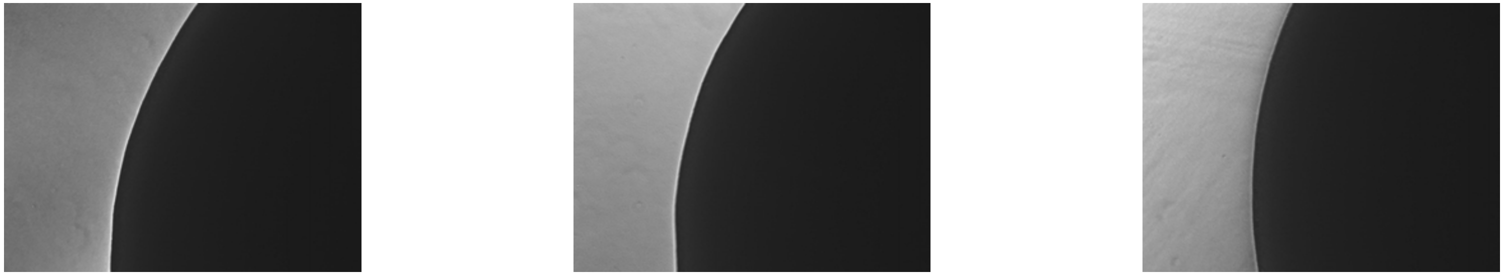

B527-15

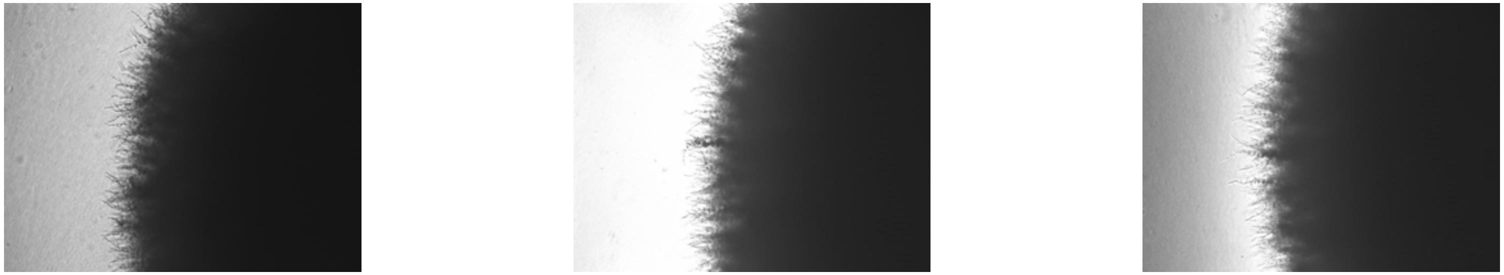

B618-15

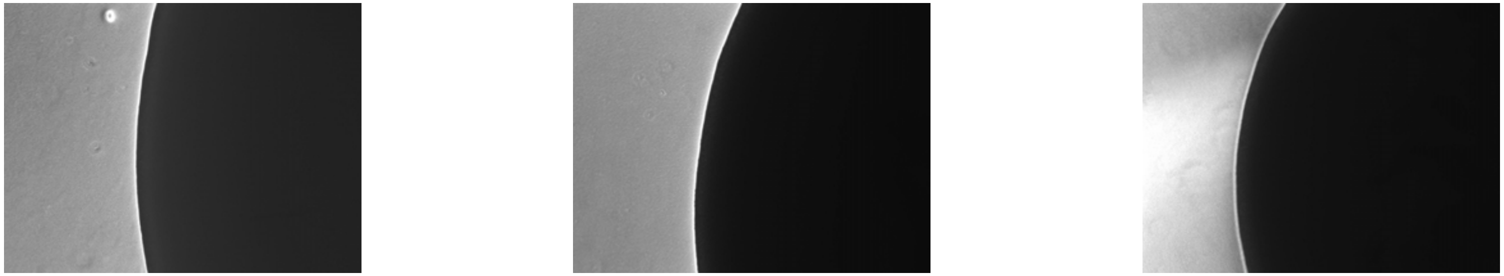

Lee's

B404-15

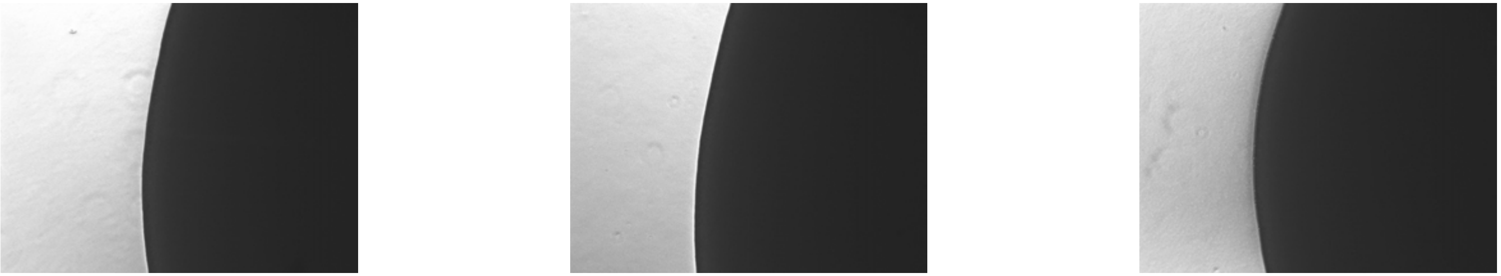

B421-15

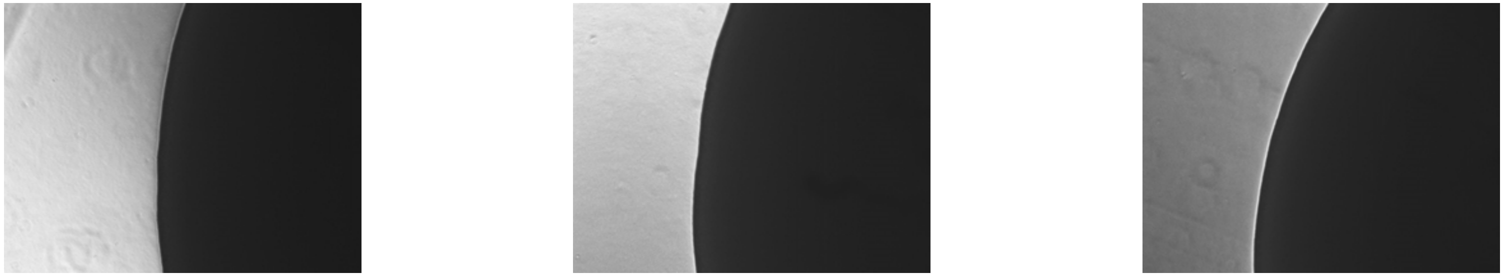

B212-12

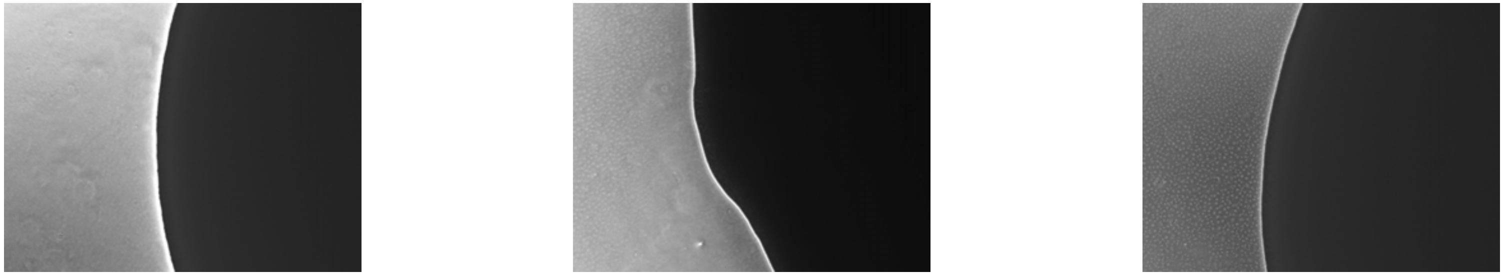

B1091-15

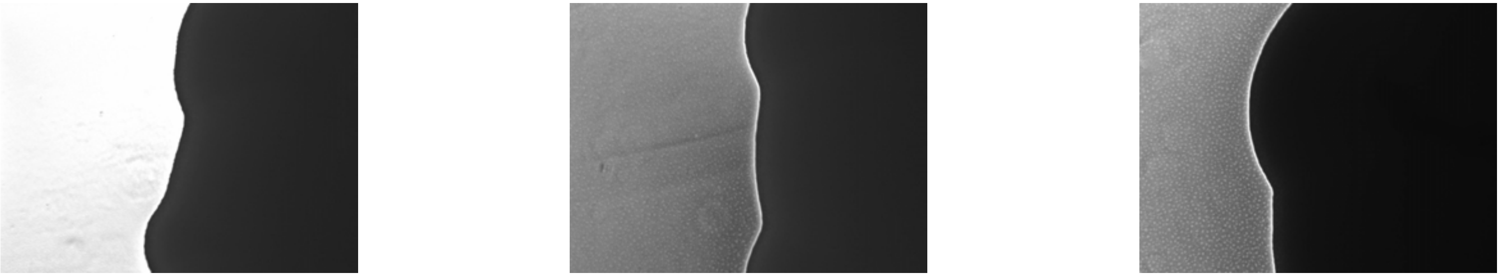

Lee's

B510-12

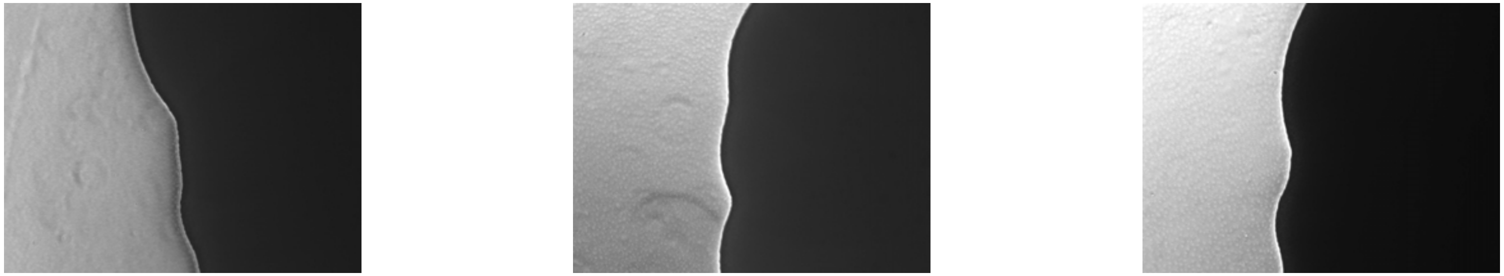

B564-15

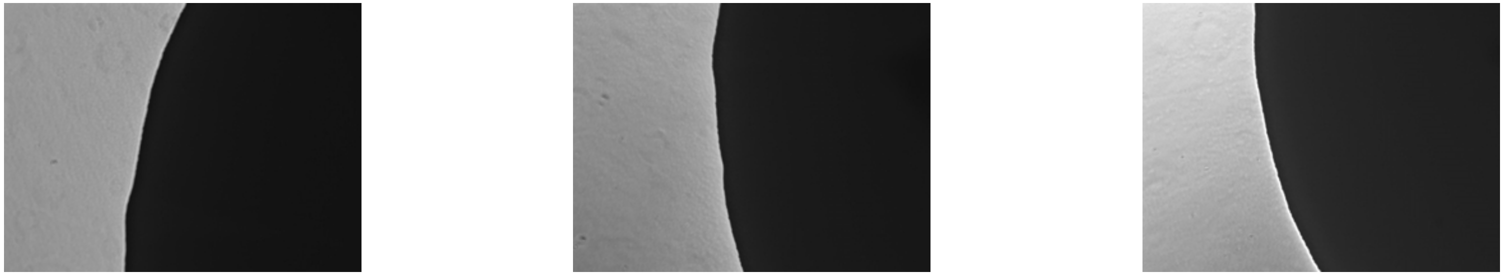

B1168-15

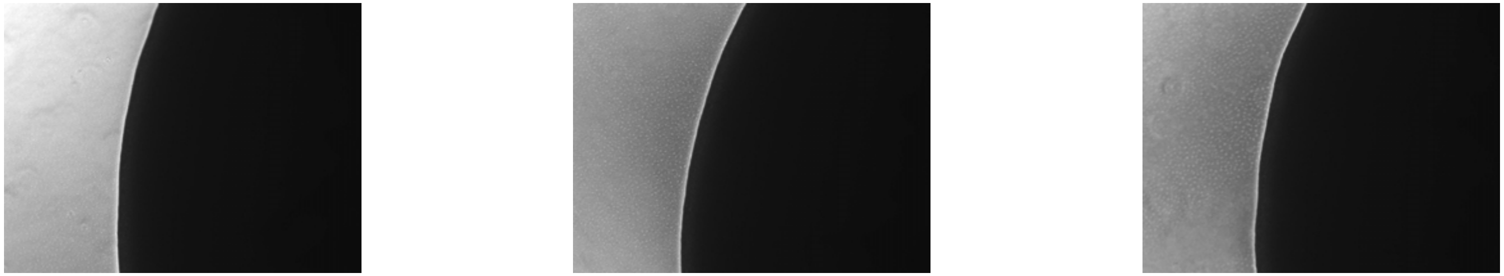

B568-15

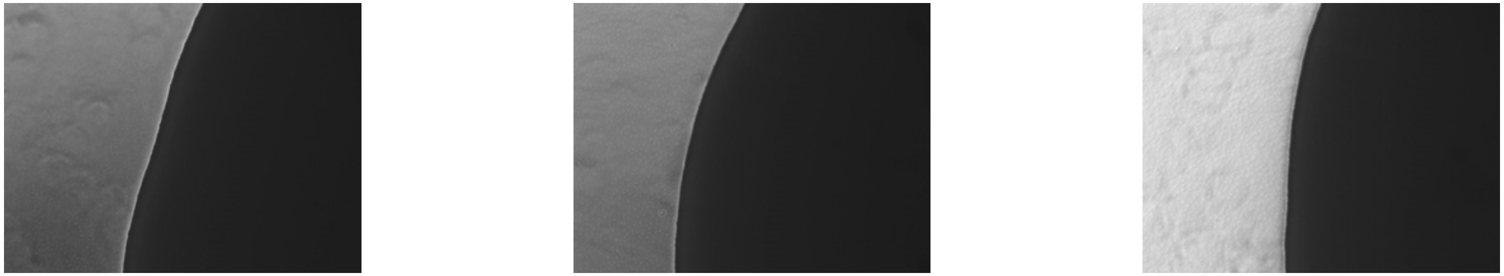

Lee's

B2527-12

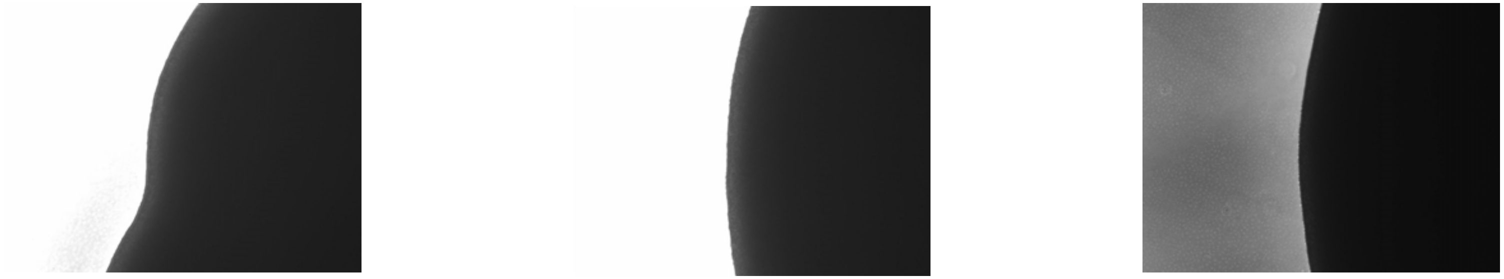

B1486-15

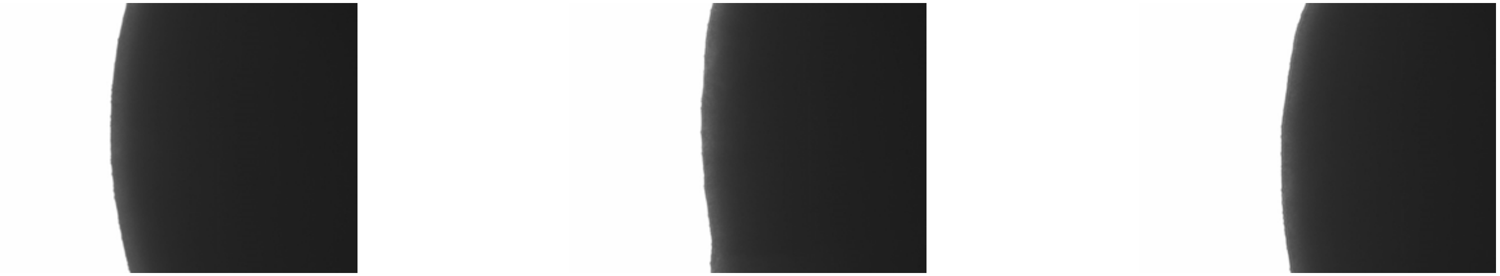

B1559-15

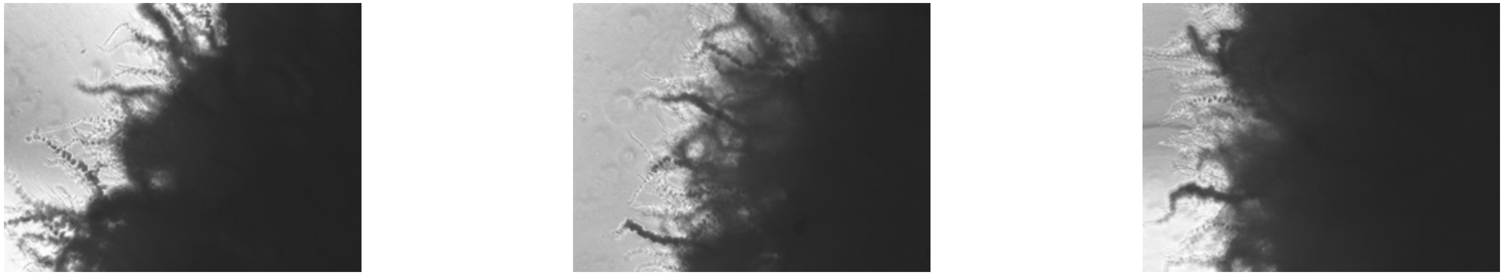

B733-15

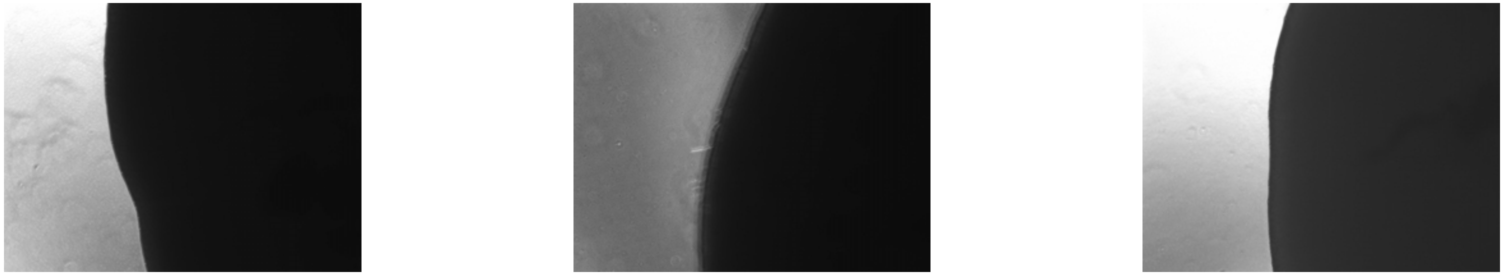

Lee's

12C

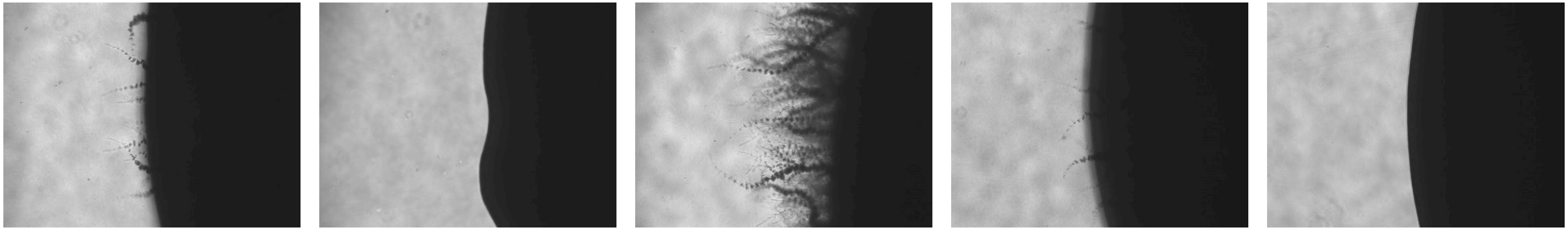

19F

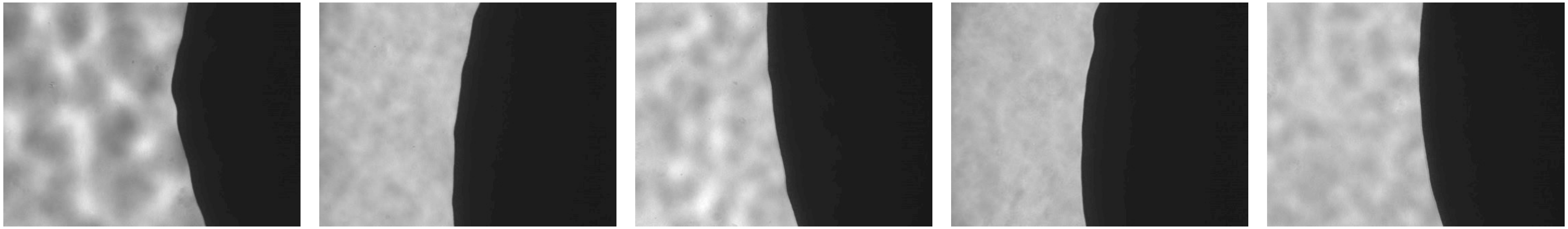

GC75

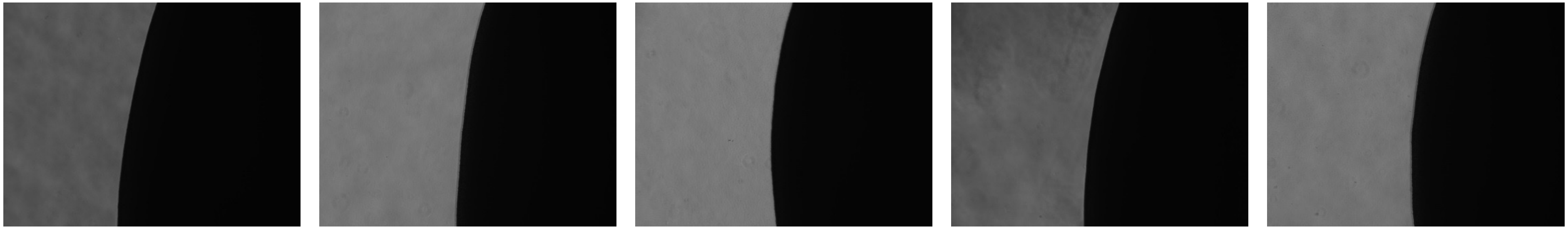

L26

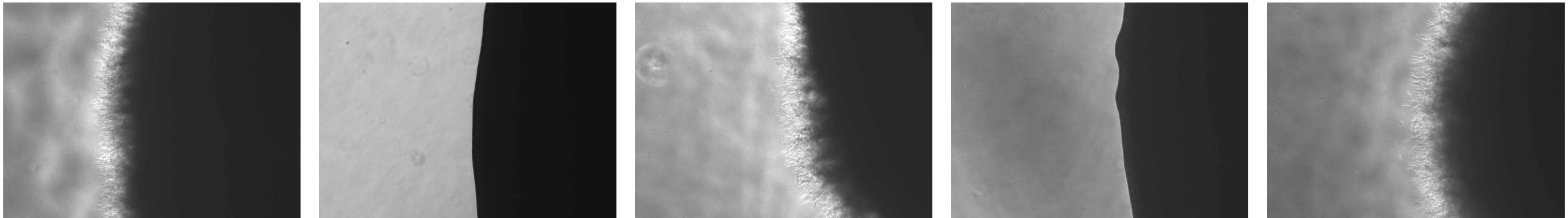

Lee's

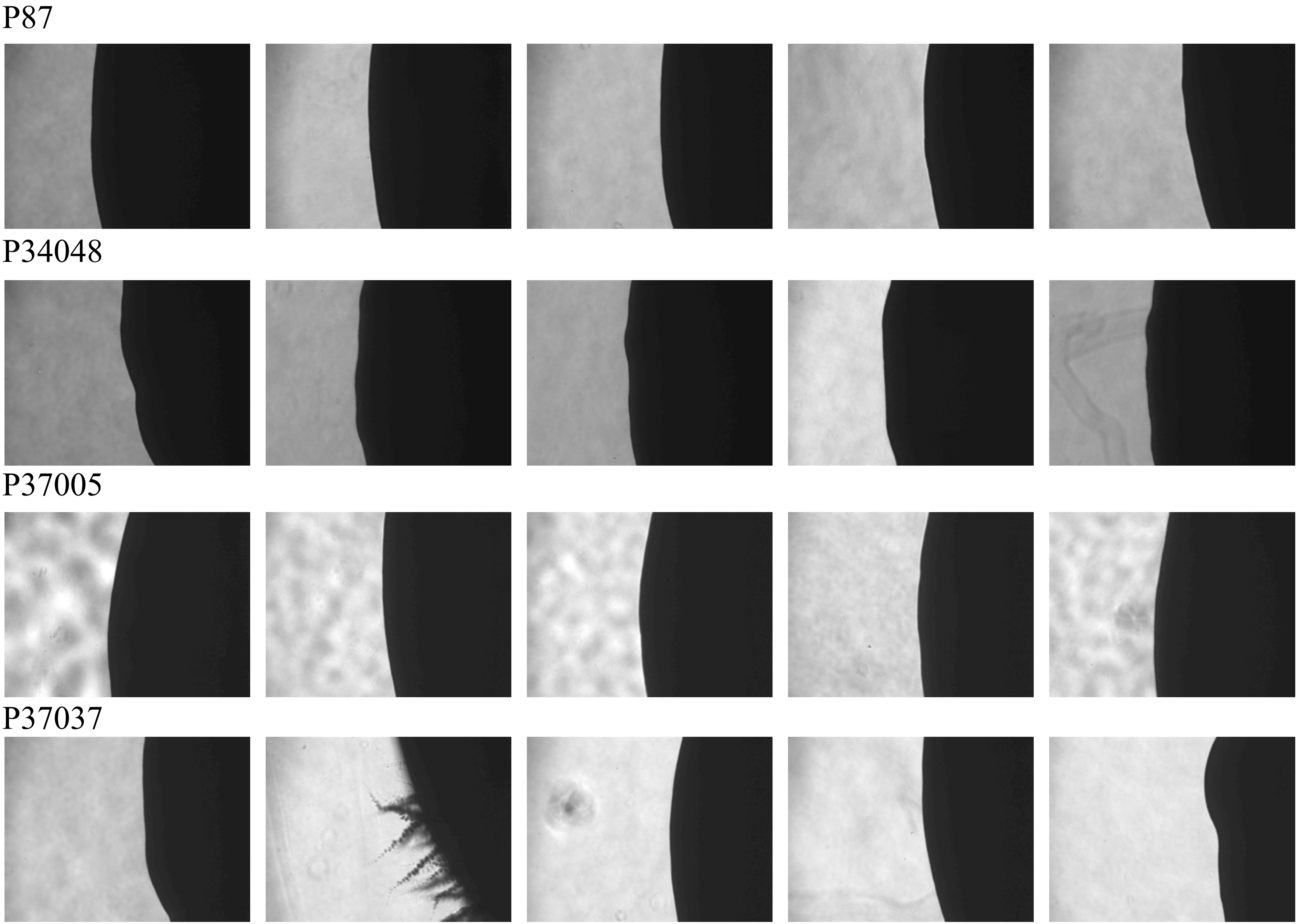

Lee's

P37039

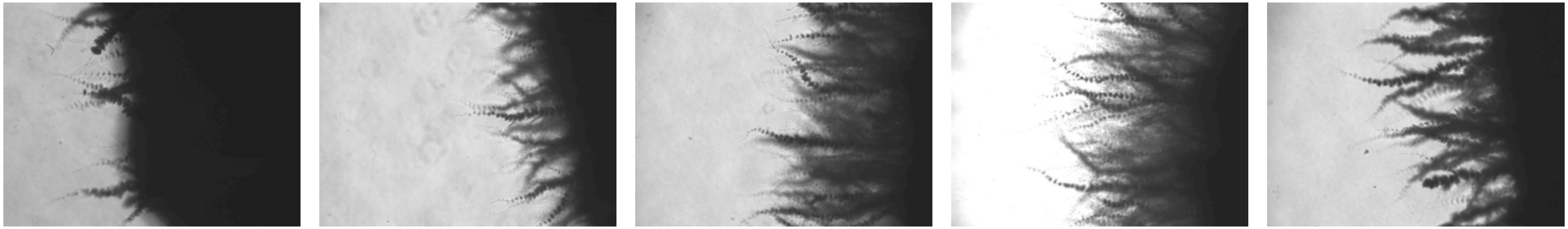

P57055

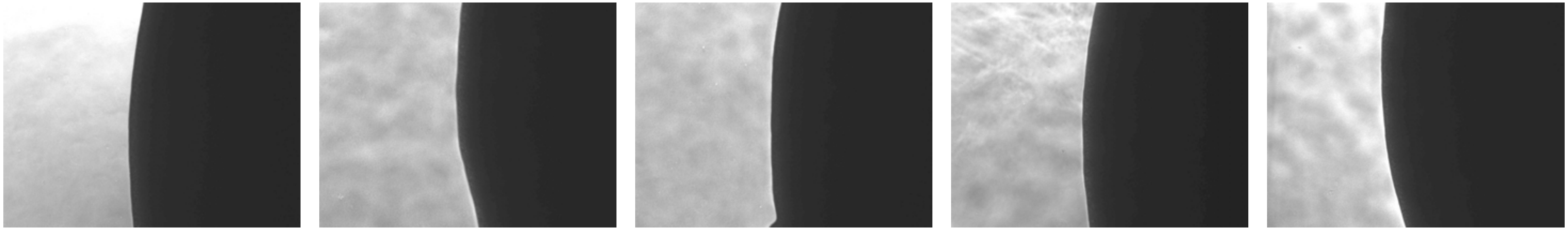

P57072

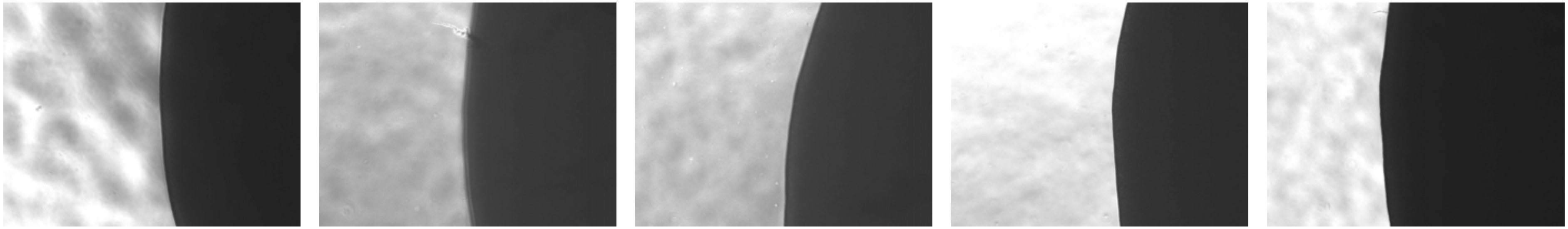

P75010

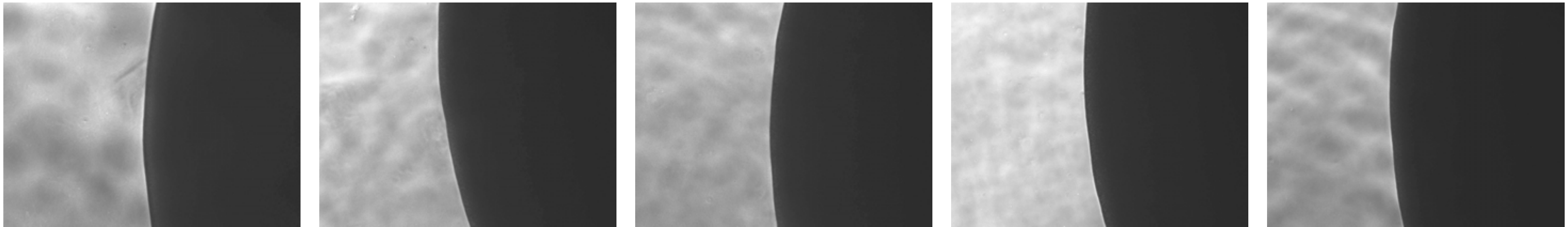

Lee's

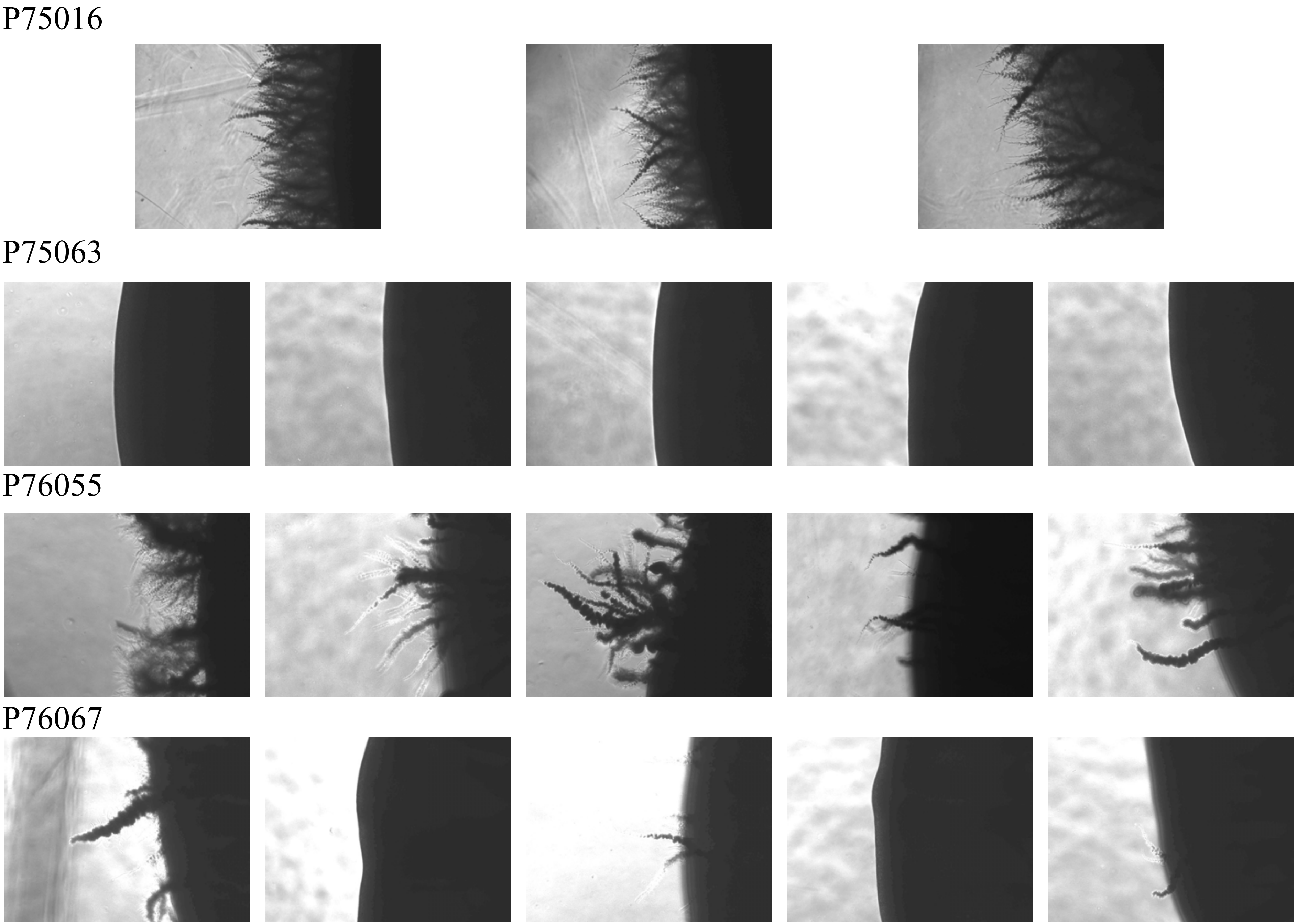

Lee's

P78042

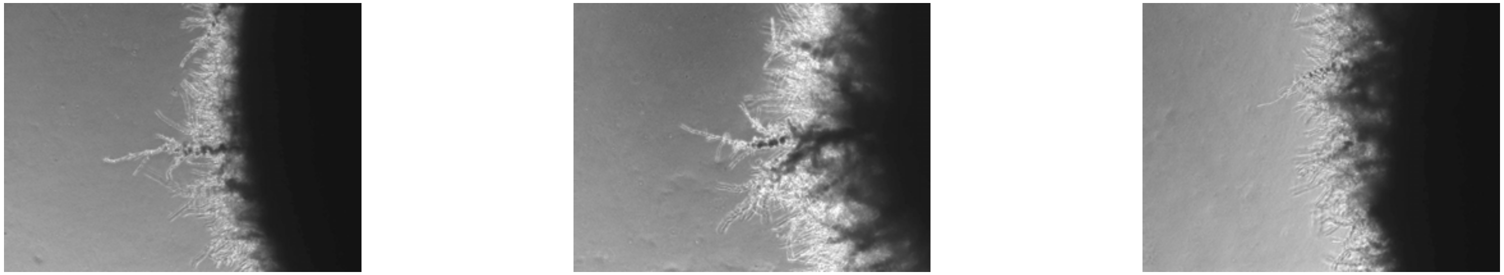

P78048

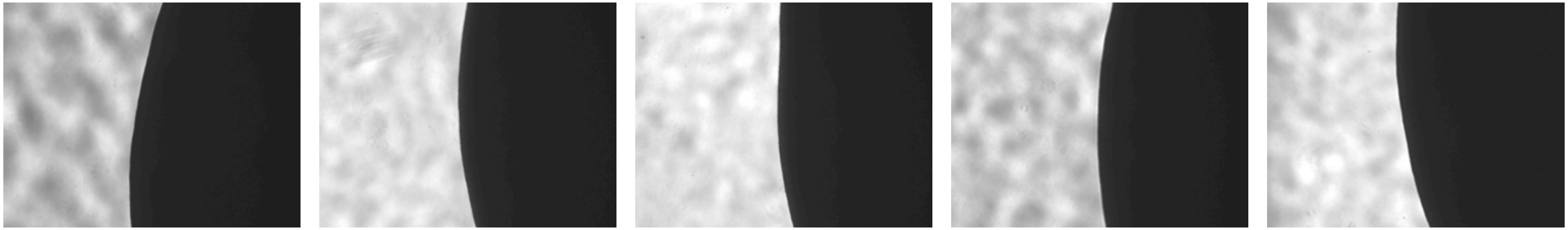

P94015

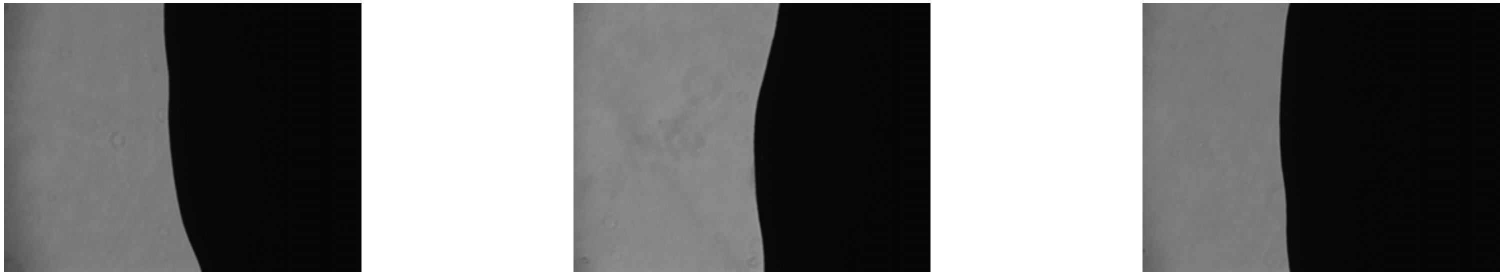

P60002

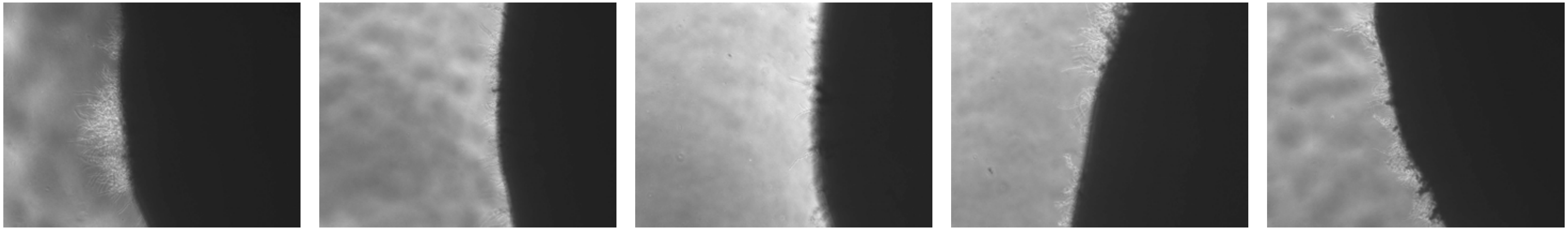

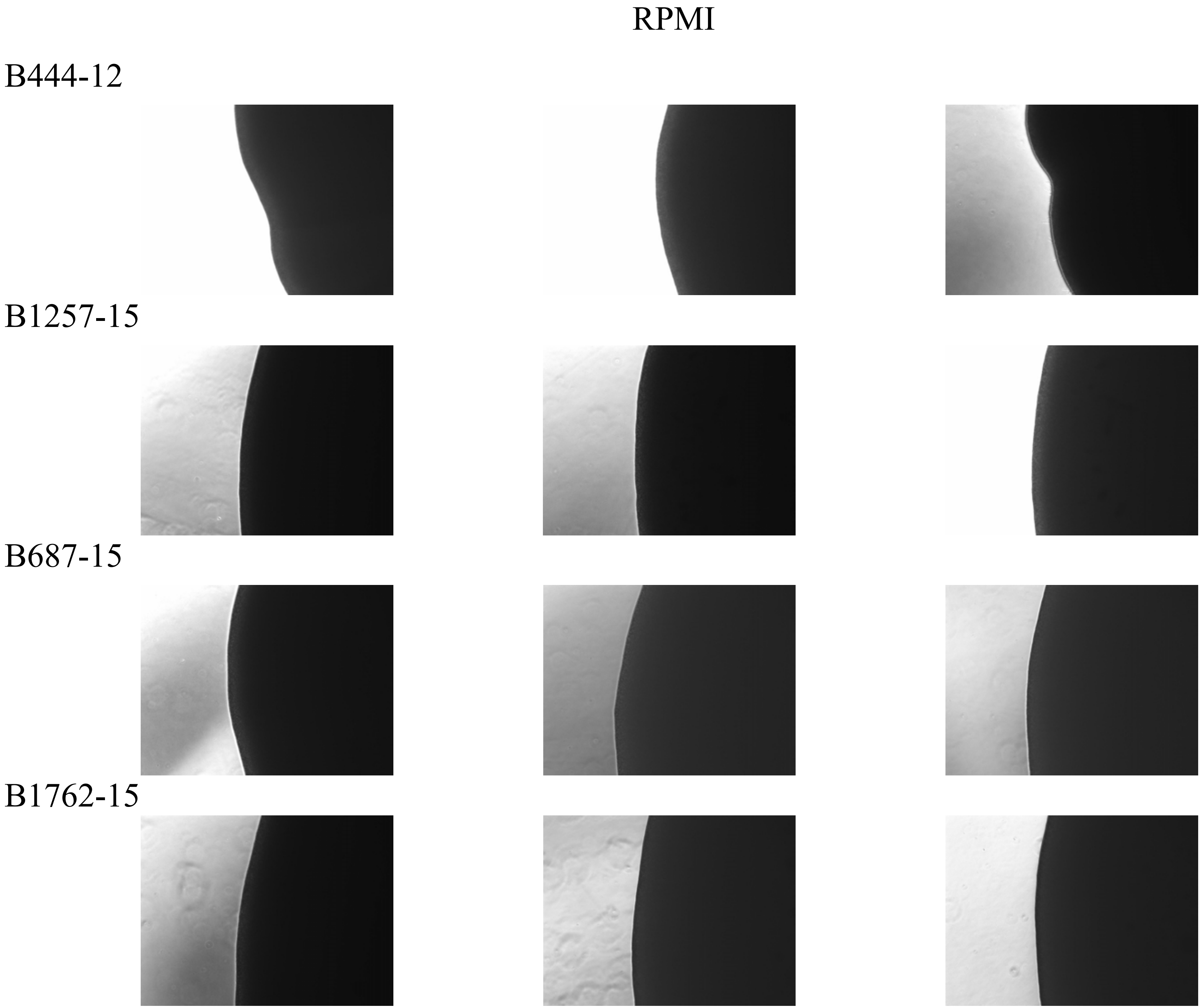
RPMI
B444-12
B1257-15
B687-15
B1762-15

RPMI

B46-15

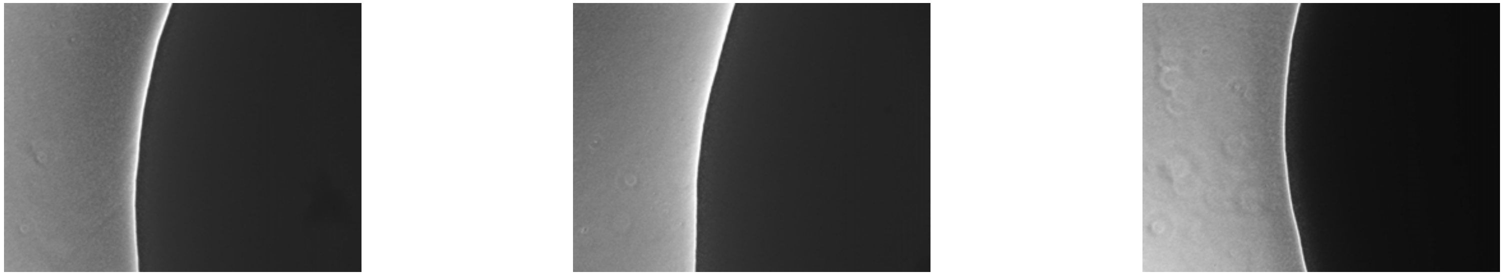

B808-15

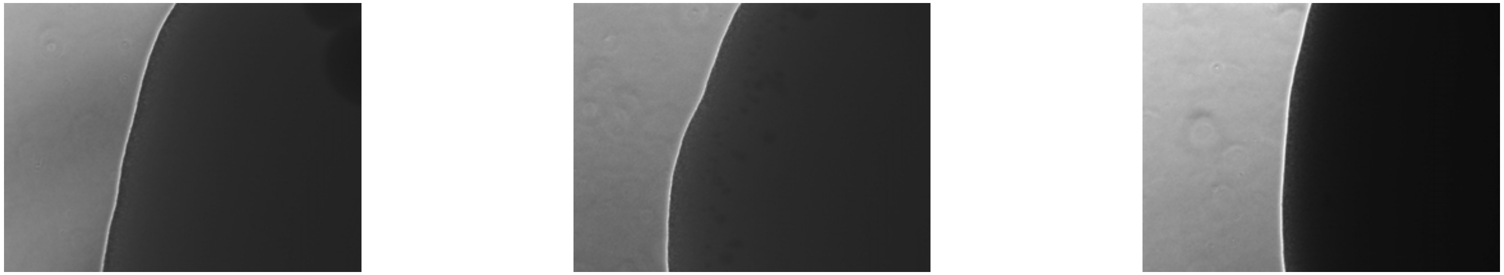

B527-15

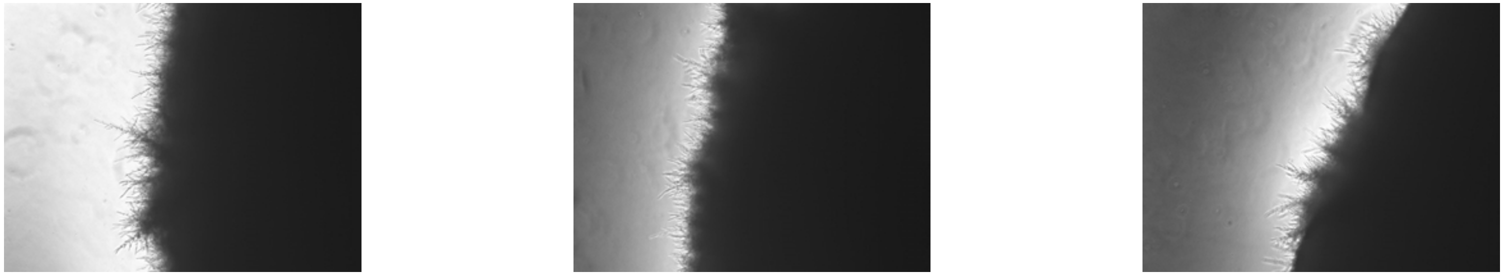

B618-15

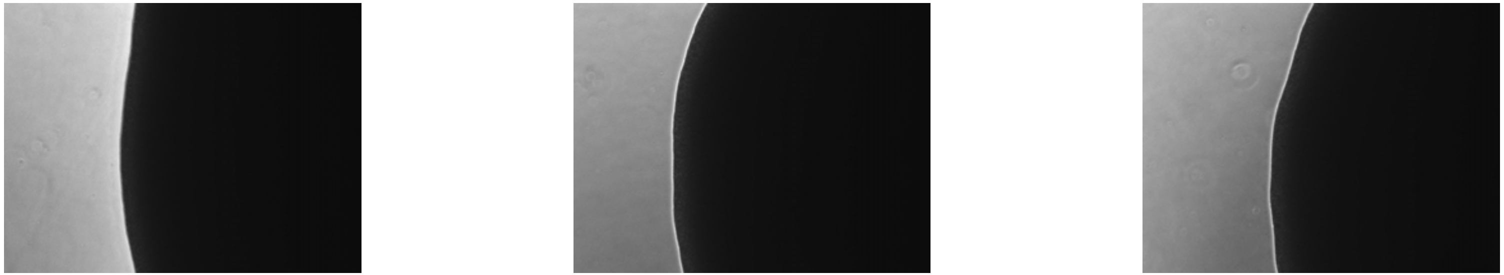

RPMI

B404-15

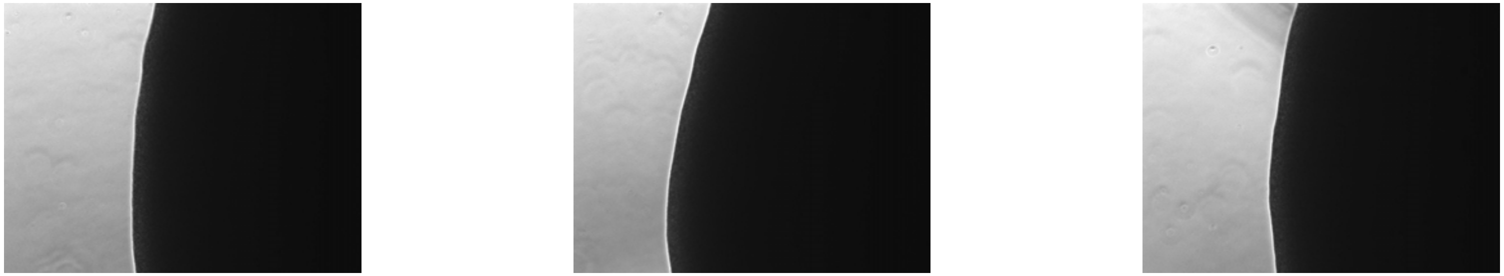

B421-15

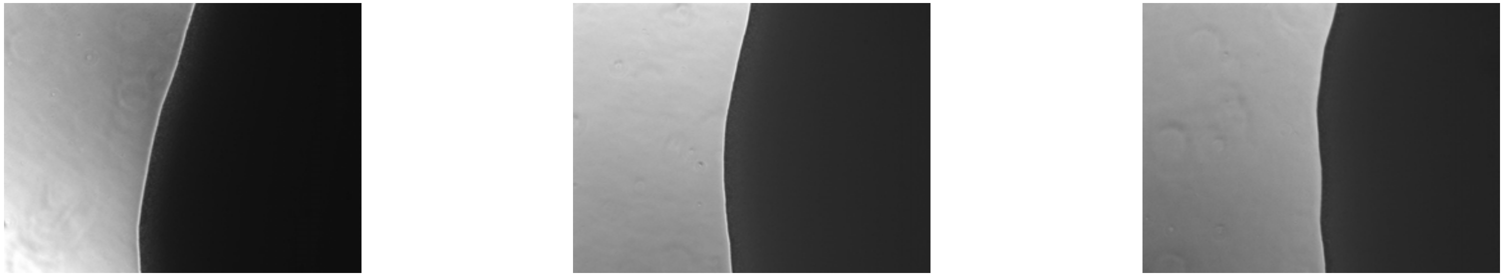

B212-12

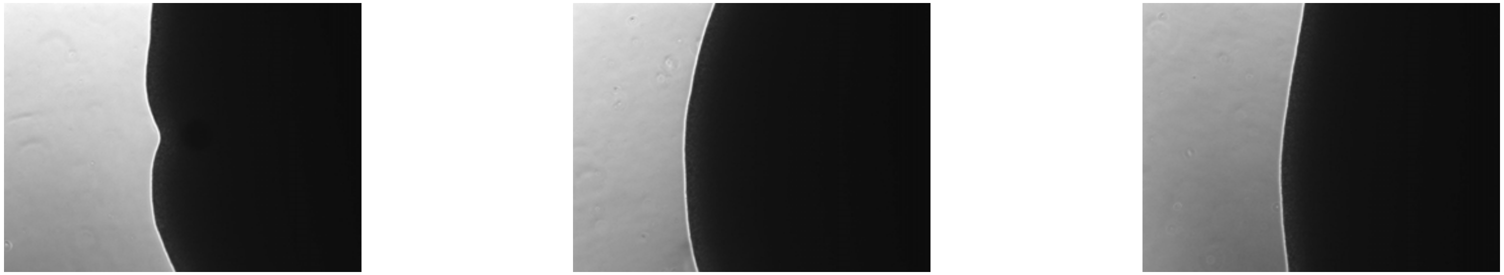

B1091-15

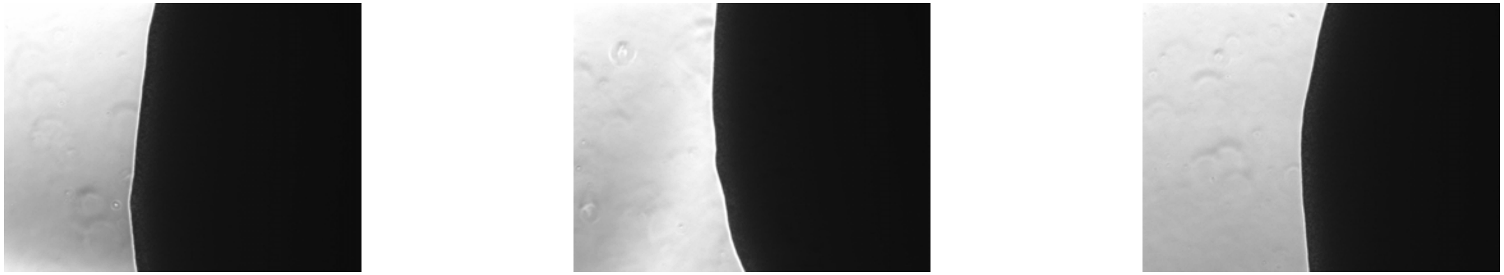

RPMI

B510-12

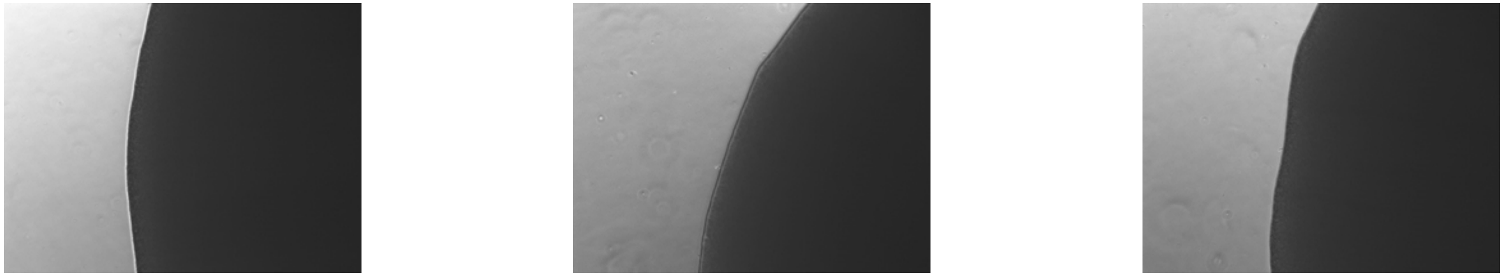

B564-15

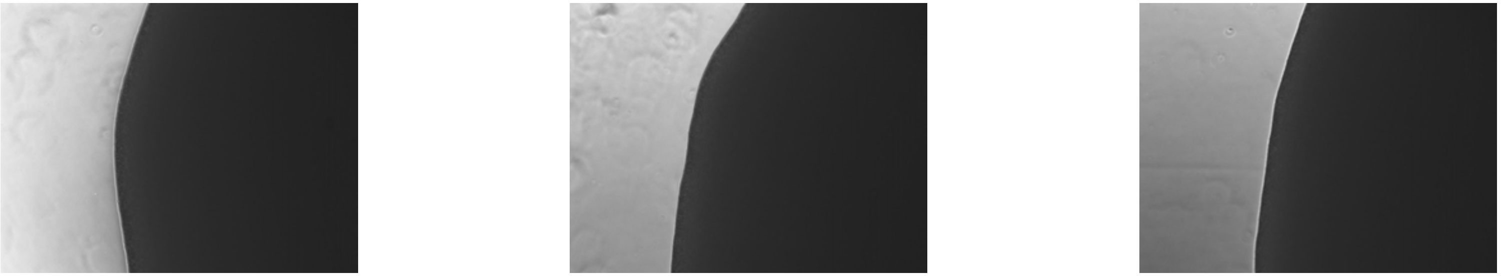

B1168-15

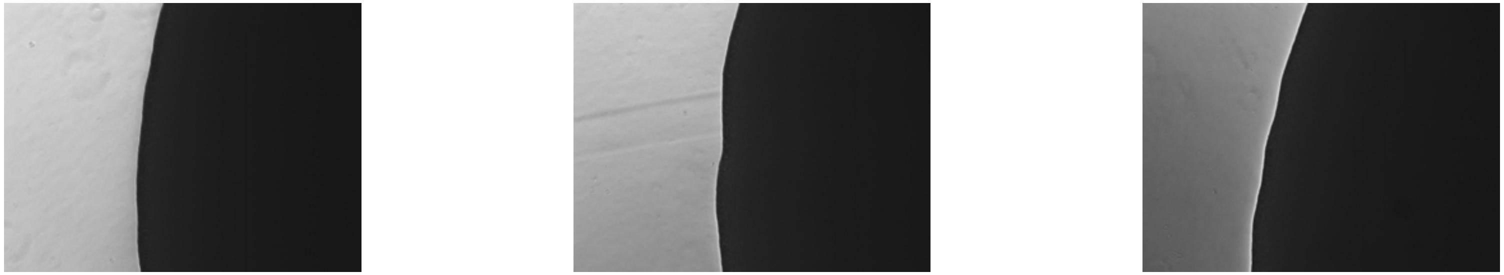

B568-15

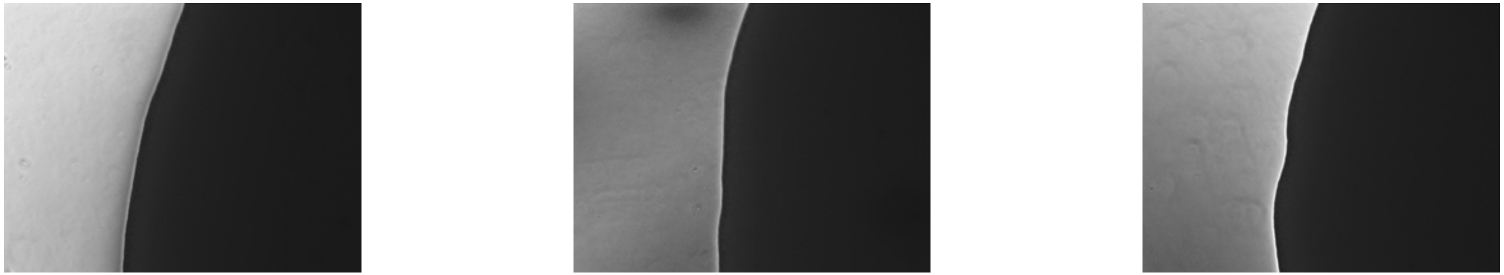

RPMI

B2527-12

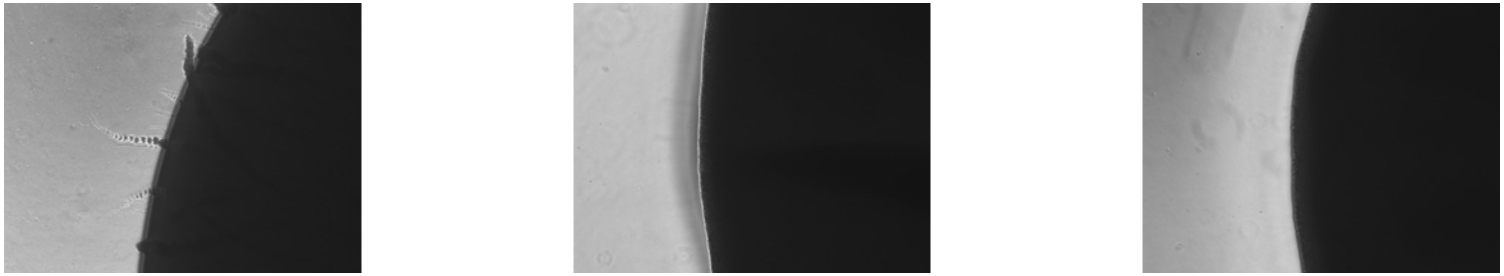

B1486-15

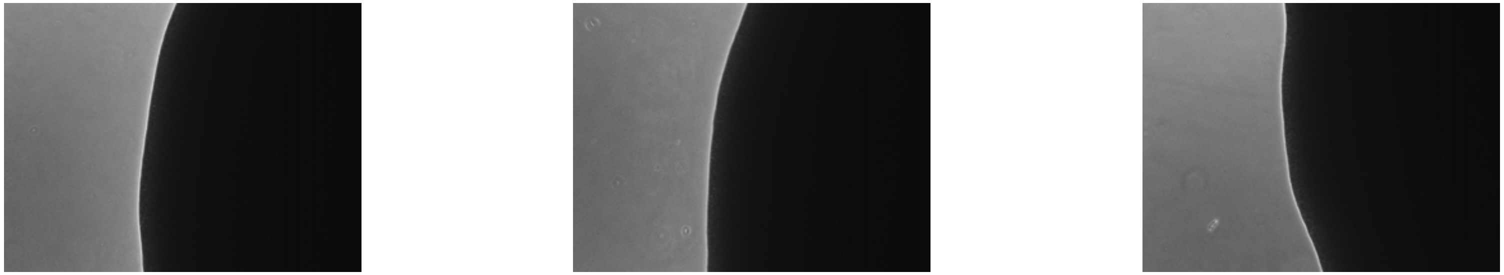

B1559-15

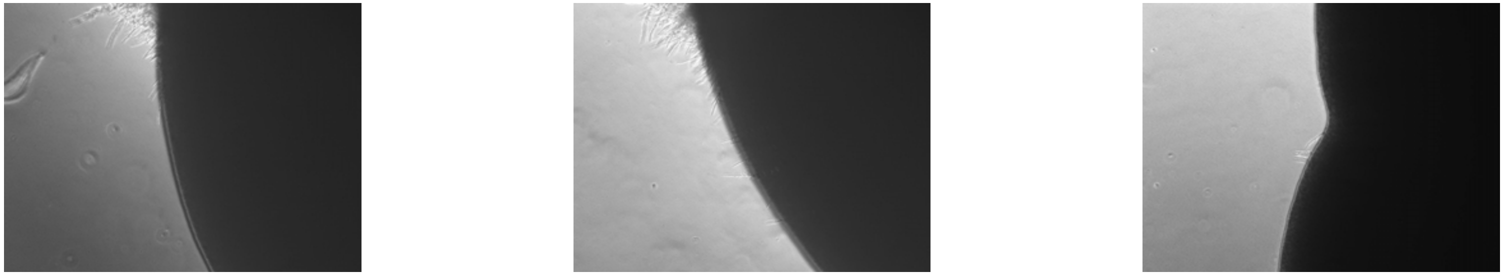

B733-15

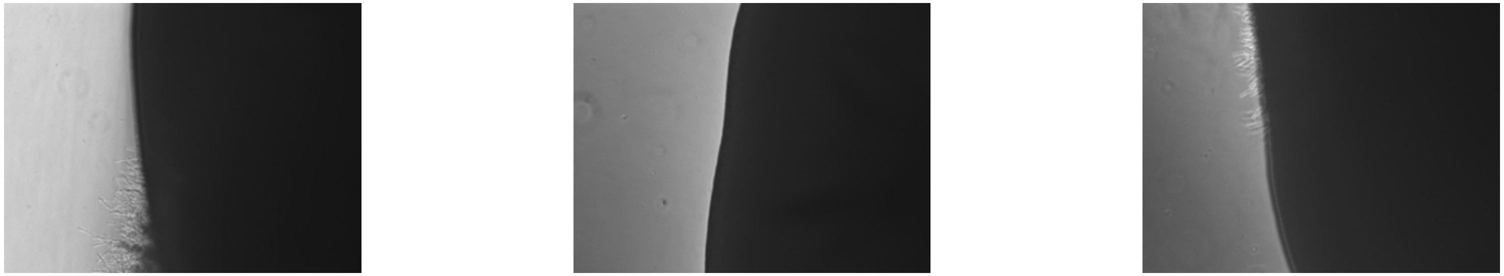

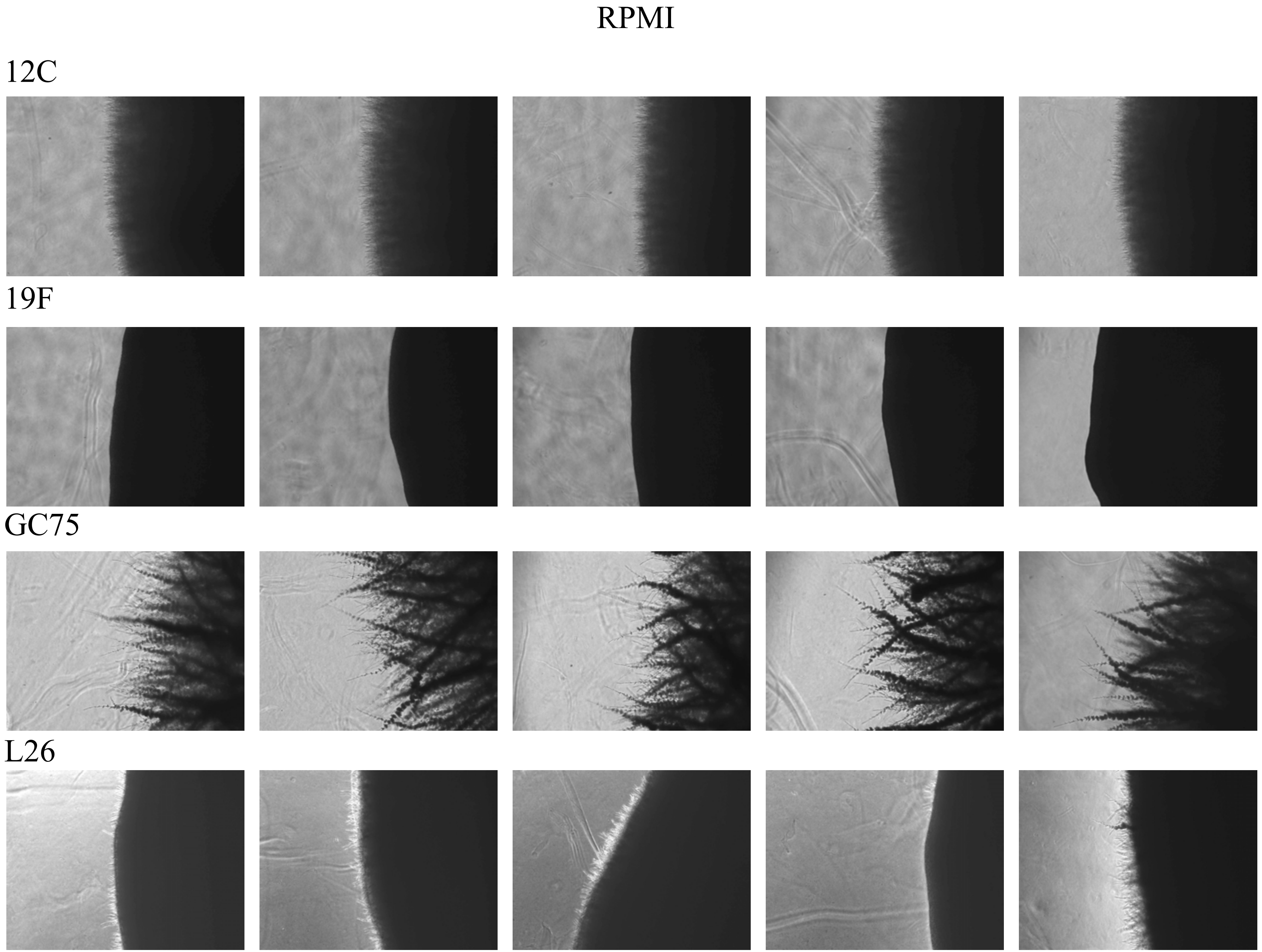
RPMI
12C
19F
GC75
L26

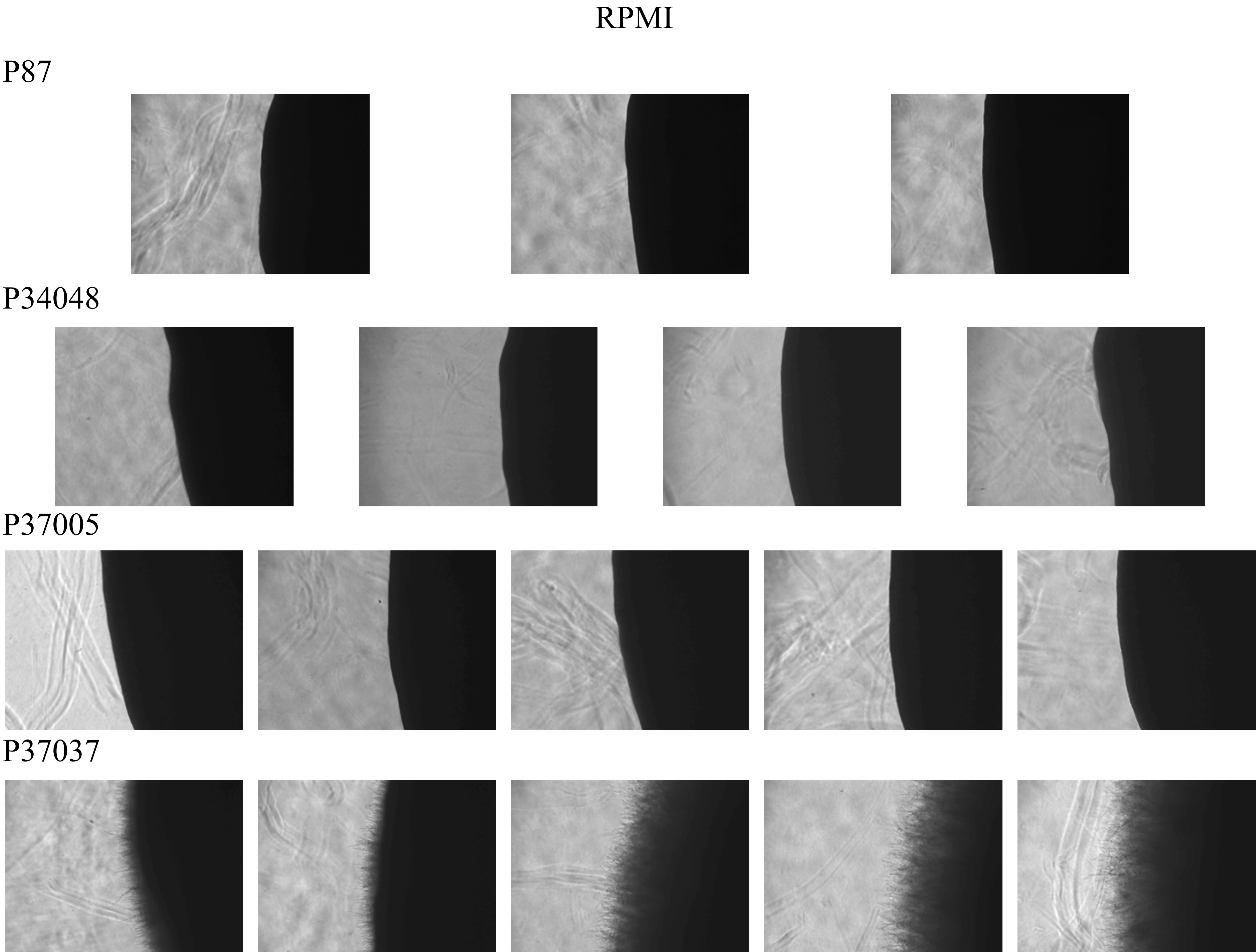
RPMI
P87
P34048
P37005
P37037

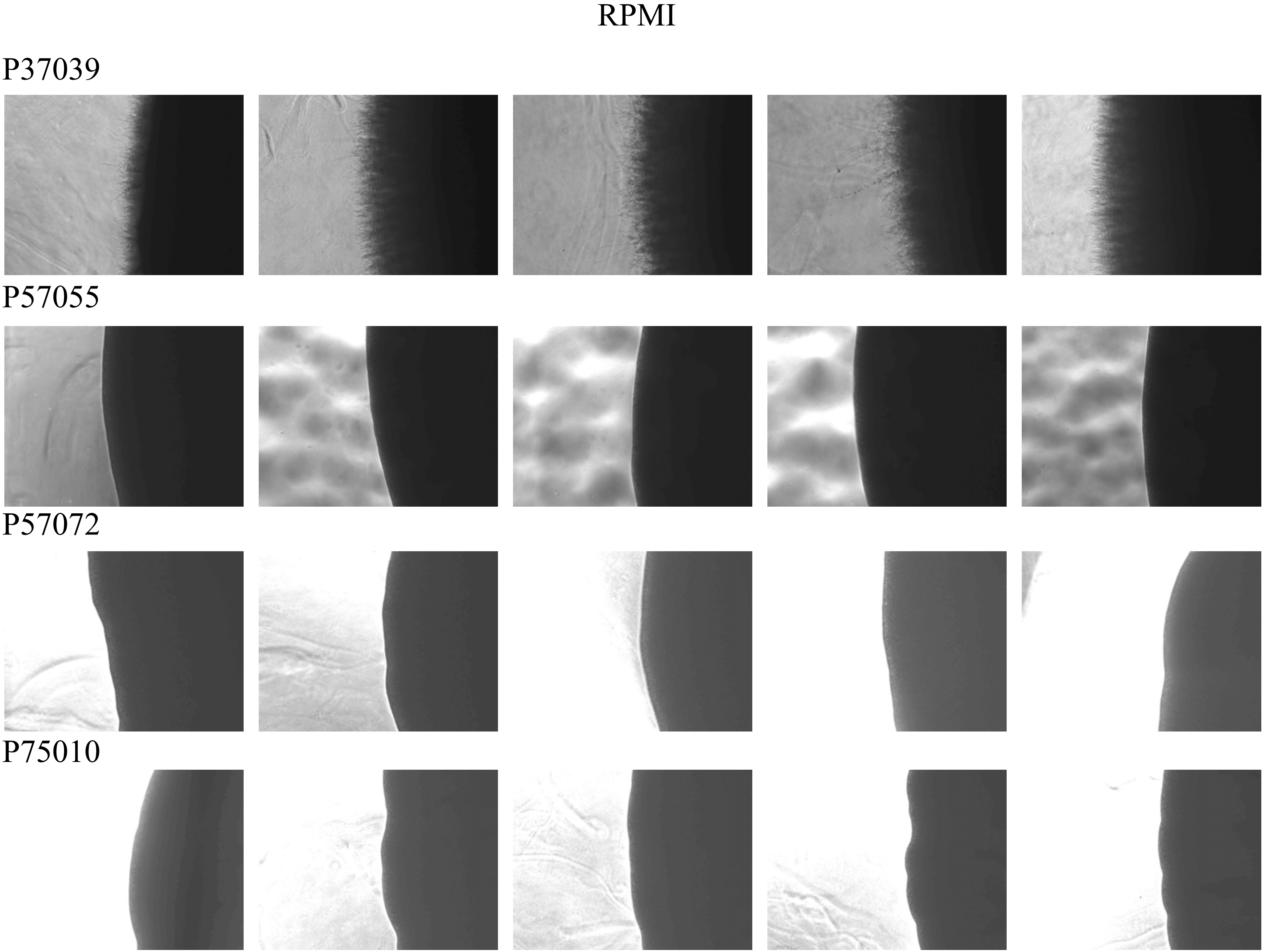
RPMI
P37039
P57055
P57072
P75010

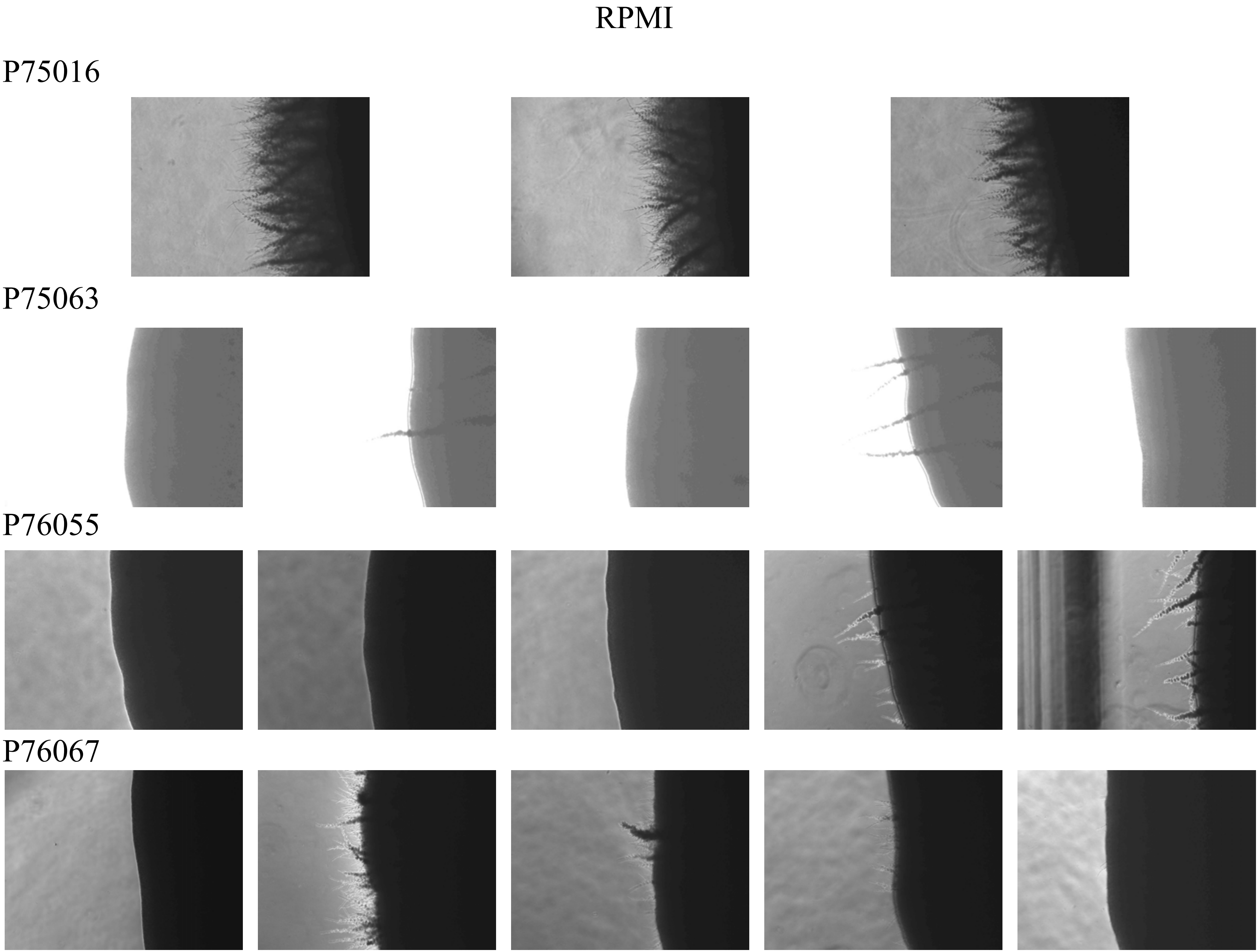
RPMI
P75016
P75063
P76055
P76067

RPMI

P78042

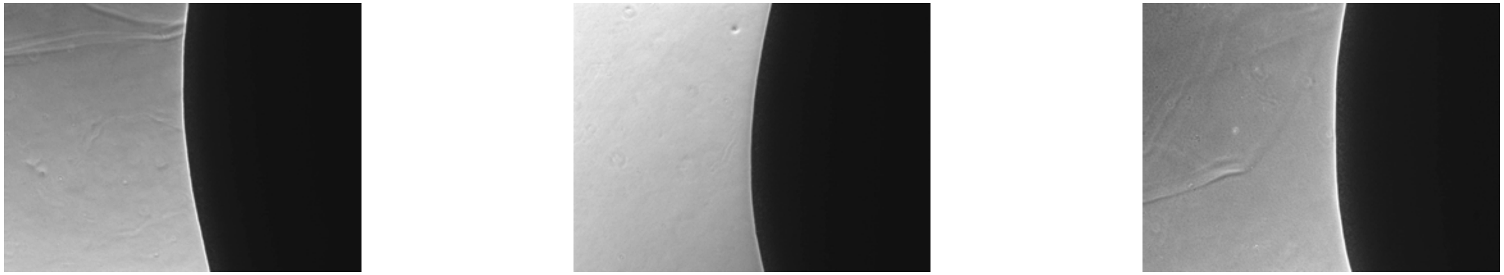

P78048

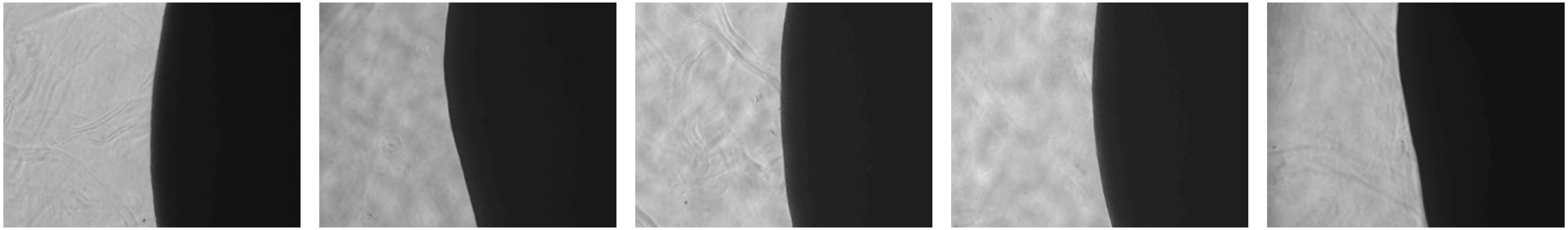

P94015

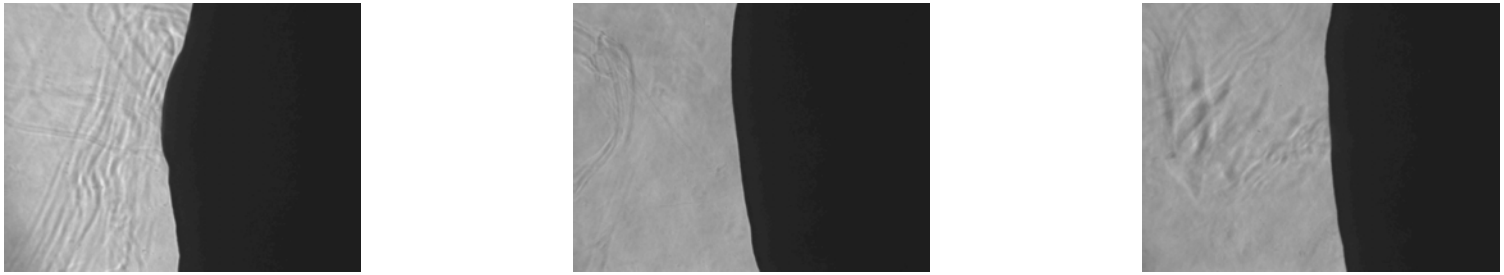

P60002

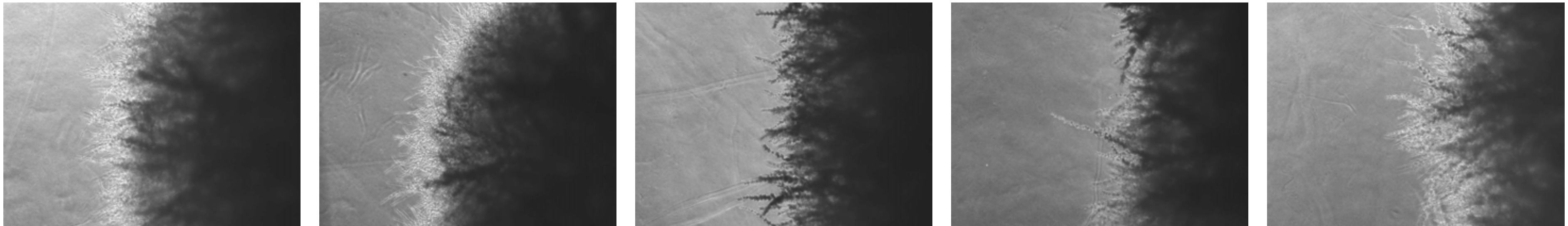

Spider

B444-12

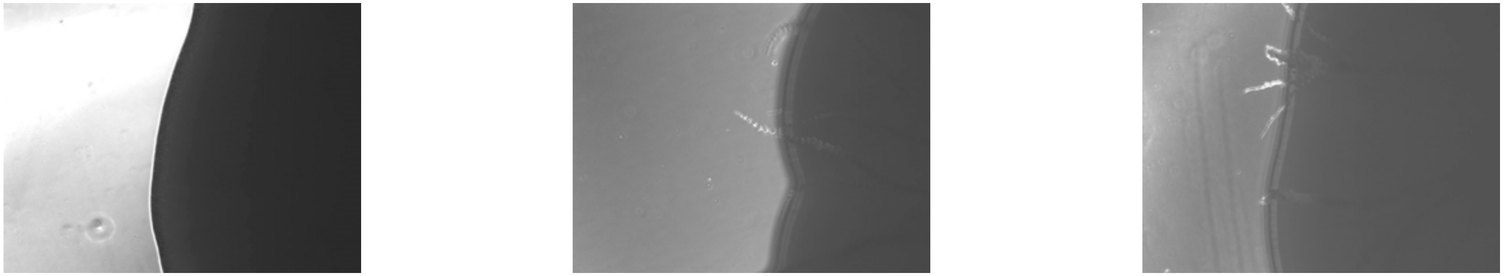

B1257-15

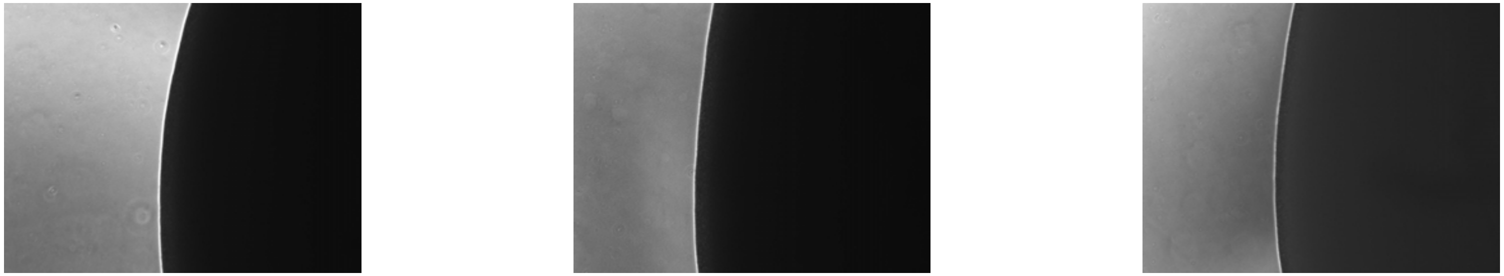

B687-15

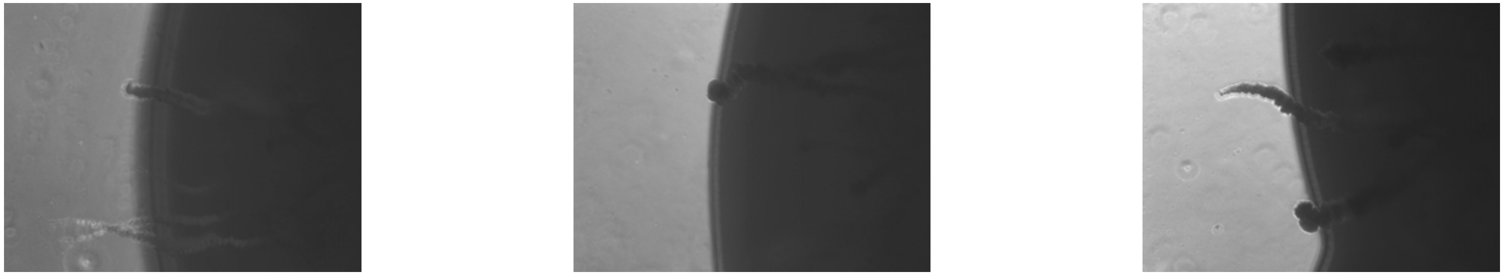

B1762-15

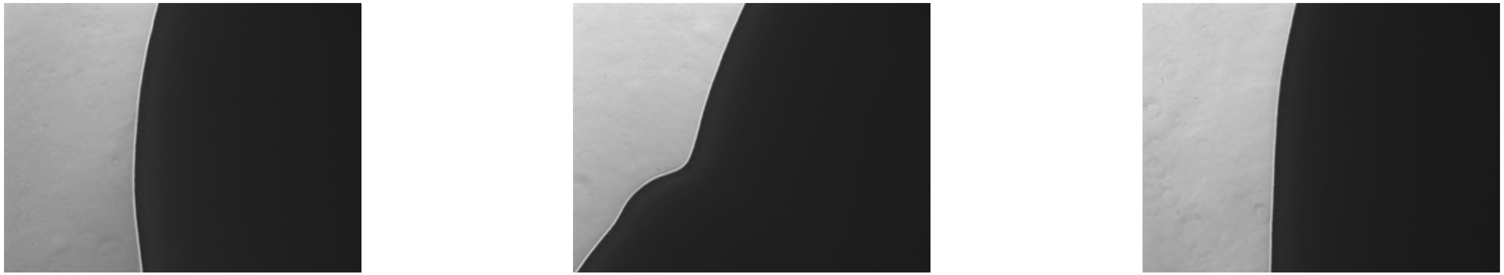

Spider

B46-15

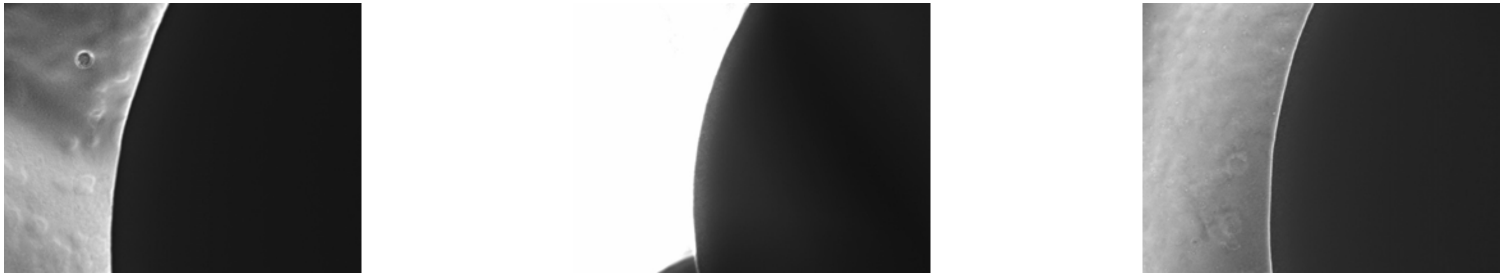

B808-15

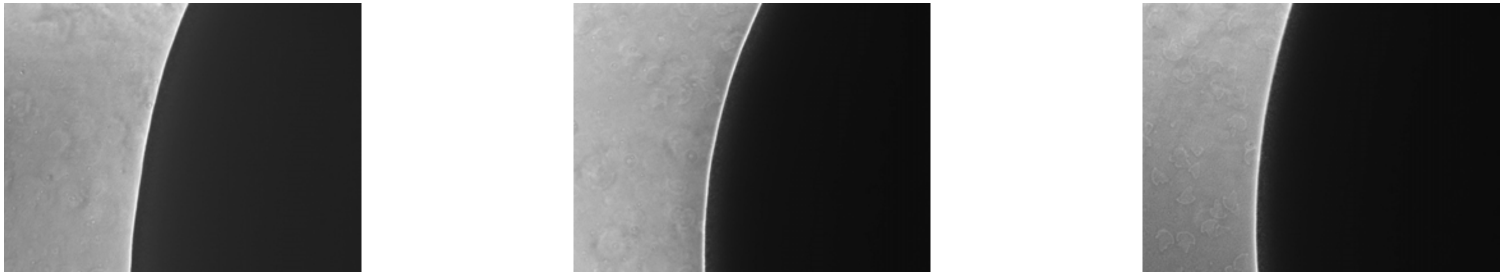

B527-15

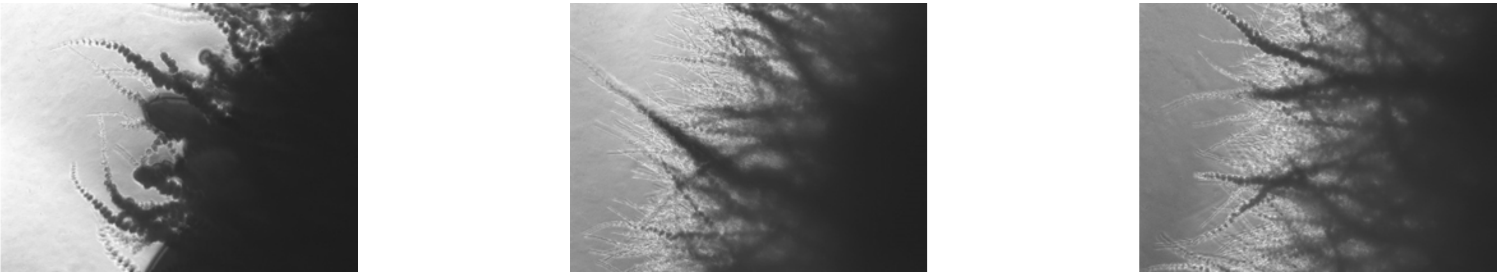

B618-15

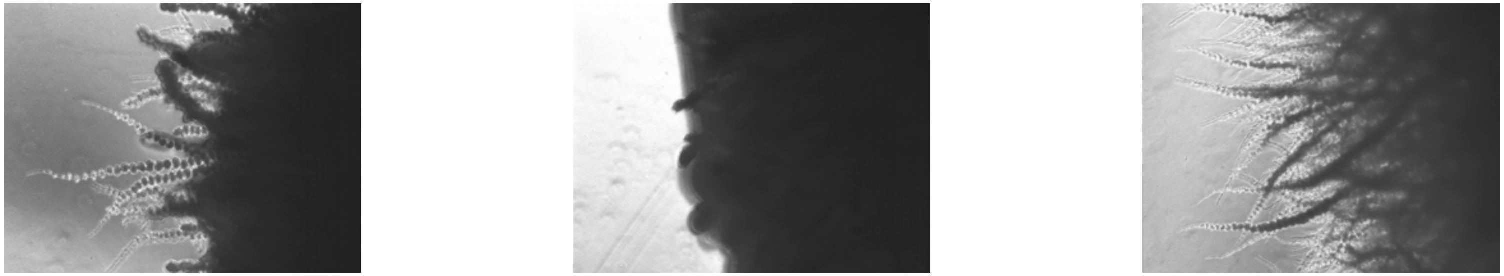

Spider

B404-15

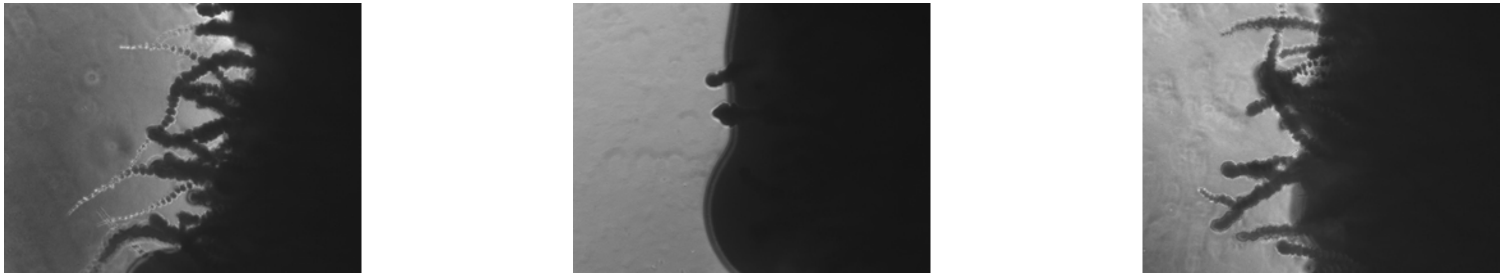

B421-15

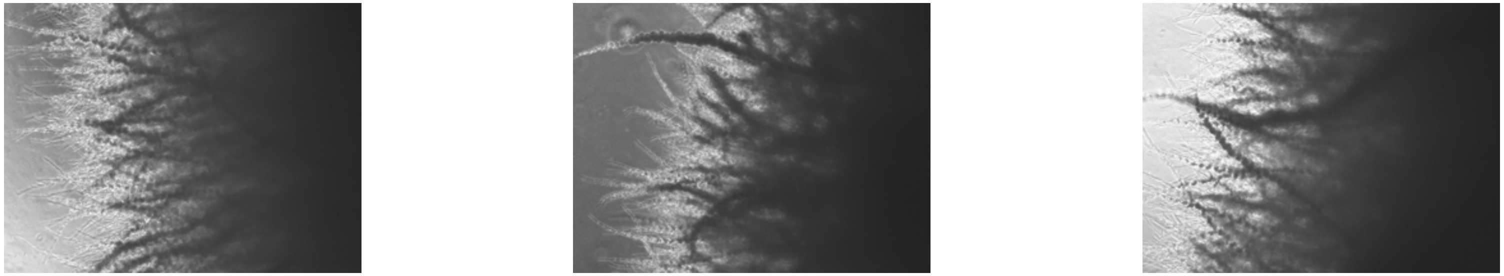

B212-12

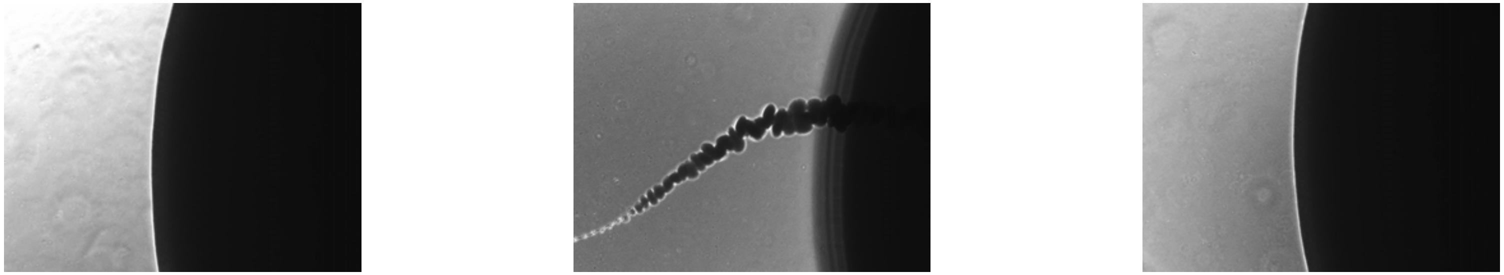

B1091-15

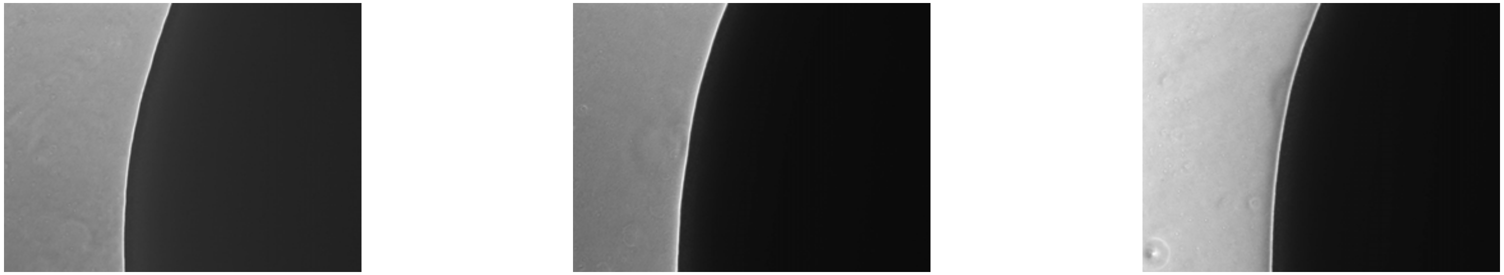

Spider

B510-12

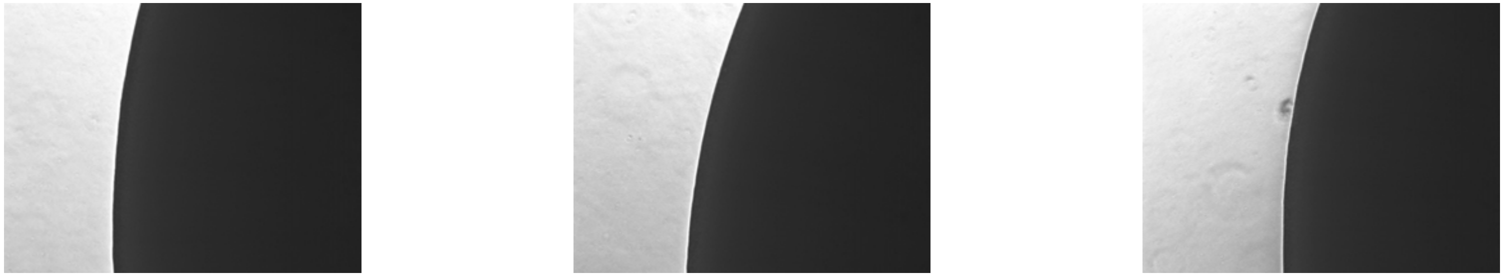

B564-15

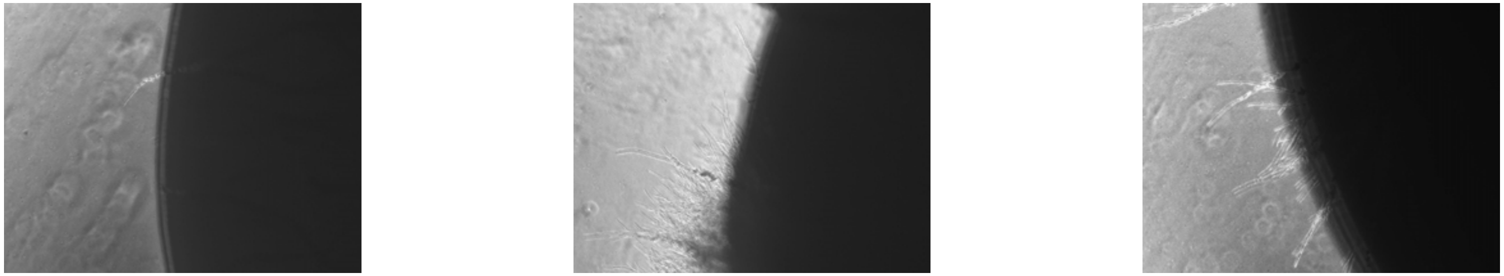

B1168-15

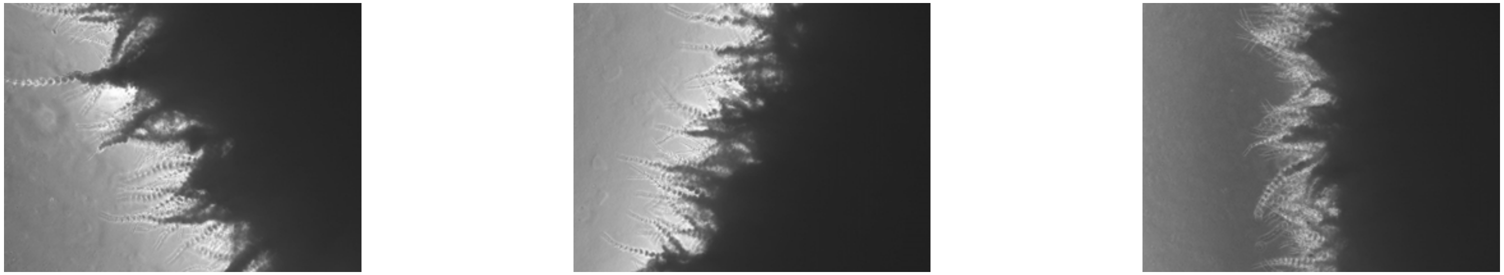

B568-15

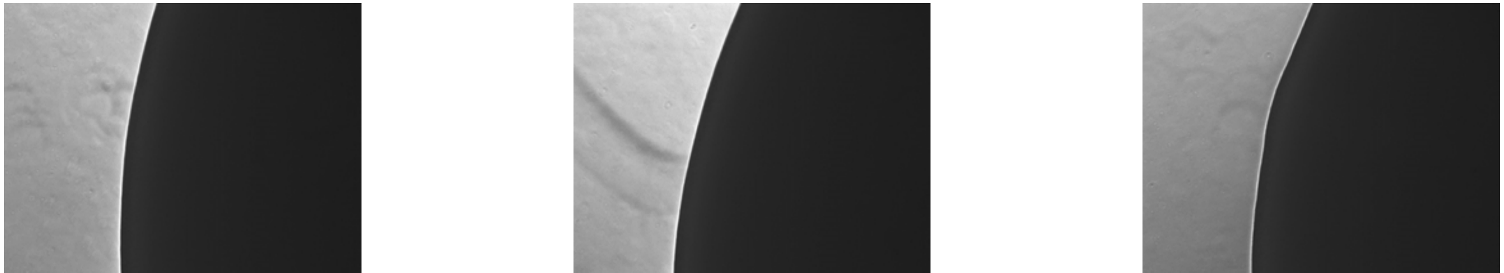

Spider

B2527-12

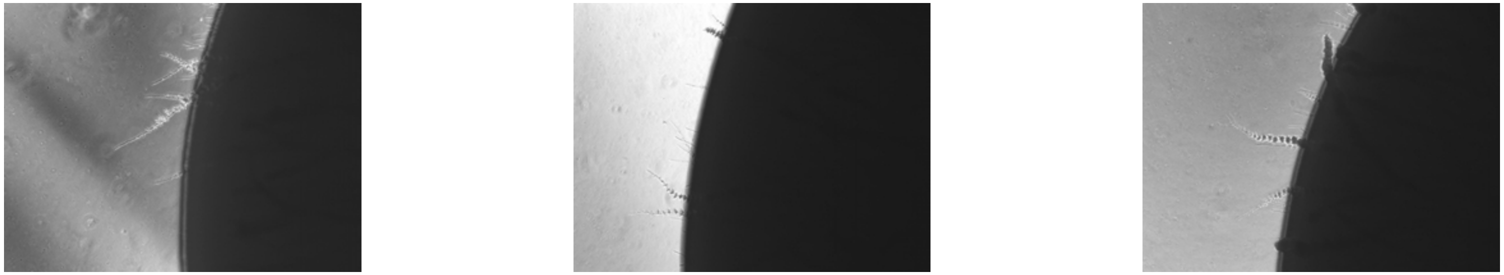

B1486-15

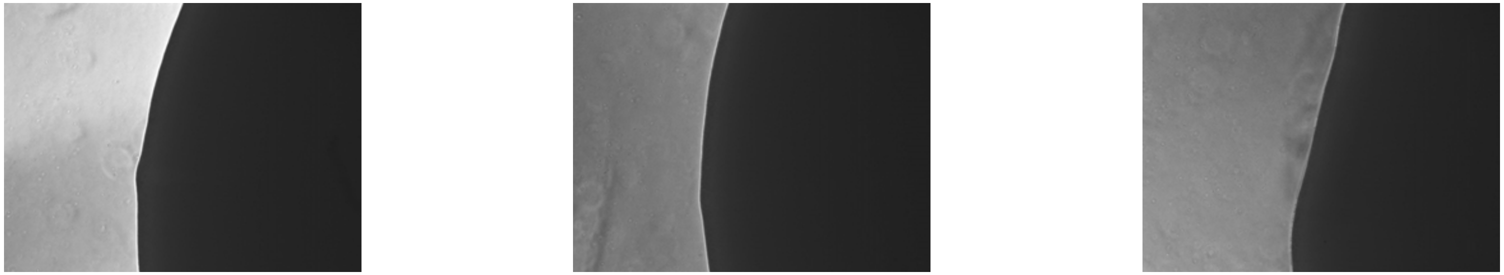

B1559-15

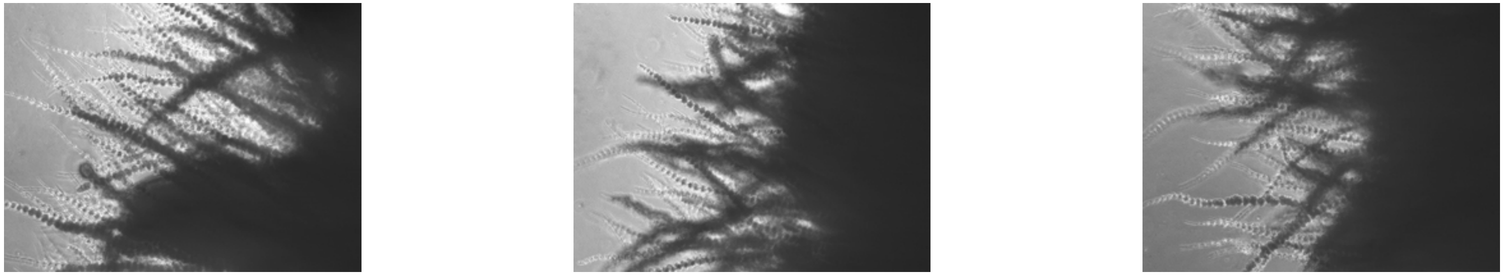

B733-15

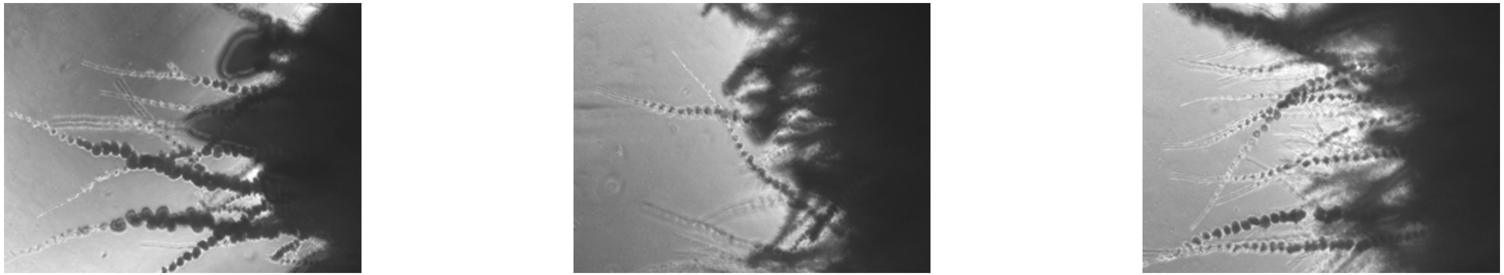

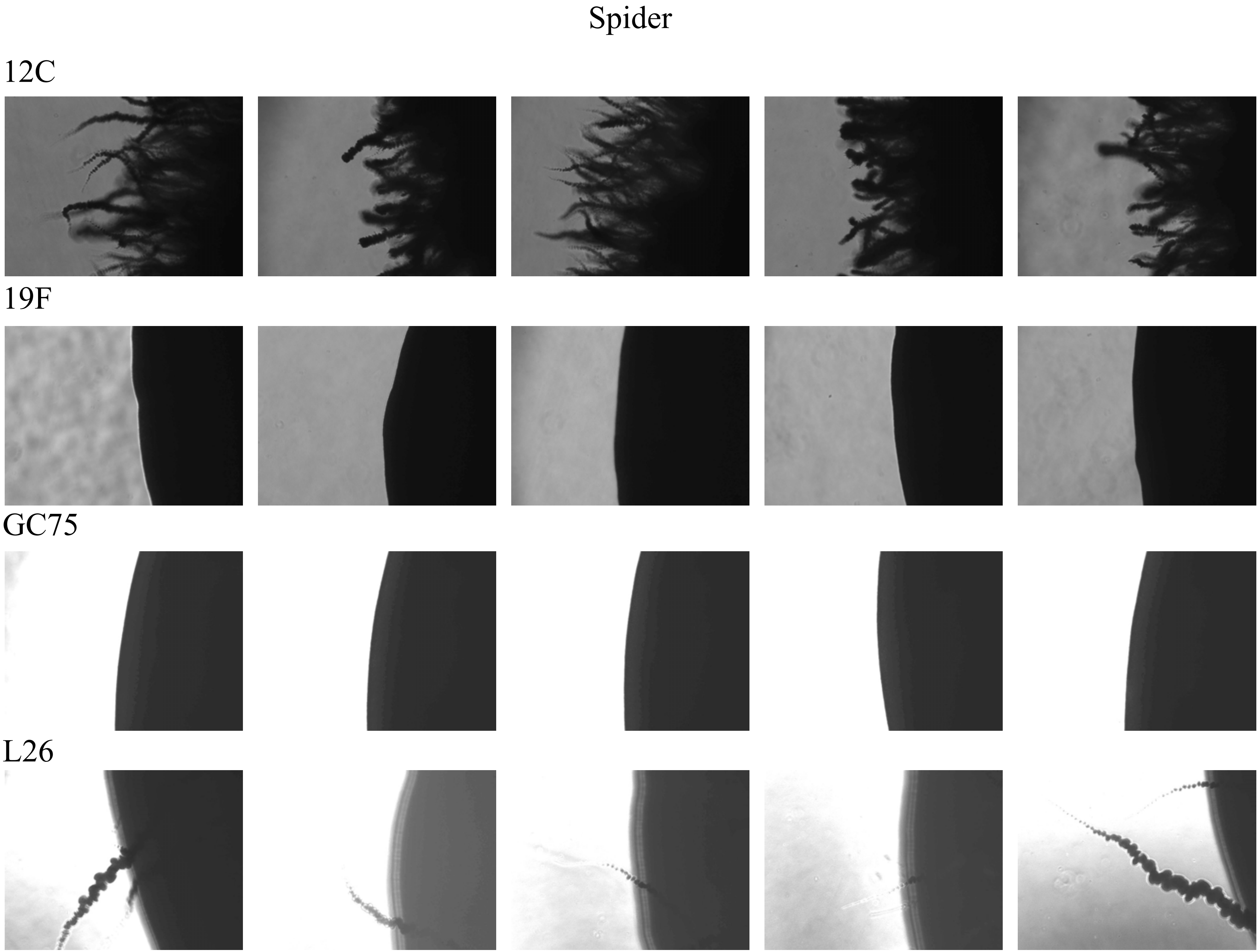
Spider
12C
19F
GC75
L26

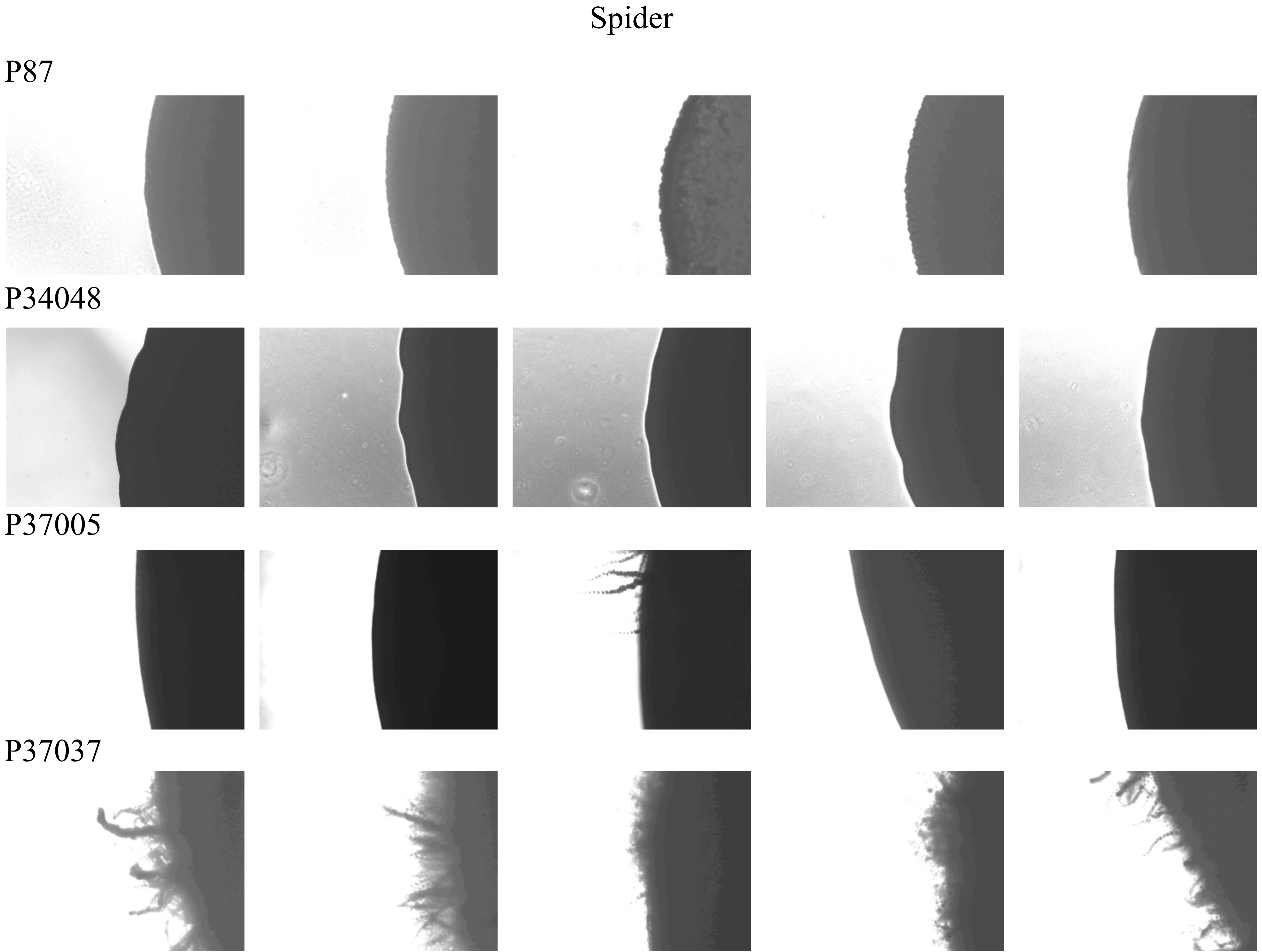
Spider
P87
P34048
P37005
P37037

Spider

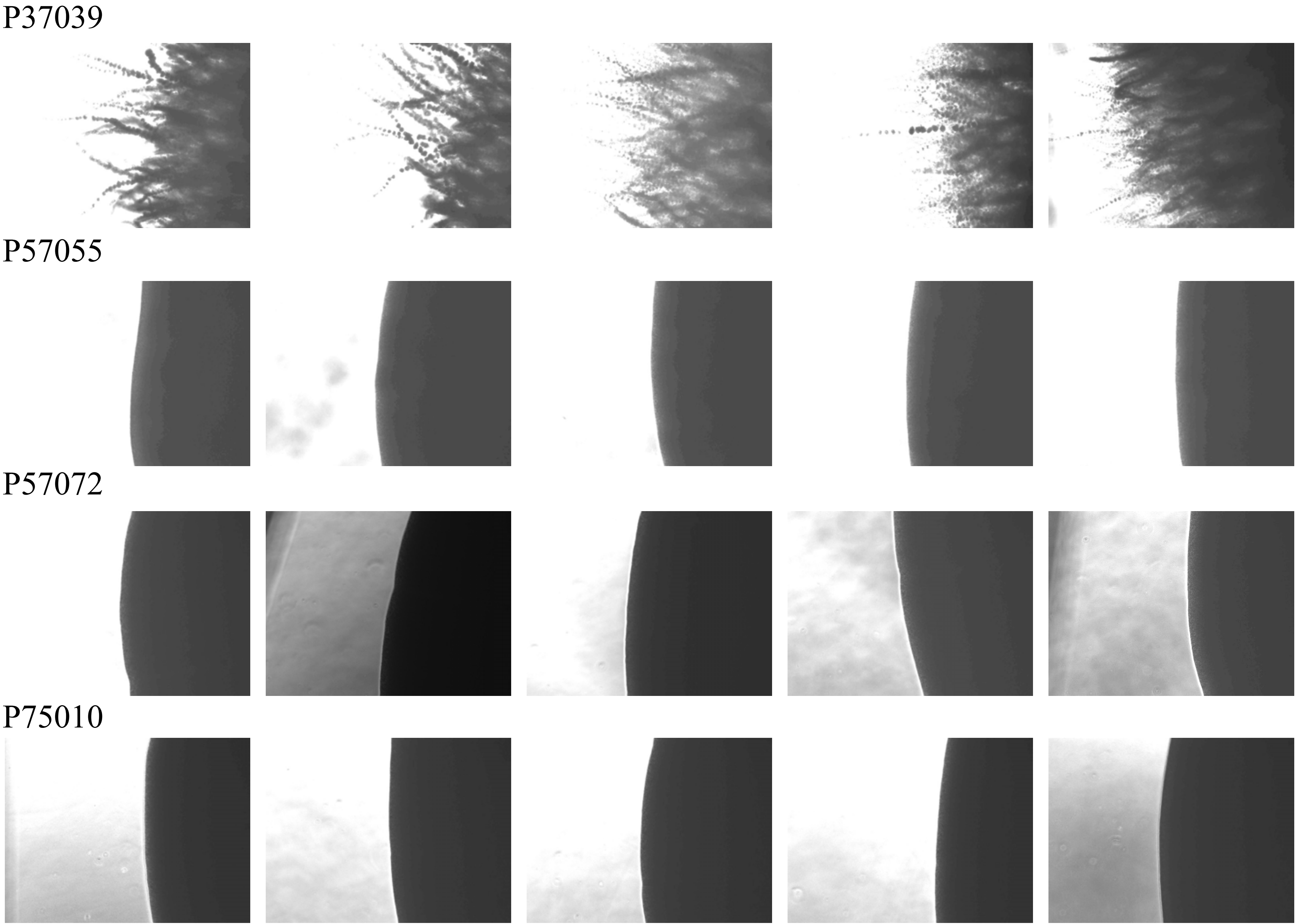

Spider

P75016

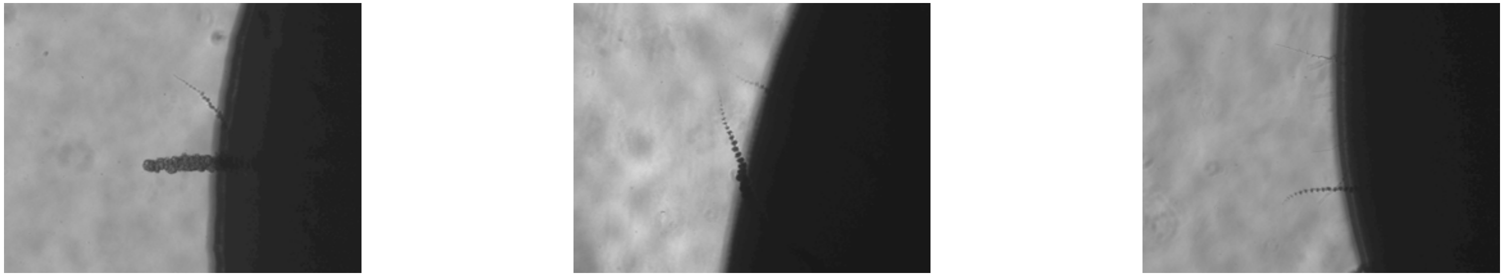

P75063

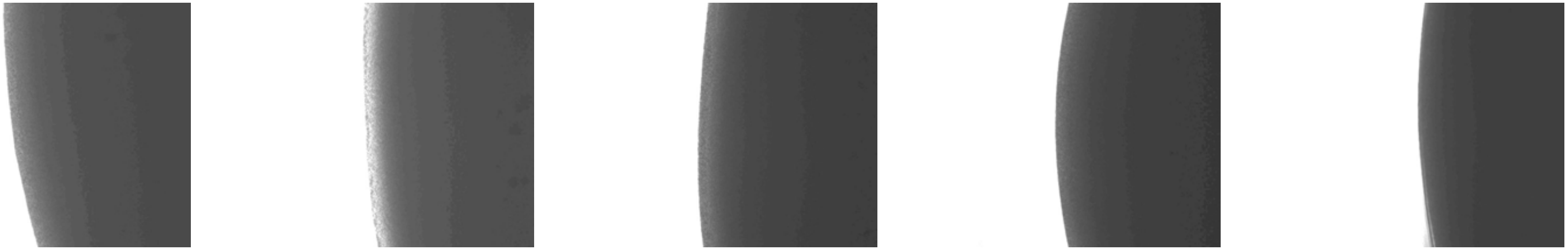

P76055

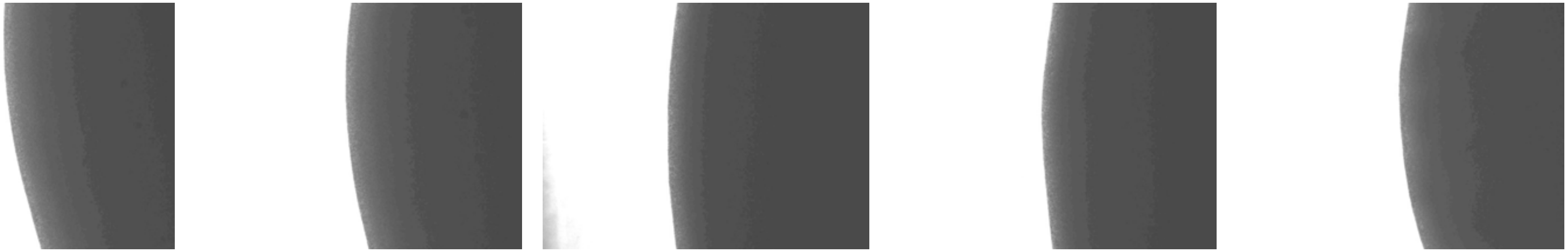

P76067

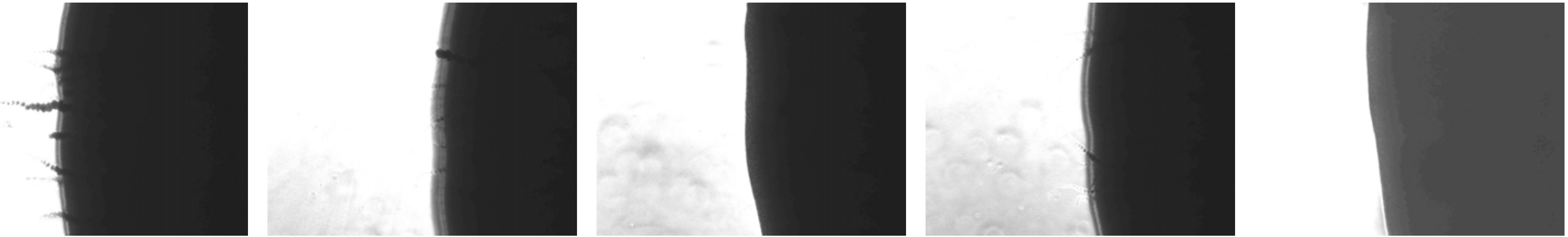

Spider

P78042

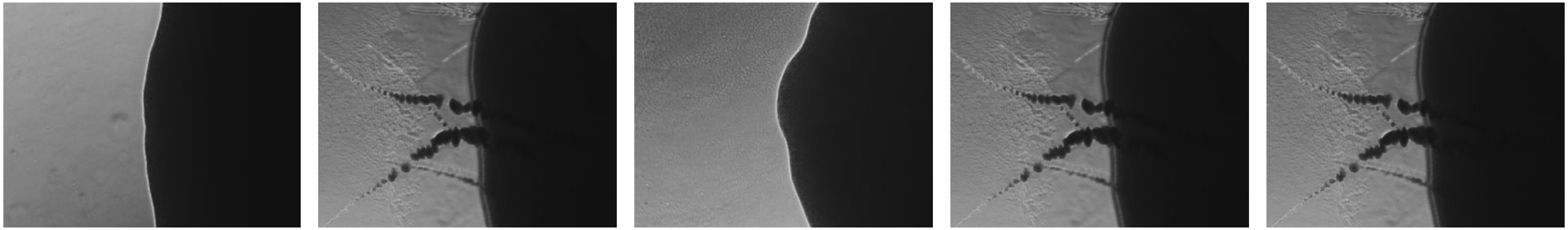

P78048

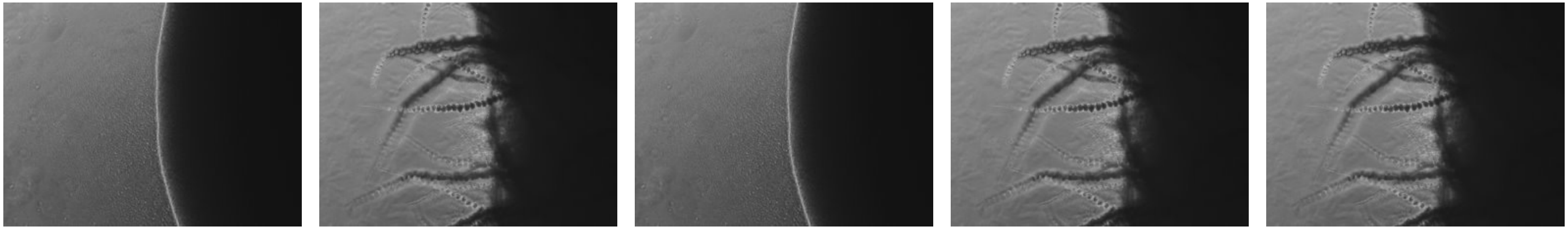

P94015

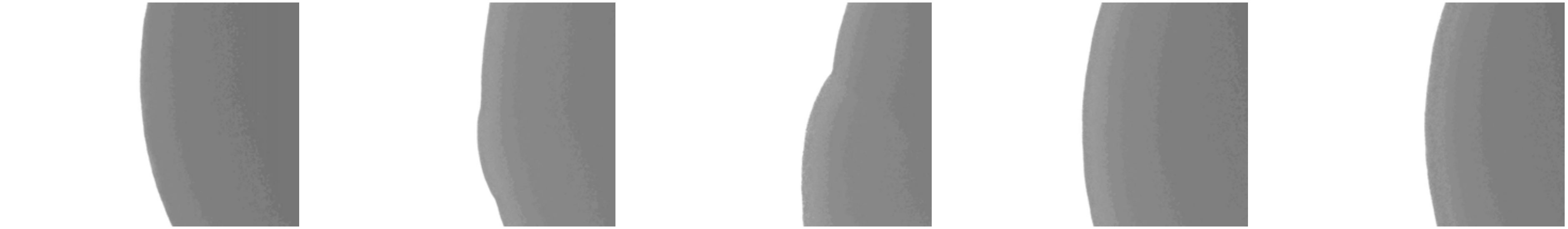

P60002

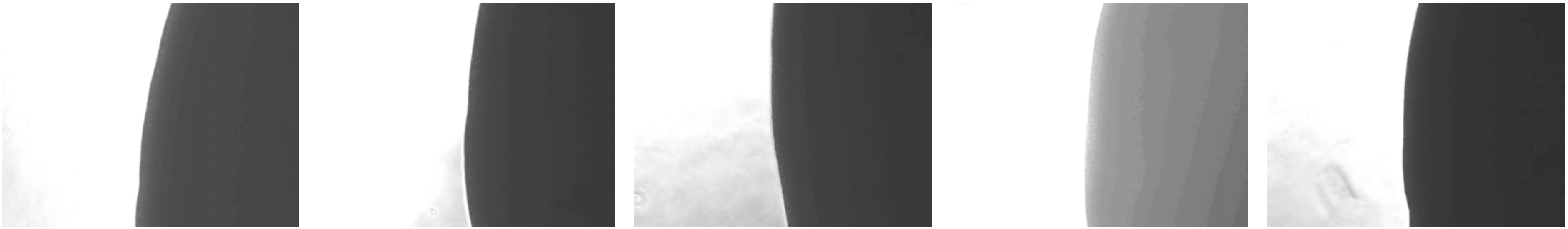

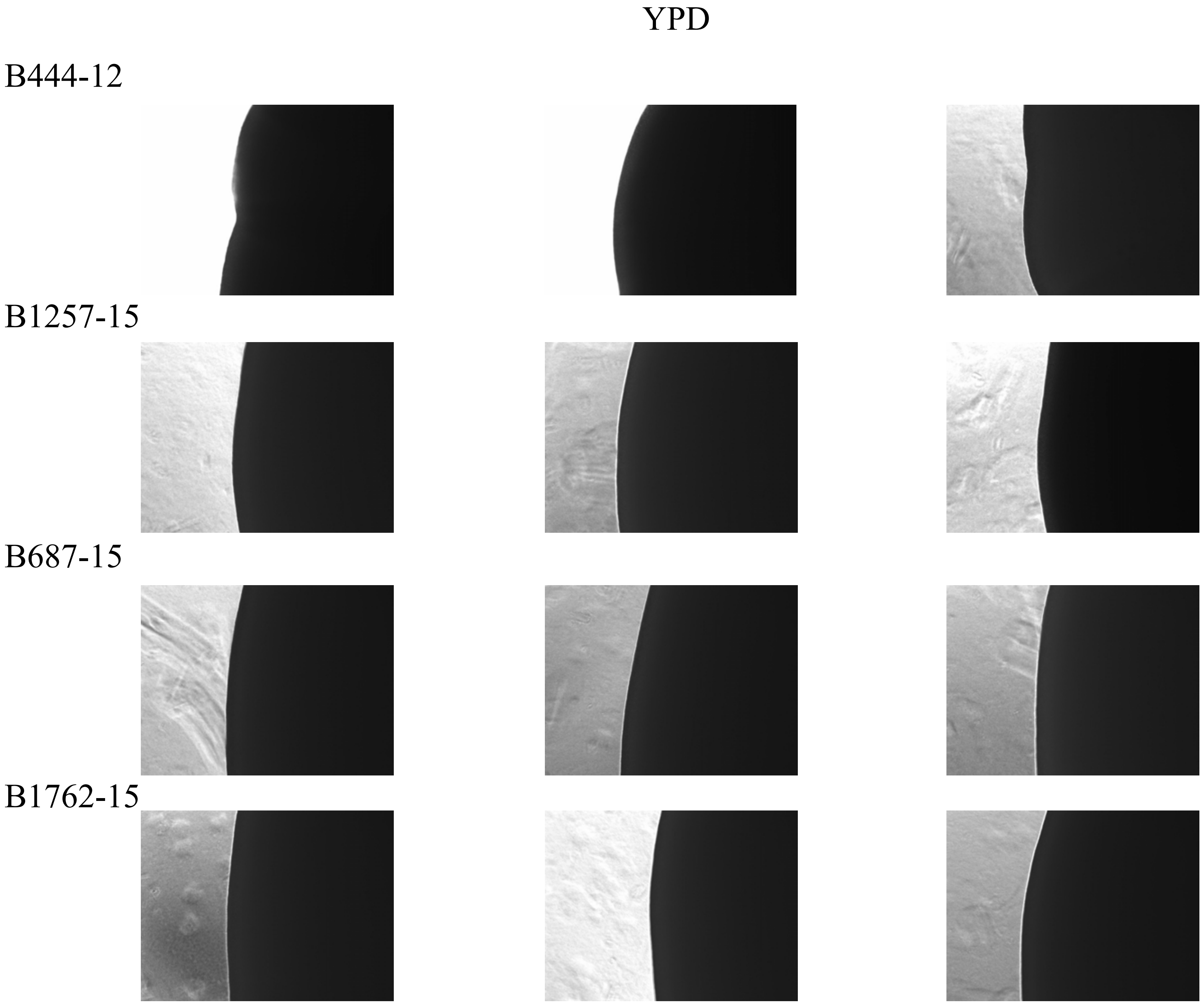
YPD
B444-12
B1257-15
B687-15
B1762-15

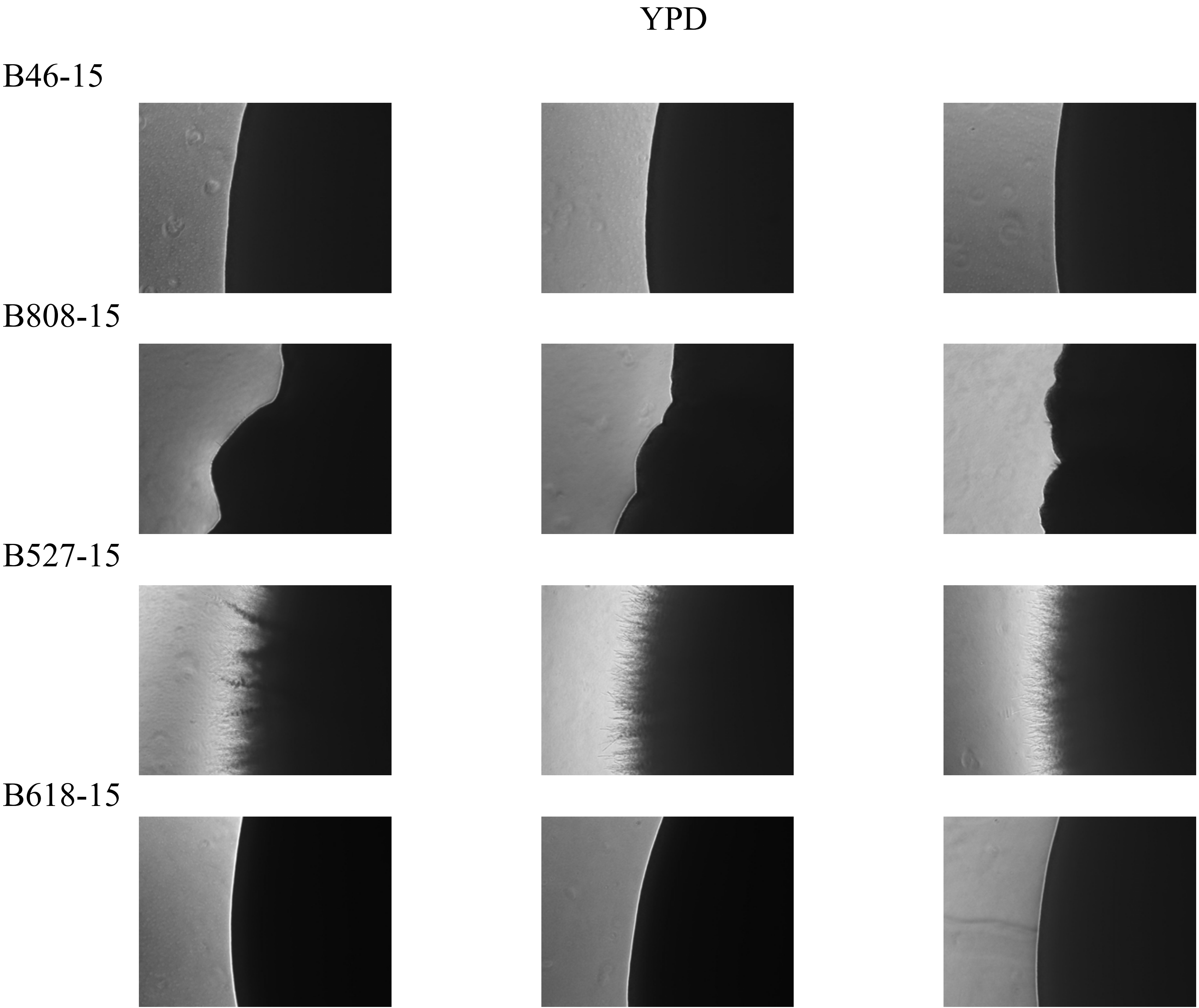
YPD
B46-15
B808-15
B527-15
B618-15

YPD

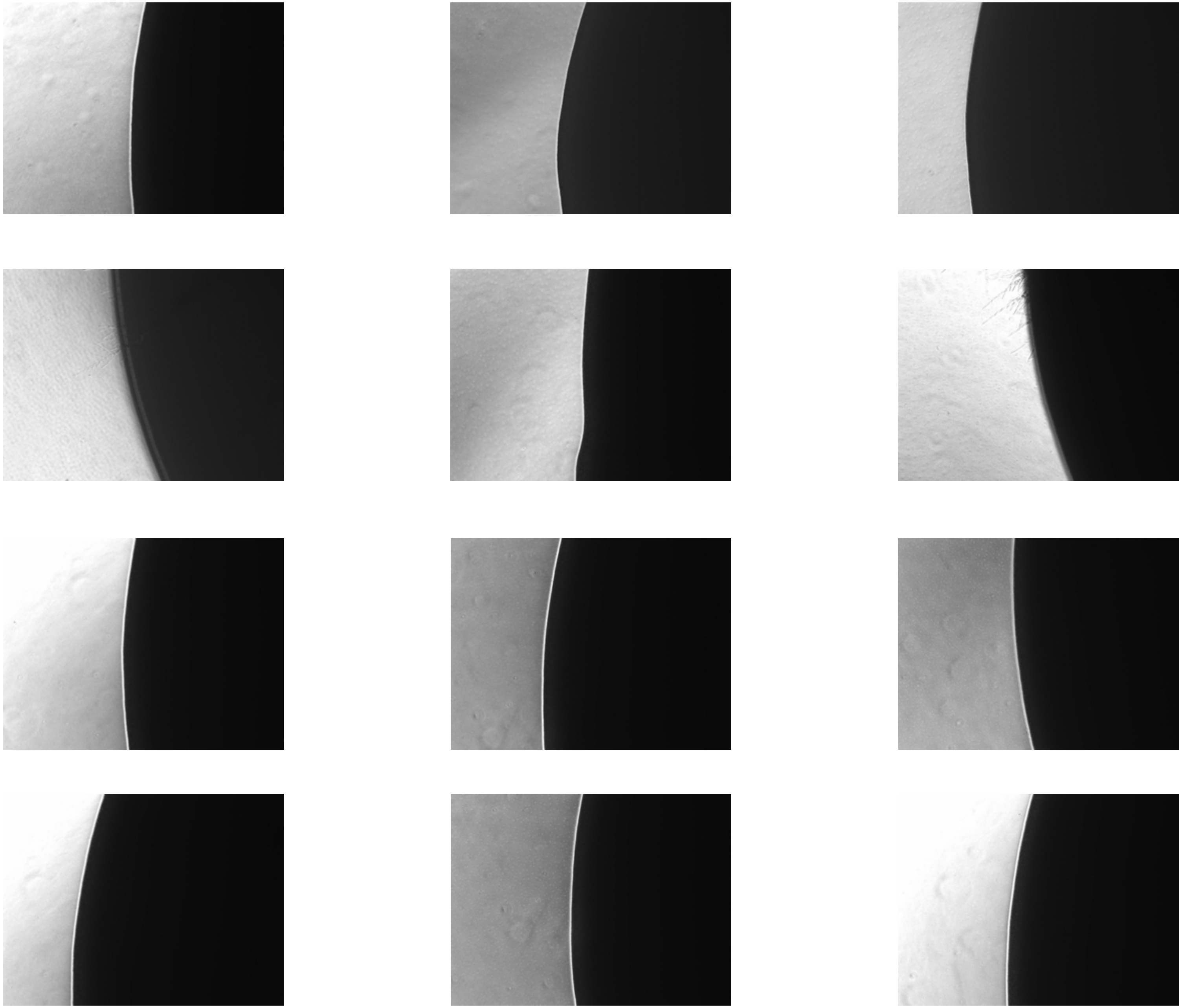

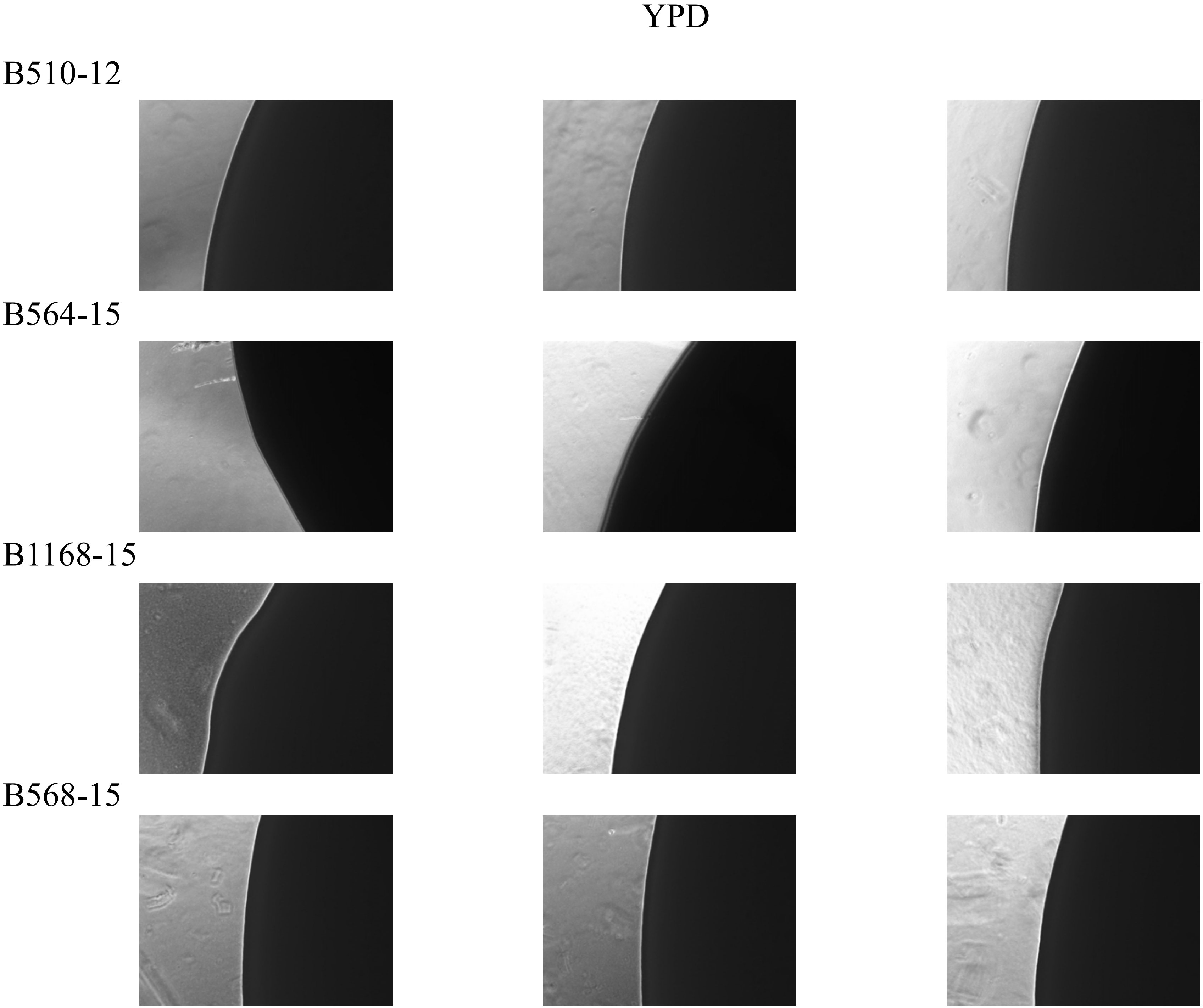
YPD
B510-12
B564-15
B1168-15
B568-15

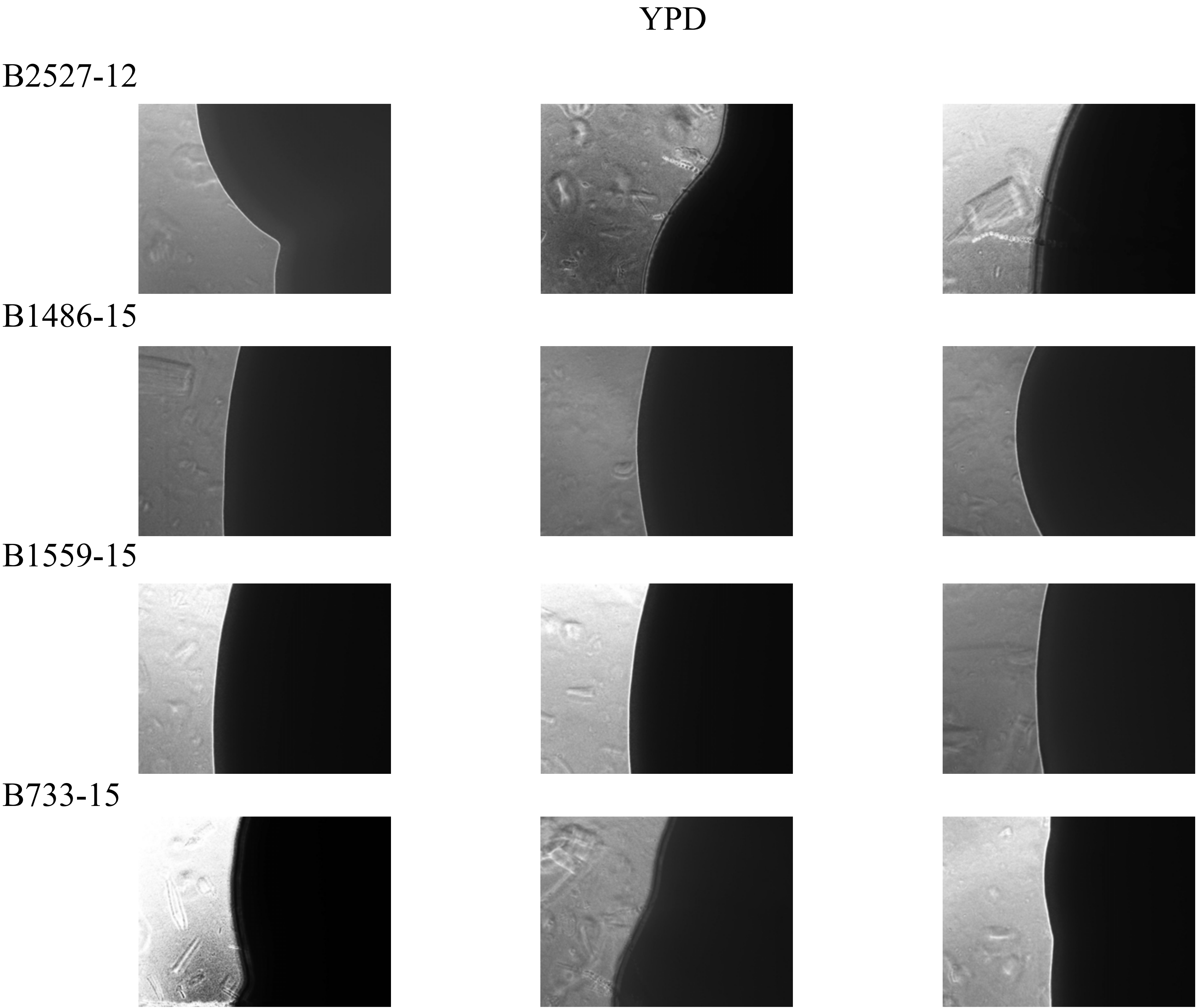
YPD
B2527-12
B1486-15
B1559-15
B733-15

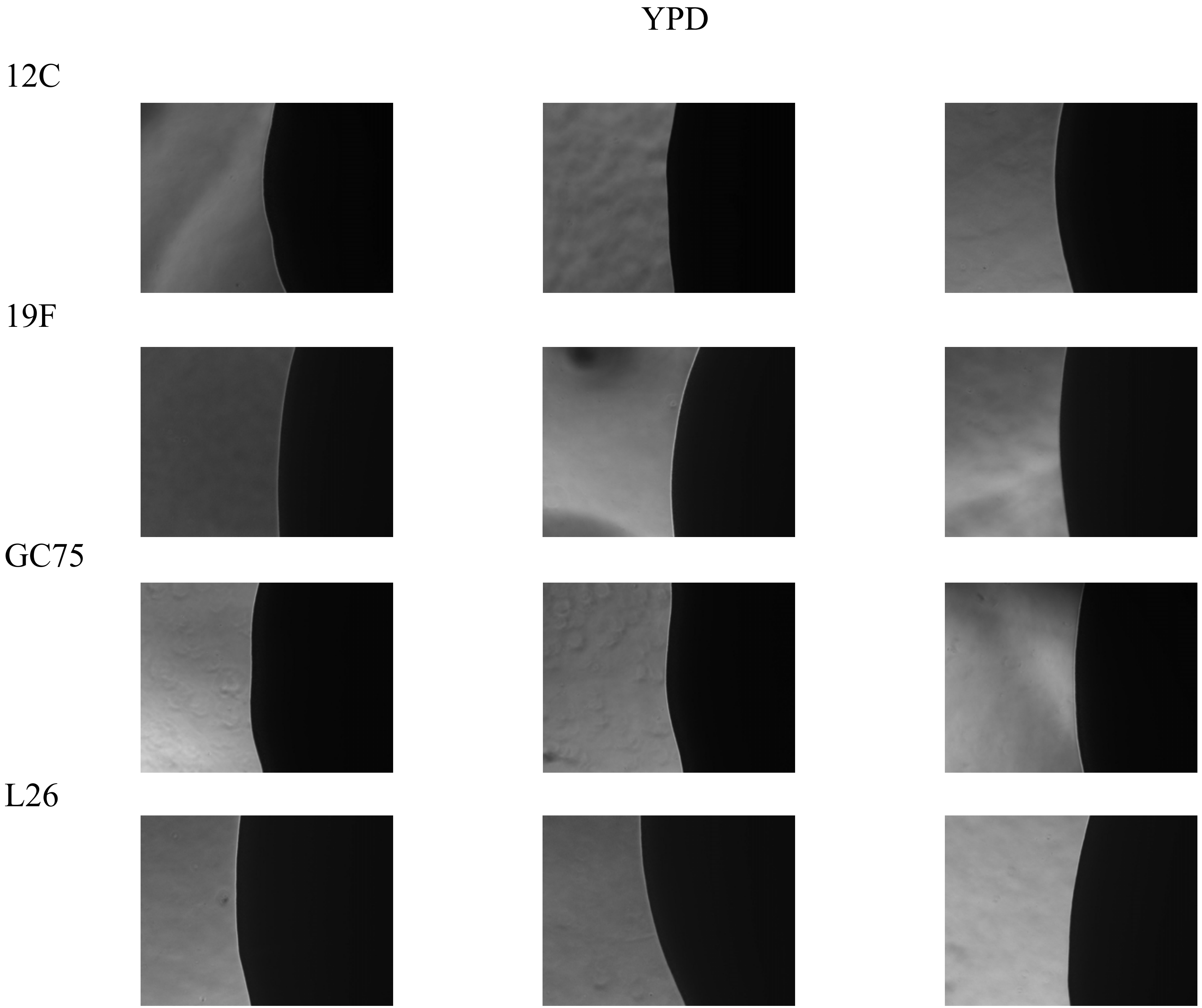
YPD
12C
19F
GC75
L26

YPD

P87

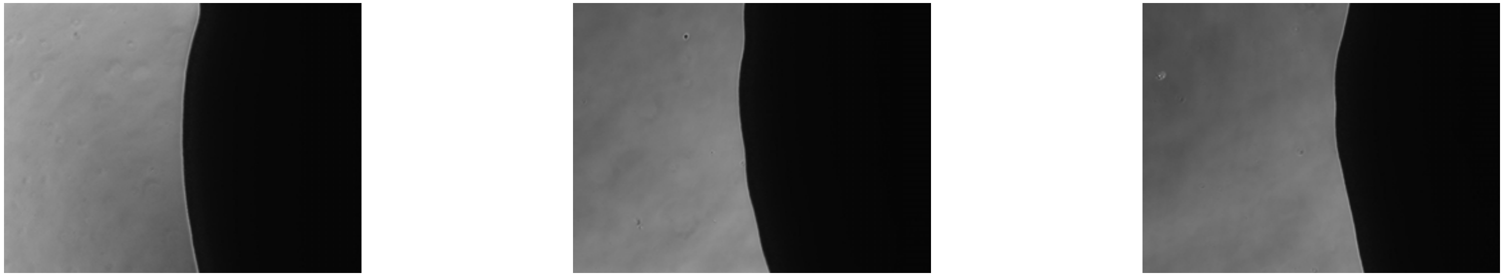

P34048

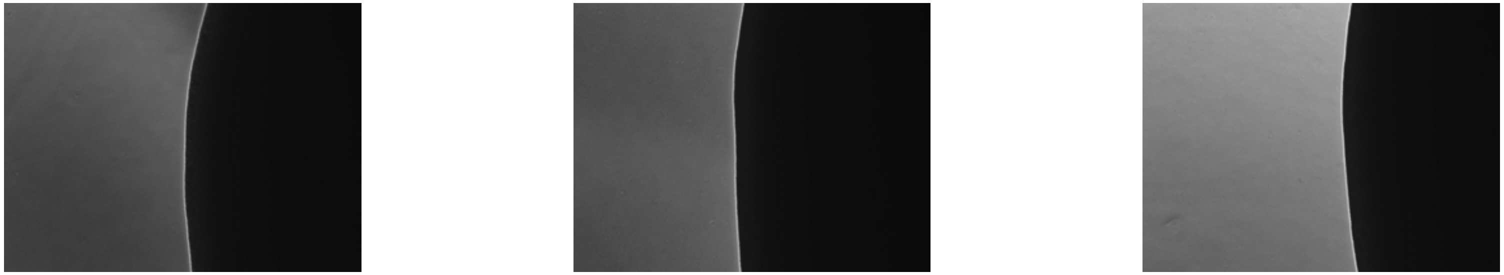

P37005

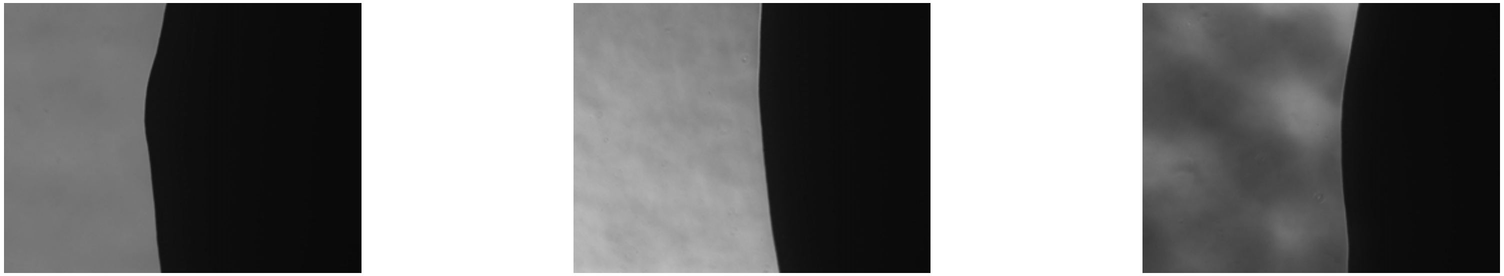

P37037

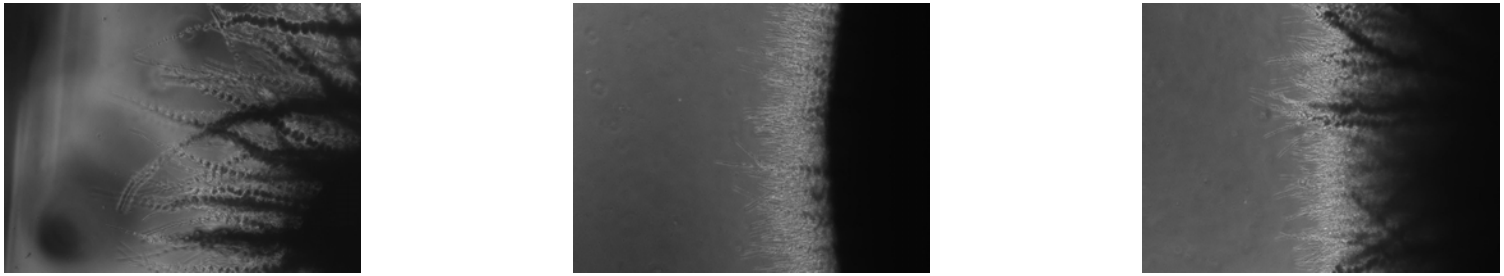

YPD

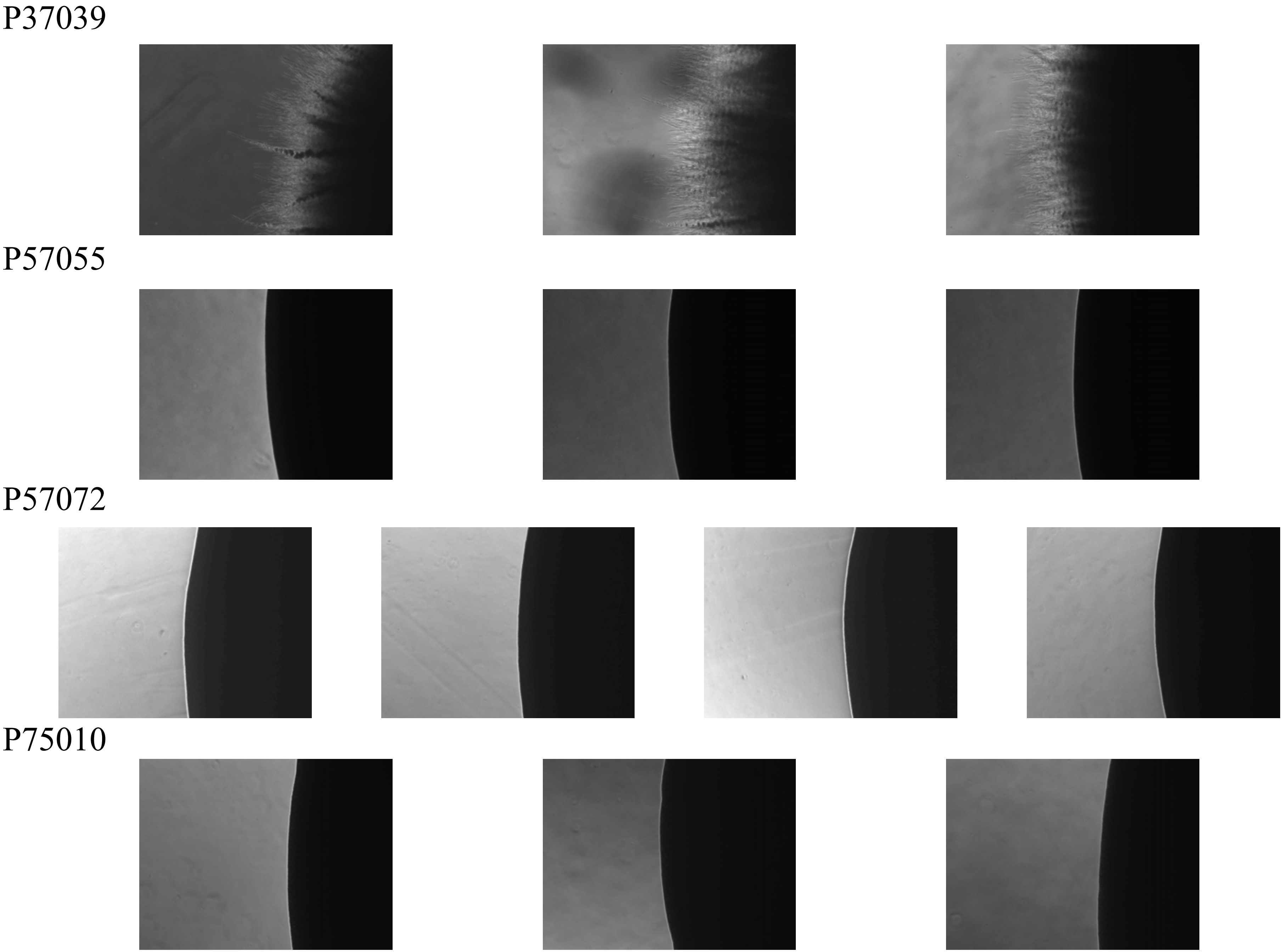

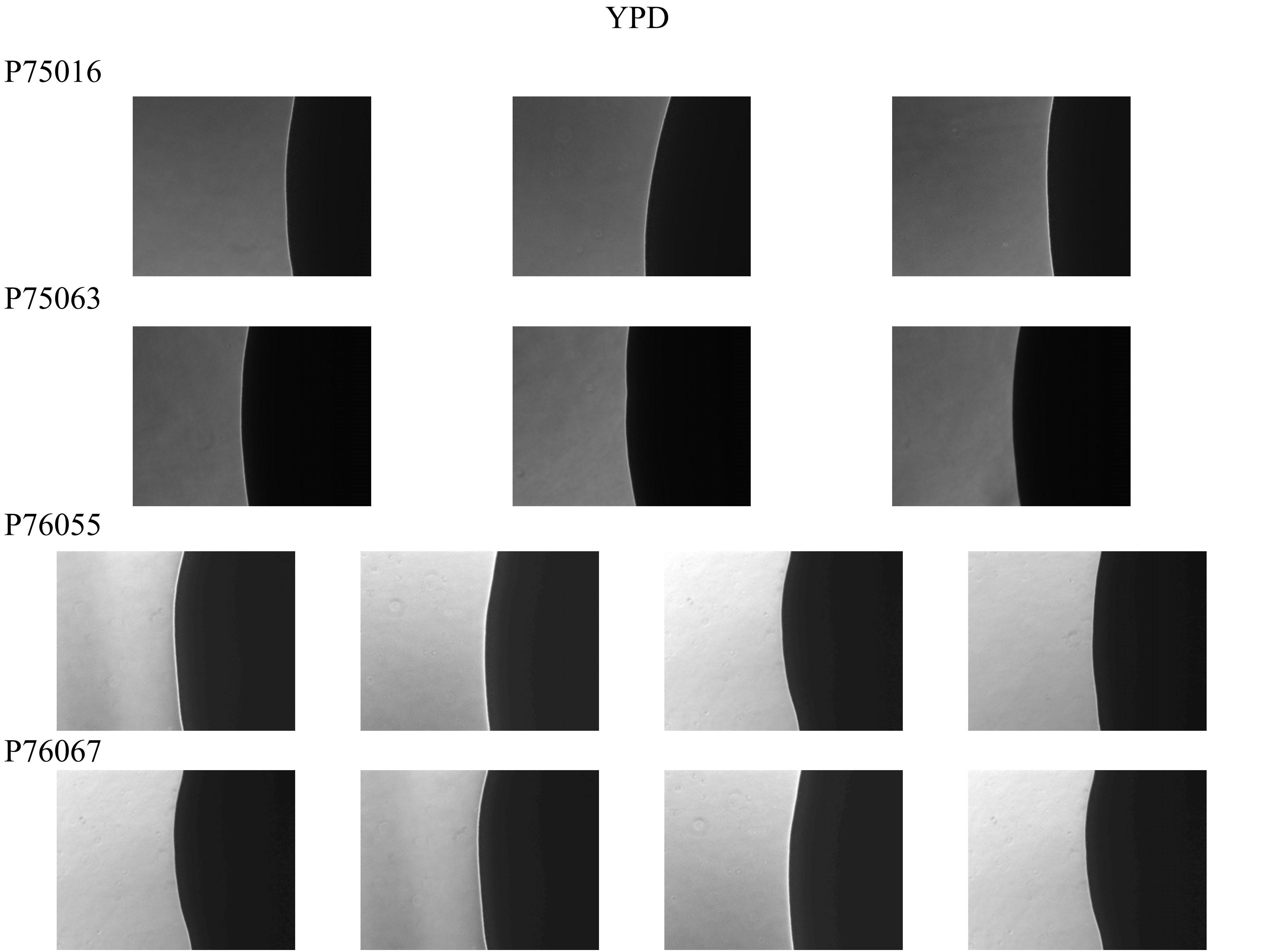
YPD
P75016
P75063
P76055
P76067

YPD

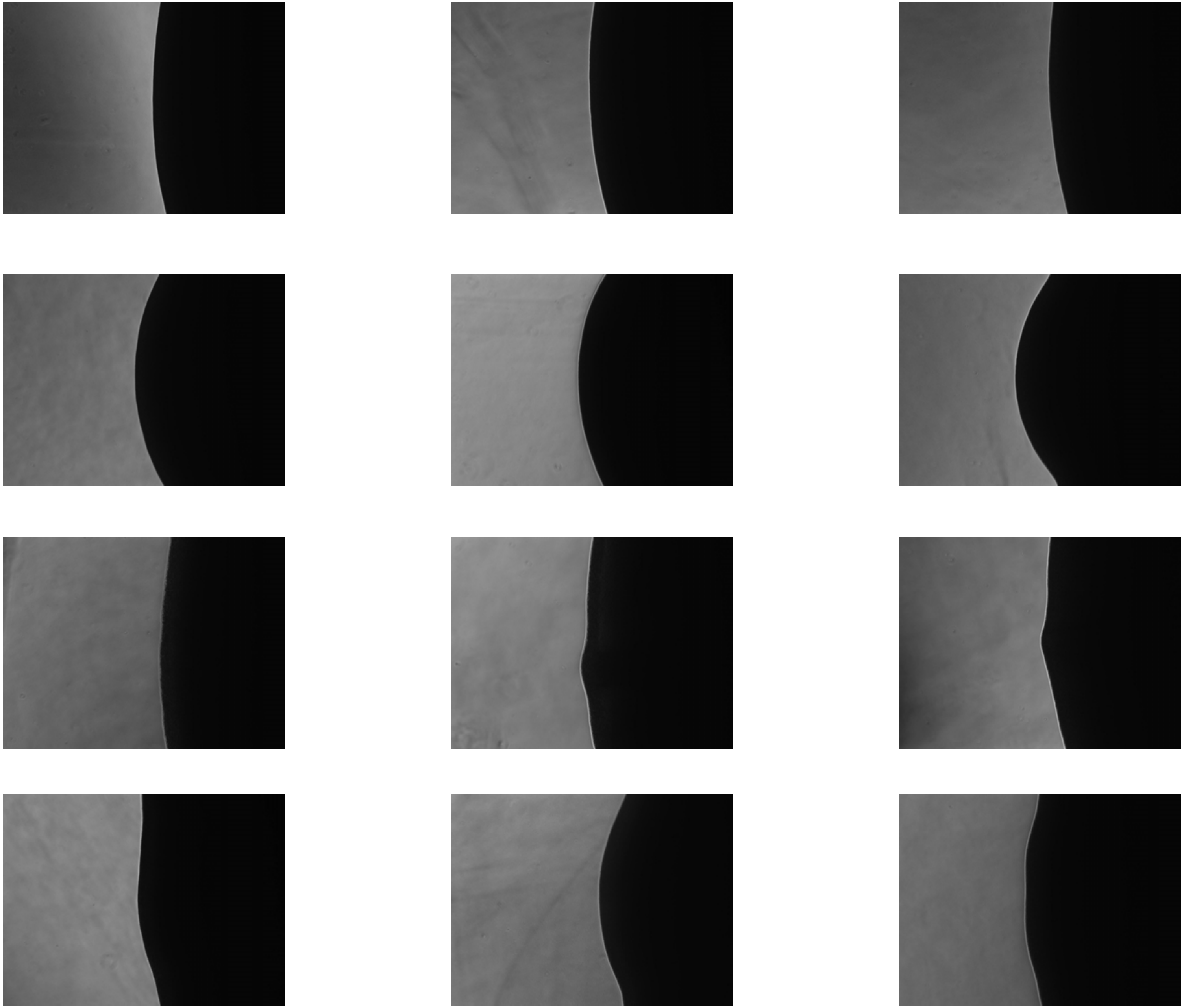

Supplement: Supplementary Figure 2 — Clinical strain filamentation in the standard solid media assay. Cells of the indicated strains were tested for filamentation in standard and shortened solid filamentation assays in FBS, Lee’s, RPMI, or spider liquid media. Cells were grown overnight at 30˚C with shaking, washed, and then spotted on inducing media agar plates and grown at 37˚C. Cells were also spotted on YPD agar media and grown at 30˚C. Colony edged were imaged after 4-5 days of incubation. [file DataSheet_4.pdf]
