# Supplementary material for: Significant variation of filamentation phenotypes in clinical Candida albicans strains
Source: Front Cell Infect Microbiol. 2023 Oct 20;13:1207083. doi: 10.3389/fcimb.2023.1207083 (PMC10623444; doi:10.3389/fcimb.2023.1207083)

Figure S3. Short filamentation assays FBS

B444-12

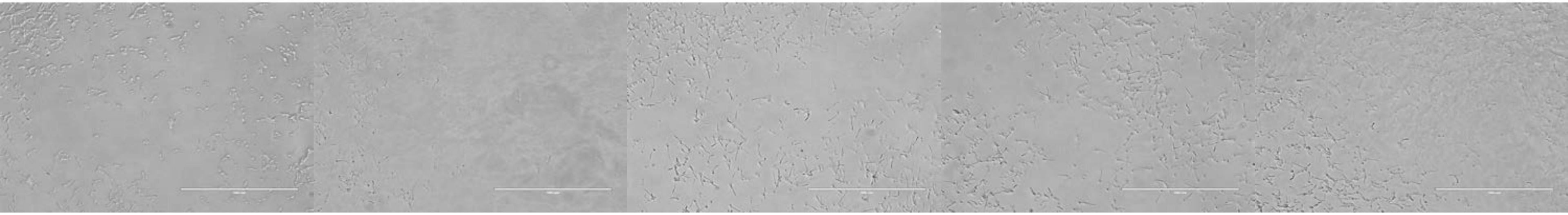

B1257-15

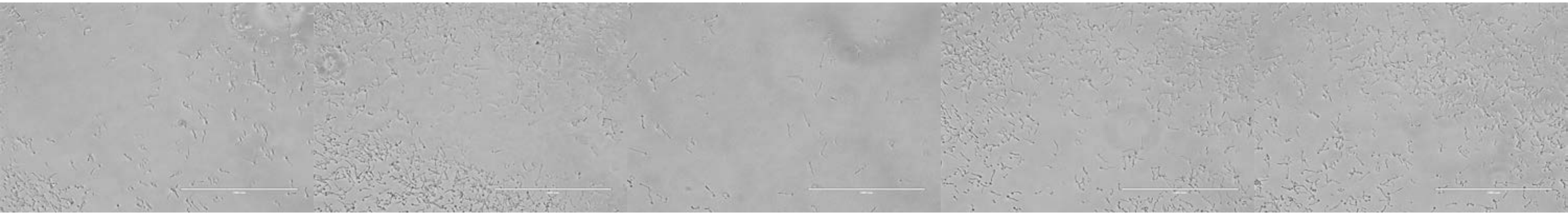

B687-15

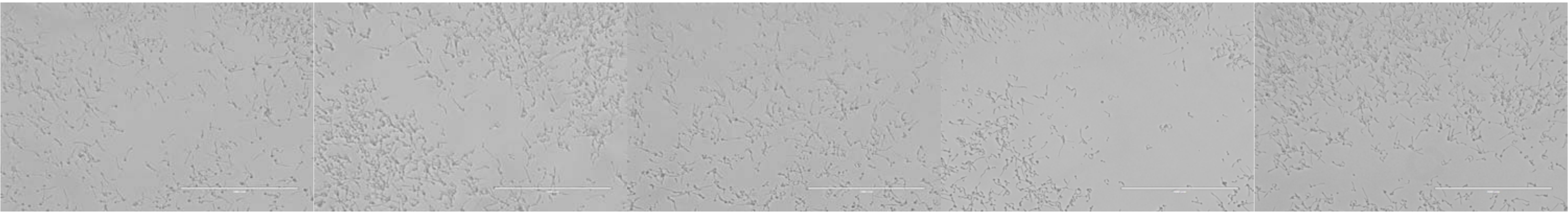

B1762-15

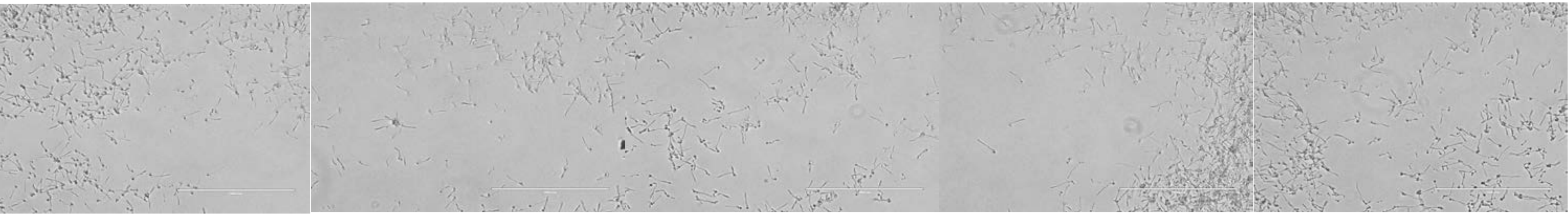

FBS

B46-15

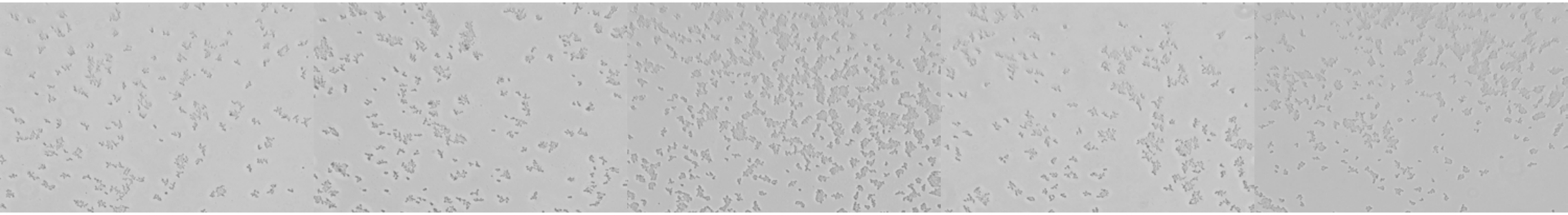

B808-15

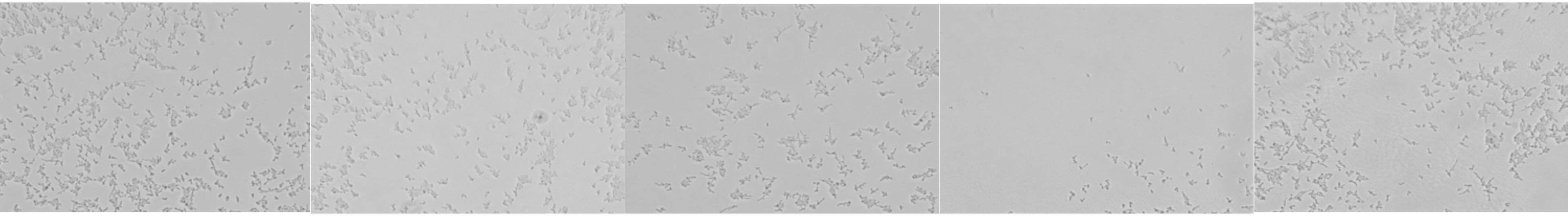

B527-15

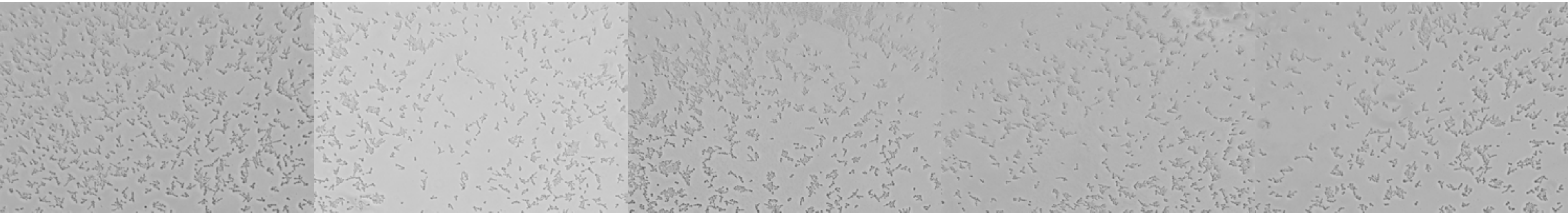

B618-15

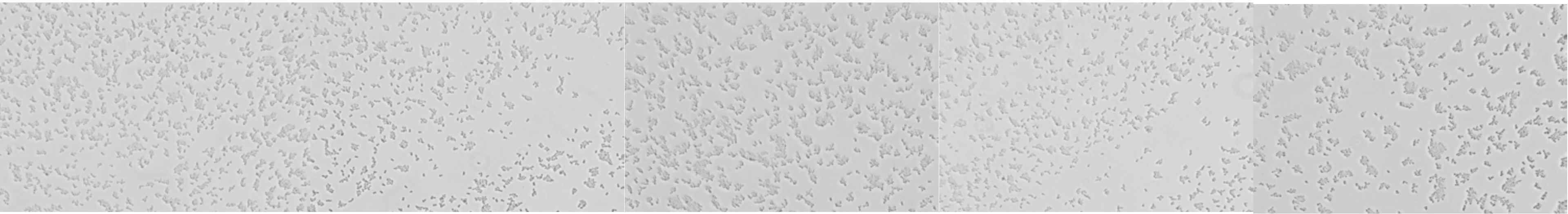

FBS

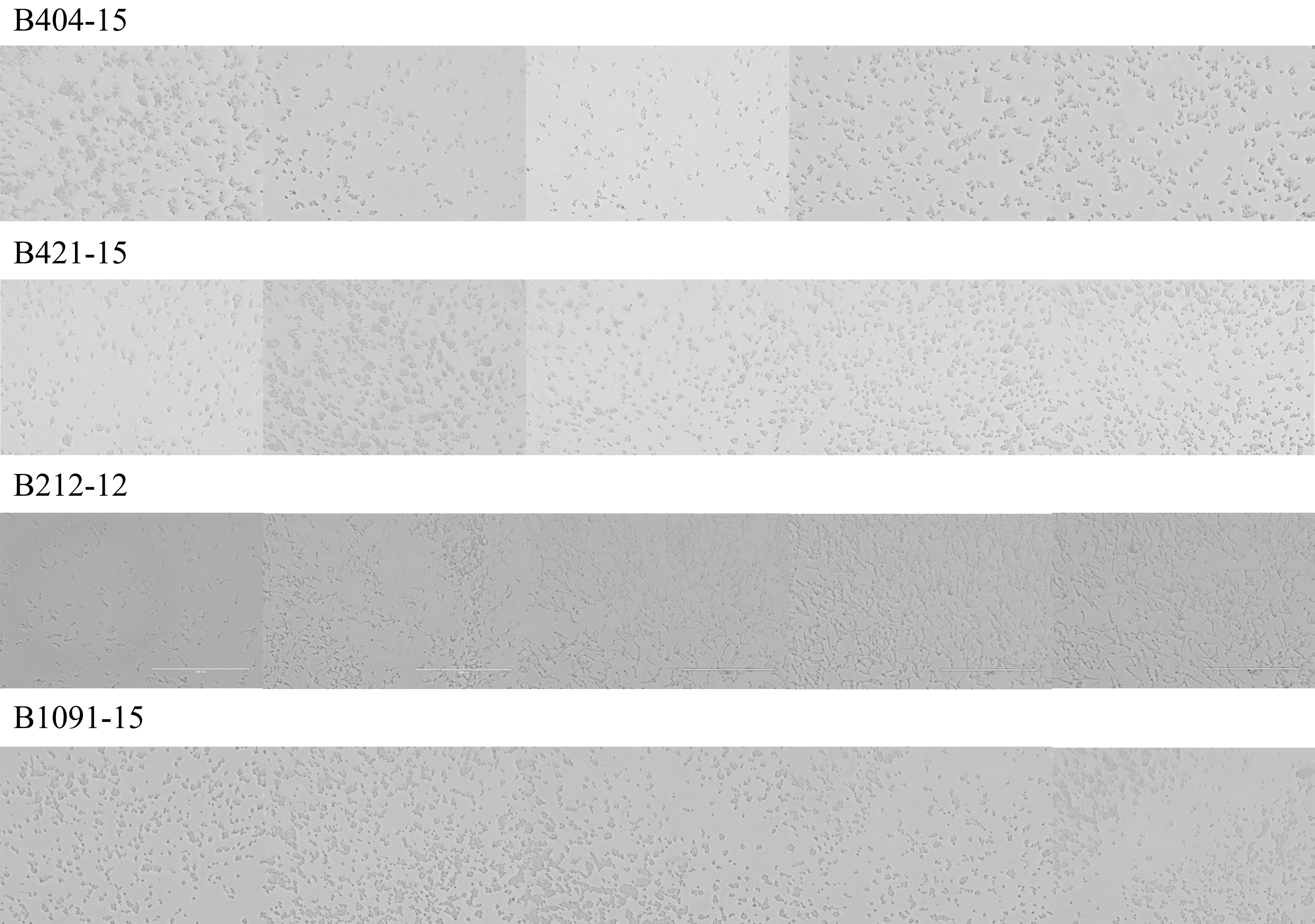

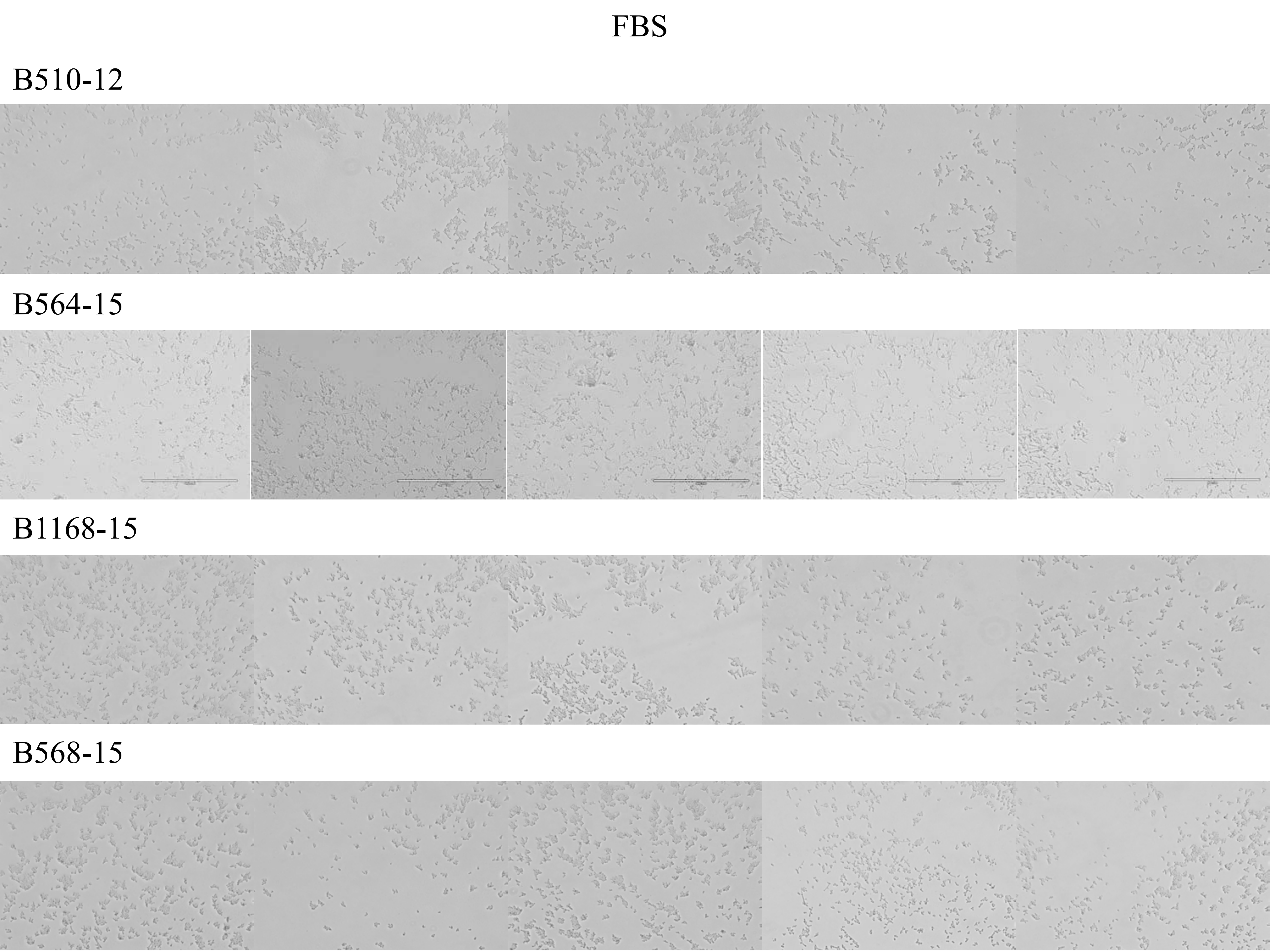
FBS
B510-12
B564-15
B1168-15
B568-15

FBS

B2527-12

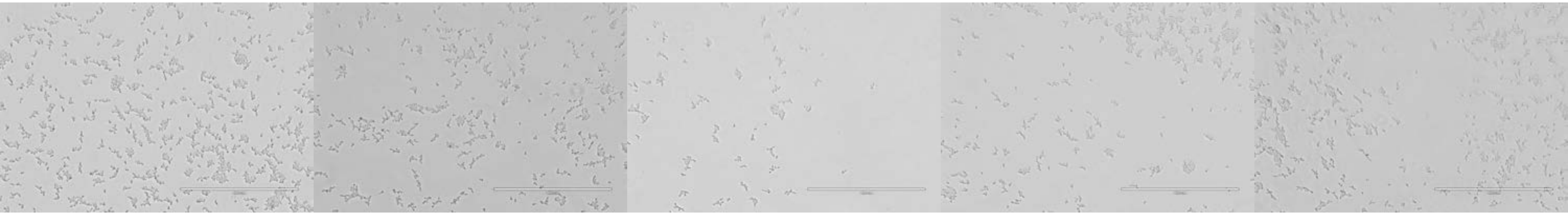

B1486-15

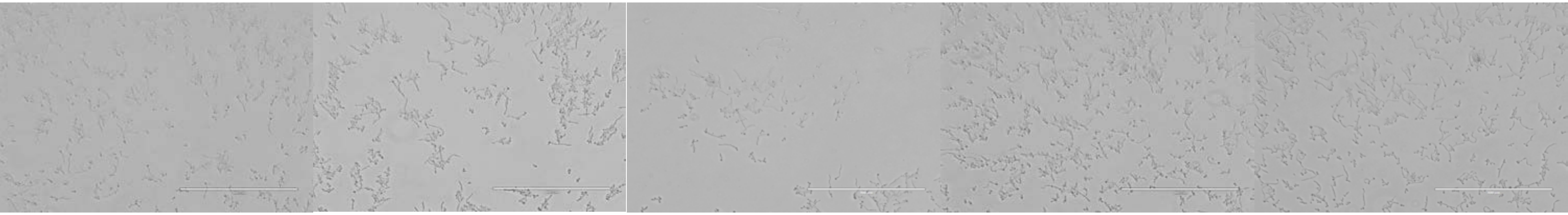

B1559-15

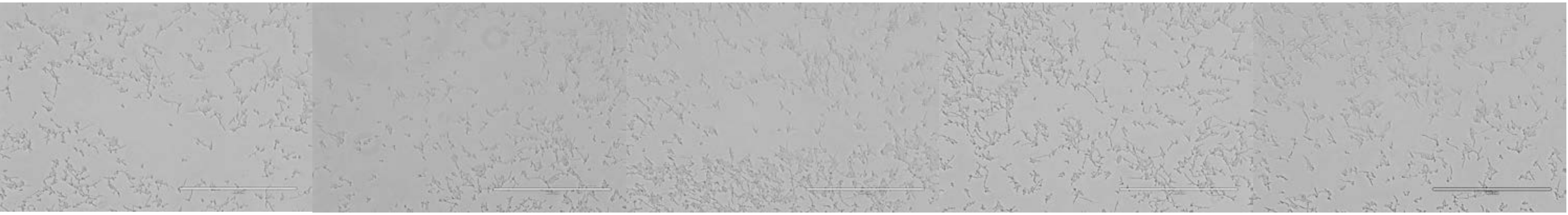

B733-15

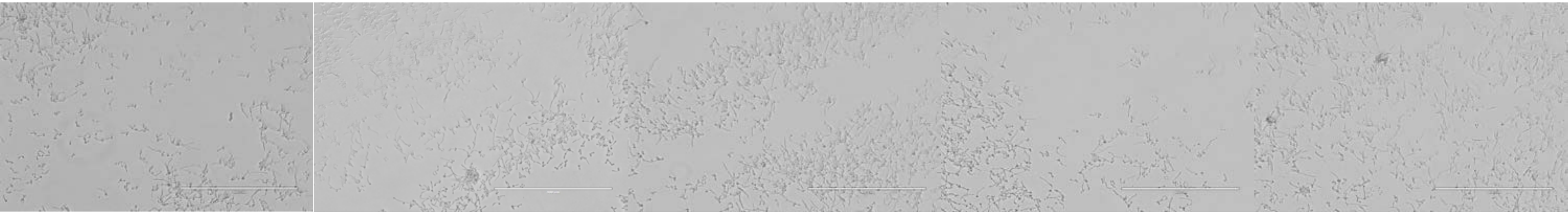

Lee's

B444-12

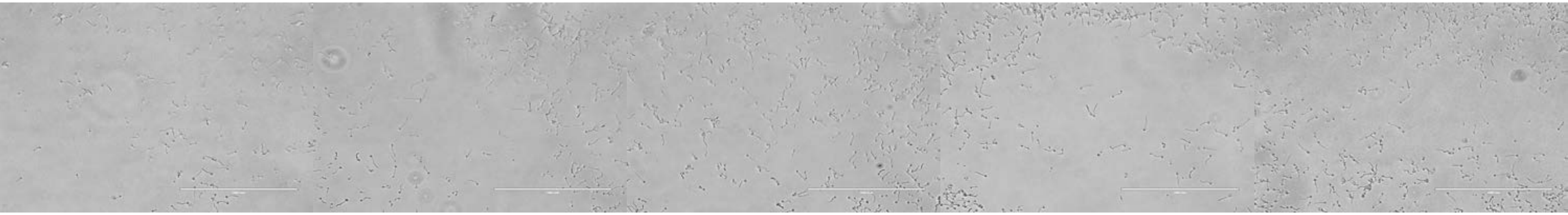

B1257-15

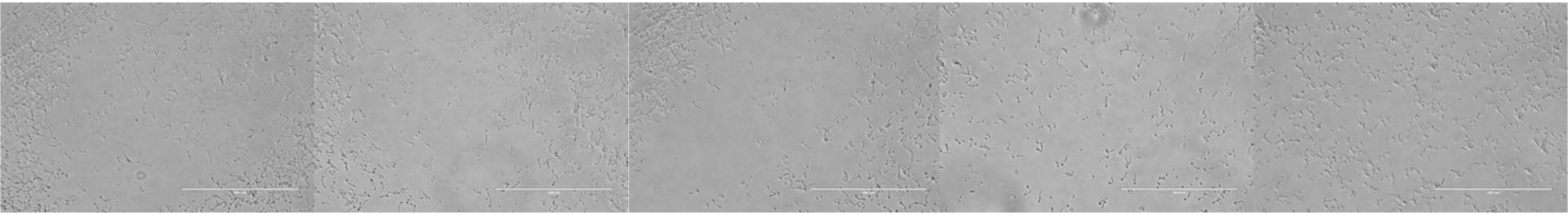

B687-15

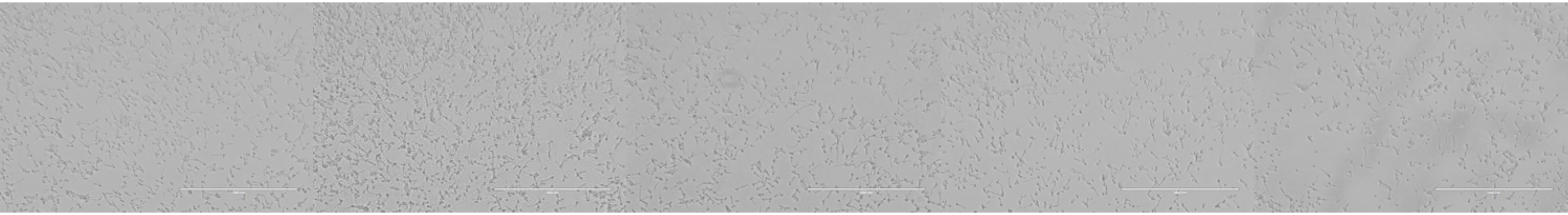

B1762-15

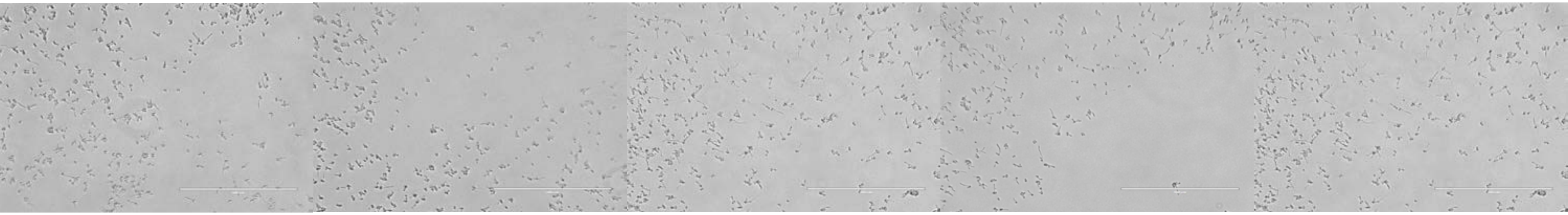

Lee's

B46-15

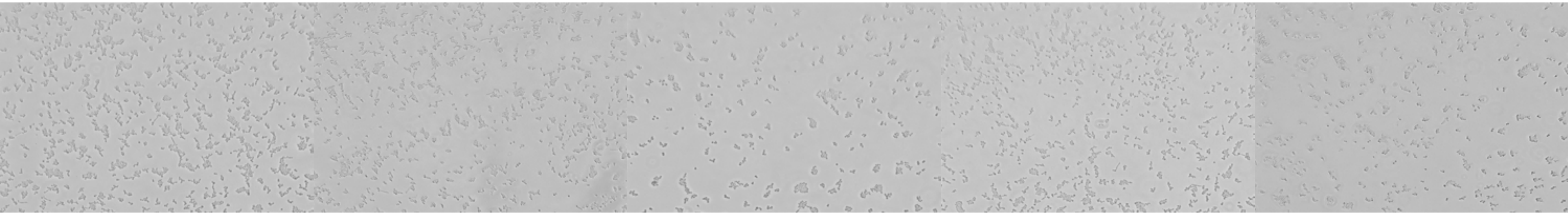

B808-15

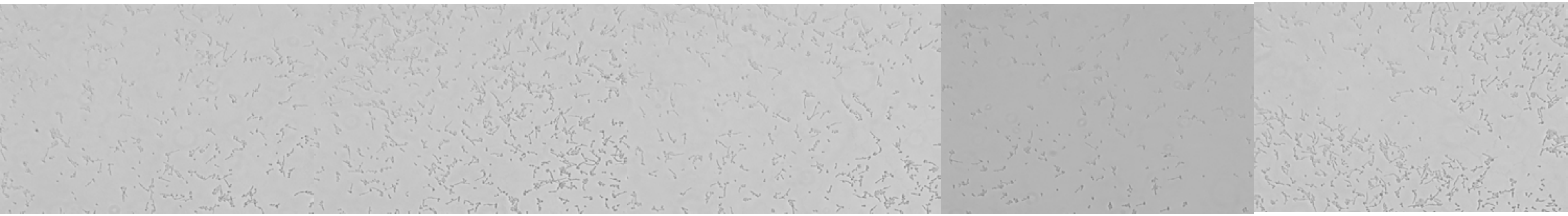

B527-15

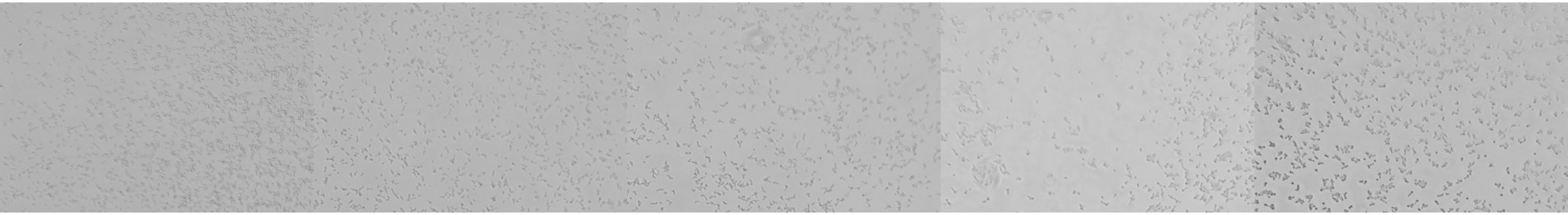

B618-15

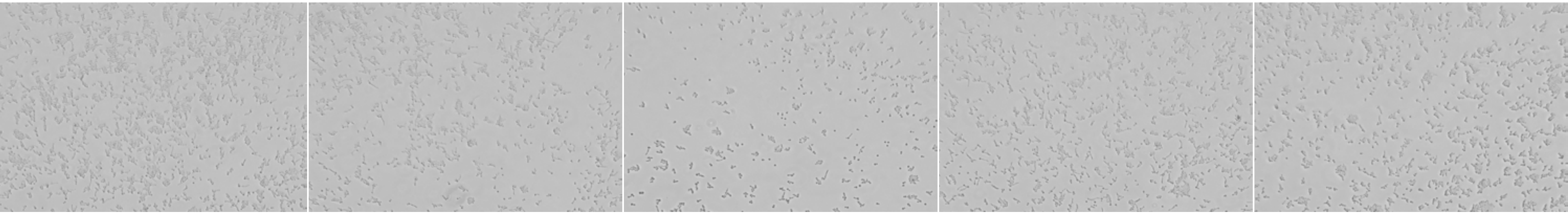

Lee's

B404-15

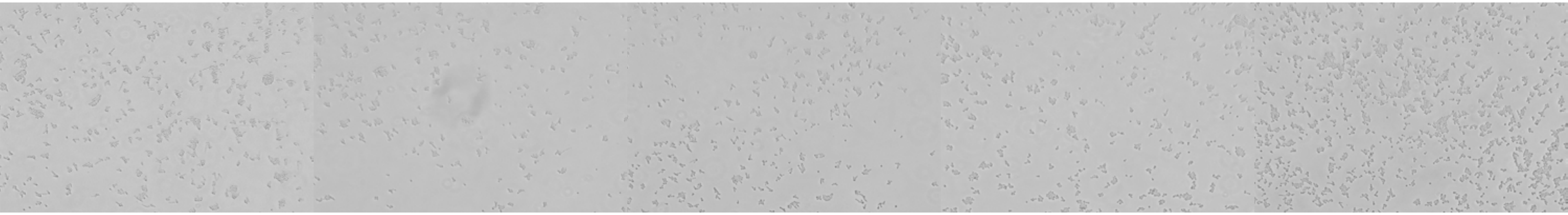

B421-15

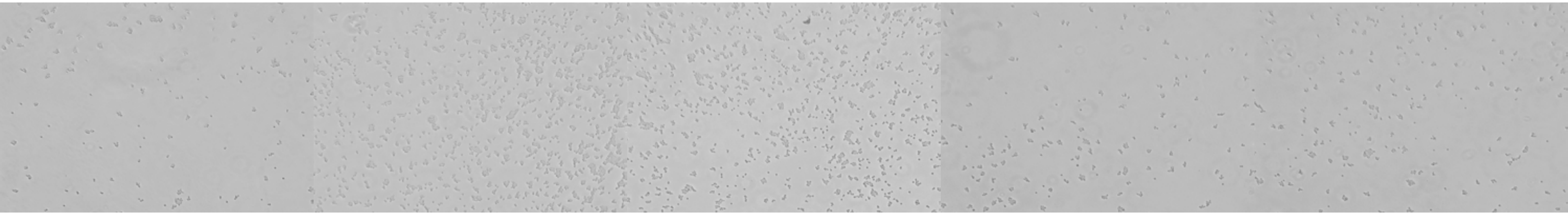

B212-12

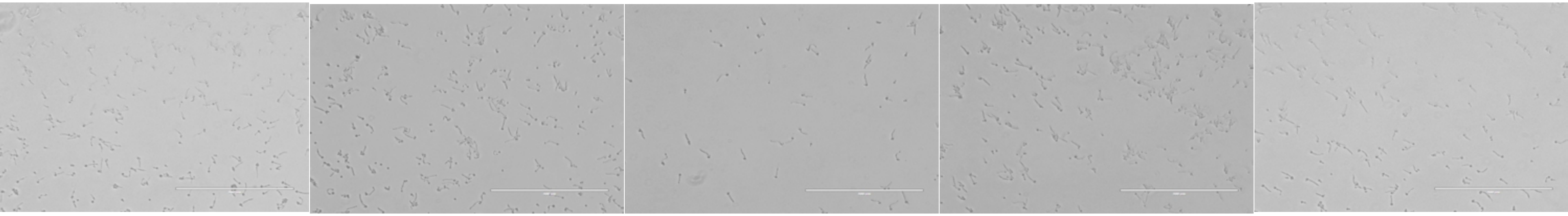

B1091-15

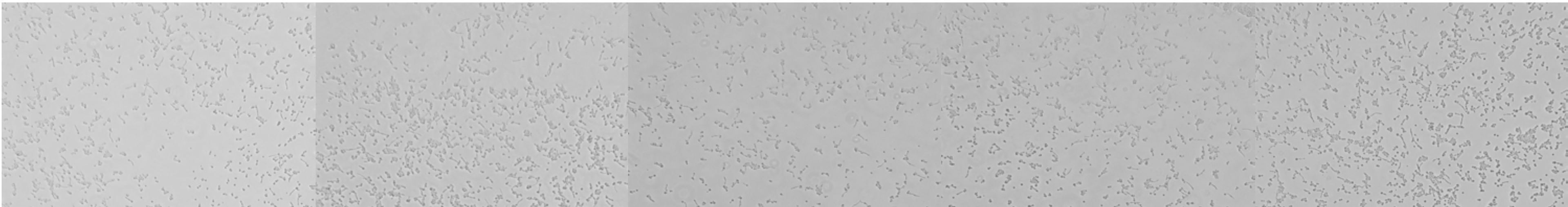

Lee's

B510-12

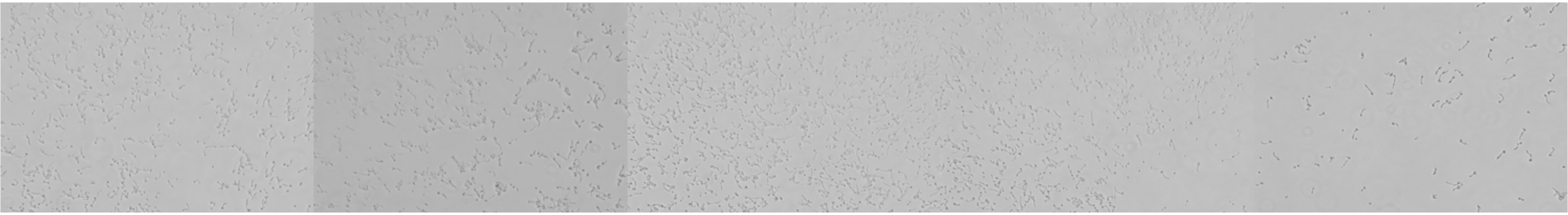

B564-15

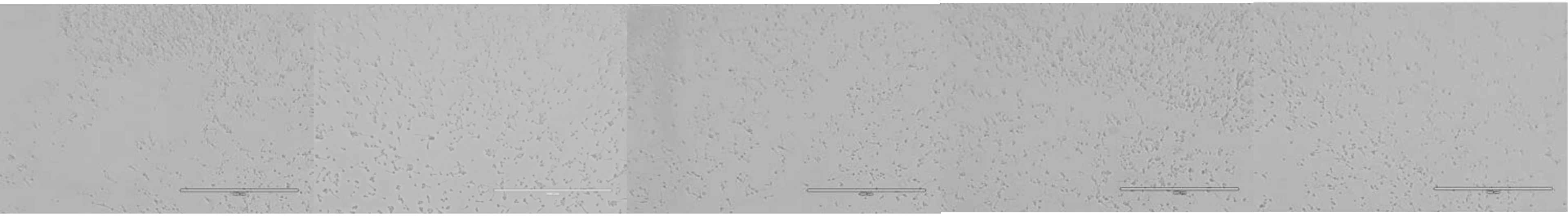

B1168-15

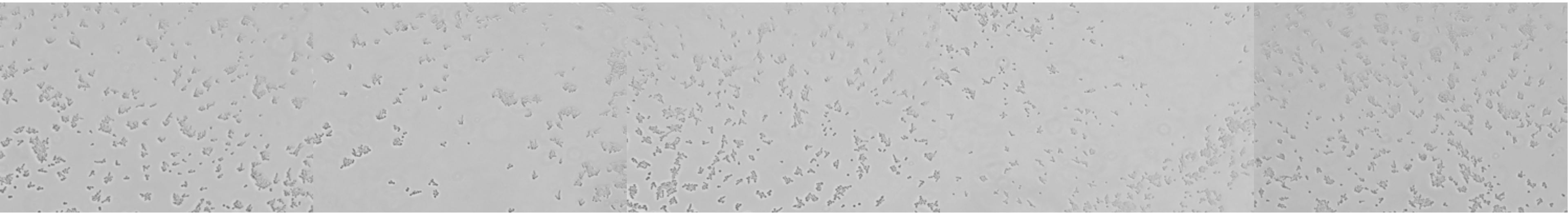

B568-15

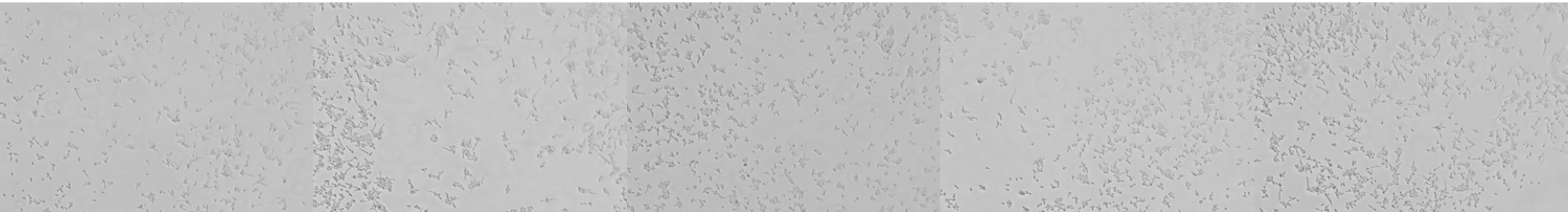

Lee's

B2527-12

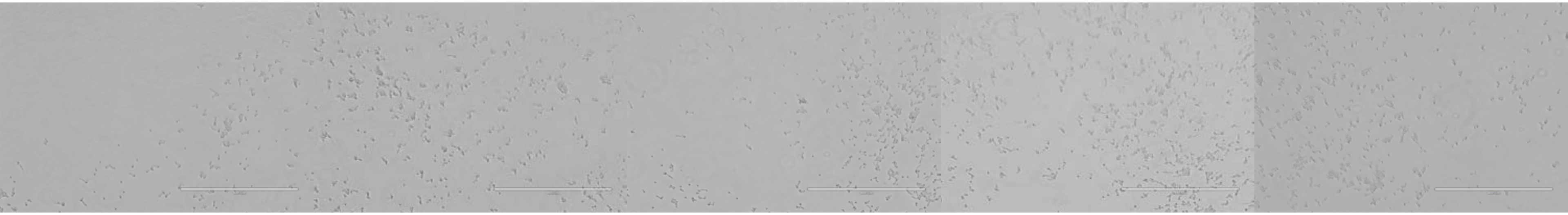

B1486-15

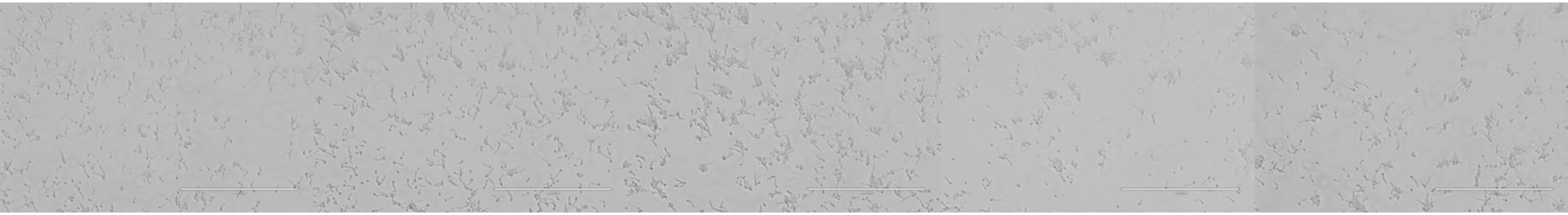

B1559-15

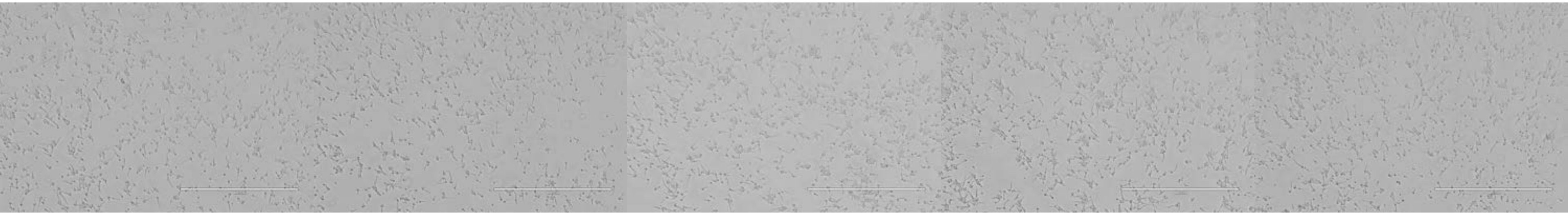

B733-15

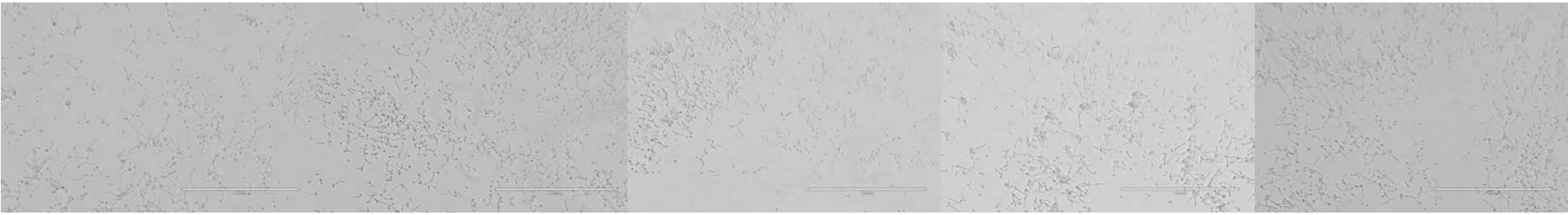

RPMI

B444-12

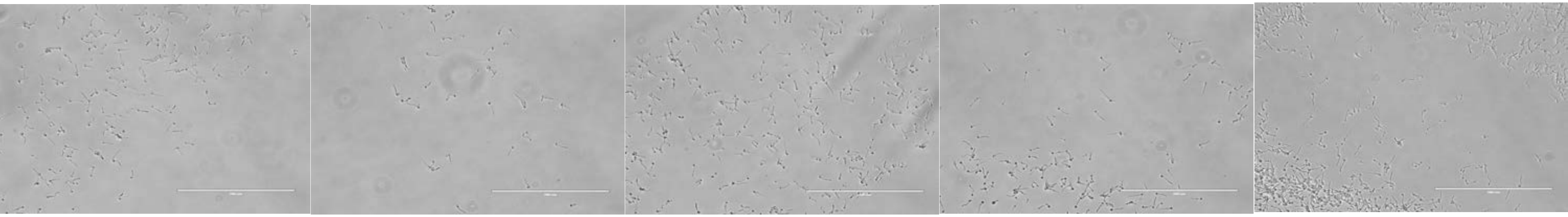

B1257-15

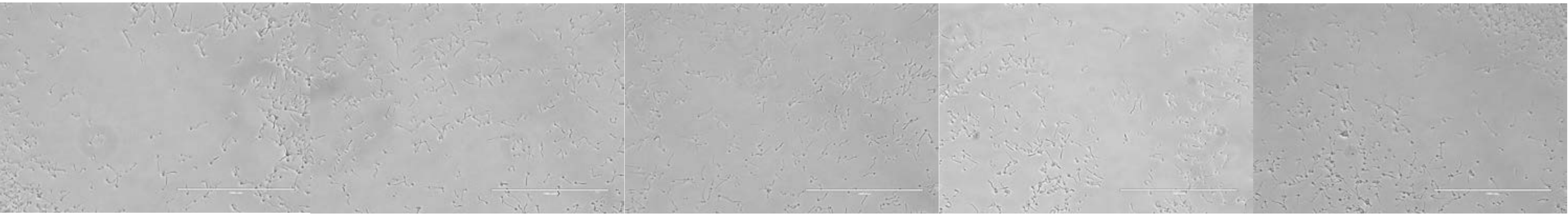

B687-15

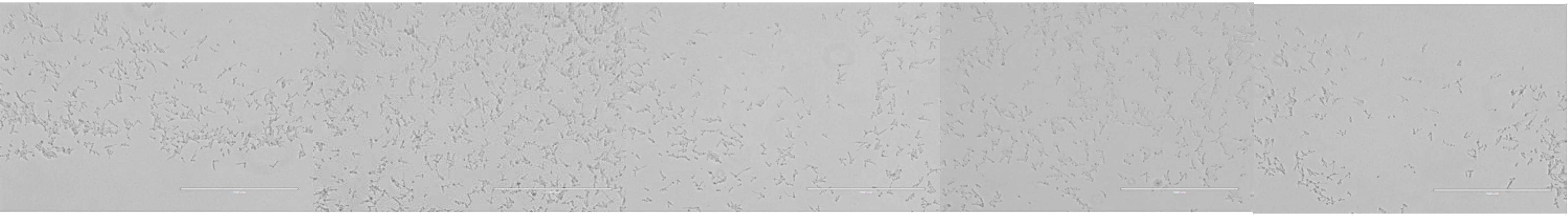

B1762-15

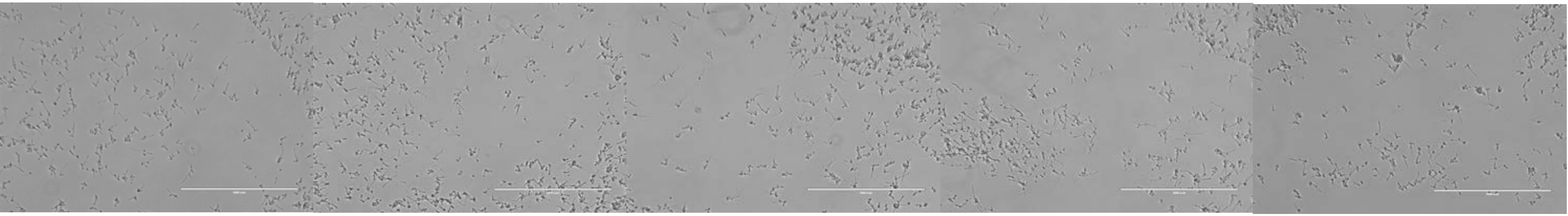

RPMI

B46-15

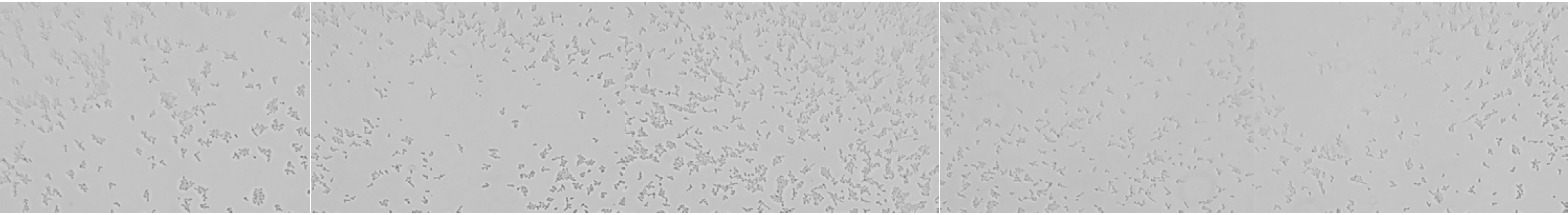

B808-15

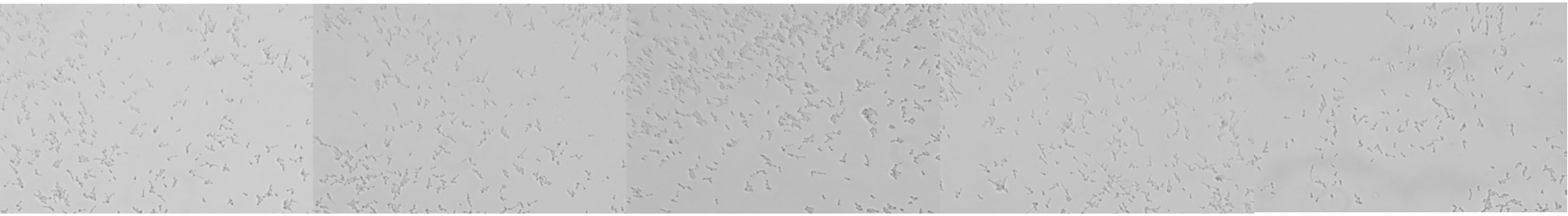

B527-15

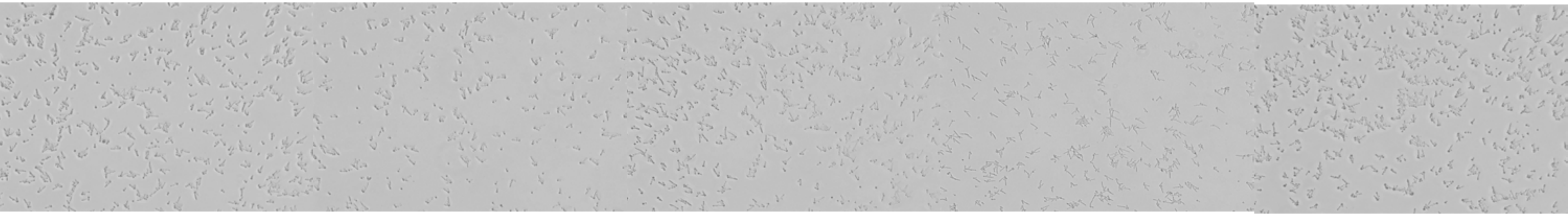

B618-15

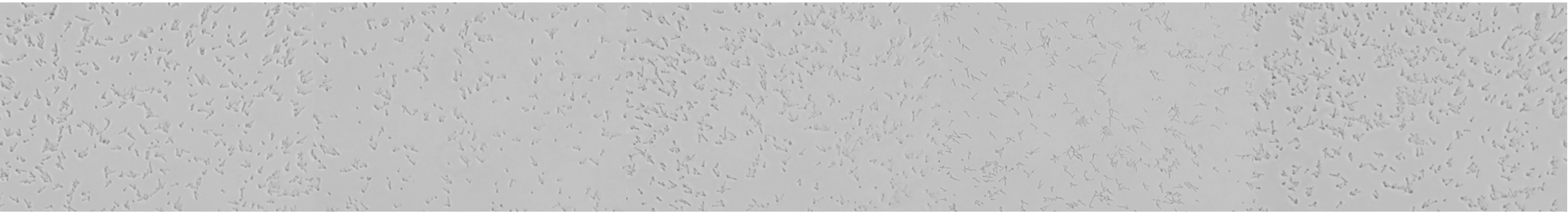

RPMI

B404-15

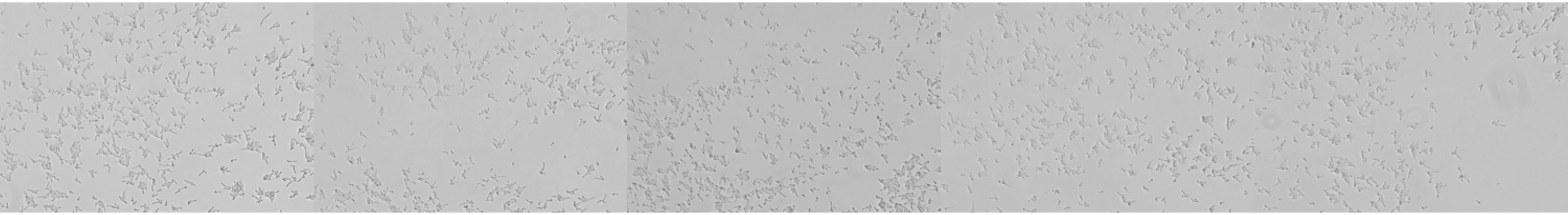

B421-15

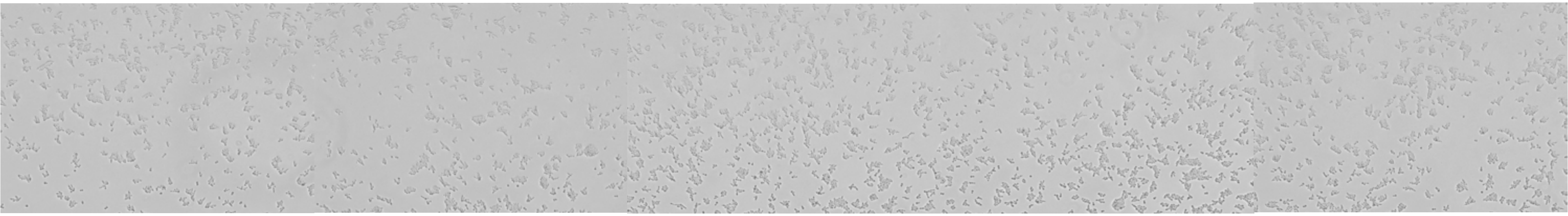

B212-12

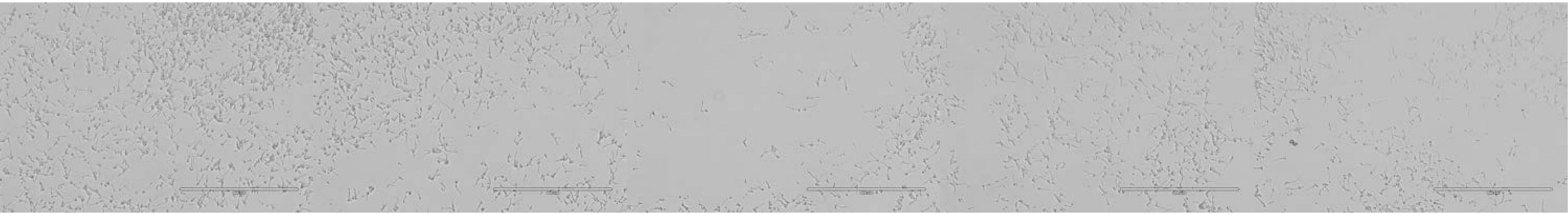

B1091-15

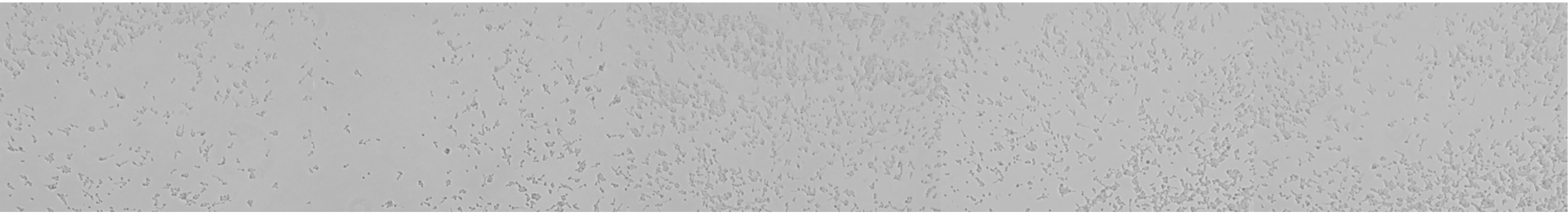

RPMI

B510-12

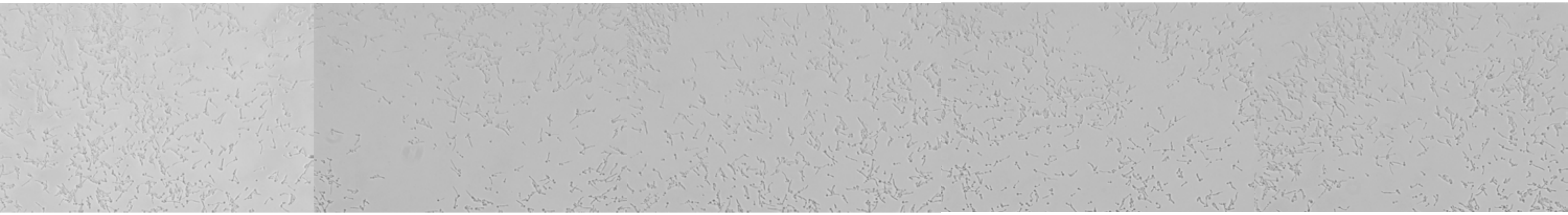

B564-15

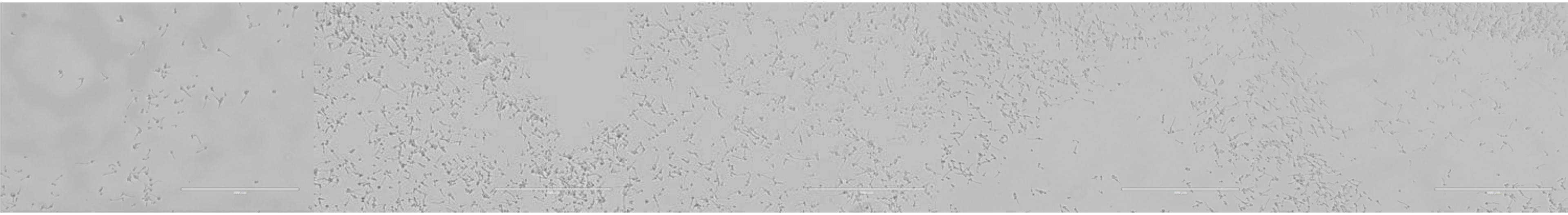

B1168-15

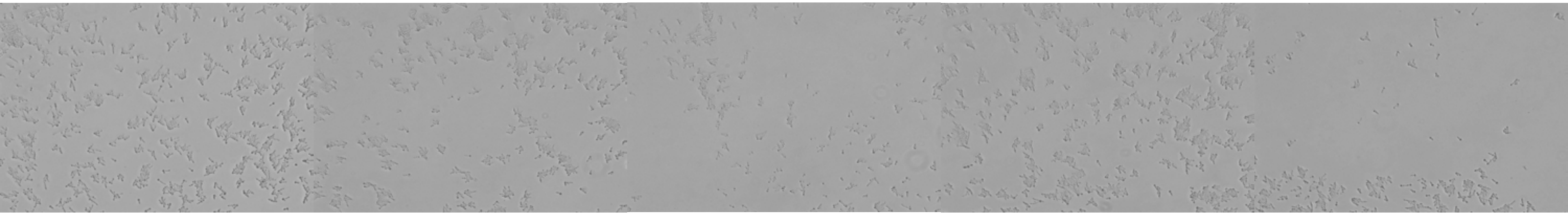

B568-15

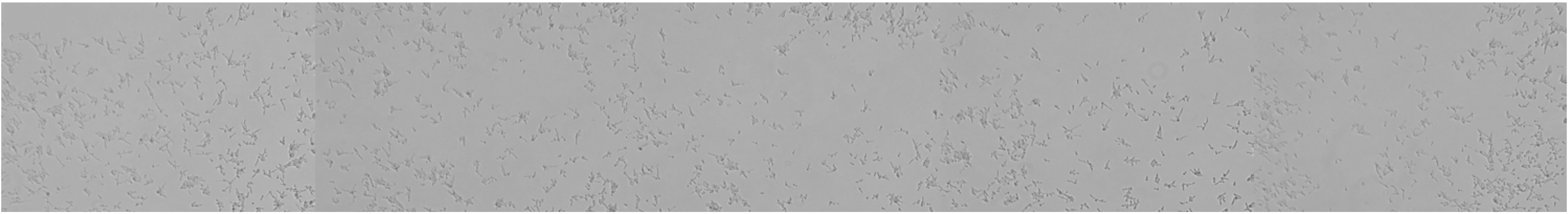

RPMI

B2527-12

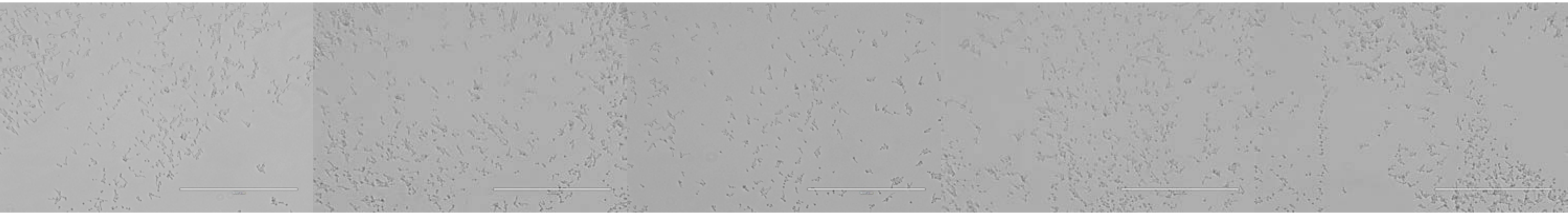

B1486-15

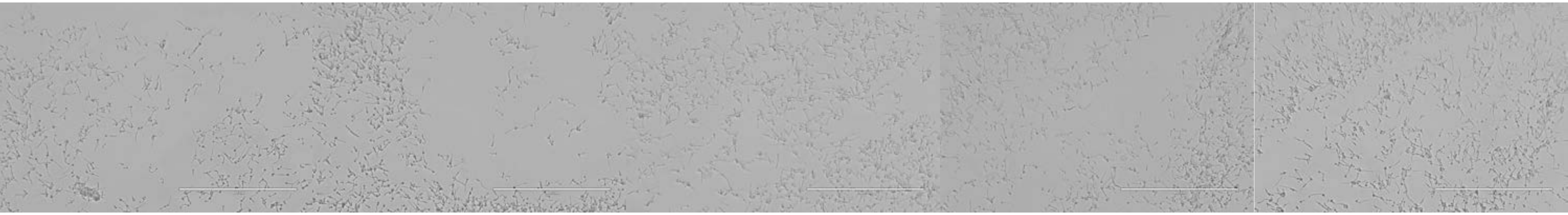

B1559-15

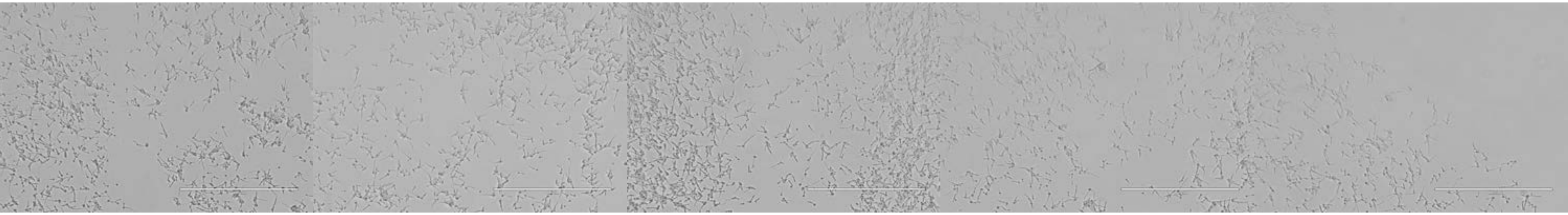

B733-15

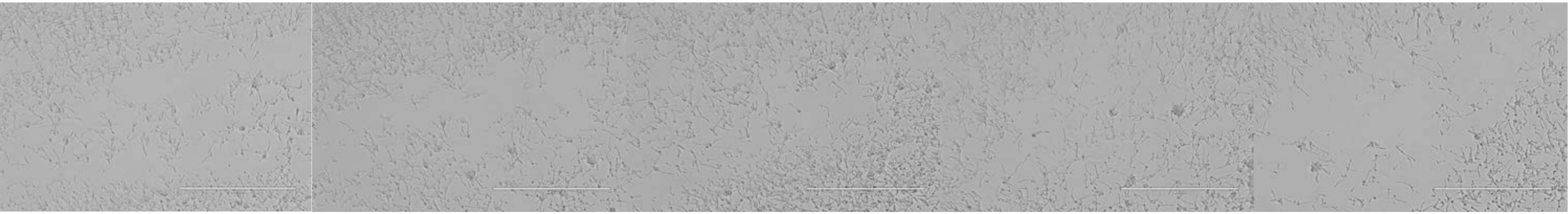

Spider

B444-12

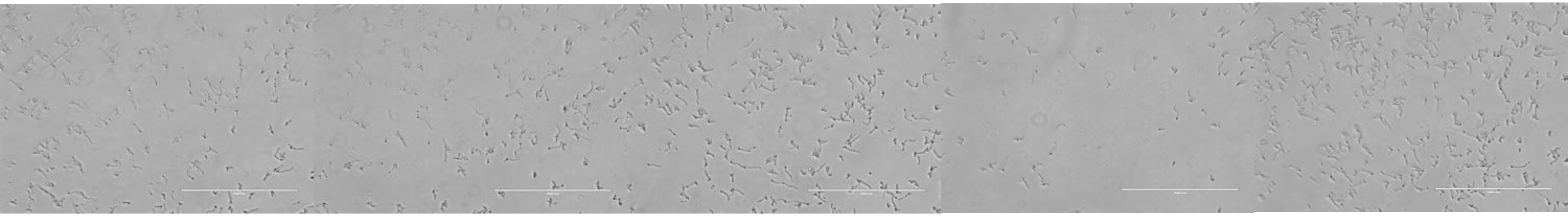

B1257-15

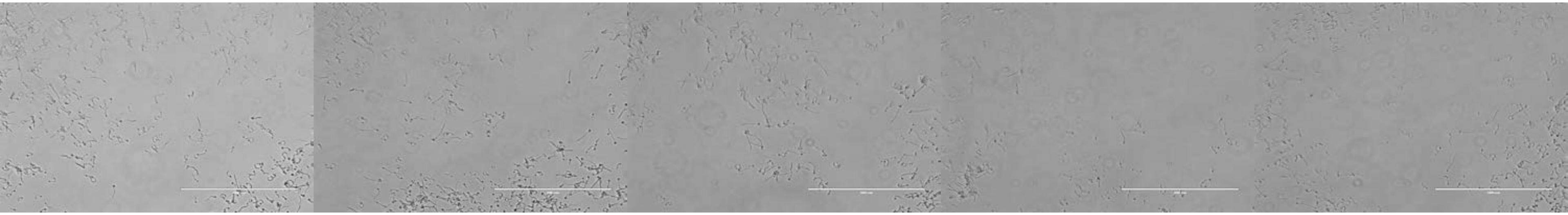

B687-15

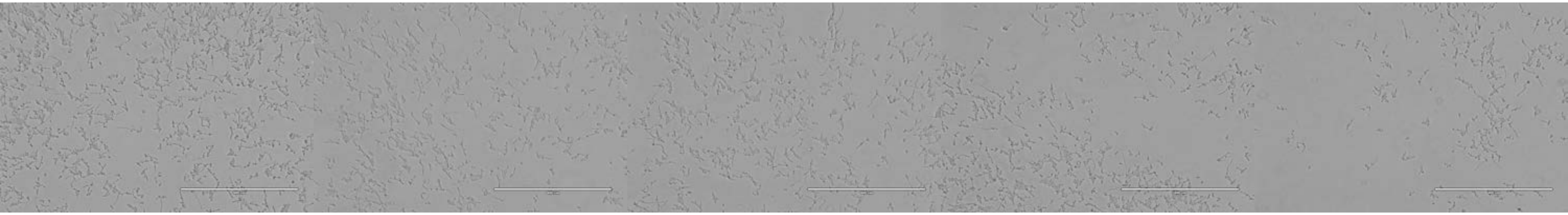

B1762-15

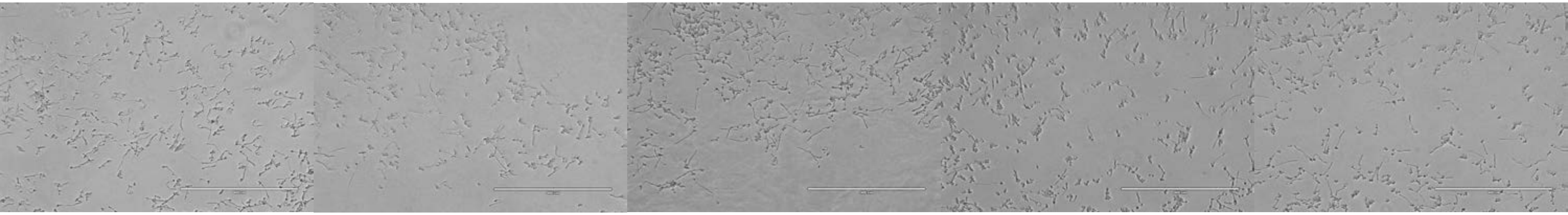

Spider

B46-15

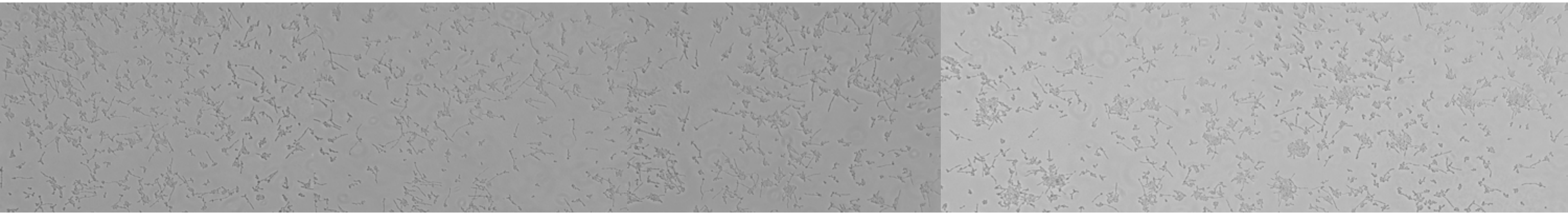

B808-15

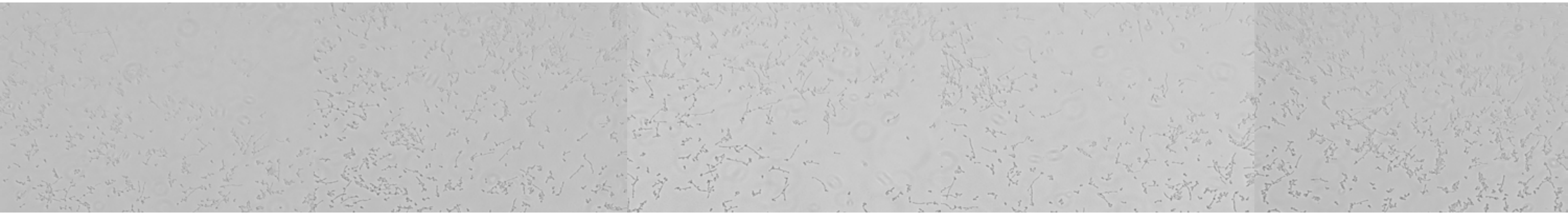

B527-15

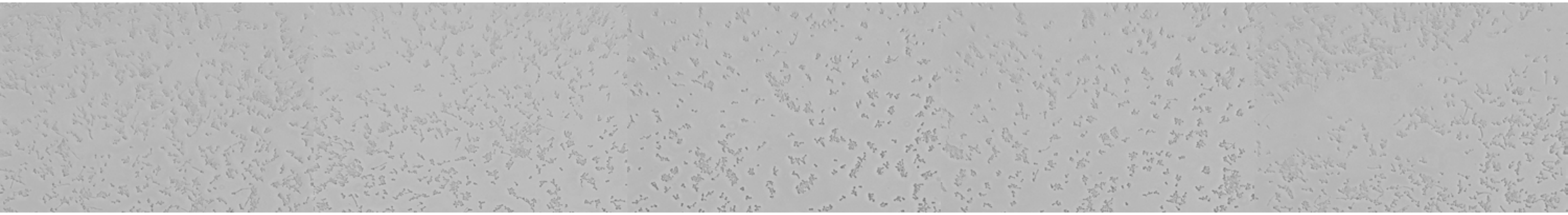

B618-15

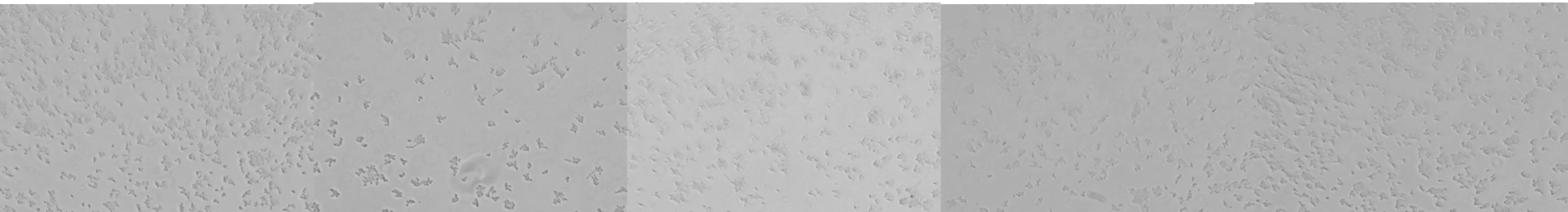

Spider

B404-15

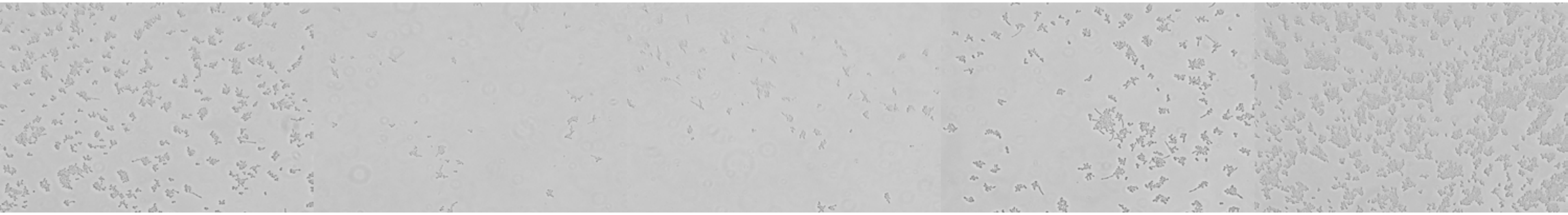

B421-15

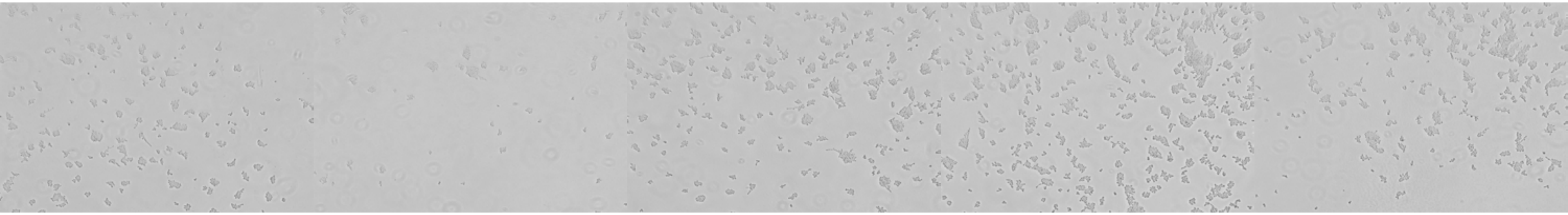

B212-12

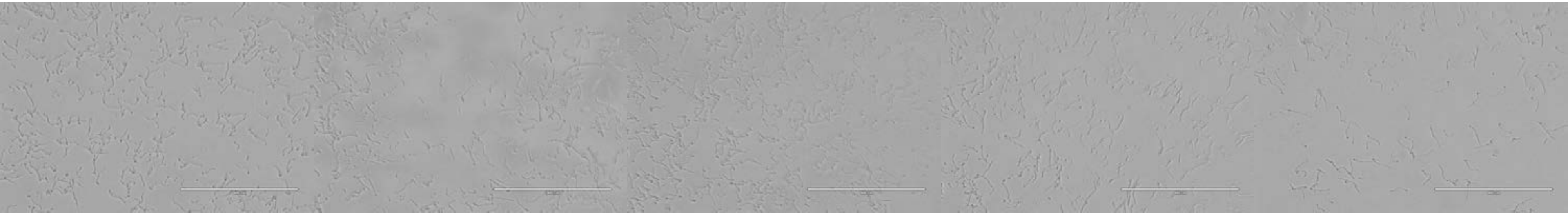

B1091-15

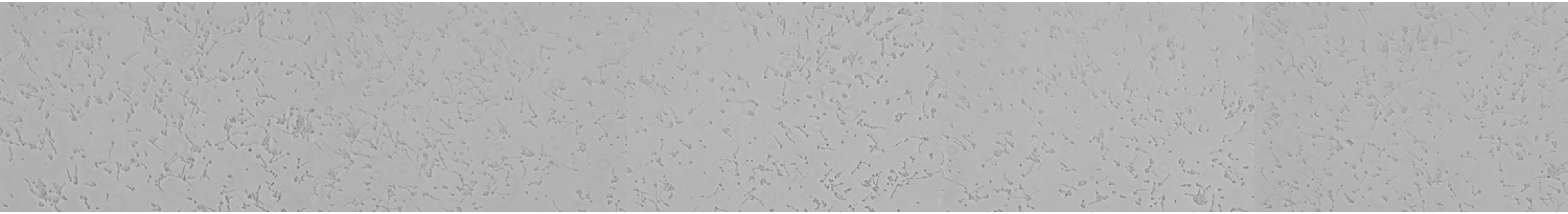

Spider

B510-12

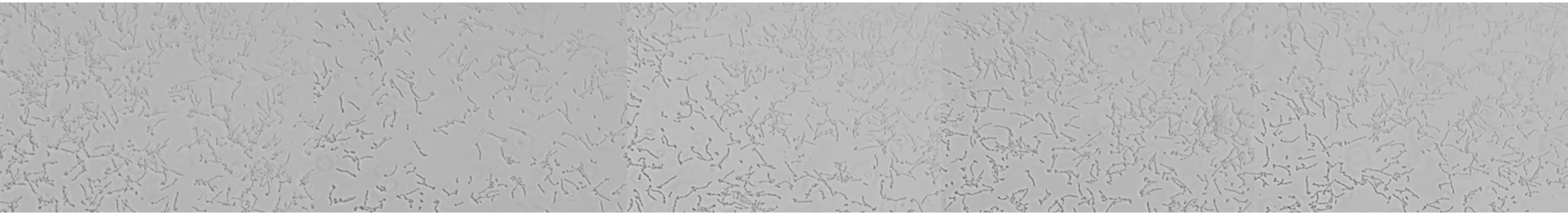

B564-15

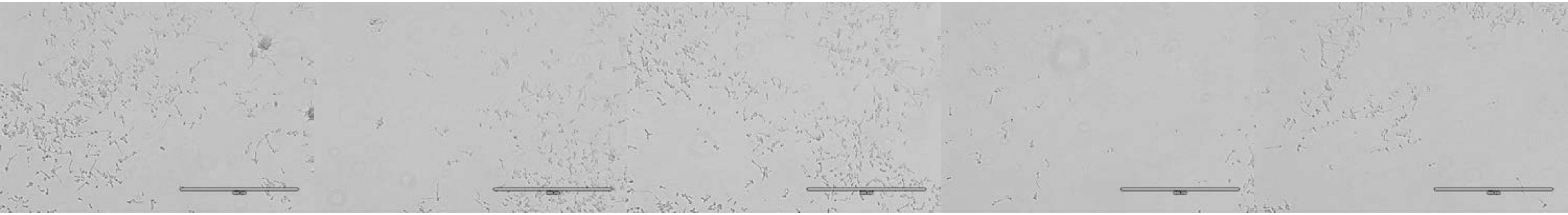

B1168-15

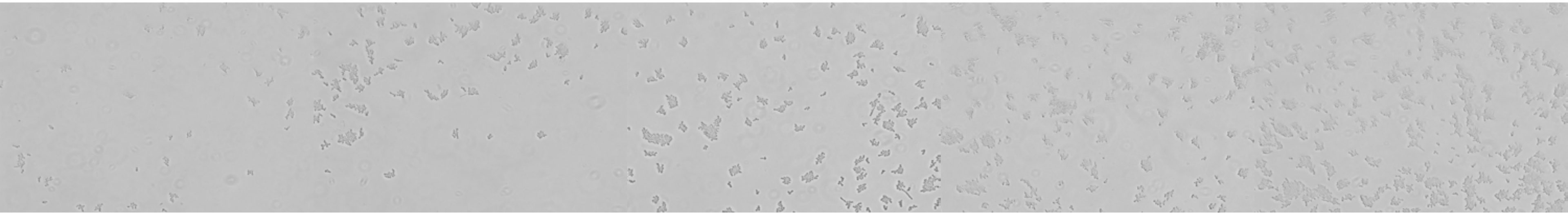

B568-15

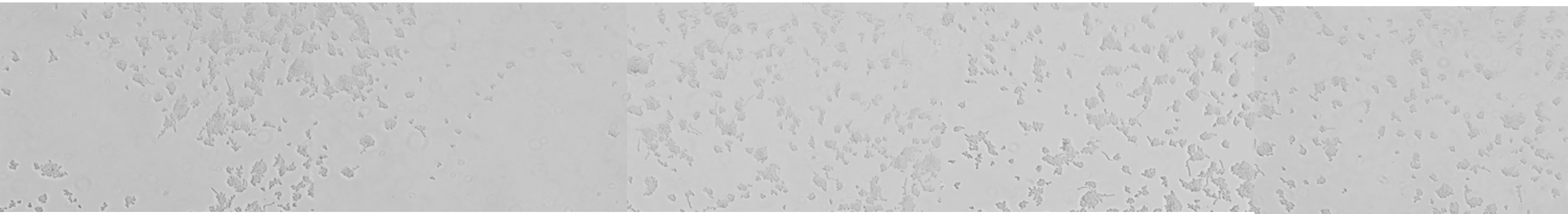

Spider

B2527-12

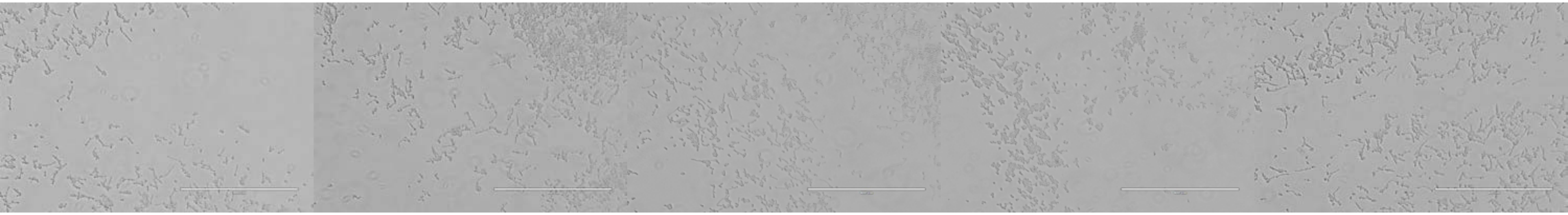

B1486-15

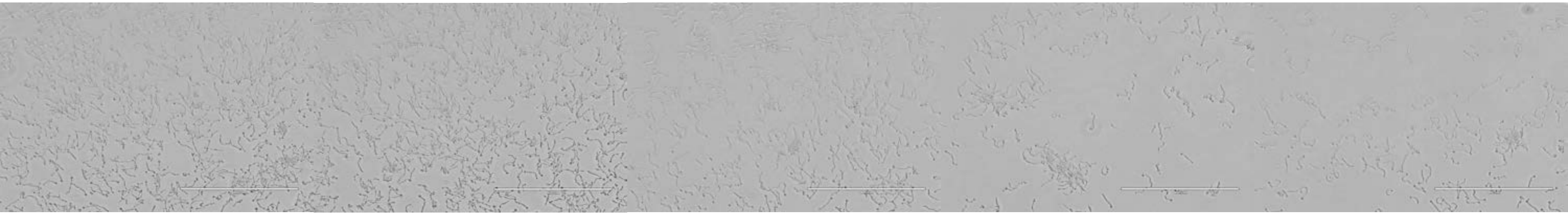

B1559-15

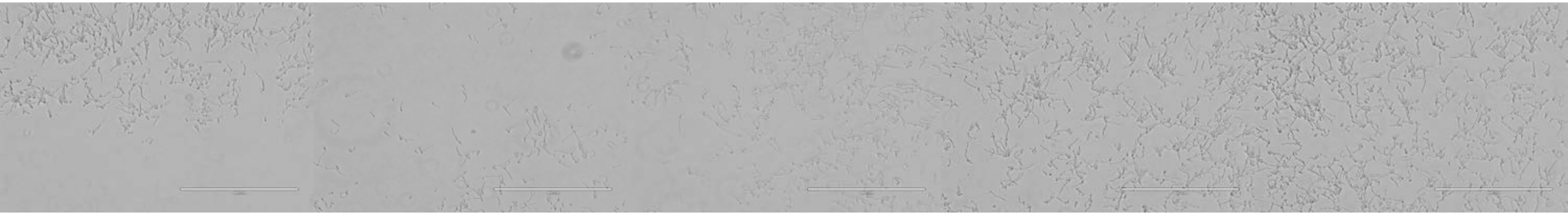

B733-15

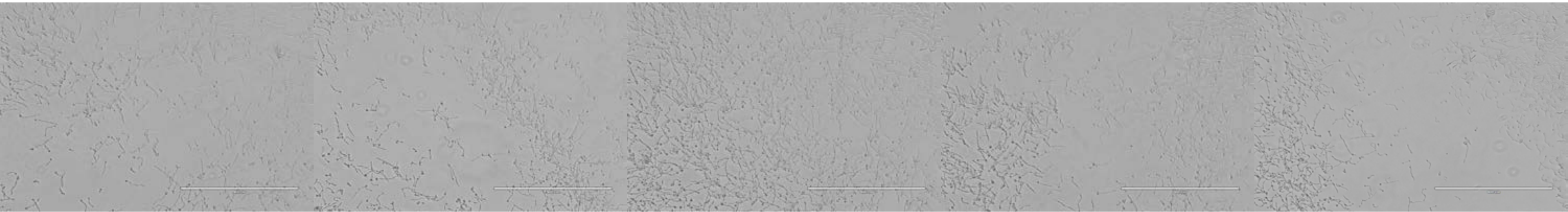

YPD

B444-12

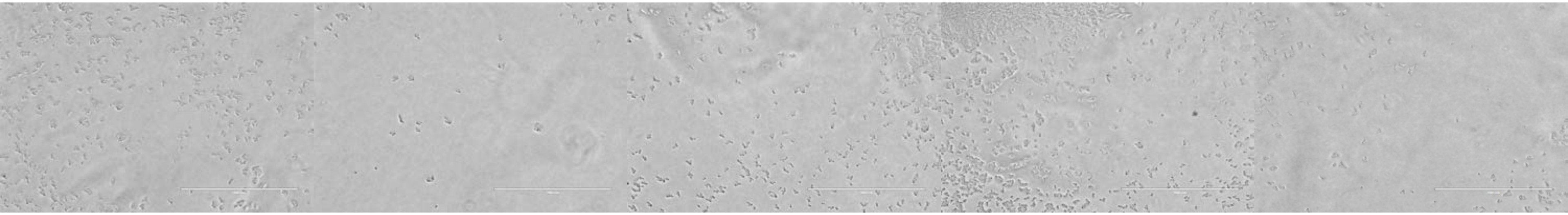

B1257-15

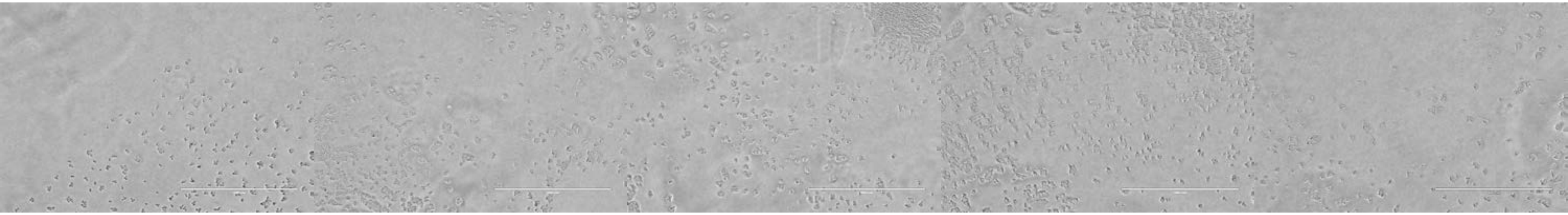

B687-15

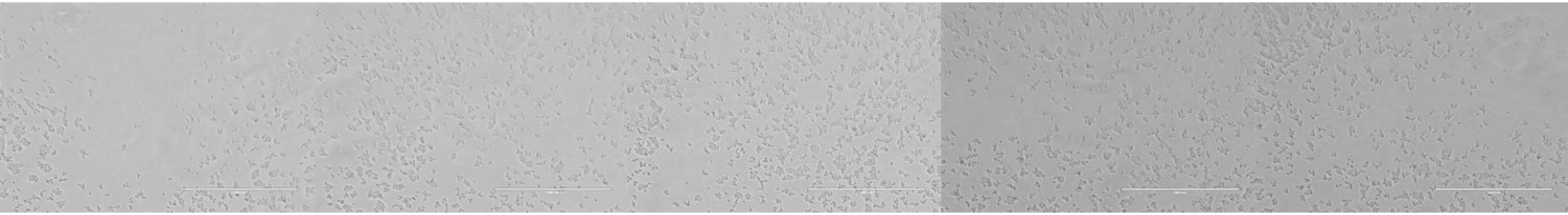

B1762-15

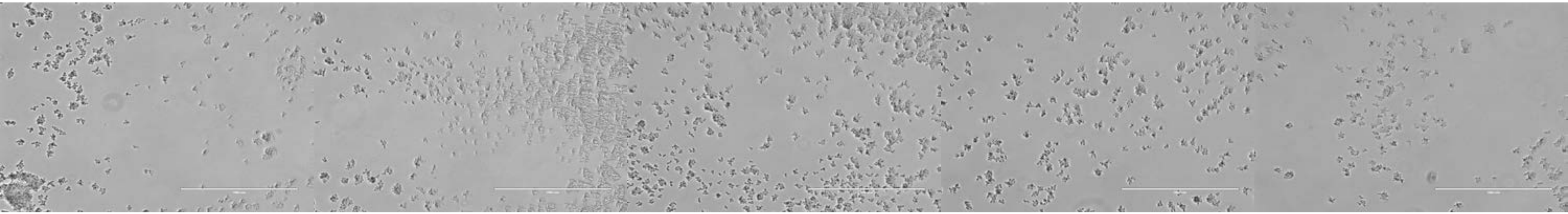

YPD

B46-15

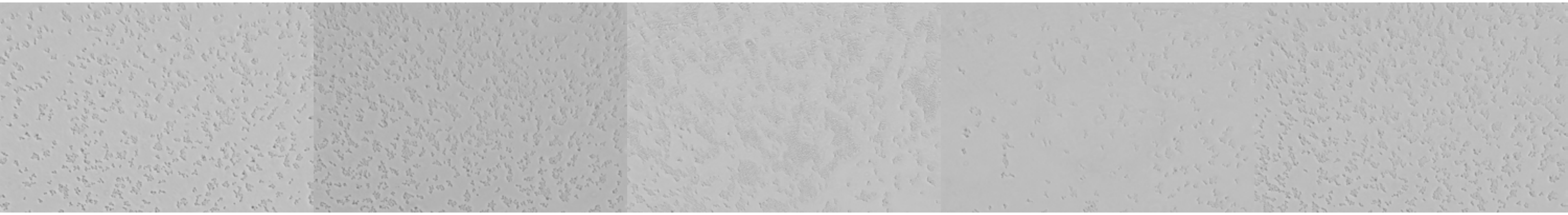

B808-15

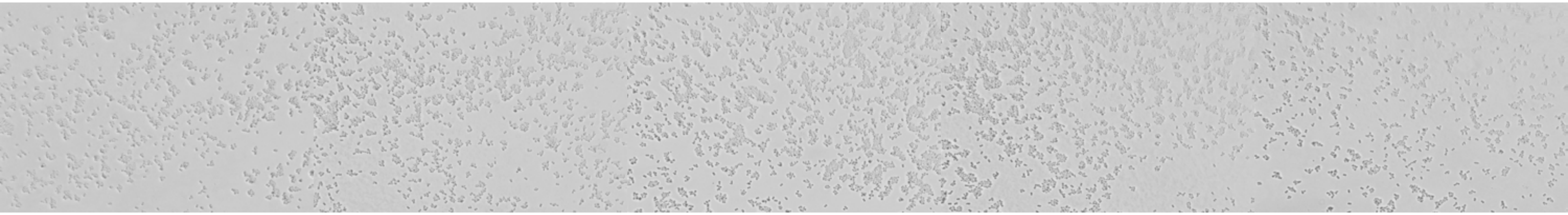

B527-15

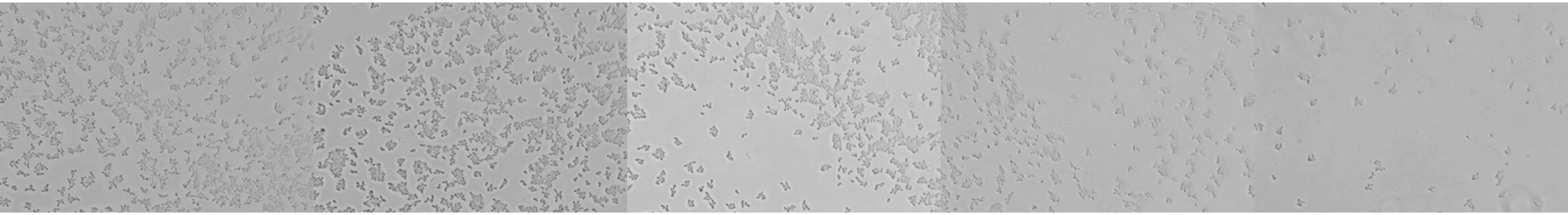

B618-15

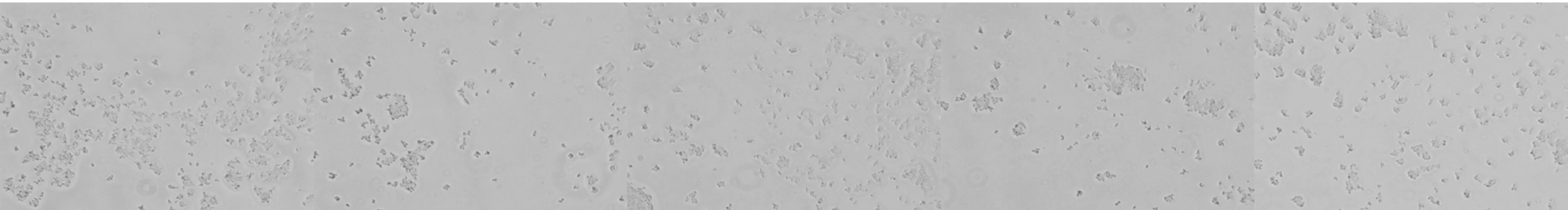

YPD

B404-15

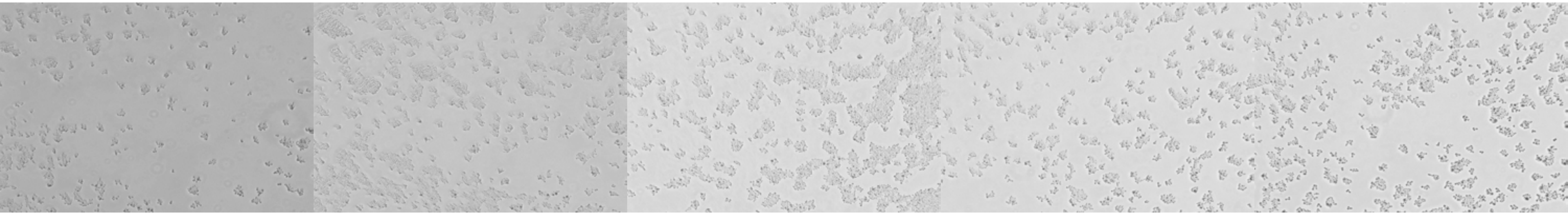

B421-15

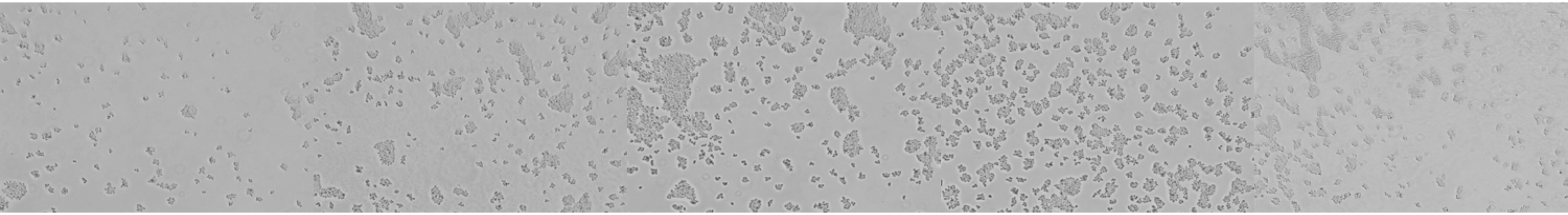

B212-12

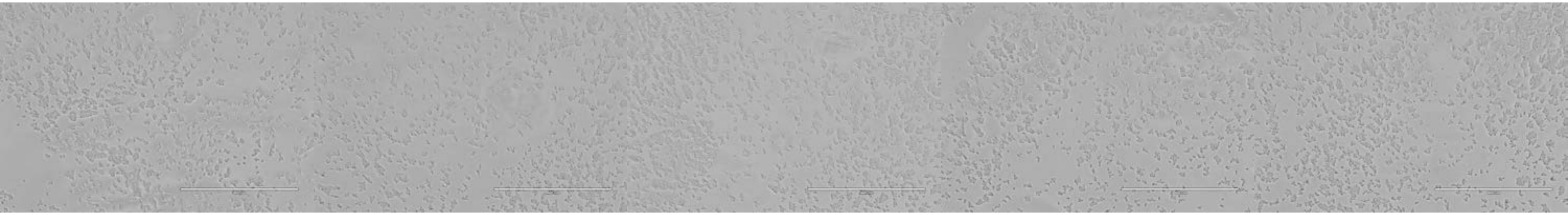

B1091-15

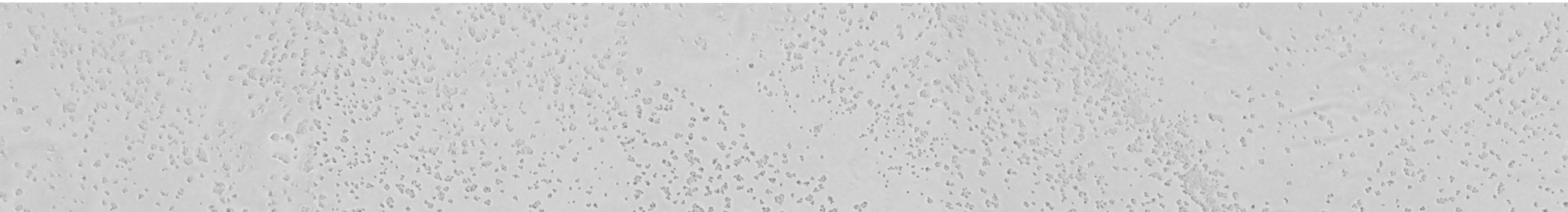

YPD

B510-12

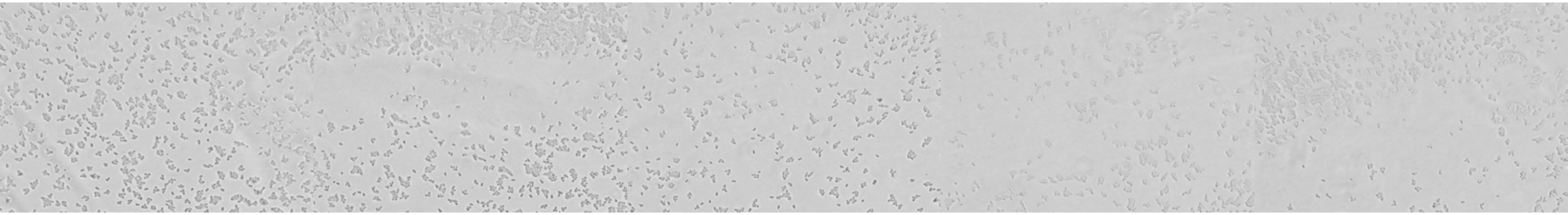

B564-15

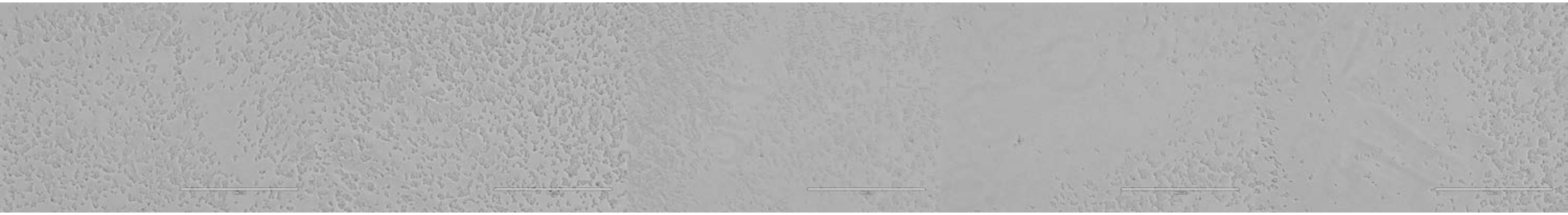

B1168-15

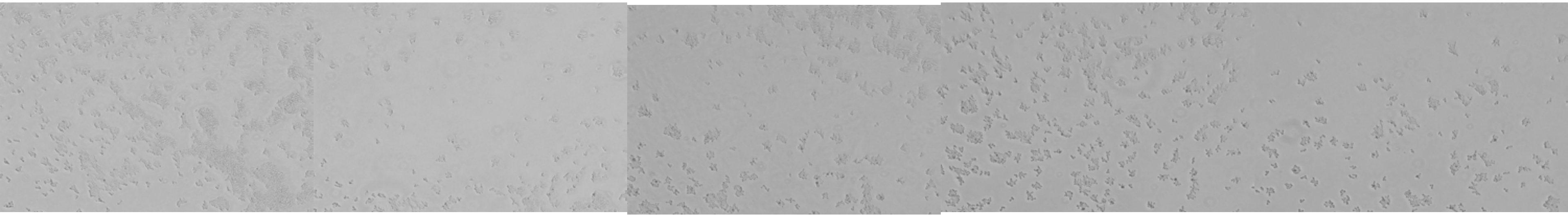

B568-15

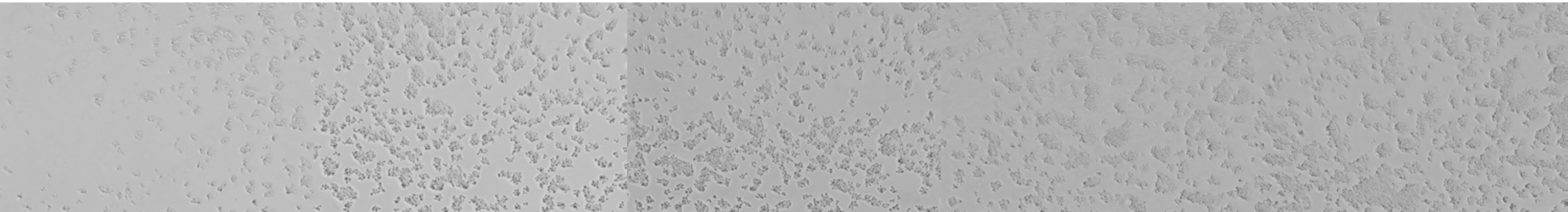

YPD

B2527-12

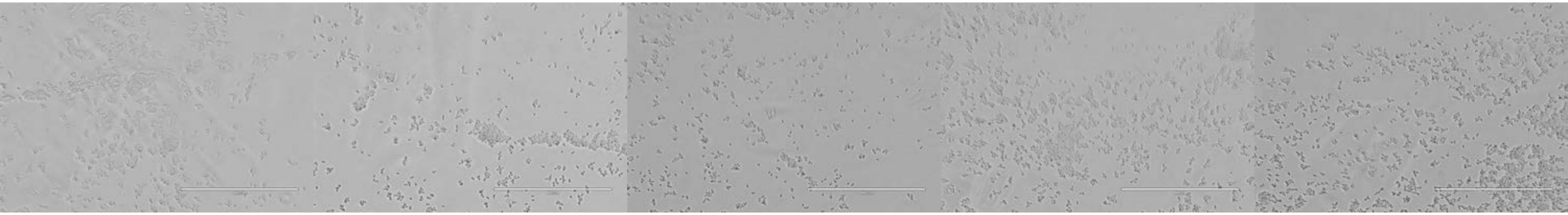

B1486-15

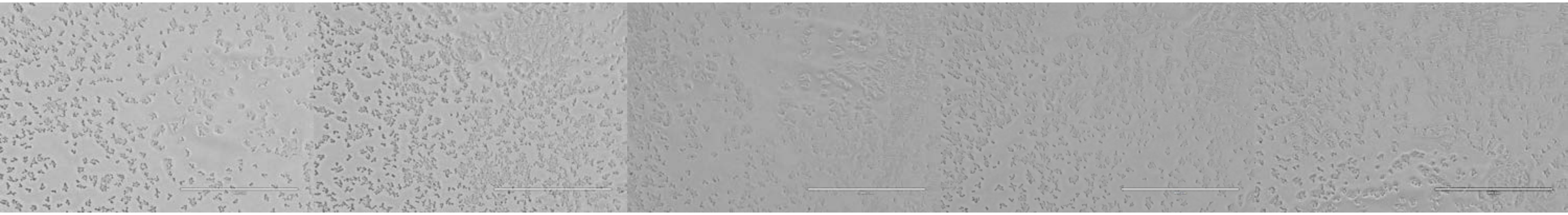

B1559-15

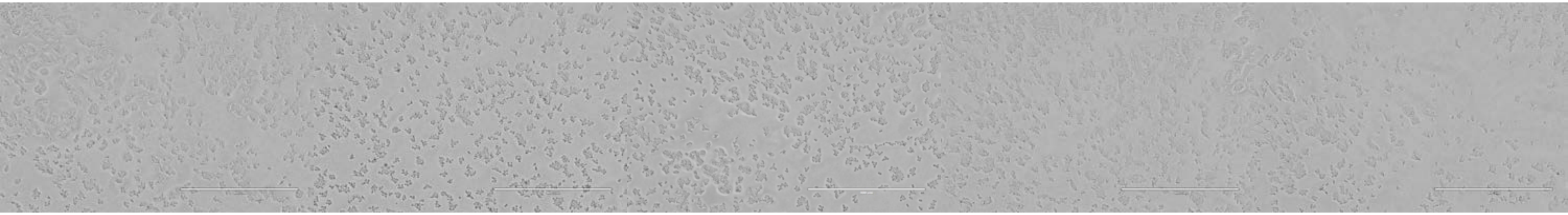

B733-15

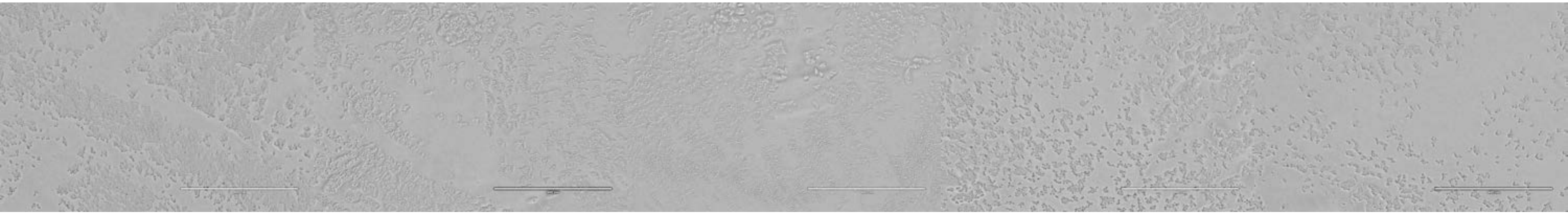

Supplement: Supplementary Figure 3 — Clinical strain filamentation in the shortened solid media assay. Cells of the indicated strains were tested for filamentation in standard and shortened solid filamentation assays in FBS, Lee’s, RPMI, or spider liquid media. Cells were grown overnight at 30˚C with shaking, washed, and then spread on inducing media agar plates and grown at 37˚C. Cells were also spread on YPD agar media and grown at 30˚C. Cells were imaged through the bottom of the agar plate after 3 hours of incubation. The images shown are a representative example of images assessed due to file size limitations. [file DataSheet_5.pdf]
