# Supplementary material for: Significant variation of filamentation phenotypes in clinical Candida albicans strains
Source: Front Cell Infect Microbiol. 2023 Oct 20;13:1207083. doi: 10.3389/fcimb.2023.1207083 (PMC10623444; doi:10.3389/fcimb.2023.1207083)
Supplement: Supplementary Table 1 — Primer Sequences for gene amplification and Sanger Sequencing. [file Table_1.docx]

Table S1. Primer Sequences for gene amplification and Sanger Sequencing

| Gene | Forward Primer | Reverse Primer |
| --- | --- | --- |
| AAT1a | ACTCAAGCTAGATTTTTGGC | CAGCAACATGATTAGCCC |
| ACC1 | GCAAGAGAAATTTTAATTCAATG | TTCATCAACATCATCCAAGTG |
| ADP1 | GAGCCAAGTATGAATGATTTG | TTGATCAACAAACCCGATAAT |
| MPIb | ACCAGAAATGGCCATTGC | GCAGCCATGCATTCAATTAT |
| SYA1 | AGAAGAATTGTTGCTGTTACTG | GTTACCTTTACCACCAGCTTT |
| VSP13 | TCGTTGAGAGATAATCGACTT | ACGGATGGATCTCCAGTCC |
| ZWF1 | GTTTCATTTGATCCTGAAGC | GCCATTGATAAGTACCTGGAT |

Table S2 Reference strains and clade designations

| Name | Clade | Name | Clade | Name | Clade | Name | Clade |
| --- | --- | --- | --- | --- | --- | --- | --- |
| SC5314 | **1** | **AM2003/0164** | **2** | **B2630V** | **5** | **L05-350** | **8** |
| RIHO16 | **1** | **PCStaff04** | **2** | **83/005** | **5** | **F262** | **8** |
| IHEM20469 | **1** | **81/066** | **2** | **F513** | **5** | **SCS125034** | **8** |
| CL4750 | **1** | **SCS66179** | **2** | **L516** | **5** | **P17-3** | **8** |
| AM2003/0101 | **1** | **JIMS500023** | **2** | **SCS72866E** | **5** | **T4** | **8** |
| J981309 | **1** | **AM2005/0433** | **2** | **JIMS500028** | **5** | **F124** | **8** |
| DPC60 | **1** | **AM2003/0191** | **2** | **JIMS160541** | **5** | **CP90** | **8** |
| 81/133 | **1** | **T30** | **2** | **SCSBB405121** | **5** | **AM2005/0431** | **8** |
| AM2003/0177 | **1** | **CP59** | **2** | **Niger14** | **5** | **AM2005/0475** | **8** |
| CLB49 | **1** | **85/019** | **3** | **JIMS500027** | **5** | **P12** | **9** |
| IHEM16348 | **1** | **D3** | **3** | **81/078** | **5** | **81/207** | **9** |
| BougnCP10 | **1** | **d21** | **3** | **AM2003/0084** | **5** | **IHEM16603** | **9** |
| SCS50393 | **1** | **d9** | **3** | **WO1** | **6** | **CCHURS10** | **9** |
| WC03-203424 | **1** | **EGPURC17** | **3** | **SCSXM69475** | **6** | **P13** | **9** |
| J940855 | **1** | **CL4753** | **3** | **b30644/6** | **6** | **CL4735** | **9** |
| AM2003/0107 | **1** | **AM2005/0394** | **3** | **CP62** | **6** | **J981326** | **9** |
| YsU568 | **1** | **SCS47669** | **3** | **90/205** | **6** | **FJ12** | **9** |
| CL4736 | **1** | **DPC91** | **3** | **AM2005/0425** | **6** | **APRURC3** | **9** |
| CP01 | **1** | **J990103** | **3** | **SCSB38619** | **6** | **APRURM6** | **9** |
| CH14 | **1** | **SCSXM69885** | **4** | **AM2004/0022** | **6** | **JIMS500026** | **10** |
| P16 | **1** | **AM2003/0186** | **4** | **C19** | **6** | **IHEM16604** | **10** |
| L2 | **1** | **EGPHRC1** | **4** | **SCS133719B** | **6** | **AM2003-014** | **10** |
| JIMS160529 | **1** | **CP58** | **4** | **HUN122** | **7** | **IHEM16473** | **10** |
| SCSM0528774 | **1** | **AM2005/0447** | **4** | **73/024** | **7** | **AM2005/0365** | **10** |
| AM2003-017 | **1** | **AM2004/0024** | **4** | **73/027** | **7** | **AM2003/0054** | **10** |
| 81/148 | **1** | **F59** | **4** | **AM2005/0355** | **7** | **85/050** | **10** |
| HK04M121276 | **1** | **G25119** | **4** | **AM2005/0406** | **7** | **BCHURC12** | **10** |
| CP52 | **1** | **L1114** | **4** | **AM2005/0021** | **7** | **BCHURC10** | **10** |
| SCS74518E | **1** | **SCS125409F** | **4** | **AM2005/0460** | **7** | **ABCURC3** | **10** |
| 81/192 | **1** | **AM2004/0006** | **5** | **T125** | **7** | **AM2005/0378** | **11** |
| WC02-202861 | **1** | **AM2004/0004** | **5** | **L1123** | **7** | **DPC64** | **11** |
| SLSURC4 | **1** | **AM2004/0007** | **5** | **AM2005/0022** | **7** | **DPC168** | **11** |
| DPC38 | **1** | **AM2004/0008** | **5** | **b31331/6** | **7** | **PCA** | **11** |
| DPC54 | **1** | **J950661** | **5** | **JIMS103102** | **7** | **CP80** | **11** |
| EGPURM14 | **1** | **P08** | **5** | **A873646** | **7** | **73/084** | **11** |
| FC22 | **2** | **DPC207** | **5** | **NCPF9016** | **7** | **DPC68** | **11** |
| SCS74495Z | **2** | **DPC208** | **5** | **b30956/5** | **8** | **CL4748** | **11** |
| F150 | **11** | **HK02M49310** | **14** | **CCHURM9** | **16** | **CP18** | **S** |
| SCS66797Y | **11** | **P04** | **14** | **X13** | **16** | **IHEM16346** | **S** |
| L05-343 | **12** | **CCHHCRM4** | **14** | **SCS1723982P** | **17** | **SCS119883T** | **S** |
| IHEM20415 | **12** | **HUN91** | **14** | **AM2003/0071** | **17** | **b30037/6** | **S** |
| P21-2 | **12** | **JIMS500012** | **14** | **SCS121377Y** | **17** | **CCHURM6** | **S** |
| AM2005/0383 | **12** | **CP34** | **14** | **JIMS146204** | **17** | **Bougn12** | **S** |
| S08 | **12** | **81/196** | **15** | **GUI3629** | **17** | **Bougn13** | **S** |
| DPC18 | **12** | **b30249/6** | **15** | **GUI4124** | **17** | **P2246** | **S** |
| P02 | **12** | **HK02M36442** | **15** | **GUI4609** | **17** | **L293** | **S** |
| S05 | **12** | **BougnCP04** | **15** | **DPC35** | **17** | **b30528/5** | **S** |
| JIMS500002 | **13** | **AM2003/0045** | **15** | **P07** | **17** | **HK04M215570** | **S** |
| P2257 | **13** | **AM2005/0360** | **15** | **HK04M142575** | **17** | **APRURC4** | **S** |
| HK04M2714 | **14** | **JIMS500006** | **16** | **P23-1** | **S** | **JIMS126109** | **S** |
| P10 | **14** | **HK03M120736** | **16** | **P22-1** | **S** | **Bougn22** | **S** |
| AM2005/0418 | **14** | **IHEM17984** | **16** | **P15** | **S** | **S09** | **S** |
| HK01M154213 | **14** | **P09** | **16** | **P22-3** | **S** | **IHEM20439** | **S** |
| HK04M245 | **14** | **P03** | **16** | **WI42** | **S** |  |  |
| HK03M34560 | **14** | **P19-1** | **16** | **J990681** | **S** |  |  |
| P01 | **14** | **P27** | **16** | **J981313** | **S** |  |  |
| P28-1 | **14** | **X14** | **16** | **AM2003/0026** | **S** |  |  |

**Table S3. Filamentation scores of the clinical strains**

|  | Liquid^1^ | | | | Solid^1^ | | | | Shortened solid^1^ | | | |
| --- | --- | --- | --- | --- | --- | --- | --- | --- | --- | --- | --- | --- |
| Strains | **F** | **L** | **R** | **S** | **F** | **L** | **R** | **S** | **F** | **L** | **R** | **S** |
| B1091-15 | 2.45 | 4.24 | 1.48 | 4.14 | 0.00 | 0.30 | 0.10 | 0.00 | 0.17 | 1.67 | 1.33 | 2.50 |
| B1168-15 | 3.02 | 4.84 | 3.72 | 5.66 | 0.00 | 0.00 | 0.10 | 3.80 | 1.00 | 0.00 | 1.33 | 0.17 |
| B1257-15 | 2.33 | 2.68 | 2.37 | 2.93 | 0.23 | 2.13 | 0.00 | 0.00 | 4.00 | 3.33 | 4.00 | 4.00 |
| B1486-15 | 3.22 | 4.47 | 3.10 | 5.94 | 0.43 | 0.53 | 0.00 | 0.07 | 2.83 | 3.00 | 3.67 | 4.00 |
| B1559-15 | 3.19 | 5.15 | 4.01 | 5.75 | 2.93 | 3.80 | 1.00 | 3.93 | 2.83 | 3.17 | 4.00 | 4.00 |
| B1762-15 | 3.54 | 4.32 | 3.28 | 4.38 | 0.03 | 0.00 | 0.00 | 0.07 | 4.00 | 1.83 | 3.50 | 3.33 |
| B212-12 | 2.29 | 3.50 | 3.17 | 4.84 | 0.00 | 0.07 | 0.07 | 0.53 | 3.83 | 3.00 | 3.17 | 3.83 |
| B2527-12 | 2.62 | 4.60 | 2.82 | 6.05 | 2.93 | 0.03 | 0.50 | 1.00 | 0.50 | 1.00 | 1.50 | 2.00 |
| B404-15 | 3.17 | 4.25 | 3.88 | 3.80 | 0.07 | 0.00 | 0.00 | 2.87 | 0.00 | 0.67 | 1.83 | 0.67 |
| B421-15 | 2.88 | 4.01 | 3.29 | 4.73 | 0.00 | 0.00 | 0.13 | 4.00 | 0.00 | 0.00 | 0.17 | 0.17 |
| B444-12 | 3.00 | 3.88 | 2.97 | 5.81 | 2.60 | 0.40 | 0.07 | 0.60 | 4.00 | 3.67 | 4.00 | 3.17 |
| B46-15 | 2.24 | 3.98 | 2.94 | 3.94 | 0.00 | 0.07 | 0.00 | 0.00 | 0.00 | 0.67 | 0.67 | 2.67 |
| B510-12 | 2.57 | 4.32 | 3.14 | 5.60 | 0.00 | 0.30 | 0.17 | 0.00 | 1.17 | 3.00 | 3.67 | 3.50 |
| B527-15 | 3.22 | 4.77 | 3.73 | 4.30 | 2.60 | 3.20 | 3.07 | 3.93 | 0.50 | 1.00 | 2.67 | 0.83 |
| B564-14 | 2.67 | 4.13 | 2.56 | 5.04 | 0.10 | 0.03 | 0.07 | 1.13 | 2.67 | 2.67 | 3.17 | 2.67 |
| B568-15 | 2.86 | 4.08 | 3.78 | 3.86 | 0.00 | 0.03 | 0.10 | 0.00 | 1.00 | 1.33 | 2.67 | 1.00 |
| B618-15 | 3.45 | 4.18 | 3.46 | 4.87 | 0.00 | 0.00 | 0.10 | 3.07 | 0.00 | 1.17 | 2.17 | 1.50 |
| B687-15 | 3.29 | 3.93 | 3.03 | 5.45 | 0.10 | 0.00 | 0.00 | 0.70 | 3.67 | 2.50 | 3.00 | 3.00 |
| B733-15 | 2.81 | 3.63 | 2.84 | 4.62 | 2.90 | 0.10 | 0.73 | 3.93 | 3.67 | 3.00 | 4.00 | 3.83 |
| B808-15 | 3.44 | 4.95 | 2.91 | 3.69 | 0.00 | 0.00 | 0.03 | 0.00 | 1.17 | 2.33 | 1.33 | 2.33 |
| 12C | 3.18 | 5.65 | 2.80 | 6.48 | 0.83 | 1.50 | 4.00 | 4.00 |  |  |  |  |
| 19F | 2.55 | 5.17 | 2.74 | 4.03 | 0.00 | 0.00 | 0.17 | 0.00 |  |  |  |  |
| GC75 | 3.15 | 4.07 | 2.42 | 4.27 | 0.00 | 0.00 | 4.00 | 0.00 |  |  |  |  |
| L26 | 3.54 | 4.75 | 4.21 | 4.08 | 0.00 | 2.17 | 2.67 | 1.67 |  |  |  |  |
| P34048 | 3.15 | 5.41 | 3.09 | 6.02 | 4.00 | 0.17 | 0.17 | 0.17 |  |  |  |  |
| P37005 | 3.22 | 6.11 | 4.35 | 6.15 | 0.00 | 0.00 | 0.00 | 0.33 |  |  |  |  |
| P37037 | 1.37 | 2.34 | 1.37 | 2.38 | 2.83 | 0.67 | 4.00 | 4.00 |  |  |  |  |
| P37039 | 3.43 | 6.22 | 4.58 | 6.54 | 3.00 | 4.00 | 4.00 | 4.00 |  |  |  |  |
| P57055 | 3.12 | 4.50 | 2.97 | 4.40 | 0.00 | 0.00 | 0.00 | 0.00 |  |  |  |  |
| P57072 | 3.30 | 5.10 | 3.59 | 5.93 | 0.00 | 0.00 | 0.17 | 0.00 |  |  |  |  |
| P60002 | 3.22 | 6.00 | 4.70 | 6.70 | 0.17 | 2.67 | 4.00 | 0.00 |  |  |  |  |
| P75010 | 2.51 | 5.18 | 4.04 | 5.62 | 0.00 | 0.00 | 0.17 | 0.00 |  |  |  |  |
| P75016 | 2.76 | 3.37 | 2.37 | 3.50 | 0.00 | 4.00 | 4.00 | 1.00 |  |  |  |  |
| P75063 | 1.40 | 2.98 | 2.37 | 3.20 | 0.00 | 0.00 | 0.83 | 0.00 |  |  |  |  |
| P76055 | 3.62 | 5.56 | 4.03 | 6.26 | 0.00 | 2.67 | 1.17 | 0.00 |  |  |  |  |
| P76067 | 3.63 | 5.20 | 3.90 | 6.97 | 1.50 | 1.33 | 2.00 | 1.17 |  |  |  |  |
| P78042 | 3.66 | 3.46 | 2.44 | 2.95 | 4.00 | 4.00 | 0.00 | 0.50 |  |  |  |  |
| P78048 | 3.18 | 5.55 | 3.50 | 4.41 | 0.17 | 0.00 | 0.00 | 1.67 |  |  |  |  |
| P87 | 3.24 | 5.55 | 4.30 | 6.71 | 1.67 | 0.00 | 0.17 | 0.00 |  |  |  |  |
| P94015 | 0.85 | 1.02 | 0.49 | 1.09 | 0.00 | 0.00 | 0.00 | 0.00 |  |  |  |  |

^1^F-FBS media, L- Lee’s Media, R- RPMI media, S-Spider media
